# A SNP Based High-Density Linkage Map of *Apis cerana* Reveals a High Recombination Rate Similar to *Apis mellifera*

**DOI:** 10.1371/journal.pone.0076459

**Published:** 2013-10-10

**Authors:** Yuan Yuan Shi, Liang Xian Sun, Zachary Y. Huang, Xiao Bo Wu, Yong Qiang Zhu, Hua Jun Zheng, Zhi Jiang Zeng

**Affiliations:** 1 Honeybee Research Institute, Jiangxi Agricultural University, Nanchang, Jiangxi, China; 2 Molecular Biology and Pharmacology Key Laboratory of Fujian Advanced Education, Quanzhou Normal University, Quanzhou, Fujian, China; 3 Department of Entomology, Michigan State University, East Lansing, Michigan, United States of America; 4 Shanghai-MOST Key Laboratory of Health and Disease Genomics, Chinese National Human Genome Center at Shanghai, Shanghai, China; The Ohio State University/OARDC, United States of America

## Abstract

**Background:**

The Eastern honey bee, *Apis cerana* Fabricius, is distributed in southern and eastern Asia, from India and China to Korea and Japan and southeast to the Moluccas. This species is also widely kept for honey production besides *Apis mellifera*. *Apis cerana* is also a model organism for studying social behavior, caste determination, mating biology, sexual selection, and host-parasite interactions. Few resources are available for molecular research in this species, and a linkage map was never constructed. A linkage map is a prerequisite for quantitative trait loci mapping and for analyzing genome structure. We used the Chinese honey bee, *Apis cerana cerana* to construct the first linkage map in the Eastern honey bee.

**Results:**

F2 workers (N = 103) were genotyped for 126,990 single nucleotide polymorphisms (SNPs). After filtering low quality and those not passing the Mendel test, we obtained 3,000 SNPs, 1,535 of these were informative and used to construct a linkage map. The preliminary map contains 19 linkage groups, we then mapped the 19 linkage groups to 16 chromosomes by comparing the markers to the genome of *A. mellfiera*. The final map contains 16 linkage groups with a total of 1,535 markers. The total genetic distance is 3,942.7 centimorgans (cM) with the largest linkage group (180 loci) measuring 574.5 cM. Average marker interval for all markers across the 16 linkage groups is 2.6 cM.

**Conclusion:**

We constructed a high density linkage map for *A. c. cerana* with 1,535 markers. Because the map is based on SNP markers, it will enable easier and faster genotyping assays than randomly amplified polymorphic DNA or microsatellite based maps used in *A. mellifera*.

## Introduction

The Eastern honey bee, *Apis cerana* Fabricius, is distributed in southern and eastern Asia, from India and China to Korea and Japan and southeast to the Moluccas [1]. The Chinese honey bee, *Apis cerana cerana* (ACC) is an important subspecies of *A. cerana* and is endemic in China. Based on morphology and bionomics analysis, ACC can be divided into nine geographical subtypes: Northern China, Southern China, Central China, Yungui Plateau, Changbaishan, Hainan, Aba, Diannan and Tibetan ACC [2].

*A. cerana* is thought to be the closest related honey bee species to *A. mellifera* and shares many biological traits [3]. For example, both are cavity nesting, and in both species the queens cannot fly when laying eggs, in contrast to *Apis dorsata* and *Apis florea*, who nest in the open and queens can fly any time (Z.Y. Huang, personal observation). The two species also have many contrasting biological traits [4]–[12] (Table 1). The most important one is the ability of *A. cerana* to resist *Varroa destructor*. Genomic studies on *A. cerana* might help us find genes responsible for mite resistance and provide more information on the relationship between *A. cerana* and *A. mellifera*.

**Table 1:** Differences in biological traits of *A. mellifera* and *A. cerana.*

Biological traits	*A. mellifera*	*A. cerana*
**Adapted to foraging in**	Large patches of monocrop [4]	Scattered floral resources [4]
**Balling as a defense against hornets**	Weak [5]	Strong [6]
**Collection of** propolis	Yes [7]	No [7]
**Direction of fanning**	Outward from entrance [8]	Inward toward entrance [8]
**Drone cell capping**	Flat, without a hole [9]	Conical with a hole [9]
**Minimum foraging temperature**	14°C [8]	8°C [8]
**Olfactory sense**	Not as sensitive [8]	Sensitive [8]
**Onset of laying workers in queenless** **and broodless colonies**	>6 days [10]	2–3 days [11]
**Resistance to ** ***Varroa destructor***	No [12]	Yes [12]

Numbers in [] are references.

Many genomic resources are available for *A. mellifera*, for example, EST libraries [13], [14], linkage maps [15]–[19], and the complete genome [20]. High density linkage maps are important tools for studying genomic organization [21], [22], identifying gene functions [23] and constructing high-resolution quantitative trait loci (QTL) maps. In addition, linkage maps are indispensable to facilitate genome assembly for full genome sequencing [17]. Linkage maps have been developed for *Caenorhabditis elegans* [24], *Drosophila melanogaster* [25], *Bombyx mori* [26], *Anopheles gambiae* [27], *A. mellifera* [15]–[19], and *Bombus terrestris* [28]. The first linkage map of *A. mellifera,* based on randomly amplified polymorphic DNAs (RAPD), enabled the discovery of a large number of recombination sites [15]. A second linkage map of *A. mellifera* made with microsatellites detected segregation distortion and a low positive interference [16]. The third map, also based on microsatellites, showed that the high recombination rate in honey bees is homogeneous within the nucleus (along and across chromosomes), within individuals and also among individuals; but numerous recombination hotspots are dispersed over the genome [17]. Two most recent high-resolution linkage maps of *A. mellifera*, based on single nucleotide polymorphisms, analyzed the association of the genotypes with grooming behavior [18] and with the performance of *Varroa* sensitive hygiene [19].

However, there are currently few genomic resources available for *A. cerana*. The genome project for *A. cerana* is still in progress. Several transcriptome studies were conducted in this species [29], [30]. However no linkage maps have yet been constructed for *A. cerana*.

Single nucleotide polymorphisms (SNPs) refer to DNA sequence polymorphism that is caused by a single nucleotide variation on a genome-wide scale. SNP markers are not only important for genome research but can also be the basis of constructing linkage maps. SNP based linkage maps are better than earlier maps based RAPD, amplified fragment length polymorphism (AFLP) or mutant phenotypes, because SNP based maps include sequence information of potentially conserved flanking regions, allowing anchoring genome assemblies and for comparisons among species [31]. SNP based linkage maps have been constructed for chicken [32], turkey [33], *Drosophila* [34], *C. elegans* [35] and *A. mellifera* [18], [19]. In this study, we constructed a SNP based linkage map for *Apis cerana* from the progeny of two hybrid queens between Central China ACC (Nanchang, Jiangxi) and Diannan ACC (Honghe, Yunnan).

## Results

### 1. Sequencing and SNP Identification

A total of 110 ACCs, including one F0 queen, two F1 hybrid queens, four F1 drones and 103 F2 worker bees from two colonies, was sequenced for further SNP identification. The sequencing depth in the 110 samples ranged from 15 X to 50 X. The sequencing coverage in the 110 samples was greater than 90%. After mapping to ACC genome, the percentage of mapping was greater than 81% (Table 2). The sequencing depth, sequencing coverage, and percentage of mapping for each sample are presented in Table S1.

**Table 2:** Number of individuals (N), sequencing depth, coverage, and percentage of mapping for F0, F1, F2.

Pedigree	N	Sequencing depth	Sequencing coverage (%)	Mapping rate (%)
**F0**	1	50 X	91.6	84.7
**F1**	6	43 X	91.5	82.3
**F2**	103	15 X	90.9	81.5

After removing SNPs whose call rates were lower than 95%, 330,701 SNPs were preserved. From these, 126,990 SNPs remained after removing those whose call rates were smaller than or equal to 99%. Only SNPs whose three genotypes AA, AB, and BB grouped within clear distinct clusters were considered to be reliable. 3,000 SNPs were selected for map construction after passing the Mendel test based on the primary honey bee pedigree. The SNP markers name and their 100 bp upstream and downstream sequences are presented in Table S2.

### 2. Linkage Groups and Genetic Length

We constructed 16 linkage groups (Fig. S1), corresponding to the 16 chromosomes in ACC. Among these 16 groups, three were very short (134.5–163.2 cM), each with 57–69 markers. The 13 other groups were longer (190.3–574.5 cM, with 62–180 markers). The average density of markers on the map was about one every 2.6 cM. A higher density was observed for group 7 (one marker every 2.1 cM) and 14 (2.1 cM) whereas groups 1 and 8 had a lower density (3.2 and 3.4 cM, respectively).

### 3. Differences between Linkage Maps of *A. mellifera* and *A. cerana*

The lengths and markers of linkage maps for *A. mellifera* and *A. cerana* are shown in Table 3. The length of *A. cerana* map is 3,942.7 cM, slightly lower than that of *A. mellifera* (4,114.5 cM). The number of markers in *A. cerana* map is 1,535, more than one-half that of *A. mellifera* (2,008). The average density of markers on *A. cerana* and *A. mellifera* maps is about one in every 2.6 cM and 2.0 cM, respectively.

**Table 3:** Genetic length (cM) and number of markers of linkage maps in *A. mellifera* and *A. cerana.*

	LG 01	LG 02	LG 03	LG 04	LG 05	LG 06	LG07	LG08
***A. mellifera***	575.9 (273)	321.9 (143)	276.9 (137)	290.9 (115)	263.5 (121)	305.7 (139)	237.9 (117)	224.3 (112)
***A. cerana***	574.5 (180)	315.9 (115)	276.2 (109)	295.2 (128)	267.0 (88)	298.5 (131)	240.5 (114)	212.1 (62)
	**LG 09**	**LG 10**	**LG 11**	**LG 12**	**LG 13**	**LG 14**	**LG 15**	**LG 16**
***A. mellifera***	220.3 (105)	232.5 (124)	223.4 (125)	219.3 (100)	197.7 (95)	208.1 (107)	184.0 (112)	138.0 (83)
***A. cerana***	205.1 (96)	217.5 (91)	208.5 (81)	195.3 (66)	163.2 (57)	190.3 (90)	148.4 (69)	134.5 (58)

Numbers outside ( ) are genetic length and those in ( ) are number of markers. Data for *A. mellifera* was from Solignac *et al.* 2007. Both species have the linkage groups mapped to 16 chromosomes.

### 4. High Recombination Rate in *A. cerana* Genome

Using a genome size of 226 Mb (ACC genome sequencing consortium, unpublished data), we calculated the recombination rate in ACC as 17.4 cM/Mb.

## Discussion

*A. cerana* is an important species of honeybee and is widely distributed throughout Asia. It is widely-kept in China, with more than two million colonies, and brings substantial economic benefits to beekeepers [1], [2]. To produce more informative loci, we used crosses between Central and Dianna ACC, which is about 2,000 km apart.

### 1. High Recombination Rate in *A. cerana* Genome

Recombination rate in ACC from this study was found to be 17.4 cM/Mb, which is comparable to published studies on *A. mellifera*. We recalculated the recombination rate for *A. mellifera* based on the first and second linkage map [15], [16], because the correct honey bee genome size is 238 Mb (instead of the assumed 178 at the time of publication). The adjusted recombination rates are 14.5 and 17.1 cM/Mb, respectively for the two studies. Solignac et al. [17] found a recombination rate of 22.0 cM/Mb for *A. mellifera* based on the third-generation linkage map. Similar to the Solignac’s results, Tsuruda et al. [19] found a slightly higher (22.6 cM/Mb) recombination rate of *A. mellifera* linkage map using SNP markers. The recombination rate for ACC, therefore, is very similar to that of *A. mellifera*, and both are higher than other organisms [28], [36].

Menzar et al. (2010) studied two chromosomes of *Apis florea* and found that *A. ﬂorea* exhibited a similar genomic recombination rate to *A. mellifera* [36]. Because *A. florea* is basal in the genus *Apis* [37], Menzar et al. suggested that the high recombination rate is shared in all honey bee species in the genus *Apis*. Our results are consistent with and strengthen this hypothesis, because our data is based on the entire genome and also provides data for a third species.

Several hypotheses have been proposed to explain the high recombination rate in the honey bee *A. mellifera* (reviewed in Menzar 2010). Wilfred (2007) provided evidence against the hypothesis that the exceptionally high recombination rate in *A. mellifera* may be the result of domestication [38], [39]. This is because an ant *Pogonomyrmex rugosus*, which has not experienced domestication, also showed high recombination rate, while *Bombyx mori* which did so, had a low recombination rate [28], [39]. Our results here also are consistent with Wilfred (2007), because ACC in China experienced some breeding and selection, but the extent of which is much smaller compared to *A. mellifera* [1], [2].

### 2. Linkage Map of *A. cerana*

The Mendel test is that genetic characters of offspring depended on heredity of both parents. A honey bee colony is composed of diploid females (queen and workers) and haploid males (drones). If a heterozygous diploid queen (Aa) is crossed with a haploid drone (B), each parent will make gametes of only one kind. According to the Mendel test, the phenotype of female progeny is AB or aB. In this study, 3,000 SNP markers we selected passed the Mendelian test.

The first linkage map for *Apis cerana* we constructed here was done with SNP markers, which is more repeatable compared with maps based RAPD and microsatellites (Table 4).

**Table 4:** Differences between linkage maps of *A. mellifera* and *A. cerana.*

	Types ofmarkers	No. ofmarkers	No. of linkagegroups	Genetic length(cM)	Recombination rate(cM/Mb)
*A. mellifera* (Hunt et al. 1995)	RAPD	365	26	3,450.0	14.5
*A. mellifera* (Solignac et al. 2004)	Microsatellites	474	24	4,061.0	17.1
*A. mellifera* (Solignac et al. 2007)	Microsatellites	2,008	16	4,114.5	22.0
*A. mellifera* (Arechavaleta-Velasco et al. 2012)	SNP	1,313	–	–	–
*A. mellifera* (Tsuruda et al. 2012)	SNP	1,340	–	–	22.6
*A. cerana* (This study)	SNP	1,535	16	3,942.7	17.4

We initially had 19 linkage groups, but through comparing the markers with the *A. mellifera* genome, we successfully mapped all 19 linkage groups to 16 chromosomes, with three chromosomes (chr. 9, 13 and 15) receiving two linkage groups and others one each. That might be caused by low mutation rate of single nucleotides in the region between two linkage groups. According to the matching results, the final *A. cerana* genetic map contains 16 linkage groups.

The map we constructed will be indispensable for QTL mapping because the average genetic distance is 2.6 cM. In some regions, the distance is <1 cM which can be use for gene cloning. Further, our map will greatly facilitate the *Apis cerana* genome project which is underway. Future studies should be tried to screen more SNPs to make the genetic markers more evenly distributed.

### 3. The Mapping Population of *A. cerana* Genetic Map

Group size is the most important factor affecting the accuracy and applications range of genetic map. Construction of a high-precision genetic map was based on large population, group type, parental heterozygosities, density of genetic markers etc. A suitable mapping population could adopt a small random population from large groups [40]. Judged from *A. mellifera* genetic maps, these mapping population includes at least two families and 100 individuals. Hunt used 142 drone progeny of one F1 daughter queen as experimental material to construct a RAPD-based *Apis mellifera* linkage map [15]. Two microsatellite-based *Apis mellifera* linkage maps were constructed that based on 192 and 187 worker bees of two F1 hybrid queens, respectively [16], [17]. Two maps with over a thousand SNP markers were produced with large family sizes (196 and 240 individual workers of two F1 queens, severally) [18], [19].

In this study, we constructed a SNP based linkage map of *Apis cerana* by an intercross between Central China ACC and Diannan ACC. The family tree contained three generations, which were F0 (3 Diannan ACC queens and 36 Central China ACC drones), F1(20 F1 hybrid queens, 20 Diannan ACC drones and 20 Central China ACC drones), F2 (workers of these backcrosses). To construct a linkage map, we extracted genomic DNA from one F0 queen, two F1 hybrid queens, four F1 drones and 103 workers of two backcrosses, and performed genomic DNA sequencing. Future studies should try to increase the number of worker samples to make the *Apis cerana* linkage map more saturated.

### General Conclusions

We constructed a high density linkage map for *A. c. cerana* with 1,535 markers and a genetic length of 3,942.7 cM. Because the map is based on SNP markers, it will enable easier and faster genotyping assays than RAPD or microsatellite based maps used in *A. mellifera*. In addition, our map will greatly facilitate the *Apis cerana* genome project which is underway.

## Materials and Methods

### 1. Ethics Statement

*Apis cerana cerana* (ACC) is widely-kept and raised in China, with more than 2 million colonies, and brings substantial economic benefits to beekeepers. Both Central China ACC and Diannan ACC were sampled from local beekeepers, and we got the oral permission of beekeepers.

### 2. Experimental Population and DNA Isolation

**Figure 1:**
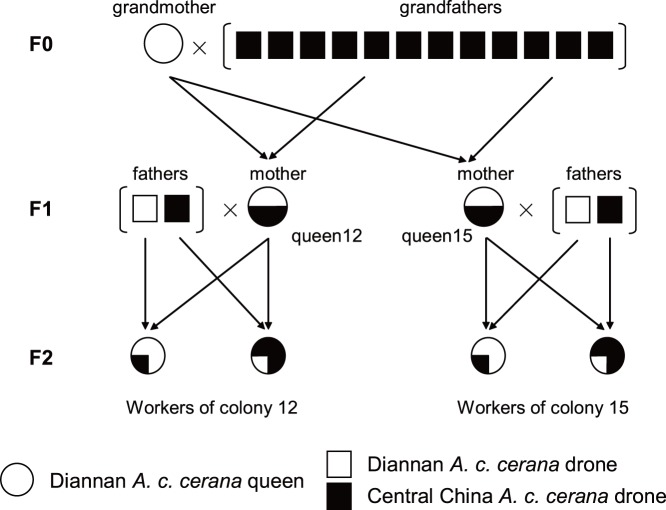
An intercross population performed to obtain workers for map construction. The grandmother was Diannan ACC queens (white, circle) and it was instrumentally inseminated with the sperm admixture from 12 drones (grandfathers) of Central China ACC drones (black, square). Two hybrid queens 12 and 15 (mothers, black/white, circle), were backcrossed to two males (the fathers), one belonging to Diannan ACC and the other one Central China ACC. The female progeny (workers) of these backcrosses were collected from two families (queens 12 and 15).

We followed the method of Solignac et al (2004) to obtain two families ACC samples. In order to maximize the number of informative loci, we used queens that were cross-bred between two different geographical strains of ACC (Central China ACC and Diannan ACC). Twenty F1 hybrid queens were developed from a cross between F0 Diannan ACC queens (Honghe, Yunnan) and Central China ACC drones (Nanchang, Jiangxi), these hybrid queens were named with a sequencial number (1–20). The distance between Honghe and Nanchang is about 1,933 km. Virgin Diannan ACC queens (the grandmothers) were instrumentally inseminated with the mixed sperm from 12 Central China ACC drones (the grandfathers). These twenty F1 hybrid queens (the mothers) were instrumentally inseminated with the mixed sperm from two males (the fathers), one belonging to Diannan ACC and the other Central China ACC. The female progeny (workers) of these backcrosses were randomly collected from two families (F1 hybrid queens 12 and 15) (Fig. 1). The honey bee colonies were maintained at the Honeybee Research Institute, Jiangxi Agricultural University, Nanchang, China (28.46°N, 115.49°E), according to standard beekeeping techniques. After inseminating, the bodies of the males (grandfathers and fathers) were preserved in 95% alcohol.

To obtain suitable SNP markers, we extracted genomic DNA from one F0 Diannan ACC queen (the grandmother) and 12 Central China ACC drones (the grandfathers). For the two families (12 and 15), DNA extracts from one F1 hybrid queen (the mother), two drones (the fathers) and 103 workers (female progeny) were prepared from the heads and thoraces. DNA was isolated by Universal Genomic DNA Extraction Kit (TaKaRa, DV811A). DNA was diluted to 1/40 and preserved at −80°C in 96-well microtitration plates.

### 3. Multiplexed Sequencing by Illumina Hiseq2000

DNA samples were analyzed by the Chinese National Human Genome Center at Shanghai with the Illumina HiSeqTM 2000 (Illumina Inc, CA, USA). 2 µg genomic DNA from each sample were sheared to 200–400 bp and A-tailed after end-blunting. Illumina paired-end adapters (Illumina, San Diego, CA) were then ligated, and ligated fragments of 300–500 bp were size selected on agarose gel. Then a low-cycle PCR was performed to obtain the 300 bp pair-end library. On average 8–10 samples with different library indexes were combined in one lane, and cluster generation was performed in C-bot and sequencing was performed on the Illumina Hiseq2000 with 200 cycles. The fastq data were transformed to the Sanger format, followed by trimming off adaptors and low quality bases at the 3′ ends by Scythe (https://github.com/ucdavis-bioinformatics/scythe) and Sickle (https://github.com/ucdavis- bioinformatics/sickle), respectively. Reads alignment was performed by Burrows-Wheeler Aligner [41]. After duplicate marking and local realignment by GATK [42], the software Samtools [43] was used to convert aligned reads into pileup-formatted files. To ensure the reliability of subsequent analysis, we removed reads containing adaptor sequence, loci with the lowest sequencing depth and loci with depth higher than twice the average level. In total, bases covering about 90% of the *A. cerana* genome were included for further analysis.

### 4. Analysis of Sequencing Data and SNP Calling

Base calling and sequence reads quality assessment was performed using Illumina’s Data Analysis Pipeline software v.1.6. Alignment of sequence reads to the reference Chinese honey bee genome (data not shown) was performed using Bowtie 2 (version 2.0.0-beta6) [44]. The mapping results were processed with SAM tools [43]. Raw SNPs were called using the mpileup command of the SAM tools package on all F0/F1/F2 samples. Clean SNPs were obtained by removing SNPs whose call rates were lower than 95%, then removing those which failed the Mendel test based on the primary honeybee pedigree. Paternity testing was performed to identify uncertain drone parents of F2 worker bees using the first clean SNPs by maximum likelihood test.

### 5. Linkage Analysis and Map Construction

We followed the method of Guo et al (2009) to construct the ACC linkage map [45]. The linkage map constructed by CRI-MAP version 2.4 [46]. The option TWOPOINT from CRI-MAP was responsible for allowing the markers to be clustered into linkage groups and determining the linked pairs of markers with LOD scores higher than three. With the option TWOPOINT, 1,535 of 3,000 SNP markers were clustered into linkage groups and determined the linked pairs of markers with LOD scores higher than three. The option BUILD was performed to construct the framework map for markers in the same linkage group [47]. The option ALL was used to incorporate the remaining markers into the framework map. The option FLIPS was run to improve the linkage map. The CHROMPIC and the SIMWALK2 package were used to scrutinize the genotype data [48]. The option BUILD was used to establish the final linkage map [47]. With the option BUILD, ALL and FLIPS, we constructed a linkage map of ACC that contained 1,535 markers and 16 linkage groups. Then, the genotype data of 1,535 markers was scrutinized by CHROMPIC and the SIMWALK2 package. The linkage map was drawn in MapChart 2.2 software [49].

We mapped the SNP markers to chromosomes by comparing 500 bp upstream and downstream of every SNP marker with *A. mellifera* genome by using Basic Local Alignment Search Tool (Blast). With this method we successfully mapped all 19 linkage groups to 16 chromosomes, with three chromosomes (chr. 9, 13 and 15) receiving two linkage groups and others one each.

## Supporting Information

Figure S1 **A SNP based linkage map for the Eastern honey bee (** ***A. c. cerana*** **).** The map is composed of 1535 markers and 16 linkage groups (1–16), 13 major groups and 3 minute ones. The names of SNP markers are shown on the right, and the positions of the markers are shown in Kosambi centiMorgan (cM) on the left.
(PDF)

Table S1 **The sequencing depth, sequencing coverage, percentage of mapping for each individual.**
(DOC)

Table S2 **The SNP markers name and 100 bp upstream and downstream sequence of every SNP marker.** Each of the SNP markers starts with the greater than sign (>). Bases in [reference base/alterative base] are base mutation of each SNP markers. The sequence in front of [] is 100 bp upstream sequence of each SNP markers, and the sequence behind [] is 100 bp upstream sequence of each SNP markers.
(DOC)
