# Supplementary figures and images for: A SNP Based High-Density Linkage Map of Apis cerana Reveals a High Recombination Rate Similar to Apis mellifera
Source: PLoS One. 2013 Oct 10;8(10):e76459. doi: 10.1371/journal.pone.0076459 (PMC3794977; doi:10.1371/journal.pone.0076459)

1 [1]

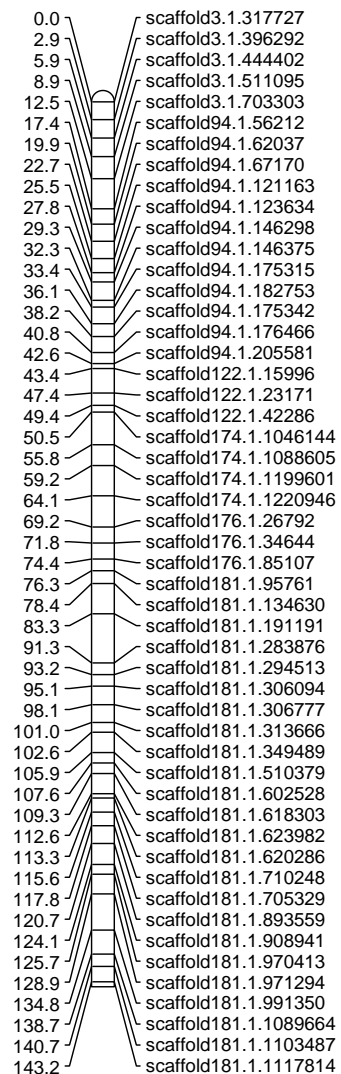

1 [2]

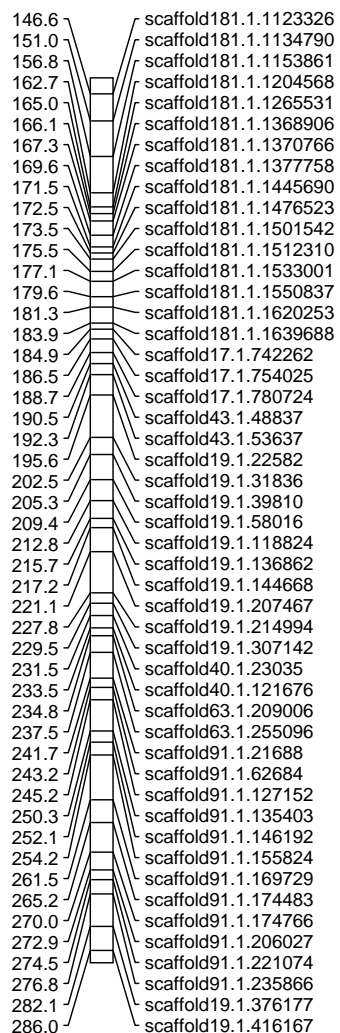

1 [3]

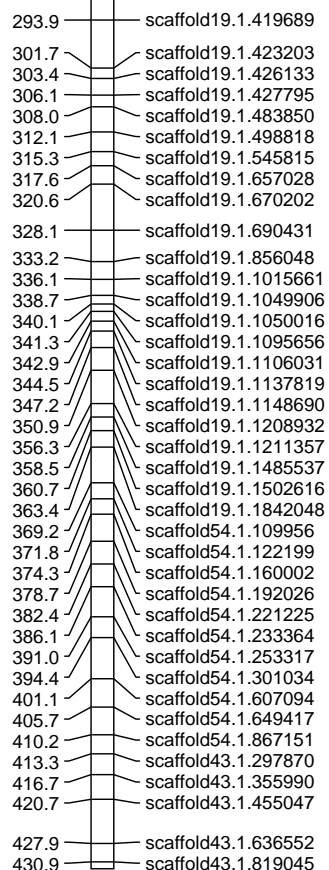

1 [4]

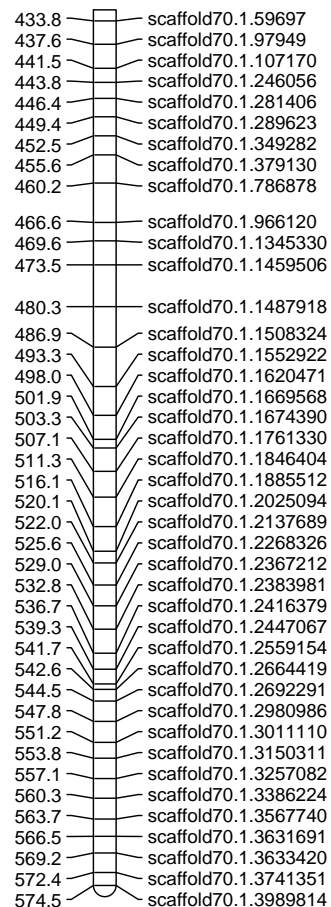

## 2 [1]

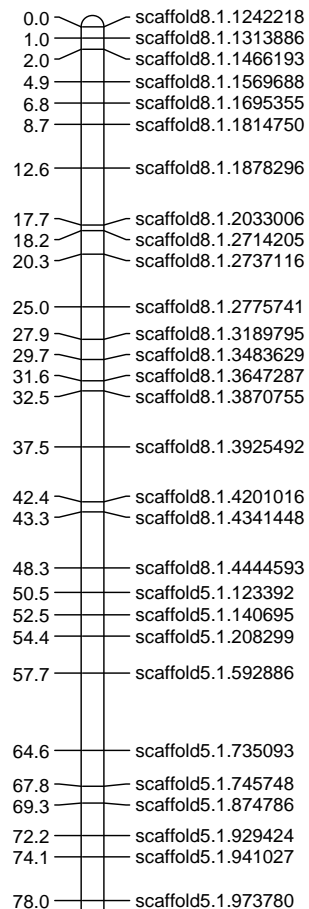

## 2 [2]

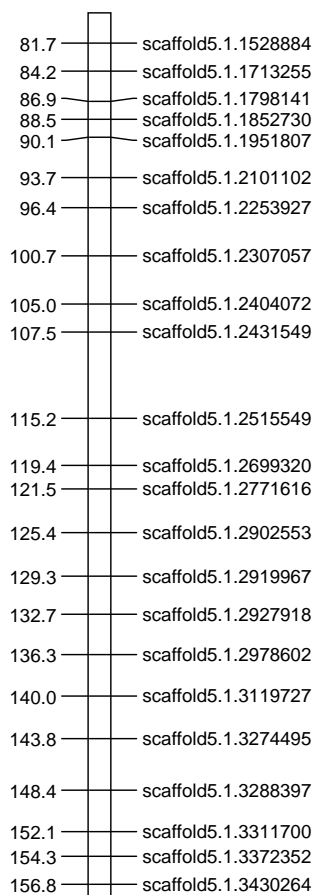

## 2 [3]

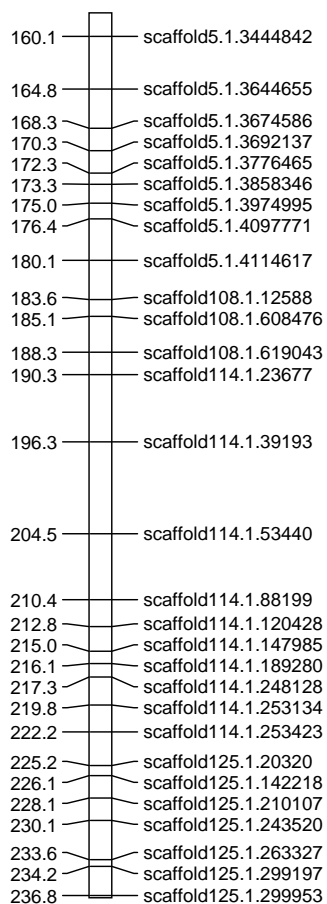

## 2 [4]

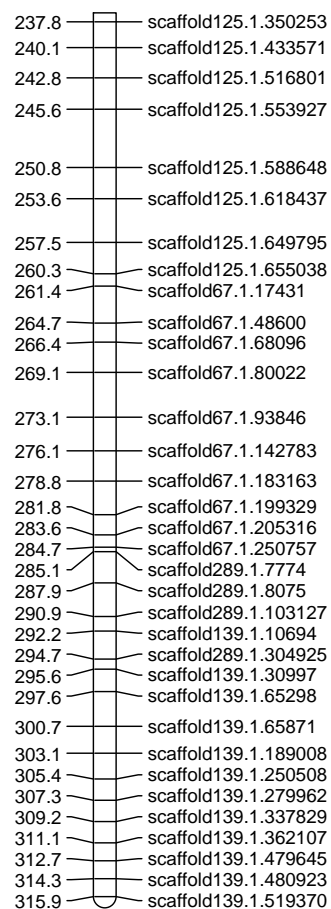

## 3 [1]

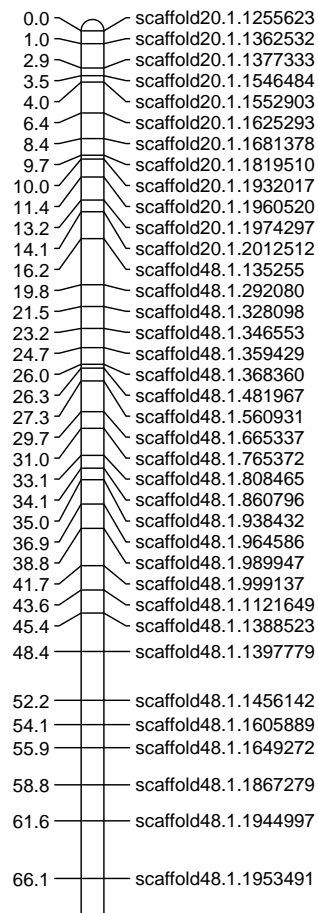

## 3 [2]

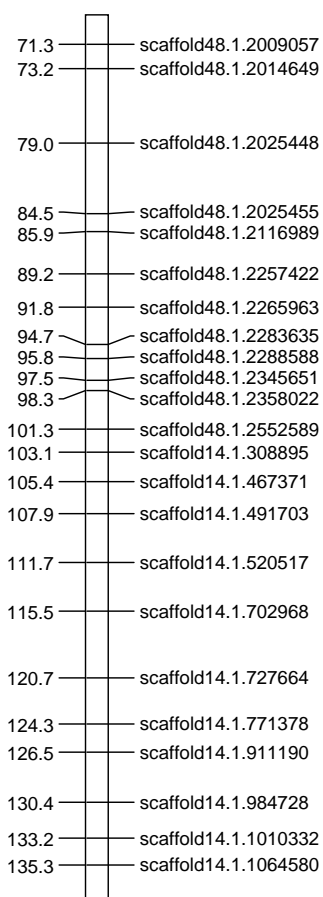

## 3 [3]

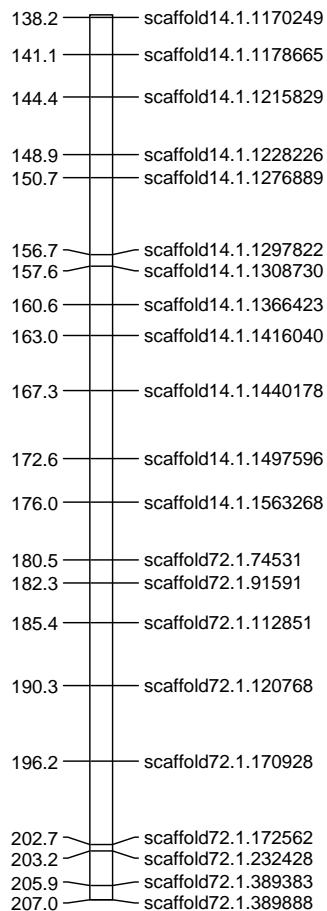

## 3 [4]

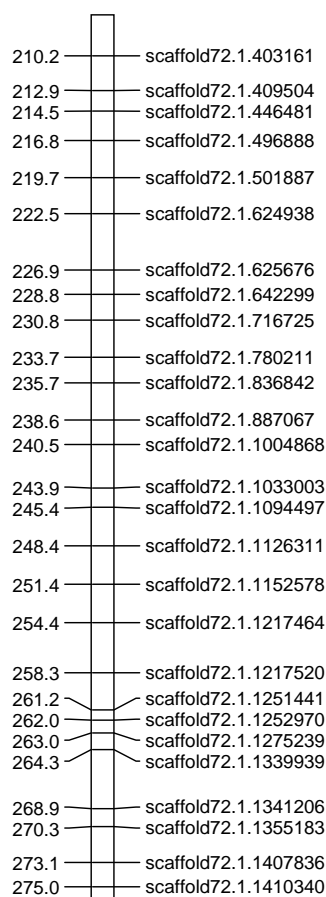

## 3 [5]

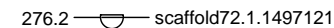

## 4 [1]

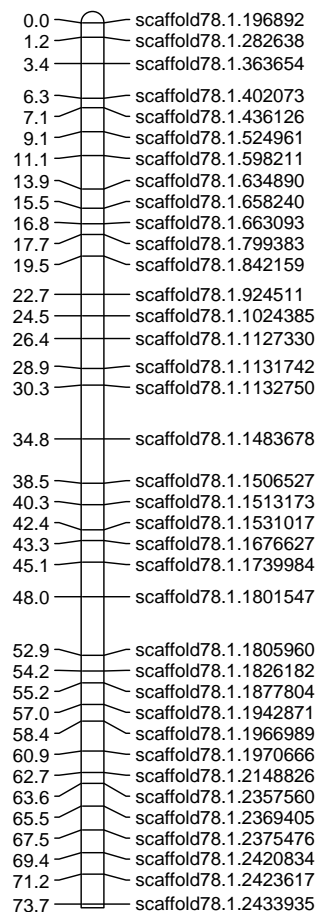

## 4 [2]

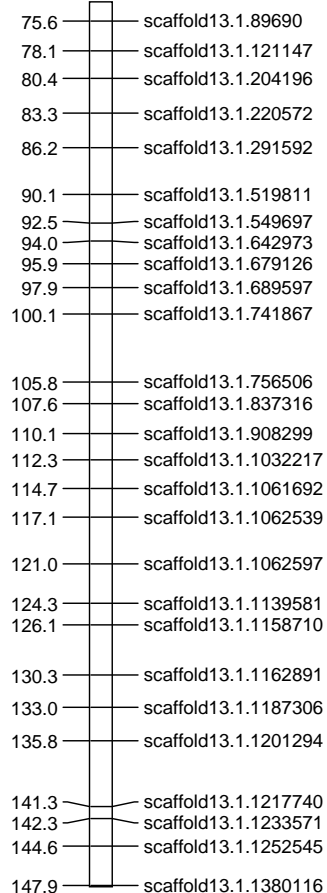

## 4 [3]

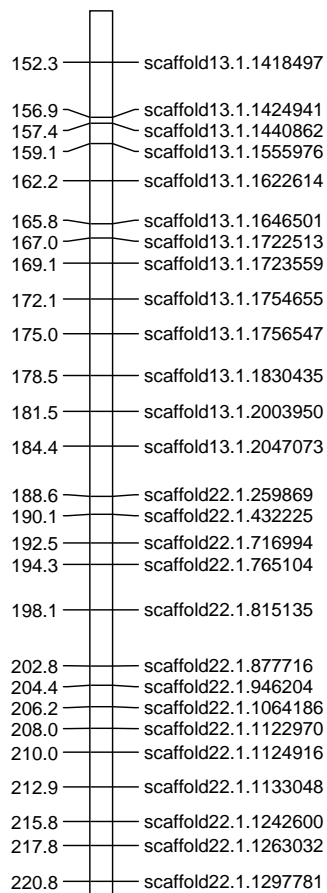

## 4 [4]

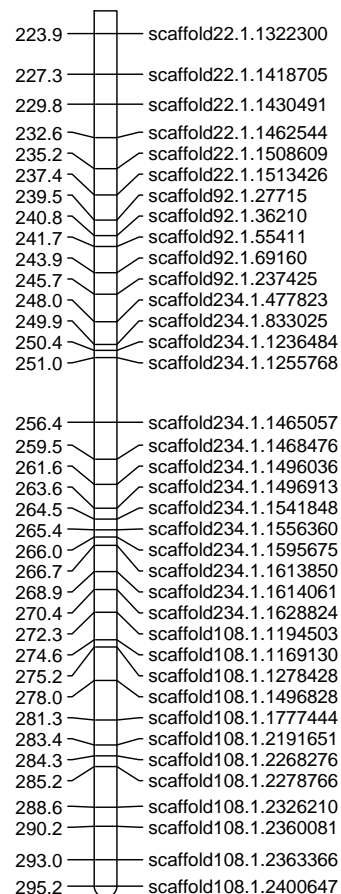

## 5 [1]

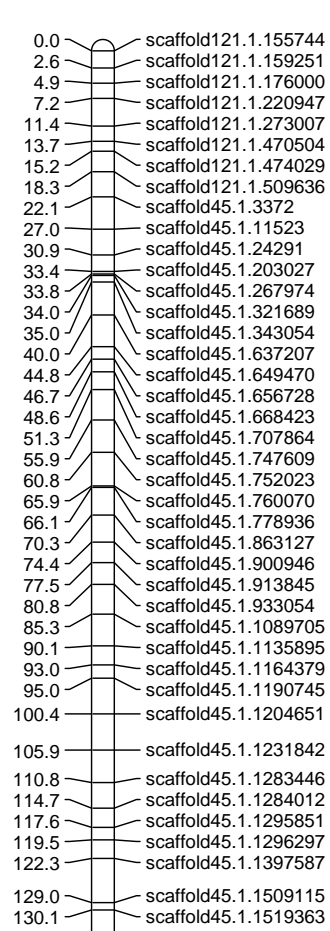

## 5 [2]

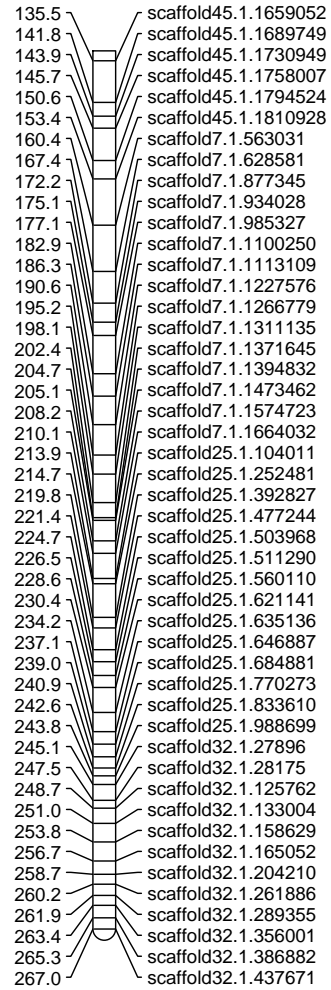

6 [1]

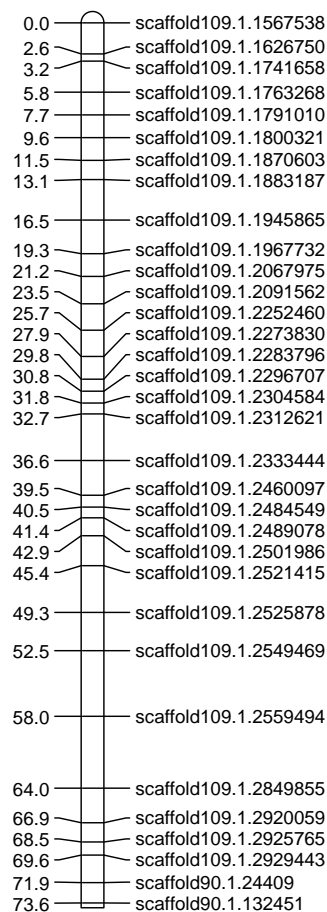

6 [2]

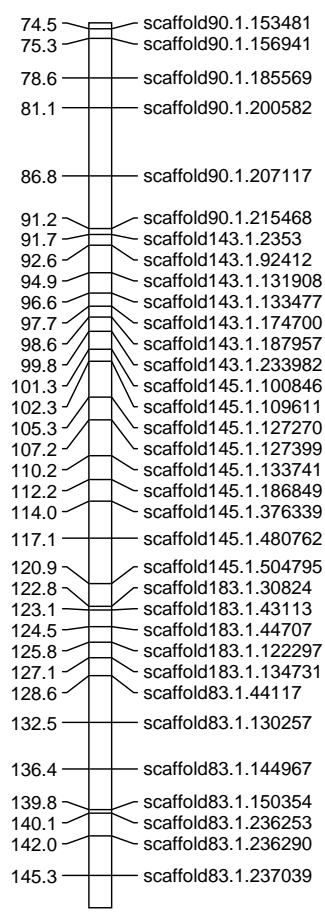

6 [3]

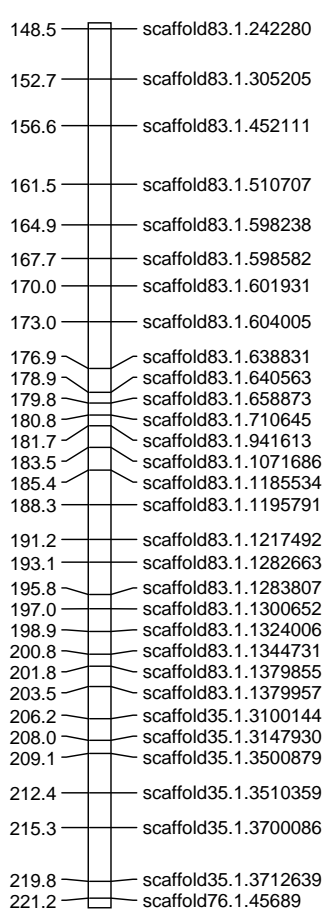

6 [4]

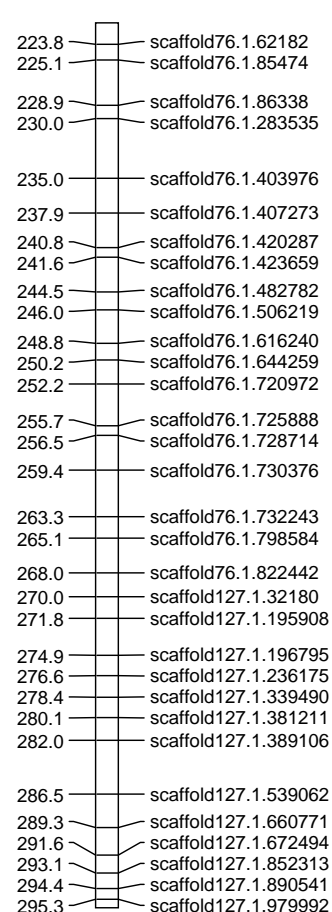

6 [5]

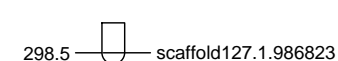

7 [1]

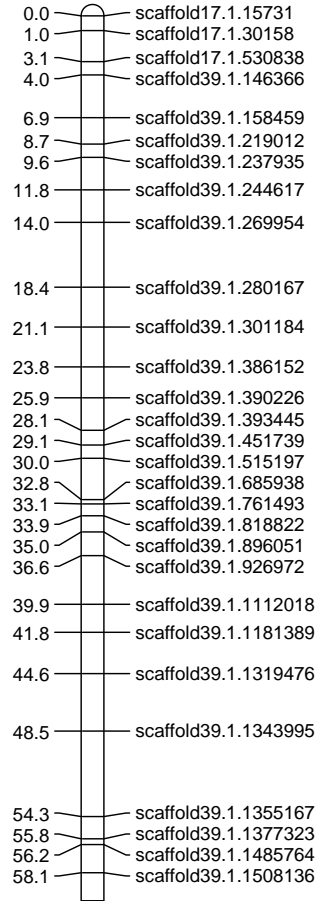

7 [2]

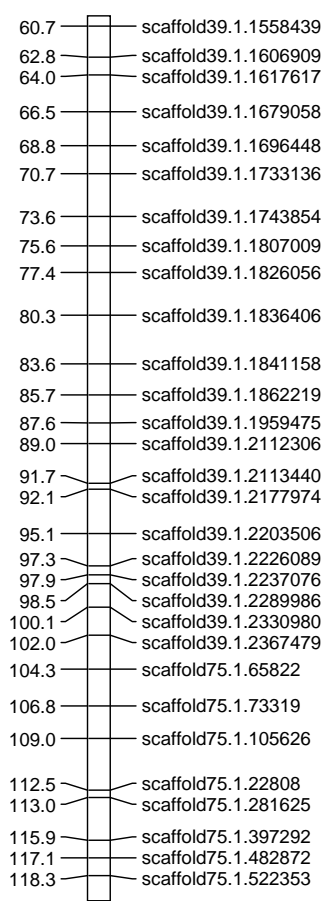

7 [3]

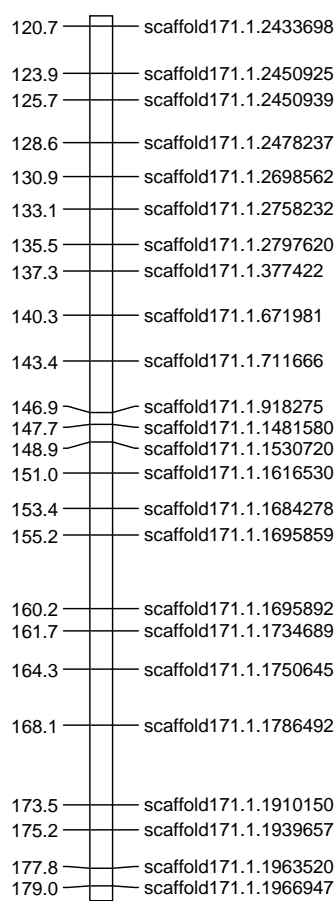

7 [4]

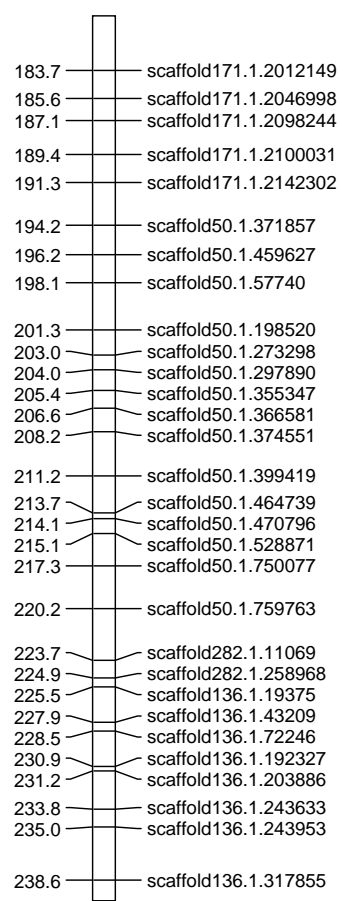

7 [5]

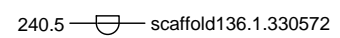

# 8 [1]

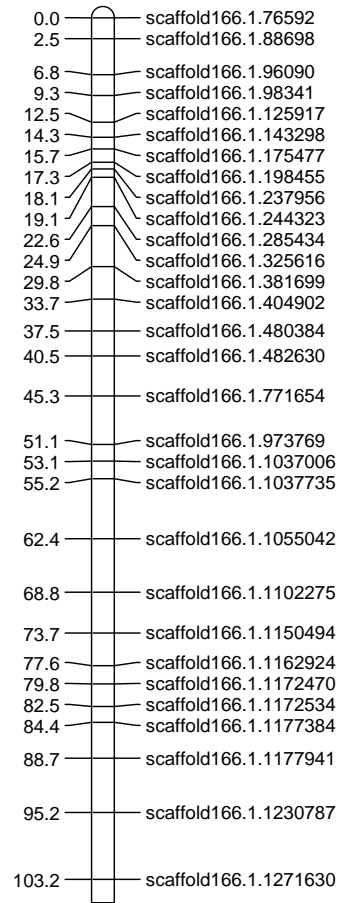

# 8 [2]

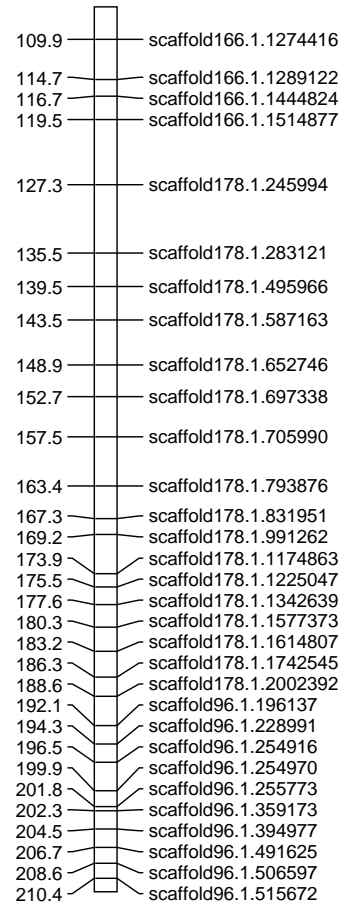

# 8 [3]

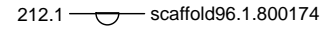

## 9 [1]

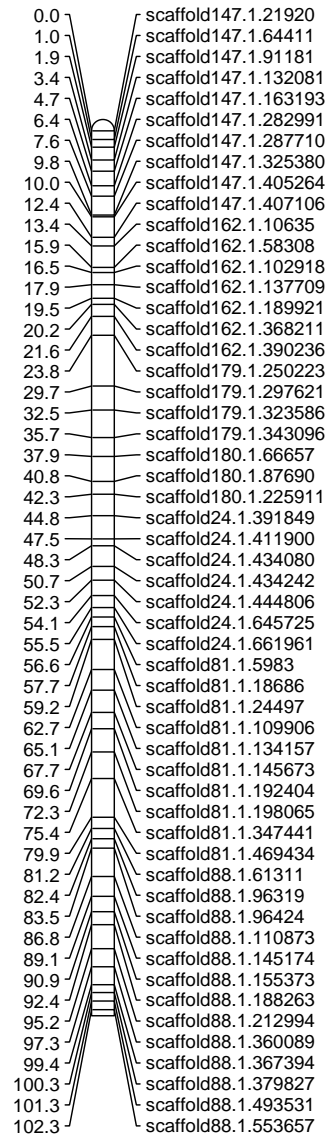

## 9 [2]

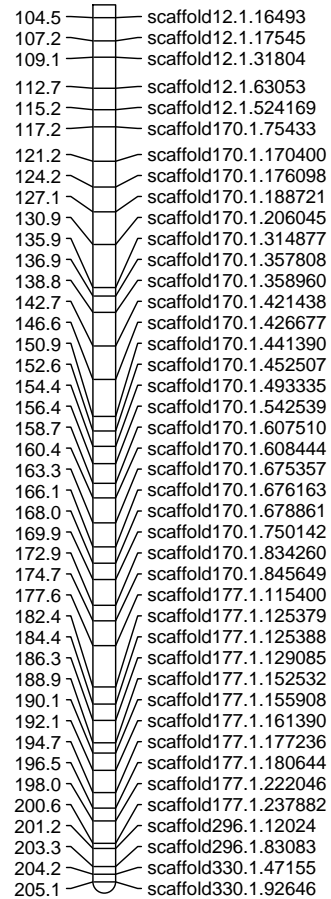

# 10 [1]

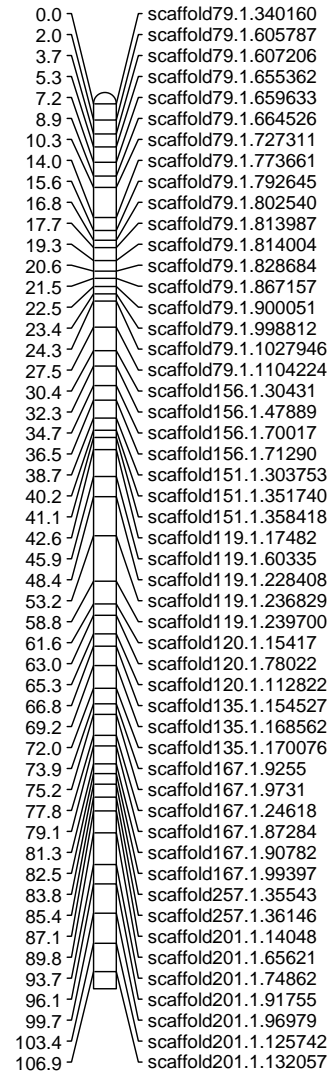

# 10 [2]

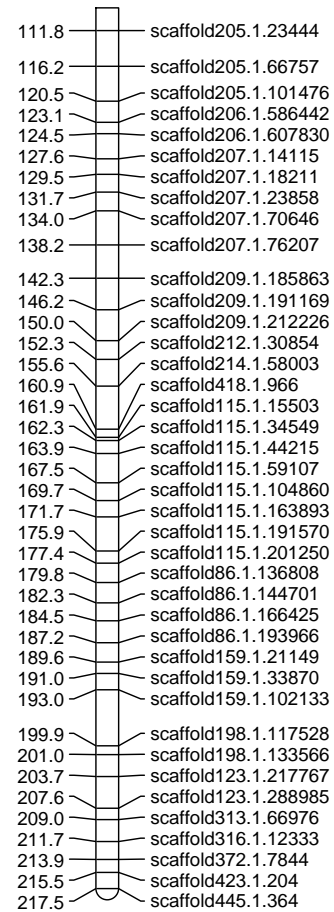

# 11 [1]

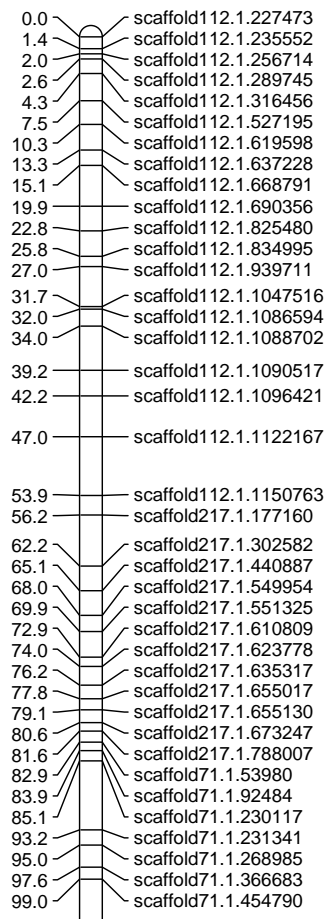

# 11 [2]

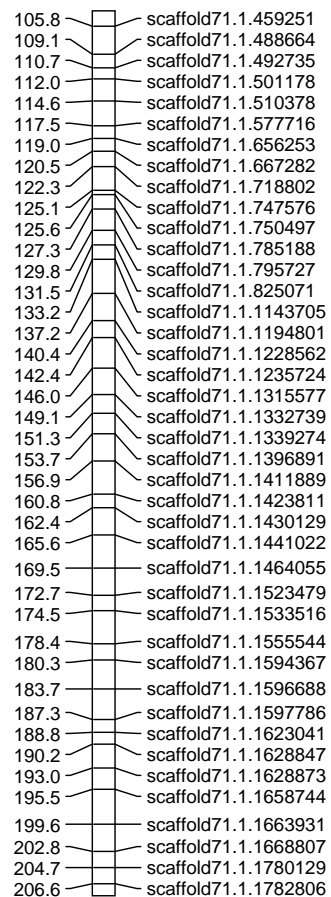

# 11 [3]

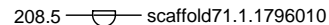

## 12 [1]

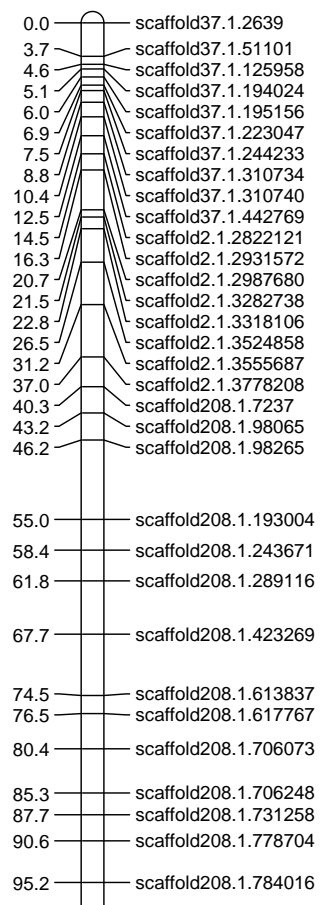

## 12 [2]

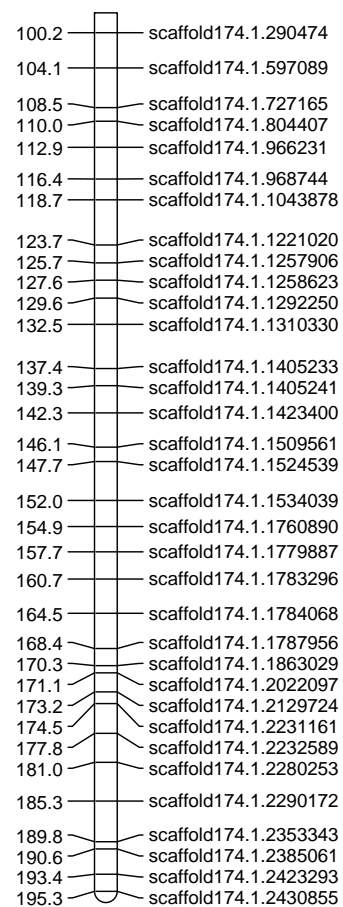

13 [1]

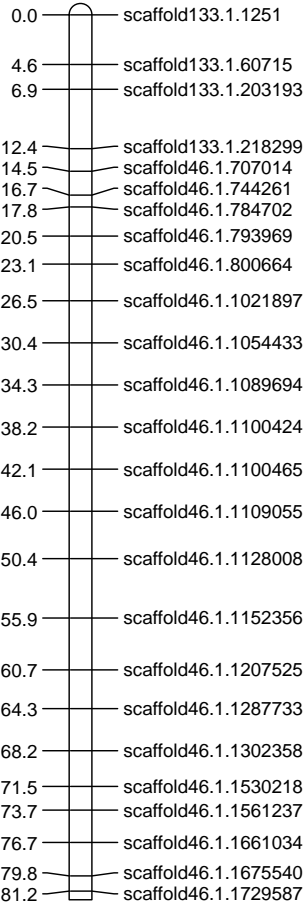

13 [2]

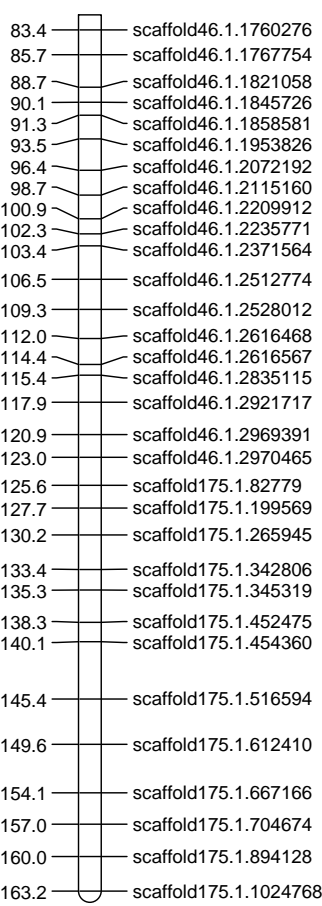

14 [1]

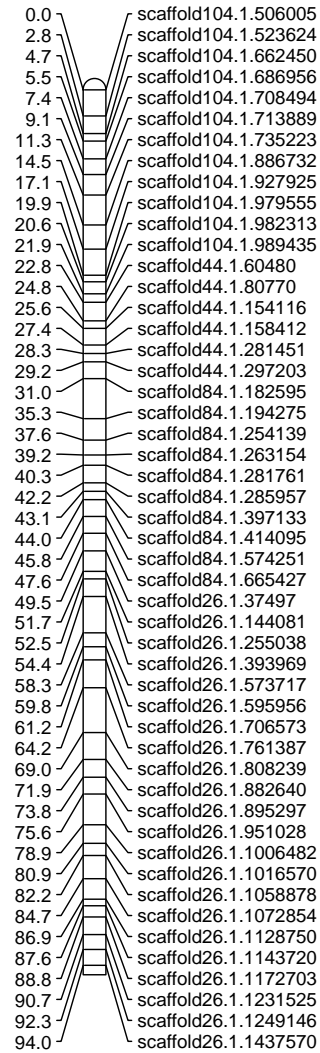

14 [2]

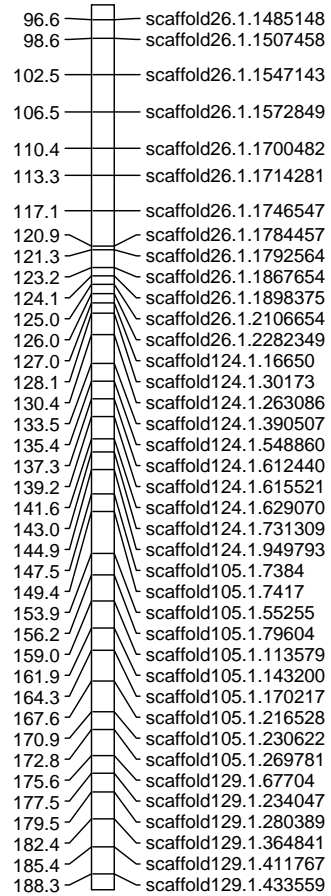

14 [3]

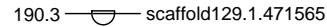

15 [1]

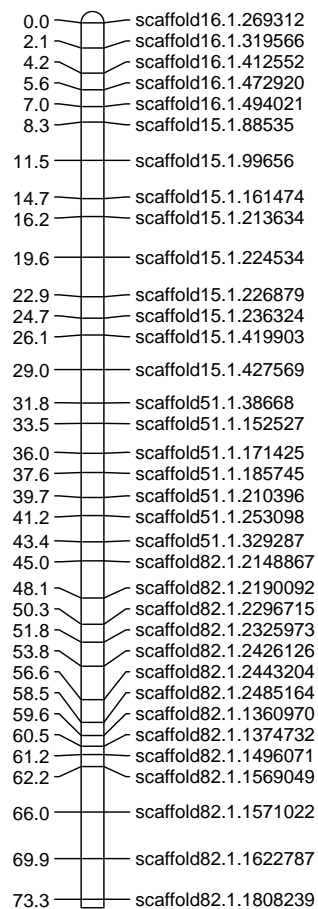

15 [2]

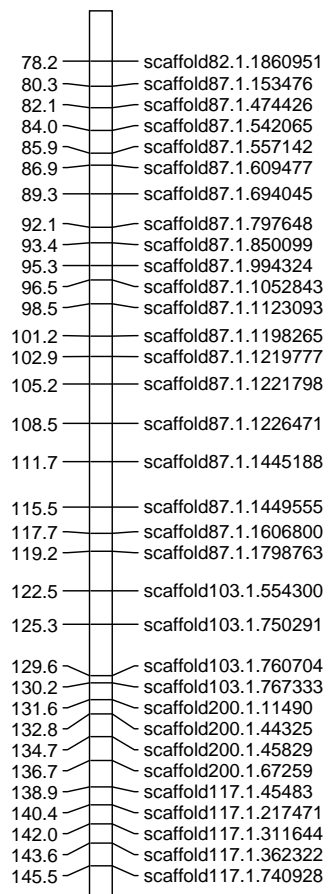

15 [3]

148.4 scaffold117.1.816577

16 [1]

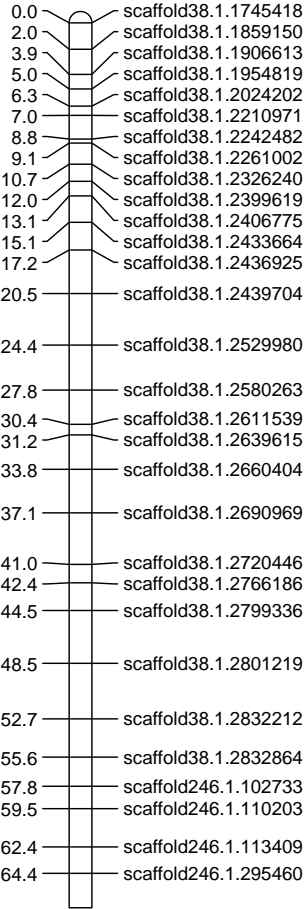

16 [2]

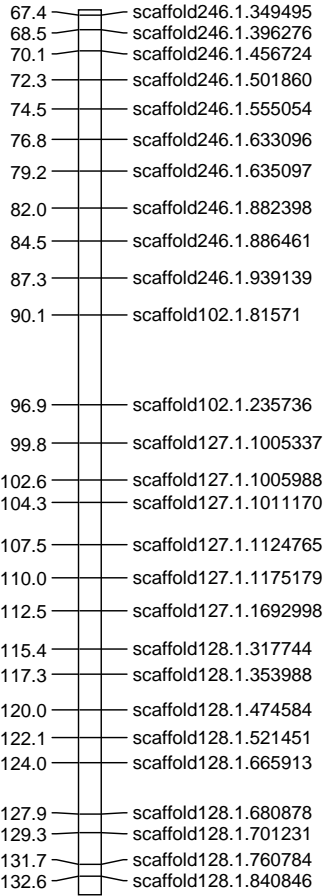

16 [3]

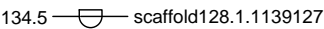

Supplement: Figure S1 — A SNP based linkage map for the Eastern honey bee ( A. c. cerana ). The map is composed of 1535 markers and 16 linkage groups (1–16), 13 major groups and 3 minute ones. The names of SNP markers are shown on the right, and the positions of the markers are shown in Kosambi centiMorgan (cM) on the left. (PDF) [file pone.0076459.s001.pdf]
