# Supplementary material for: A SNP Based High-Density Linkage Map of Apis cerana Reveals a High Recombination Rate Similar to Apis mellifera
Source: PLoS One. 2013 Oct 10;8(10):e76459. doi: 10.1371/journal.pone.0076459 (PMC3794977; doi:10.1371/journal.pone.0076459)
Supplement: Table S1 — The sequencing depth, sequencing coverage, percentage of mapping for each individual. (DOC) [file pone.0076459.s002.doc]

Table S1: The sequencing depth, sequencing coverage, percentage of mapping for each individual.

|  | **Depth** | **Coverage (%)** | **Mapping rate (%)** |  | **Depth** | **Coverage (%)** | **Mapping rate (%)** |
| --- | --- | --- | --- | --- | --- | --- | --- |
| **F0-Yunnan Queen** | 50 x | 91.6 | 84.67 | **F2-12-18** | 12 x | 90.8 | 81.64 |
| **F1-Queen 12** | 54 x | 91.6 | 80.62 | **F2-12-19** | 15 x | 91.2 | 82.74 |
| **F1-Jiangxi Drone 12** | 39 x | 91.5 | 83.51 | **F2-12-20** | 12 x | 91.1 | 83.37 |
| **F1-Yunnnan Drone 12** | 32 x | 91.5 | 83.46 | **F2-12-21** | 13 x | 91.1 | 81.19 |
| **F1-Queen 15** | 46 x | 91.5 | 79.42 | **F2-12-22** | 10 x | 90.8 | 81.93 |
| **F1-Jiangxi Drone 15** | 48 x | 91.5 | 83.71 | **F2-12-23** | 10 x | 90.4 | 80.72 |
| **F1-Yunnan Drone 15** | 38 x | 91.5 | 82.86 | **F2-12-24** | 11 x | 90.9 | 82.91 |
| **F2-12-01** | 10 x | 90.4 | 80.73 | **F2-12-25** | 14 x | 91.1 | 83.69 |
| **F2-12-02** | 11 x | 90.6 | 80.92 | **F2-12-26** | 14 x | 90.9 | 83.82 |
| **F2-12-03** | 7 x | 88.3 | 77.84 | **F2-12-27** | 15 x | 91.2 | 82.87 |
| **F2-12-04** | 13 x | 90.9 | 82.25 | **F2-12-28** | 14 x | 91.2 | 83.98 |
| **F2-12-05** | 11 x | 90.1 | 81.18 | **F2-12-29** | 19 x | 91.4 | 82.47 |
| **F2-12-06** | 12 x | 90.5 | 81.69 | **F2-12-30** | 20 x | 91.4 | 82.78 |
| **F2-12-07** | 11 x | 90.8 | 81.81 | **F2-12-31** | 15 x | 91.4 | 82.78 |
| **F2-12-08** | 11 x | 90.4 | 81.01 | **F2-12-32** | 17 x | 91.4 | 81.31 |
| **F2-12-09** | 11 x | 90.9 | 82.96 | **F2-12-33** | 18 x | 91.4 | 81.09 |
| **F2-12-10** | 10 x | 90.6 | 81.26 | **F2-12-34** | 16 x | 91.4 | 81.17 |
| **F2-12-11** | 13 x | 90.9 | 80.71 | **F2-12-35** | 18 x | 91.4 | 81.17 |
| **F2-12-12** | 10 x | 90.3 | 82.12 | **F2-12-36** | 17 x | 91.4 | 81.94 |
| **F2-12-13** | 10 x | 90.4 | 81.29 | **F2-12-37** | 13 x | 91.4 | 80.9 |
| **F2-12-14** | 12 x | 90.5 | 80.17 | **F2-12-38** | 15 x | 91.2 | 81.04 |
| **F2-12-15** | 14 x | 91.1 | 82.21 | **F2-12-39** | 12 x | 91.1 | 82.5 |
| **F2-12-16** | 9 x | 89.7 | 82.61 | **F2-12-40** | 22 x | 91.4 | 83.31 |
| **F2-12-17** | 13 x | 91.1 | 82.91 | **F2-12-41** | 20 x | 91.4 | 82.11 |
|  | **Depth** | **Coverage (%)** | **Mapping rate (%)** |  | **Depth** | **Coverage (%)** | **Mapping rate (%)** |
| **F2-12-42** | 25 x | 91.4 | 83.46 | **F2-15-02** | 16 x | 91.4 | 84.09 |
| **F2-12-43** | 18 x | 91.4 | 80.64 | **F2-15-03** | **8 x** | 91.2 | 84.08 |
| **F2-12-44** | 16 x | 91.2 | 80.66 | **F2-15-04** | 21 x | 91.5 | 84.81 |
| **F2-12-45** | 27 x | 91.4 | 85.33 | **F2-15-05** | 7 x | 90.9 | 83.27 |
| **F2-12-46** | 20 x | 91.4 | 84.94 | **F2-15-06** | 18 x | 91.5 | 81.24 |
| **F2-12-47** | 20 x | 91.4 | 83.51 | **F2-15-07** | 14 x | 91.4 | 84.19 |
| **F2-12-48** | 17 x | 91.4 | 80.79 | **F2-15-08** | 8 x | 91.1 | 82.76 |
| **F2-12-49** | 11 x | 91.4 | 77.07 | **F2-15-09** | 20 x | 91.4 | 83.08 |
| **F2-12-50** | 9 x | 91.3 | 82.72 | **F2-15-10** | 33 x | 91.5 | 75.28 |
| **F2-12-51** | 8 x | 91.1 | 64.04 | **F2-15-11** | 10 x | 91.4 | 74.17 |
| **F2-12-52** | 11 x | 91.4 | 81.83 | **F2-15-12** | 18 x | 91.5 | 83.52 |
| **F2-12-53** | 10 x | 91.3 | 83.04 | **F2-15-13** | 6 x | 90.6 | 51.21 |
| **F2-12-54** | 15 x | 91.4 | 83.21 | **F2-15-14** | 8 x | 91.2 | 83.22 |
| **F2-12-55** | 8 x | 89.7 | 82.53 | **F2-15-15** | 14 x | 91.4 | 84.55 |
| **F2-12-56** | 7 x | 85.1 | 70.56 | **F2-15-16** | 9 x | 91.3 | 81.67 |
| **F2-12-57** | 8 x | 87.1 | 79.89 | **F2-15-17** | 13 x | 91.4 | 81.67 |
| **F2-12-58** | 7 x | 88.7 | 79.88 | **F2-15-18** | 9 x | 91.2 | 79.18 |
| **F2-12-59** | 19 x | 91.3 | 70.71 | **F2-15-19** | 23 x | 91.5 | 82.55 |
| **F2-12-60** | 7 x | 85.6 | 74.23 | **F2-15-20** | 19 x | 91.4 | 83.45 |
| **F2-12-61** | 22 x | 91.5 | 82.81 | **F2-15-21** | 19 x | 91.4 | 82.93 |
| **F2-12-62** | 8 x | 91.2 | 83.58 | **F2-15-22** | 21 x | 91.4 | 83.44 |
| **F2-12-63** | 15 x | 91.4 | 82.85 | **F2-15-23** | 17 x | 91.4 | 83.35 |
| **F2-12-64** | 12 x | 91.4 | 84.25 | **F2-15-24** | 9 x | 91.1 | 85.25 |
| **F2-12-65** | 15 x | 91.4 | 82.81 | **F2-15-25** | 24 x | 91.4 | 83.42 |
| **F2-15-01** | 8 x | 91.2 | 83.76 | **F2-15-26** | 23 x | 91.4 | 83.53 |
|  | **Depth** | **Coverage (%)** | **Mapping rate (%)** |  | **Depth** | **Coverage (%)** | **Mapping rate (%)** |
| **F2-15-27** | 24 x | 91.4 | 84.58 | **F2-15-33** | 17 x | 91.3 | 84.39 |
| **F2-15-28** | 20 x | 91.4 | 83.85 | **F2-15-34** | 23 x | 91.4 | 84.76 |
| **F2-15-29** | 20 x | 91.4 | 81.68 | **F2-15-35** | 18 x | 91.2 | 84.47 |
| **F2-15-30** | 16 x | 91.2 | 81.17 | **F2-15-36** | 15 x | 91.2 | 82.65 |
| **F2-15-31** | 16 x | 91.1 | 82.91 | **F2-15-37** | 22 x | 91.4 | 82.91 |
| **F2-15-32** | 11 x | 90.5 | 83.39 | **F2-15-38** | 21 x | 91.4 | 83.71 |
