# Supplementary material for: A SNP Based High-Density Linkage Map of Apis cerana Reveals a High Recombination Rate Similar to Apis mellifera
Source: PLoS One. 2013 Oct 10;8(10):e76459. doi: 10.1371/journal.pone.0076459 (PMC3794977; doi:10.1371/journal.pone.0076459)
Supplement: Table S2 — The SNP markers name and 100 bp upstream and downstream sequence of every SNP marker. Each of the SNP markers starts with the greater than sign (>). Bases in [reference base/alterative base] are base mutation of each SNP markers. The sequence in front of [] is 100 bp upstream sequence of each SNP markers, and the sequence behind [] is 100 bp upstream sequence of each SNP markers. (DOC) [file pone.0076459.s003.doc]

>scaffold2.1.2822121

AAGAAGCAGAGTTTAAAGAACCGGTTATTTATCCAGATATGATGTGACAATCAATCGTGAACCGTGACCCGAAAAAGTGAGATACGTGTTAAAATGCCAG**[A/C]**TTCATCGCAATAACCTCGAACAAGCTGATTGACAGGACAAGTTTTAAAAATCACAAACAAAGACCAATTGCGGCCAATTACAAGTTGCAAGAGGAAAAAT

>scaffold2.1.3778208

ACCTAATAAATATATACCATTCATATTTGTTCATACAGCAGGTATCCCATTAAAAAATAATCCATAATTTCCCAAAAAGGGATAAAATAATATACAAACT**[A/G]**CCGACGAATTTAATACAACAAAAAATACACAATTCCAAATGTACAAACAATATACTAACCAGTAGCCAAATATTCAAATAACAACTCTCTTGACAAGACA

>scaffold2.1.2987680

AATTGTTTGACTGACATTCCATTTACTCTTATATGTATGTATACGAGTAAAAATATCTGTAAAATAATTGATTTTATTTCGTATTAAAATTTTATTAACG**[C/T]**ATAACATGCTTATATTTAATTTTTTTTTTATATTTTATGTTTTAGGAAAATTTTTATTCTAGAATTTTATTAAACTAGAATATAGAAAATTAAGAATTGA

>scaffold2.1.3282738

TAATTTATTCAAATTTATTTTAAATTAAAAAGATAACTTTAATTGCTAAGTGTCTTCTCACCTTCGTTGCATTGAGAATGTAGTTAGCCTCTTTTAACAG**[C/T]**AACAGAAATATCGCAGTTGCAATACCTGATTGCTCTACTATAACATTTACAGTAGATATGTGTACAATTCTTGCTATCTAAAATCAAAATTGAATTAAAT

>scaffold2.1.2931572

TTAATTTTTAAAATTTCGGGATGATTAAAAAATCTAATGCTTTGGAATATCTTGTGCGCGAAGCAGCGTACGATTCTTGTTGTTGCAGAGAAAAGTGATT**[G/A]**CAAAAGTTTCTCCAAGTTTAAAAACAGGCTAACGGTCTCTTCGAGATAATTAAATTTCACTTTTCATTTGGAAGTTTAAAAATTCGGAACTTTATAAAGA

>scaffold2.1.3318106

CTTCATAAACGAGGATTAATTTGTGTTCAAATATTTTTTAGAAATACAAAAAGTGTGTAAAGTTTTATAACTTTGCTTAAAAAACATTTAGATTTTATTA**[G/C]**AATGATAATAATAATCATAAAATGTGAAAAGATAAGATTTTTTAATTTAATAGGACTAAAATTTTTTTTATGTTAGTTTAAAAATAATTTAATCGTAATA

>scaffold2.1.3524858

CCCCACCCCCCTTGAAATCGAGAAATTGTGTTCTTATCCTGGAGATGTTGAAGAACGAGCTAAACAAAATTATTAGTGTGCGTTATTTGAATTCGAGGAA**[G/C]**TTGATATTGTTTGAAATGAGTTGAAAATAGTTTAAAATATCTTTATCGACAAATAAGTATATAAGTGTTGATAAAATAAGAATAATTAAGGAGATATGTG

>scaffold2.1.3555687

CTAATTCAATATATATATATATATATATATTAATTTATTATAATATATCAATCTCTCCCCCTCTACATACATCCAAAAAACTTTTATTCCTTTCCTTGCT**[G/A]**CAATCACCAAAAACAAATCACGGATTGCATTTGAAACATCCGTATAGAGATACAAAATAACTCGATGGACAAAAGAAAGAAAAAAAAATCAAAGCCATCG

>scaffold3.1.396292

GAACTAATCTAATCTAAGTTGAAAATTAATATTTTTGAATTTTACTCGTTAAATATTATCAAAAAACGAAAAACTGTCAACAATTTTAAAACAAAATTTC**[A/G]**TTATTAATAATAAATATGTTCAAATTACTAATTATCGTATTAATGATTCTTCATGATTTTGAATATTTTAAATAAAAACATAAATTATAAAAATATCACA

>scaffold3.1.703303

TGGAAATATTAATGAAACGAAAAATATAAAAAAAATATAAAATTTTAGAATAATCTTACAAGACTTTGCACTAATCGATTAATCAAAATAAATTAAATTT**[A/G]**CGGTAATTATGCACTTTTCTCATTCATTTCCTTGATATTGATATATATTAGGGACAACGATTTTGCATAATATTATTCAAATATTTTAATAATTTCCTAA

>scaffold3.1.317727

TATGAATTCTCGTCTTCCTTACAAATTGACAAATTGGTTTCGATTCGTATCTATGACGAAATTTATAGAAAGTTTTATACCGCCATTCGTATCTTCCCGT**[C/G]**ATCTAACCTTGATTCGATTGTCGAGAGAAGACAATCTTGCAAATTGATCACAATAATTGTAATTGTAAATATTTCCTGAATACGATTTTCAATTTTGATA

>scaffold3.1.444402

ATCAATTTGCAATAATTAATCGACACATTTCCTAGTATAAATATCTCCTTCATTATCATCAACTTTTAATATATTATTGTGTAATATAAAATTGAACGAA**[C/T]**AAGATATTAGAAAATTGAAGGCAAACAATGTTACGAACGAATCTTGCATAAGATAGTGCTCTGATAAACAAGGATCCGAAATCCACAGATTATCCACATA

>scaffold3.1.511095

GAAGAAAGAATGAGAGAGTCGTTCCCCTTTTCATAATGGTTAAGCAATTTCGCGATCTACTCTTCGTCCTCCATTATTATTTTCCGTATTACGCGGATTC**[G/A]**CCGGTTTTTTAATGAGTCGTTTCAATAGAAGAGACCTCTTAACCAATAAATGTTTCACGCTACAGGATTCTGTCTGATGGTGTCTCATTATTTGTCACAT

>scaffold5.1.140695

CAAAATTTTATGACAATATTTCAAGTATATTGTTATTAAATTTTATTAATTATAAAAGGATCATAAAATCGTTACAGAATTTTTTTTTCAAGAGAATTAC**[A/G]**TACCATTGTCAAATATTGTGTCATCCAAAATATTTTGTTCCTCGTTTGGAAAATAATAATTGTTCGAAGGAGATGCCATGTGAATTTATAAGCAGACGAG

>scaffold5.1.1528884

AATTTATTTCGGTAAATTTTATCTTTTGTTCATTTTATTAAAAATACGCGCTCGTTTCTCTGTTAATTACAACATTCGTTCATATTCGTATATTGTATCT**[A/G]**CAGGTACAGATTATTCCAAAATTAACCTGTTTCCTATATCTCAATACGAGGTTTAACAAAAGGCGTACTACATACACATTGGATTCACACGTGGAACTGT

>scaffold5.1.2978602

TCTGATGGAGAGACACAATTTTCTCCAAATTTTCATCCAAATTGCCTTTCGTAAATTATTTTTGTGTTCTCTTCACTTTCCTTTTTTTTTTATTTCTTCT**[A/G]**TTTATTTTGTTCGAATATCGTATGATACTTTAAACCAAGAAAAAAAAACAGGAAGAATAATACGATTAATACGATTGTTTGTGCGATTTTTGCCAACGTT

>scaffold5.1.3311700

TTCTTATATAAAAATTCTCCTTTGAATGGAATCTAATAACTTTCTCAAGAATTTTTGAGATTTATTCGAGATATGAATTCGGGAATAAAAATCGATGAGC**[A/G]**TAAAATGAAATAATTCTTATTTCTAGATTATCTATATCATAATTGATCTCCTTTTATCCTTTCCGTAGAATTGCTTGTTCAATTTTGTTCAGAAGTCCAA

>scaffold5.1.3430264

AAAATAATTTTCTCTCTCCTGAATTCCTGTTTACGTGCTCAAGAGGTACACAATTTATTCATTTGTTTATTTTAATAAAATTATATATACATATATATTT**[A/G]**CGAAAAATATATTCTCTATCACACAACAAAAGTTTTTCGTTTCGAAGGAATATATAGAGTGTAGAAATATTTTAATGGAAAAATAAACTTCTTAAATTCT

>scaffold5.1.4097771

TTTATATGCAATGATCATACAATTAATAATTTTAACAGTTCTTGGATTTTTATAATAATTATGATAATACAATTGACGTCTAATTATTCGATTTATTCTT**[A/G]**CTCGTCTTGAATCCTGTATTATTGACTATTATAGGGAGCTTCGAATTTTGAATATAATAATAATAATTGACAATTTATTGCTTATGAATTTTTGATAATT

>scaffold5.1.745748

TCATTCGACTAGTGTACCAATCGATAAGGATGATGAAAAACCTGTTATGTCAAAGGTATTTTAATGAAACTTATAAGTATATGTTGTAATCTGTTCTATC**[A/G]**TATCTTTTTTAAAATGACACTTGTCTCAATTATATTATTATCAATTATATTAAGGTTGGCTGTGCCATTAACAAAGAAGATAAAGAGGCTTTTGCTTTAA

>scaffold5.1.1798141

CGATACGAAATCTTTTAGTTAGCCGAATAACAAAATCCTCGTGTGATCTCGTATCTTTTTCTAGAGAATCGAGTAGATAGAGTGTTAAAAAACGTATATA**[C/G]**ATCTTAAGGAAGGATTAACGAGAAATGACAGCGATAATATTTCAATTTCGCGAATTAAAAGCGTAACAGTTCTGACCGATCGCAGCCAGGATTAACTTTC

>scaffold5.1.2101102

TATATTTTTTAATTTTATTTAAAAATACAGTGGAACACAAAGATTACTTACATATCCTTTCAAGAAATTGGACGATCGTCGATCCTCATTATCTTAAAGG**[C/T]**GAGCTACGCCAATGGCGAAGGGTTGAGATCGACCGTTGGTGGGTGTCTCCTCTCTTTATCTCCTTGCACAGATTTTAATATAAATTATAATCTAATGTCG

>scaffold5.1.2404072

TGTACACATCCATAACTTATTAATTTTAATGTTTAATCAAATATAATTTGCAATAAGATTTTCACATAATGATTCATAATAATTAAATAATAATAATCTG**[C/T]**AAACTAATCTTTTATATTTATACAAACCTATAATATAACATCGATCTTAAAAATCTTAAAACATCCCACAGCGATATTATTTATAATCGAAAATTGATTC

>scaffold5.1.2515549

TATACGGTTTAAATATTTGGACGTTTGGTCGGAAAGAGGGAAGGTATTATTCATTATTGGGCGAAAGAAAATTTAGAGAAAATTTTAGGAAGATAATGGA**[C/T]**AAATGGGGAATTTTTATAAAAATTACGAAGCGTGTAGTAACGTGACCAATCGATATTCGTGAAATATTAAAAAAAAAAATAAAAAAATGAAGAAGAGGAT

>scaffold5.1.2771616

AAGACTTCGTTGCCATAAATACCATAAATATCGCAGGTAATAATATATTCTAACGTTTAAAATTTTAATTCATCGAAGTTGGGCTAACTATATCCTTTCA**[C/T]**GTCTCACGGAATACAAGTTTCAGCATGAAAAATTTCATCTCGGTGATTAACGTTGATGAATGACGGAAAGGAGAAACTTGTTTTCTCGATCCTTCCCTCC

>scaffold5.1.2902553

AGGGGATCGAGATAACAAATGAGACACGCCAAGTTTTTGACACGAAATTGTTATATCGATTCTTTGATAGAAATACTTTCTTAATCTCCCATATAACCAT**[C/A]**GATAATATGGTTATAATACTCATCCAGTAAGCATATCTATCACATAATTTTACTACTCGATTAAATTATATTAAAAATATTAAAAATTTAAATTGTTTAT

>scaffold5.1.3274495

GATCTCTCGTCGCCGTGATCGTTTTAACCTGAGTATTCGGCGCTTTTGCACCGATAAATACATTCCCCGAGCTTTTTCTCTTGTTTATCGTTAAATCAAA**[C/T]**GATTAATTATTATTATTGCCACTTGAGCTTGTAATTGGAACACGATGGAAAGGACCCTACTTCAACAACAATGGTAGCGCGCAGAGTTCCCATGTTTTTC

>scaffold5.1.3444842

GAGGATACGAGATTGATACATCGTGATGTTATATAAGAAATCTCAGAAAATTAAATTGATTGAACAATTTTGAAAATAAAGTAAAAAATGTACAGGTTTG**[C/A]**TTTTCCTGATTGCTGATATTGCTAATTGTTCTTTATTCTTGTTTCATACGTTACAATGTTTGTAATTATTGTACAGGAGATAAAATCATTATACAATTAA

>scaffold5.1.3692137

AGGGGCTGTGACTTGATTTCCTGGCTGATTATTCAAAAGATTATCAAATGATGGAACCGAAGGTGATGGACCCACATTTGCAGCACTCTAAGTAACATAA**[C/T]**AAACAAAAAATAAACAACAAAATAAATAAATTTCAAATCATATTCAGAAACAACGAAAGAAAACAAAACTAATTAATAATTATCGATACATCTATCTTTA

>scaffold5.1.3858346

GGAATTGAAAATATAATCATTAAAAATTGAAAAATCAATTTTGAATTAATATAAATTTGAAATTAAAATAATTGTCGAGAAATCTGCATAAAAATGAACG**[C/T]**ATGTTTGATACGAGTCCATATTTTAAGATATTGTCAAGTAATATATGTAAATGATAATAAAAAAACATAGTAGAAAGATTTTAAATATTAATTATAAAAT

>scaffold5.1.4114617

TTTTCATAATGTTTTTAAAATCATTCATTCTAATAGTCTAATAGTTCATTCTAATGGAATAATATGATTAGATTTTTGGATATTGGCGCGATATATTTCA**[C/T]**GTATCATTTATAATTGAAATTCACGAAATGTACACTTGGAATTAAAAAAAACCTTTAAAATTTTAAAGGATGGAAAGGATTAAAGGATAAATTTACAGCA

>scaffold5.1.1713255

TTGCGATGAACGCACGATTCAATCGACCTACATTTTTTTCTTTTCTTGAAAATACCACGTACTACGTTTTTCCAAGAATCGCGTCTGGAGCTTGGGATAA**[G/C]**TCAGTGAGACGAGGTTTTGTGAAATTAGTTAAGTTAAATTTATATTTCCACGGTTCTTCCTTCGATAGTAAAAAACATGATCGATTCCTTTTCTATTTCT

>scaffold5.1.1852730

TATTAATTTTCCAACACATTCTTTTGATGAAGTATCAATGAGATTTCACTTATGAACTATTCTCCGAGATGAGAGGCAAAATGAAGAAAAACGAAAAGTT**[G/C]**AACTAATCCGACTATTTTGGCGTTATTAACAATTTCCAGTGTCCGCCATCTCGAGCGTTTTAATACCCCATCAAGTTAAAGGAGTCCGGTTAGTAGAGTG

>scaffold5.1.1951807

TTTTTGCTATTTCATTGTGTTATCCTCTCGTGTGCGTGTTTAGGAACAATGCCAAGAGATTAGAGATCTTGGAGTAAGGAGATCACAGCATGTGATTATC**[G/A]**TTACGTTCACGAATGGACTTGTTCCTCTCTTCTCGAGACAAAGGATATAAAAAAGGAGATGAGATAAAATAAGCATTAACGAGAAAGAAAGAATTATGAG

>scaffold5.1.208299

ATATTAAAGGAACTTTTCGAGCAACGAGATCGTTTCATTAGATAAATTACAATTTTCACGTAAAAATTTGTACCAAATTTATACGATAATTTCGCACAGC**[G/A]**TAGCGATCTAACTAAATTATTCGTACCATTAAAAAAAGAAAAACAATTTTAGTCAATATGAGTAAATATTTCAGGGCAGACGAGGTAGAATAAGCAAGTA

>scaffold5.1.2253927

ATATCATCGAGTACTTTCGAGAGAAATTTTCTTTACTCGCTTTATACAATGATACAATGATAAATATATAATTTTATGTATCAAAATAAAGTTAATATCT**[G/A]**TTCATTACAATGTTTCGAGATTTAGATTTGCTGTTTTGTAAGTTTCAAAATACTTACTCTTAATGTCGTTAAAGCGTATCTTTCGTCCATTGGAAAGTCC

>scaffold5.1.2307057

ATATTTTTGTAACTTATCGCCCACTTGTCATAAATAAGTTTGCCTTAATACACGCGAAATGGTTAATCAGATCGTTCCTTTCCTTCGTACATAATAAAGC**[G/A]**TGATTTCTTCGTTGATTTGCACGTGATTAAGTTGAGATCAAGACGATGACGTGACGAACAAGTAAAATCGATTTTTGATCGACATACGTGTGAAGAAACA

>scaffold5.1.2927918

TTGAATGGAGCGAAAGTTGTTGGATGTTTTATTTTGGAGTTGAATCGTGGCCTTTGTTTTTTTGGAAAAATGAAAGTACTATACTATTTGGAGGTTACTA**[G/T]**CTTTCTAGTTTCGAAAAATATCGTCCATCTCACAAATGACTATTTTTTACCGTTATTAACTATCTTCCTTTCATCAATAATGTTGAAAATAAATCGATAC

>scaffold5.1.3288397

TTTCTCGCCACCAAAACTTTCGTTTCGTTTGAATGGTAGATCGATGAACTGGCAAAGTCTTGATTTTTTATCCGTGATTCATGTAATGCTAATAATACTC**[G/A]**TAGGTTATTCACAAATATTTCAATTATTAGACATTTAAAGAAAAAACATATTTAAAAAAATCTTCTTATCCTTTTTGAAGATAATATATCAATTTTTAAT

>scaffold5.1.3372352

ATTATTTTTATTGTTTTGTATTCAAAGCATCTGTAGCTTCGTTATTTCCTTTGTAATACATGTATATAATGATAATATTTTATACGTACAGAATCTAAAT**[G/A]**CTTTCTCGCCTGTGATTTTATATCATCACGTTAAATCTTTTGAATTATTTGAAACTTTGATTATATGCTTATTAAAATTTAATACGAAATTAAAATTCAC

>scaffold5.1.3644655

GACTTTTACTATCGTACTGCTATCCGACTTTTTCGAATAAATTAATTCTATGAAGTTTTAATTTACTATCCACGCTTGGCAACATGATACCATTTATAAA**[G/C]**TAACAAATTCGAATTATTGTTTATTCAAAACTATAAACATTCAAAAAATCAACAATTTTAAAAGAATCTCTCAAAAAGTTTACATCCGCCAACACATCTA

>scaffold5.1.3674586

AATAAAATACTTGGTTAATCATTTGAGAGAGCTCAGCAAAAATAATAAGTTTTTAATTAAATATATTTCTCAATCGTTTAACATTATTTTTTTTTAAAAA**[G/C]**AATAAAGATTATTTATTTAATATTGCATTTAATAATCAATATCAGATGAAATTTTTGTCAGAAGAAATCTTCAATGTACTATAAATAAGAACTGTTGGCT

>scaffold5.1.3776465

TAATAATGTCAAATTGTAATTTCTTTAAAATTATTCAATTTTTATTCGAAGTTTTTTTTTAATATTATAGAATAAATGACACATTACAATATAAAGTTAT**[G/A]**CAAGATATCCTAAAATCTTTAAAATTAAAAAAAATTAAAACATTTAATATTGCAAAAAAAACTCTTATTATAATAAATAATAAATGCAAAAGAAAGAGTT

>scaffold5.1.3974995

ATTAAAATTAAAATAAAACTATAATTTAATATTATTAACAAAATTAAATTTTCAAATATCTAAAGTCGAATAAGTTATTTTAAAAGTTAATAATATTTTT**[G/C]**ATGTACGATAAATTTAAAAATATCAATGTTTGCAAAAGAAATATAAGAATATCGATGAAAACTATTCAAAATATGATTTTTATATTTTTTTATGACTTTA

>scaffold5.1.592886

AATTCGAAAATTATTATAAAGAGATAATCATAAAATTGTATAGAATAATAAAATTATCGTTATTGCTGTCAGATTTCAATTTCTTTCTCTCTGTTGTGCC**[G/A]**TGTAATTTAAAGTTTTGCTGACTTATAAGTATATACATATGGCTCGTTTCCTATCATTCGATTTTAATAATTAGACATAATAAATTGTAATTAAATTTTG

>scaffold5.1.735093

GATGTTACACAAGAAGGCAAACATTTATTCGCCATATTCCCGTCTTCATAAAAATGAATATTGATGCGACACGGTTATAACTTGTGGAAAGAAAAAGCAA**[G/C]**TTTTTGAGATGAAAGTAAGAGGAGTGGATGTCCACGCGTGGACTCTCTGTAGAAGAAAATTAGAAAAACTTTAAAGAAGGAAAAATTATCGGTTTGATAT

>scaffold5.1.929424

TATCTATCCTCGAAGGAAACCAATGTCAAGGAAACCGATATATAAACGATAAAGTTTATTCATAATACCTCAGCAAATCAATTCTAATCCTGAACCAGTT**[G/C]**AAGCAGAAAACCAAATCCTCCCTAAACATCGCCACTTCGACCTCCTAAACTCCTTTAACACCGCGATTCTACGTTCAATTCGAGGAGCCACAGGTTAAAT

>scaffold5.1.973780

TTTGCGAGGAACGTATGATTAAGGTTAAATACAAAAGGGCCGAAATTATTGTTCGGAAATCTCTCTTCTTATTTTCCCCCGCTATAAGATAAAAGGATTT**[G/A]**CAGGATCCTTCGATGAAAGAAATCGTAACAGGGGGACCATAAACATTCAAAGATCGAATTAACTCGAGGGCGATCTCTCTTTCCATTCCCTTTCCCTGCT

>scaffold5.1.123392

GTTAATGACGCCACCACGGATGTCGCTGCACGCTACAAGACACGCACTTTTGTATCTCATTTATTTCATGTTATATTTCTTTTTTCTATTTGTTTTAGAA**[T/C]**AAATTTCATATTAATTATACATTCTATCTAATTATATAATATATACTTCTTTATCTGATTACATAAGTATAACTCATATTATTTACTTTATATAATATAT

>scaffold5.1.2431549

TTCTATAAATTTTGAAGTGTGTAGAATAAATTTGAAAATTCGAAGGAATCTATCTTTGATTTAAAAAAATTACGTAAAGTTAAATTTTTATTAAAAAAAA**[T/C]**GACCAGAGTAGCGAATTTTCTAATAAAAAATATCTAGTTTCTTTAGATTTATGATTCAAAAGTAGATCGTTAGAGGTAAAATTTATTTTACCAGGAATGA

>scaffold5.1.2699320

TCGAGTTTATACGATTTTGCAATAATGAGGATTGTATAAATTCTTCTGTTCGCGAGTGTATATTGGTTTTTTTTTTTTTAATGAATGTGTTTTTATCGGA**[T/C]**GAGAATAATCAATTATCGAAAAGATAAATTTAGATAAAGTAATTGTATAAATATAAACTTACACGAAACTAAAAGAAATTTACTAATAGTTTGTATTATA

>scaffold5.1.2919967

CGATCGTACGAAGCTGTATCGAAATCTTTATTTTTAAATTCTTAAAATCGCGACTATCGAATAAATGTTTTTCATTCAACTTTAAAACGTATAACGGAAA**[T/C]**AGTTGAAGCACAGTTTCTTTATACAATAATTTAATGCGTGAACAGAGAATTTGATAAAGGATTCATCGTTGTTTAAAATTGATCATTTGTGGAATTAAAA

>scaffold5.1.3119727

CATCTTCCGTTTCTTTTCACGCCAATTGCCAATCAATTGTGGTATGCGATTTTTTTTATCGATGTTAAAGTGACGCGAAATCCAAAAATAGATCCTTAGA**[T/C]**AAATTCATAAAATCATCCTACAACTGCGCCATTGAATTTCTGTTCAAGCGAAGGAAAAAACGATTTTCACAACGTCGTATAATTCGACGAGAAAAGTCTC

>scaffold5.1.874786

GAGATTGTAATAAATATTGGATATTTTTGGACGAATCCTTTAAAGAAGTGGTAAAATATAATATTGAATTTAATCGAAGATTTTTATATTTTTTTAATAA**[T/G]**AATAGATATTTTCATTCGTCGAATGAAGAGCCTGATGGAAAAATAATACATAAATATTTTGAAAGAAAAGTGTACAAATATTTTTTAATAGTAACGCAAA

>scaffold5.1.941027

GTTATTATTGTCGATGTTCCGTTTAGATTACGAATCGTTGATTCGTTGCAATGCATATATATCCTTTTTCCCTCTCTTTCCCTTTATCTCTTTCGCTCTG**[T/C]**ATTCCTTTCAAGAACTTTTCTCAAGAGTGAAAATTTCGGAATAATTAGATCCAATGAATCATCGGCAGGACTTTCTCACGATGCTTGCAATAGAACTTAA

>scaffold7.1.1266779

TAAACAATACGTATTATATTTTAAATAATTCTAAATTTTACGAAAGATAATGATTTAAAAAAAAAATCGTAATCCAACAACTACTTTTCAAATTTAATTC**[A/G]**TGAATATTTAACATACATTATAGCAATTAATTGCGGCTAATATCAATTAAACAAATTTTATTATTCAATTTTATTTTTATTTTTAAACCTGCGCCACAAC

>scaffold7.1.1311135

TTTAATAATTTCGGTGGTGAAATATTTTGGTGGAATATTAATGTGCGGTTAACAATAGTAAGAATGTTTTCTTTTCATTTTGTCGATCTATCGTACGAGC**[A/G]**TTTGTAGAATTATAAAATTTCTCGAGAAAGATTCTATTTTTATTTGGAATACAAAAATTAAAACGTTAGACGAAATAACATTGTTATATTATATTATAGT

>scaffold7.1.628581

TGCATTGCAAAATTGCAACACGTGAAAGGGAGAATTGCTGGAATGTTACTTGTTTTTTTTTTGCACGGTCGAGTAATTATACTCATATGACACGTTTGAT**[A/G]**CATGCAAATAATATTTAACGATTAATAAATGAAATATTTTATGACGAGAATATAGGATTTTGTTCCGTGAATTTTTATGATTTCCTGAAATTTCATCATT

>scaffold7.1.1113109

ATGATTTGACAATTTACATTTGTCAGGACAAAGTTTGTATAATCTTGTTTCATTTTTTCAATTTATAAGTTATAATCATTTTTAAAATGTTTGTTTAAAA**[C/T]**GAAGTAATAACTTTCTATTTTCTTACATACGTAAAGCCATTGCGTTTAATTTATGTTATAAATGTATAATAAATTAAATAAGTTTTGCAATATTGGATTA

>scaffold7.1.1371645

ACCAACTCTTTATTTTCTTCAATCGAAAAATATCAATCTTAAAGGATCATAATGCTTGTACGCAGATAAATTTCTTTAGACTCACAAAAATTCCAAAATT**[C/A]**TTTCTTCGTGGGAAATCAAGTACAAATGGCCCTTCTGAAAATTTAATAATCGTTCCTCGAAGGACCAGAAACCAGAAATGGCAACGAGATAAGAGGTGAC

>scaffold7.1.1473462

ATATTAGTGATAAATTATTAATATTTTATTACATTGTAAAAATACGTATATATATAAAATTTTATGTTTTACATAATCATGATATACAATTGTAGTTATA**[C/G]**TATAAGATGTAATTAAATCTGATATTAGATTCTCTAAATGGAATTTTTATAAATTATAAAAATATTATAGTGAAAAAAATGTGATTGCATTCGTTTAATT

>scaffold7.1.563031

TTCTGTATAACAGGGTACAGTGATTTCTCGAGTGAATTACAGTGAAAGCAATATGTGTTATGAACGATGCGACTTTCAGCGTATAACGATCGTCGAATCG**[C/T]**GTTTAGATCGTATTTATTGCACGTCAACAGAGACAAATAAAATCTTGGTCCGCCCTCGTCCATAATTATTGTTATAAATTCCATTCACGGTTCTCGAGCG

>scaffold7.1.934028

TTATTATGTTAAGAGCATTATAGGTATGAGGATAAGAGATAGGGATAATTATTTAGAAAAAGATGATTAAGAAGTTATTAACAGAAATTTTTCTTGGGAA**[C/T]**AGATTAGGTTAGATTAGTTTTAATTAGGTTCTGGATAATTTGACTTCACGGTAGTCAGATTAATAAAACTAGTCCAAACTCAATGCAAACTATTAAAATT

>scaffold7.1.1227576

CTTACAACTATATTTCGTGCACTTCGATTTCAACTATTTTGCATGTAATGATTAATCTGTTCACAGTGAAAAAATTATTAATATATATAAAAGTCGAAAC**[G/A]**TTACAACGATACATCGACTTAAACTCTTGCTTGACTCGATTACACATCACAACGTGTTTCGATTAATCTACTTCGAATTGAGAATGCTATCTGATAGCAA

>scaffold7.1.1664032

CATAAATAAGTTTACAATGCAAAGAAAAAATAAAATGTTTATATAATAAAAAAAACGTATACAATTAAAACTCGCTATGTACATTTAACATTAAAATTTC**[G/A]**TCAAAAATAAAAGATGCTATTTTTTAGCAGAAATGAAAAAATGATCTCCCGATGTGAAATAAGAGAAAAATTAAATAAAAGGATATCGAATGAATGGAGA

>scaffold7.1.985327

GTTGCAATGATGGACGGTAGTTTGTTGTTATTTAATATTTTTTAAGAAATGATCGATGAAGGTTTAATAATAATATTCTATTGATTGACTATTAAAAATT**[G/A]**CTACCAAAGTATCATCAAAGCTATTTTAATTAATAAATATTTTTTTATTTTAGAATCAATAGGAAGTATTCTTTTCTTTCTTGAGAAGGAAAATATTACT

>scaffold7.1.1100250

ATCGTTTCATAAGAATTTTTGTACAATTCCTCATTTATATTATAGAGTAATAACGCAAAACTTAAGAGAAAACTTTATAATTAACAATGTAACAAATATC**[T/G]**AAACGTCAAAGTTTTTTCTTTTTTTAAAATTTGTTTAACAAAATTAATCAATTTCTTTCACTTATTATAAAATCCTAGAATTCTGATCTTTTTTCCTCTC

>scaffold7.1.1394832

GGTTAAGAGCGAGGTTTTCTGAAATTTGTATATTACATAATATTATAGAATAGTTGTTTTACTGCTTTGTGCACTCGACAAGAGGATTGTTTCTGATACA**[T/C]**GATTGTTTCATCTTGCCACATGAGTGAGCCATGGCTTGAGAGGAGTTTATTCTCTAGTCGGATTTCAAGAGCTTATGCTTTTGTTATGAAAACCTTGCGG

>scaffold7.1.1574723

TTATTATCCAATTTTCGTTTTTTTTTATAAATAATACCCGATCAATTTTCCTTGTTAGAAAAATTATGCCTGTTAATTGATAATCCTCAAATATTTAAAA**[T/C]**AATAAACTGCGTTGAAACAAAAGAAATGGCGATAATACAATTAAGTCAAAGACTTAAAGAGTTAAAAGTTCTCTCAAGTTCGATTCGAAAGATCAATTTT

>scaffold7.1.877345

ATTTTATGACAAATATAATTAAAATAAAATATTTTTCATTTAAAGTTATAATGATAAATATTTAAAAGAATTTTAAAAATACTCTTATACCTTTATTTAA**[T/C]**AAATTTAAAATTTCTTTTATAAAATATTTATTATATAGATATTGTTATACATTGTTATACATTGAGTTATCTATATTATTATTAATTAATTACTATTACA

>scaffold8.1.1569688

TTGTCTTCAAATCGAAAGAACAATCGTAAAATAGTTACAACGAGAACTATCTTATTAATCTAAATTTATGTTCGAGTAATCGGGGATCTCCATTCCTACG**[A/C]**TCTGCACATTCTCGTTACATATCCGACCTACGTTGTTCCGAAAGAGTTCATCTGGAAAGGAGTTGAACGGTTTCGAATGTATTTAAAAAGTTTCCGCTCG

>scaffold8.1.2737116

GAACCGGAACCTGAAAAGAAGACTCCAGAAGGTTTAAAGAATTAAATTAACGAATAAATTATTTCTTCTTGTTGCTTCTTTACGTTTTTCATTATCTCTT**[A/G]**CAGAGGTATTCCGCAAAGTTCTCATAAAAACTATCGTCAGCTGGGCCGAGGAATCCCAGATCGAGACTCCAAAGCTGGTTCGAGAAATGTTCAGTTTGCT

>scaffold8.1.3189795

GAAAAGAAGATGGAAAGCGAGAGGCGAAGGACGAGAACGAGAATGAGAACGAGAACGGGACAAGAAATGATGGAGATGAGCAAGGGAAGAAAAAACACTC**[A/G]**TGGCTAGTTTTTTTCACCCTTGGTCATGATAAAGAAGAAAAATTAAAAAAGAAAGAAAAAAAATAAAAACGAAGGGAGAAAGCAAAAAATAATGCACGGA

>scaffold8.1.3870755

CCTAATAACTACCAGTTATCAGCGTCGTTCGATTTCTCAGCCGCACGGCCATGTTCGCTGTGTAACACGCGACGTTGCGACACGAAGATACATGCATCAC**[A/G]**TTGAATTTTCGATGTGAACGAGGACAACAACGAGGAGAAATCCTTTAAAAATCTCCCCTTGTTGTAACCAGTTTATTCAAAACGTTAAGAAACGTTGAGA

>scaffold8.1.1242218

TGAAAAATCTATTTTATAATTTCGAAATAACTATTTATATATATTTTATAAATTAATACTAATTACAATATAATAAATTGAGAATAACGTGTAAAAAAAA**[C/T]**GTATTTTCCTGATAATTTATTCGATTCATGAAATGTTCTCCAAAGAATATCGTTTAAAGAACGATATTTACATTTTTTATCATTTACATGATACACGCGT

>scaffold8.1.1814750

TGTTTAATATTTAATTGTCTATTTTTAAAAGCCTTAGCTAGAACAATTATTAAATTGTGTTTACAAATTATAACTTGTATAATTGGTATGTAACTTCATT**[C/G]**AACTTGTTGATTTTAATAATCAGAATGTAAGAGCGAATGTGCGTGAAAGTACTGTTTTAGAAAGAGCTACGATATTTTTGGAACACGAAGATGATTAGTG

>scaffold8.1.3647287

CTGAACTGATCTCAAAACGTTAAAACATAACTTTAAAGTGGTAATAATTTTCCTTTGAAAGATGGCGCTATTAACTAAATTTAATTAAATAACCGCGAAA**[C/T]**GATTTTAAATTTAAAATAAGTAAAGGAATATTCTTCATCTATTTCAAATAAAAAATTGTATAATAAATTTGCTAATTTTTAAATTACAAATTTAAAATAC

>scaffold8.1.4444593

GCCTGTTACGGAGAGAGAAAGAGAGAGTATGTGTGTGTGTATGTATAGTAAATTGCGAGTCAAAAAGTGTTTATATTTTGAGGAAGATGAGATTGTAAAA**[C/T]**GAAAATTTGTATTTTACGATACACATATATAGATAATAATAATCGAGTAATCCTGTTAAAGTTATTTCGTAATTAAAAAGATACTATCTATAAAATATAT

>scaffold8.1.1466193

TCTAAAATCGAAAGATCATCTTCATAGGTGTAATAGGTGTAATAACACGGAGAAAGGAGGAATGATCCAATTTGGAGAAACTAGCAGTTGCGTAATGGGC**[G/A]**TTACGACGGTTCGTGACGTCGTGTGAGATAATATGAGCGAGAAAGCGACGTCGAAGCGTGCACCTTCATGGCCGTATTGAAAAATAAATTGCAATATATA

>scaffold8.1.1878296

CGCGGATGGCGTAGCGCGAGTAATCGATTCTGCCATACCTCGATGTGTTTCGTATATATATATATACGGAACATCTCGTACGGGATAATCGTTTCGAGAC**[G/T]**AGAAATTTTAAAGCTCGTAAAGCTCGATGGAAGAAAAGCTTGTTCGAGGAATTATTCTGTTCCAATTAATTAAAAAAAAACAAAAAAAGAAACGGCAAAT

>scaffold8.1.2033006

ACATCCGGTTAGATTCATTCGGAAGCGAATTATCGCCGAATCATTCCTCTTTAAAAATATTTATACCTTCCGATTTCGAAAAATTACGAGAGCTAACCGA**[G/C]**TGTTCTCTATCCTTCCATGCGGTTATTACAGGTGGAATCAATTATCGGCGATGAATGAGCCGCGAAAATAAATCCAGGCAACGCCATAGGTCGTTTCATT

>scaffold8.1.2775741

TGCGCGATTGTTCGTGCTTTTTAGAGGAGTGGAGGAGGGGAAAGGGGCGATTAACGTTTGCATTTCGTGGAATTCTTTGTTTATCGCGTTTAACGCGTGA**[G/C]**TCGAAAAAGCGTGGCGTTACTGCCGAATGTGAGATATATAACCGTTTAATTTTATCGCAACGCGAAGCGTGAACGGCCATGAATAAAGATAGAGCGGGTA

>scaffold8.1.3483629

GAATCGTTTTAAAACGTTCCAAGCGGAGAAGTTGGAGTTGCAGAAAATTATAATTATTATTATACCGTTTGATCGTAAATAGTGGAACATCGCGTGAAGC**[G/A]**TGGCACGCTTTTAAGGAAATAATTAATTGGTCGGAAGGTAACGATTTCGAAAACCCGTAACAAAAAATCGGTATAAAGGTATCGAGGGTTATACGCGTTT

>scaffold8.1.3925492

GTATTGACTATTGAACGTTAATTAATTTTTATTATCATAAAAAGATGGAACATTATAAGTGAAATCTATATGCTTATTATCATTTGAAAAAAACAAGCTT**[G/A]**TTTCGCATTTTATAAAACTAATGAGAATAATTATATATATATATTTTTTTTGCACATTCGGAATTTTAATGCAGCTATAAGTGTAATAAGATGACTGTGT

>scaffold8.1.4341448

CTTATATTATACATATAATACATATAATAACAATATGAAATTCTGTTTTTTGTAATTATATTTTAAATACTTTGTAATAATTTCATCGATTATATTTTTC**[G/A]**TACTTATAACGATGTTTGAAACATTGTTACTTAACCTTCAATTAATATTAACAATAAAAATGAGTTAGACTTAAAGACATTTTTTTTATATTTTACGTCG

>scaffold8.1.1313886

ATTGATAATAAAGTCAATAAAATCGATAATAAAAATGAGAATGTTTTCGTAAAATAAGAAAAAGAAAAAAAGAAAAAAATTGTCATTTCTGCAGGTATGG**[T/C]**ATTGTTATTGTATATCTACGTGATTGTTAAACGGAAACAACTTTTAAAAAAACATATTAACCAGTTCTTGTTTCGTTTTTTTTTCTTGGAATTTCCAACG

>scaffold8.1.1695355

ATCGATAATACTTCGTTTGTATATGCGACATTATAAACAAAAAATGGGAAAATATTTGTTGTATATTATATTCAATATTTTTAAGACAAAATACGATTAA**[T/C]**GATTACTTAATAATTGTCACGCAAATCATCATCATCATCATCATATAAATTTATTAACGTCAAATCGGACGAATGATAATTCGAGGGAATTCGAGCGCGA

>scaffold8.1.2714205

CGTCGAATAACGTAAAATTTTCAAAGTAACGATTTTAACTTGAAATTTCCATCTCTTAGCGGTCGCTAATAATTTCCTACGCGCTATCGCGTGTAATTGA**[T/C]**GAAGAAGGAGGTATAGTAACGGATACTTCTAACGAAAATTTTCACTATTTAAAGTAAGGGTTTCAACTTGAAATTTCCACCCCTCAACAGTCGCGCTCCA

>scaffold8.1.4201016

CTCAACTCAATAAAAAAGCGAAAATATTCATCTTTTGCGAGCAATGTATCTTGGACGAGAAAATTATTCGTAGAGATTAATTTTTCAATCGGTAACGAAG**[T/C]**GATCAAACTCGTAGCCTTAATGACTTGGAAACAAGTTTCCACAGTTTTGGTCGATATGCGTGTAGATATATTACAATACGCGGATAAAATGATAAAGCTT

>scaffold12.1.16493

ATTAAGATCATTCGACGCCTAGAACGTTGGAAGAATAGAGAGATGAATAGAAATAGAAAAATGATGCGTCAGATAAAATATATCGCGATGTGCTCTTGAA**[C/G]**ACCTCTTTTCTTTCTGGTGAATGAAGTATGTTAACGAATCAACAGGTTTCTTGCAATGTATACAGCCGACTAACACCGAGAAGTTTATTAAAGTTCTGTT

>scaffold12.1.63053

CAATATTTTATTATTTAACCAGGAATATCGTTTTTATAGAAAAGTGACAATTAAAAAAGATAAATTTTTCAGAAAATCGTTTAACAATTTTTTATTCAAT**[C/G]**ATTTTATAACGAAAATATTTTTCTTCTCTCACAATTTTTCTATTTATTTTTTTACGTACATTATATTCTACTCTTCCAATTTCTTTTCAAGTCCAATATT

>scaffold12.1.17545

ATATTATTAATTAAAAAAATTTATTTAATTTATTTAAAGTTAATTTATTTGATGTTCTACCAATTTCATTAAAGAAATGAAAAAATATTGAGAATTTAAT**[G/C]**TAATTCTAATTCGATGGATGTATCATAAACCATTATTCGTATATTAATGTCGAATATCTTTGAATTCAAATATTAATTAATTCCAGTAGCATCATTCGCA

>scaffold12.1.31804

AAGAATTTTTACGAAGACAGAAGAAACATGTTTATGCTGATTGATTTTCCTTTTAGACTTGAAGCTTTATATATTTTCGTGTATAAAGATTTACGACGAA**[T/C]**GAATGTTTAATATTGAAATAAACGTGAAGCGTTATAACTCAAAATATTTTGAATTTGGCTGTGCATAATTTTGAATTTCAATTTTTATACGATAAAAATA

>scaffold12.1.524169

TCTAAATTTCTAAAATTCATTTTAACTTCAACTTCAATCGATCAATTTTCTATCGATCGTCAAATAAAAATCGTTATATACAAAATACATAAATAAAGAG**[C/T]**AACGATTACTTTCCGGCTTAAAATTATTTATATTAAACGATGCAATTAATACAACTTGACAACGTCGATTCAAACGTGTTGACAGAAGAAGATCAAAGAA

>scaffold13.1.1062597

GTTATTTAATTAATTTCGTGTCCATTGTTTTTTTGAAAATCGCAAATAAATCTGATTTTTCCTTATATTTCTCTCTAATGAAAGTAATTGTCAAACTGAC**[A/G]**TCCAAATATTTTTCTTCTTCTTCCTTCTTTGTTTTAAATTTTTATTTATGTATTAATTTTTATTCTGTTGTGATTTGAAACGATAAAACATTTTCTCGTT

>scaffold13.1.1233571

TTGTATCTCTGCTTGATGTTATCTTTAAAGAAACAATTTTTAAACGTCTTCGAACCACGTGTCTGCATTCCATGCATTCTCTTTCTCCCTCCCCAACTCC**[A/G]**TTACACTTTTCATACGCAATTCCACTGTTCCGCAAAATTGAAAAAGTGTATAATAATTGCAAAAAATAAATAAATAAATAACTCGTCAAAAAATAATTTT

>scaffold13.1.204196

TTTTTACGACAAGCTTGAATTGCGCTGAGCATTTAGACATGATCATAACATGATAAATTCTCGGACAAAAATTTAAAATTTAATTTAATATCAAATCTTT**[A/G]**CTTCAATATTTTTAAAATAAATCTTTCTTAAAAATTATCATCTCGATCGTAAAAACGATATTAATATTTAGCATGCGTTTAAAACGTGTTTAAAAAAATT

>scaffold13.1.519811

GAATTGAAGAAACCACAACGCTTTGACTTTCAGTCTACGATTTCTGTTATTTTTATGGAATATTATTCCATATATATATATATATTTTTCCACACGTTCT**[A/G]**TTCGTTTCATCCATTCGCGTTTCAAACTTTTGAACTCGCCTCGATAAAAATCTGCCACCGACTTTCAAAAGGCAGTGGAGGAAAAAAAGTCAGTCGAAGC

>scaffold13.1.642973

TAAACATTCTGATTTTAATTTAATCGTAAATATTCCAGTAAAAAAACAAACAGATATGAAATTTGTATTATATCTGCATGTCAATTTAAAACAAATCTTC**[A/G]**CTTAACAATAAATATACGAATAAGAAAATTTGTTTTATAAGAAATTAGCTTAAAATTTTTATTATACAAATAAAAATATATCAAAGAATACGCGATGAAT

>scaffold13.1.837316

CGGTCGATCGAGCGAAATGAGGCAACTTTGAAATATTTTTTAATAAACCTCGCATTATATTTAATATAATCCAACCTGTAAATCCAACGAATTTATTATC**[A/G]**CCGAGATCAAAGGATATTAATCAATAAGGATAATTAATAACAGAATTAAACAGGATTTAATTTCAGAGATGAAATTAAAACAAATTTAATTTCGAAAACT

>scaffold13.1.1032217

ATATTCTCAACTAAATGGAAGAAATAGAATATTAGTATAGAATACATATTAAAAGAATATTGAAATTAGTATATAATTATTAAGAATGGCAAGATCGAAA**[C/T]**GATATAATAGTATCGAAAAGAACTTTCAGGTAAATTAAATTTTAAAAACGTCATTTTAATCTTGAAGTTATATATTTTAAAATATAAAGAAATTATTTTA

>scaffold13.1.1062539

AATTTGAGTAAAAACTTGACTGAGAAATATTGGTTAGAATATAGATCGGAAAACGATTGTTATTTAATTAATTTCGTGTCCATTGTTTTTTTGAAAATCG**[C/T]**AAATAAATCTGATTTTTCCTTATATTTCTCTCTAATGAAAGTAATTGTCAAACTGACATCCAAATATTTTTCTTCTTCTTCCTTCTTTGTTTTAAATTTT

>scaffold13.1.1201294

AACTTTTAACTTTTATTCAAATTAAACAGAAATATTTAATTCTTATAGTATTGTTTCCATATAGTATTGTCCAATTAATCTTTGAATTATCAATGGTGGA**[C/T]**AACAGATCGATTTACGCTCCTCGAAAGAGTCAATGCGCGAGGATGAGTCACTCTGTCAAACTCACACTTCCTCTGTTGATGGCTTTCGTTTCGACAAGAA

>scaffold13.1.1252545

TTGAAGTTAGGCCTTCTCTGTCCTAATAAATCCGCGTTCGTTATCTTTTCCCTTCATCTTTGATATTTTCGAGTATAAGAAAGAATGAAATTTTCAGTCG**[C/T]**ACAATTCCGTCCATACAAAACGCATCTTTATATCGCACCGTGAATATATTTTAGTTTTCCTGAATTATTGTTGATAATTTCGTCGCAGCCTGTCGACGAA

>scaffold13.1.1440862

CTTCTAATTCTAATGGTGGTATCTTTCGTGTGTATATTCTCATATGACCCAAAAATGTTTTATATACTATACTAGAATCACGTTTTTTTGTACCATTTAA**[C/T]**GCTAAATGAAGAGCATTTAAAAACCATGAAAGAAAATCAATTGGATCGCCTAAAAATTTTATTTTCAATAATTAATAATGAAAAACAATAATATTTTTGT

>scaffold13.1.1646501

TCATTATTCGGCCGACAAGTCGGTGCATGTACCGTTCCCTTCCCTTGCATTCACACATTGCGGATCGTTTGTGCAAATCAGAAATGAAATTAAATTGCAA**[C/T]**AATATGGAAGCTTTTAGACAATTTCTGTGGTATATATTCCATTATTTATTATATTTTTTGTATTCGTGTGAAGTTAAAAATTACGTTCATAGGAAATTAT

>scaffold13.1.1722513

ATATTCTAATGCGTGTTGAAGGAATAGATAATGATAGTTCAGAGCTTCAGAAGTAATAATTGTGGTGCAAAACTGTTTTCATCGCGAAGTAACACGTTAA**[C/G]**TCTAAACAGCACACTTAACAGAAACATAGTTTCCTTAATCTAGGTAACAAAGAATTACTTTCATTGTTTCTAAAGTAAAAGTATCTTACGACGTATAATT

>scaffold13.1.1723559

TGGGATGTACGTAATTAATTAATTTCTTTTTTCTTTTTTTTTTTTGTTTCGTGAATGGTTTAATTGTGCGTGGTTGGAATATCGAATTGGATGGATTACA**[C/T]**GAACAAATTATTGCATAGGTTGTTATTAATTCTTGATACGAAGATTAAAGTTAAGTTCATCGATCGACTTTTGTATATTTTATATTACGTTCCAGGTTAA

>scaffold13.1.1754655

AATAAATTATTCGATCGATATATTCTTTAGAAAATAATTATTAGTTTCTTTACGAAACGATAGAACAATAAGGTAAATTAATCACATTTTGTATGACGAG**[C/T]**ATTCTGATAATTATTTTTTCGAATTATCATAATCGATTATCTTATTTATTTTTAGCGATGTATCATCGAATACGATAATAATGGAGATATGTTAAGGAGA

>scaffold13.1.549697

ATTTCTACATACCTGGATCGAGTCCACTTTTAAATTCCATTCGAATGCGCAAACTTGAATCGACTTTCTTCGCATCTGTCTGTGCTTAGCTTTTCAACAG**[C/T]**AGATACCATCTTTTTCCATTTCCGTTTTAATACCACTCTTTCAATCTAATTGGACTTACTCGCAATCTTAAAAATAGTCTCAAACGAATAATTGGAACGC

>scaffold13.1.679126

TATTGGTTAAGATTTATTCTAGGGGGAGGGGAGAAATAAGATAATTCGTATTTCTTTTTTTAAATTTTCACTCTGTATGATTATTTATTGACGAGGTTAG**[C/T]**ATTTTGTGTTGGAAAAATTATTTAAAGAAATTTTAAGAATATATTAATTGAAGAATATATGACTTTTTGGTCAGTTTATCTGATTGTATCTGATCTGTTG

>scaffold13.1.689597

TTGTCAAAATTTCAATAATTTTTGCATTATCAAATTCAACTGCATCATTACGATTCTCCTATCATTAAGCAATTTTTTATATTAAACGATTAATCCCTAA**[C/T]**GGTTCATAACATATTACGTTACAACATAGAAAAGTCGATAAATAACCAAATGTATAACCACAAATCTTCATAAATTTTCTCCAGGAAAACCGAAACCTTA

>scaffold13.1.1061692

CTTTTATCAAGATATTATTAAGAATGAAGGAGAAGGGAAATAATTATGGAAAGATAATAATAAAAAGTTTTTCTCGAAAGAGATAAAGAGAAAGAAAATT**[G/A]**CACGATAGTTTTTACAGTTTTTCTTCATTAATTTTTATGAATCTCTCGTTTTTTAAAATCTCTCTCTCTCTTCTTTTCTTTCTTAAGTAAAATACACGAT

>scaffold13.1.1162891

TATTATATATTTATTATTTAATATATCTGTAAATATTGAGATGATTAAGAATATGATATTTTATAAGATGCGGAGAGAGATTTTTTTCAATTATCATCAC**[G/T]**AAACTTCACGCAAAATGAAATAATTTTGAAAAATGGAGATTTAAAAAATTAAAATTAAAATAGTTCGTGTCTATTTTCATTTTTCTTTTCAATAATTTAA

>scaffold13.1.1187306

TTATATATATTTGGAAAATATATATCTGTCACAAAGTCTTGAAATATTCGAAGGATCATTATCTGAATTTTATCAATTTCCAAAAACTCGGTTATCAATC**[G/A]**TTGCAAATTAATCGAATGGTCTGTATAAGCATCCTCGTTTTCCTCGTTTTACACGTTGATCTCCTCTGGTTTACAAGTCCTACATGACTACTCGAAATCG

>scaffold13.1.121147

ATAAGAAACGCATCGAATTATATGGATAATTTTCTTTTGTATTTTTTAGCATTTCGATTTTTTTTTCAACATTTCACACGTTGTGAGGAAAAAGTACAAT**[G/A]**CAAATATGGAGGAATATACATAGATACCGGCGATAAAATGGTTTGCTAGGATGAAATGCGATGAAAAACGCGTTAGCTTTTTAATGGGTAAGCAAAAGTG

>scaffold13.1.1217740

ATCCGCCAGTTCTCCGCGATGTACTTCCTTGAGAATTTTAGAGAAGCCCCTGGGGAAAGGTCCGTGAGTTTGGAAATTTGGGAAATGTCGTCTTCGTGTT**[G/A]**CAATTTTATCCTAAAATCGTCGATGATTTTTTTCCCCTGTTTTTAATACTTTTTCTTTTTTTTCAGTTGCTGTGAAATCTTGAACATTTCGAAATTTTTG

>scaffold13.1.1380116

GATCTTGAACGGATCGATGGAAAGAGGATGGTTTTTCGCTTTTTTGTTTGTAAATGAAAAGTTTCTTTACCGAAAGAGGTTAAATTTTTGTTAATGATGC**[G/T]**AAGAAATACGAAGAATTAGCTGATAAAAAAAAATGGTATAATTATGAATATATTGTATGGAAAATTTTTAAATCGAATGATGATATATTTATTTTTCGCT

>scaffold13.1.1418497

ATATTGTATCATGAAAAAATAAAATAAATGAAGAATAATTTCGGCGGAGATATATATAATTCTAAAAATCATTTCGTACTCTTAAAGTAGCGATTGCAAT**[G/A]**CGTGATTCCAAACACTGTTAACGGATGAACATGTCATGAATCCACAAATATGATAATTATCATGTTCGATTAAACCTTATATATTTACCTAATATTACAC

>scaffold13.1.1424941

TTTTATATATCGAAAAAAATGGAGTGGAAAATTGAATTGAAGAAATTTTACAAACTTTTGCAACTTGCAAATTTTACAAAATTGCAAAAAGTTCGATTCT**[G/A]**CTTGATCAAGATTGGATCATATTAAAAAATATCAAACCTATATTCCTAAATTTATTATAAATGCTCGAAAGAAATGGGAAAAAATGGAATAATTTACAAC

>scaffold13.1.1555976

TTAGAAAACTAGAAATTTCTAAATCCTATCTCCATATCGTTTGTATACCTTCTTCAAGAAATATTTCTATCTAATCTAGTTTATATTAGAAATTCAGATT**[G/A]**CTTGTTAGAATTAGAATTTCTGCAGTCTTCGTTATTAAAGAAAGCCCGAGGGGGTTGGAATTGAAACCTCGTTAACACTAATGAGTTGAATAATCTCTCA

>scaffold13.1.1622614

TTATTATCATCTAATTGTTTCGTGTGTTATTTGAAAAATTATTCGCTCGAAATAGAAAAATTATTTAAAAAATTTCCAGAGATATTTATTAAAGCGACGA**[G/C]**TAAAATCTCTGGAGCTATCTCAGGACAATAATTCAACTTTCCATGTTAAAATACACAAAATAATGAATCAAATTGTCAGCTTAGATCATAGCGTCAGCCG

>scaffold13.1.1756547

TTTCTGGTAAACTGGCCCTAGTTCAACGAGTTTGATTATTTCCGCCTCGACGAGAAAAGTGAAACTCTCGATTATTCCTCTCTCTGATTCTGTGATTCGC**[G/A]**TTTTATATTGTGATACTCATGATTTTCGCTTTCGTCGATACATTCAGGTTAAGTTTTATTTCCTGTATAGAGAAATATGTTTGTGAAATTGTTTCTGTTA

>scaffold13.1.1830435

CACACGATTTTGAAATATTCCTGATAAAAGATAAAATATTTGGAAAGTTTACGTTACGTACGCTCCAATTTATATATATTGACAGATATAATTATTATCC**[G/A]**CCGGTTAATTAATTATCGAAACGAGGTTGTATCGATCGTATACTTAAGATAACGGGGGAAAGAAACCAGATTTTCTCGTTTCGTTTCCTCCGAATGAATA

>scaffold13.1.2047073

ATTTCTCCACAAATCGATCGATCAAAAAAATAATAAAAATTGCAACAGACGCGATAAAATTTGTATTTTCATTGAAAATTCGCAACTTTTCAGAGACGTC**[G/A]**TTTTAATTAATTAATCAATCGGTTAATTAATAATCAAGTTGAAGCATATTTTTCAAGCCTTCTCGTCTTTTCCTTCGAATATCCAACGGTCGTCTTCTCT

>scaffold13.1.291592

AACGATTGTTGATCCGAGTAGGCGAGTGAATTTTTATACGACCAAGAGTTTTTTAAATCGTCGAGGGAGTATGAAAGAAAATTGCACGATTGTCAAAAAA**[G/T]**AAAGGTAATTAATTATTATATATACGTGTAATAAAAATTCCATGATTCTTTCTTTTTACACTTTGTGTCGAAGGTGAACATGGATAATATCGATGAAATT

>scaffold13.1.756506

TTAGATTCCCTGTTTCGCGGTGATAAATGTCCACGTTTTGAAATAAACGAATTATTGTCAACGTTACGAAGCGAACAAATTTCCAATGATGAATAACCAC**[G/A]**TTTCGTGGTTTTTATTTTTTAAAAGAAATTATTTTTAAATTTGCATTTCTATGTATAATTATATTTTTATGAAAAATATTCCGATTATTATATTTCCAAT

>scaffold13.1.89690

TAGTTGTTTGAGCTGTGATCTCGAATTATTGAATTGCGGAGTTCAATAGGCAGGTTGGTTAAACGAGAAATTAAAATTGTGCGAAAGAAAAGGCGTGCTC**[G/A]**CAAAATCTTTTAAATAAAATCTGACTATTTTATTGGAAAATATCATTCGTAAAAATTTGTTAGATATATTTTATTCAGAATTAGATTTCTTTATAGATTT

>scaffold13.1.908299

TGAATTATATCGATTAAAAGATATCTTAATCTCTTTGCAAGGTCGTGAAAGAAATATTGAATTGGAAAATATTTTAGTACGATAGAAAGAAAAAGAAAAA**[G/C]**TTTTATCGATCACGATGAAAATTTTAATTTTTGTTTTTAATTTAAAAAAAATTTGCATCGATTTCTCATACAGAATTTCGATAATCGTTCTACGAGATTT

>scaffold13.1.1139581

GTATTTTCGATTAATCGTAAAACGAACACTTTATAAGATGAAGCAATGTAGATTTTATAATATTCCAACTAAACAAGTAGCAGGATAAATACAGAAAAGA**[T/C]**GAAGAAGTTCCAGAAGCCCTTATCCCTCTTATCGTCCTCTTAACAGAGGAGAAACCGAAGCTTCTTATCGAGGGAAAAAAAATTCTCGACGACCTCGAAG

>scaffold13.1.1158710

TAATATCACTTAACTTTTCTACGTCATAGGTGTCGCGACGACACCTTTAAAGAGTTCTTCGTCACTCGTGGGTAAGAAATTTCGATTATGATATCAACCA**[T/C]**GTAATTGCACAACTCGTGAAAACTTGGCCGAAAGTTCAACGTACAGAATATCTTCTTGAACATATTTCGTCGGTCAATGCGCAGCCATTTTTAGGTAGAT

>scaffold13.1.2003950

TATGTGATTACGGCTCTTTCCACGTGATTTATCGGTTCCTCTTACGGTGTTCCAGTTTAAAATCTCGTTTGCTGTATTTCAGATCATTCCGAGATCCGGA**[T/C]**GCGATAGATCGTTTTAATCCTCCTTATTAAAATTTCTTTCTAAAATTCGATTGAAATATATTTTTGCCAATTTGTTATTAATTTATCGTAAAATCAACTT

>scaffold13.1.220572

AATTAAAATAAGGTTTAGGAAAAGGGTTTCTTTATTCAATCATTTACATTTTATTTTTGTTTTAATAAATTTGCAAAAGTGAGAAAAAAATGAAAGATCG**[T/C]**ATAATAACAATCATACAGTTGTTCAAGTTTCAATAATTTTAGTCCAATAATTGTCTATCCTGTGTTTCAACACTCAAACAACATTCAATTGCAGCGTGTC

>scaffold13.1.741867

CATATATAATTCTCTTATAATTCAGATTAAAACATATTTATCACGTATTTATCGCATAATTTGTAAATATTCGTCGAGTGAAATATTATTTTCCACGGGG**[T/A]**CTCATTATTAATTATAACTCGATCCAACAATTAGAGCAATCACTGTCTGTTTACCGGTTAGACGAACCGTGAAATCAATAATCGGAGGGAAATGAGATAA

>scaffold14.1.308895

ACATGTATTCACATATCGCGTTGCAAAAATTTGAGAACGAGCGTAGAAGTGGTGTATAAGAAACAATATAGCGAGTCTTTGATAAATAATTCGAGAAATT**[A/G]**TTTTAGAAATCGTCATAAATTCTAGCGTTATTAATAATGTAACTCCATCTGTGTTTTCTGAATTTCTCATTACCCTAATTATCCTATTTTCTCGGTCCAC

>scaffold14.1.1010332

TCCTTGAACGATTGAAATTGCAATTTTGGGTGTCACGATAATATAGATAAGCGATTTGAATTGATAAATGAGATTAGTAATCTTTTACAATAAAAAATTA**[C/G]**TCAATTACTTCTCTTCATTCGTAGCATATCGAGATTTTAGATGGAATTTTGAAACGCGTTTTCGTCGAGTTTTAGAGGTTCAGAGAGTTATGAATACTCA

>scaffold14.1.1178665

TTTAATTGTTCTTGGAAAGAGATATATTAATTATCCGGGATATAACGAGTTTTTTTCCACTCCGTTTTTTCAATTTTTAAACGAAATCCAAGTTTTATAG**[C/T]**GAAGTAAAAAAAATAATGAATTATATACCATACCATCTCCCACAGAAAAATTTGAATGTTGAATATAAAATAAGCTCGTTTTTCAAGAAGCACGATGTTA

>scaffold14.1.1297822

CTTGGAAATTCCATGACGAAAAATGACTCGACGTTTTTCTTAAATTATTGCGCTTCTTCGTCGATCTTTTGAATTTTTAAAATATTATCGTGATATTGGG**[C/T]**AAATTCGAGAGAGCCCAACAGAGGATCGGATAAATATTTATCAAATATTTTAATTCTGTTTTATTTAAAAATTATAGATTATTATTTAAAATTGATTATT

>scaffold14.1.1497596

AGCTTCCGGCACGGACGATTTAACGTCCAATCAATCGAGAATGAGCAATCTATACGAAAAAAGACGCTGATAAAGAGCAGTTATTAAAATTCTTTTACTG**[C/T]**AACAACGAAACATACAAGTTCGGATACAAGTTCATATTATAAGCCCCTCGGCATCTTGCGAATTATCCATGATGTAAAGGTCATGGCCTTAGTTATCACC

>scaffold14.1.1563268

TTAAATCGAAATAACGTGTTTTAATGATTAAAACACGTGTATAATTAAATTCATACGTTACAATTAATCGAATAATGTAAATTTATTTGTCTCATTTTCT**[C/G]**ATATACAAAATCGTATAATACGTTTTATAAACCTGCTTATTATTCTTTCGAAAAAAAAAATAAAAAAATAGAGGATAAATCCACGACCGATAATAATGAT

>scaffold14.1.520517

AACTTGAGAATGGGATTATAATTAAAATTGTATAGTTTTGCTTTTGAAAAAGGTATTACACGACTTATTAAGAATTACGGAATTACTCATTTATTCTTTA**[C/T]**GATTCCTGAATGAAATGGATTTTTGTACTTGTAAGTTCTGAGCCTTTTTTAAAATCAAATTTTCTCTCTTTTCTCTCCCGATTAACCGAGAAAGAAGACG

>scaffold14.1.984728

CGGAAGAACCATATCCGAAAGATCCATGTGTTCCATCACCTTGTGGTCCAAACGCGATGTGCAAAGCCATAGGAGAAATTCCAGTCTGCACTTGCATGAA**[C/T]**AATTATATCGGAGTTCCACCGAATTGCCGACCAGAATGCTCTATCAATTCGGATTGCATAGCAGATAAGGCATGCATTCAGGAGAAATGTAGAGATCCTT

>scaffold14.1.1064580

GCAAGGCGATCGCCGCTGATACCACCGATCTTTTCACTTTGCTTTTGTCTCTCTTCTCCGGTTCTTTCCCTCTAAACTCAACACGTATCTACCCTGAGAA**[G/C]**TTGTCTTATCCAAATAATATCCCGATAATATCTTTCGTTCGTACGTATTTGAACGGCAAATGTTATCAAATTTATAGAAAAATCGTTTTAACTTTTAATC

>scaffold14.1.1170249

TCTCGATATTAAACAAAACTATTAATTAAAGATCTGAATATATTATGAACAAAGAAAAATATATATCATTTTTTGAAAATTGTATCAGTAGAAACATTAT**[G/A]**CGTATTATTTCATTTAATTCGATGCCTACATGTTACATTCCTTTAATAAAAGGTTAATCGACCCTGAGACTATTAAGAAAGAGGAGATATATATATCTCT

>scaffold14.1.1215829

ATATTTTTTCAGAATTAATGTCTTGGAAATATCGTTTTCTTTTATCAAAGCCAATATATTTTGTTGACTCGAAAAATGGTATGGAATGCATCTAAAAAAC**[G/A]**TAATTCAAACGTGATAATTTTGGACAATTGAGAATTAATAACCTGGAAACATCGCTTCCTTTAATCAAACTATTTATTAAAAAGTTCGAATCTATATCGA

>scaffold14.1.1228226

TTATTACGGACAGCGTATTAAGCGTCCACGCGCAATTCGATTCTTATTATCACGTTCTATTATTACGAAATACTTTTCTCGCTCGTCCAAATTTATCCTC**[G/A]**TCGAATTTTCAAATGATCGAATTTTATCCTGGCGAACACGAATTGGTACAAATCGAATATCTCTTATTTGATTTAAGCCAATCATCGGCGAGATTTTAAT

>scaffold14.1.1276889

CAAGGTATGGTTACCTGACGGAGAAAAGTCACATTAGTCTTATTCTTTCATTCTTTAAATATCAAAGTTATTTAATTTCATGTATAATTTGAAAATGCGT**[G/A]**CAAATTTTCTATTTAAAATATAGCTATTATATTTATTTGCATTTTATAACAACCTCTTGCGTTAAATGTCGACATACTTATCGATTATTGATAGTTGAGA

>scaffold14.1.1308730

TATATATTTATGCGTAGTAAATAGAATTTTTTTTCAATGATTTTCAAAATCATCATTTCTTCTCTTTCGAAAGTCCTTTTTATTCGTCCTAACTGCGAAA**[G/T]**CTTCTTGGATGATATTATATCTAGTCTCTTAATGCAATCTCTCGTTTAAGAAAGAGTTCTCTGGATCCACCCATAGAGCAAGCCACGAAAAAACGTTGGA

>scaffold14.1.491703

TTTCCTAATTTTAATAGGGTTTAAGGTAAATGATCTAATCGTCTTTTCTTTACACGATCATATCTCGGGAACCGAAAGTCGTATCGAGACAAATAAAAAT**[G/A]**CGTTTTAAAGGGGAAGATTACGCGCTTCTGATGATCTTTCGTTTTTCGAACGTTCGGAGTTATCTTTAGAGTTATAACGATTCAAAGTTTTCCTAATTTC

>scaffold14.1.702968

CCATTCTTAATCGTGCATCGTATTGGATTCGATTTTTCTCGATATTTCCCCATACTTTTCGTGATTTAATTGTTAATATTTTTAAATCTTTTCGAATTTT**[G/C]**AGTTGAATTTGTATTTAATTTTTCGTATCATATCGTAATACAGCGATATCAAACTATTCGTTTATAGAAATTATTGGTTCCCTACTTTTAGATTGAAAAT

>scaffold14.1.727664

TATACTATGGGGCGTGTTTAATGATTTTCCATCGTATCGTTGTCAGATCCCCTCCCTTTTTCGATGGGAAAAGAATGGGAAGTTTCGTGAAAAGAGTTTC**[G/A]**CTAATATCTCGACACTGGGATTGCTGTGGGTCGAGGAACGAAAGAAGAATAAGAGTGTGTATATATATAAAGTTTTGTGTACCAAGAGTCGAATAGTATT

>scaffold14.1.771378

CGGAAGTTGAAAAACAAGTTTATGACGTTTATTAGACGTGTTTATAAGCAAATTGATCAATTTCTAAAAAGGTAATACAAAAATAATTGGACTGCCATTT**[G/A]**TCCAAGGTTGTAATTACCTGGTAATTAATTGGTTCATTTTATTGGCACTTGAGTTAATTTAGATCATTTTCATAAAGGATGTTGAATTTGTTTATTTCTT

>scaffold14.1.1366423

GCAAAAAATTATGATCTTATGAAACTTACTTTTTGGACTCTTTCGGGATTTGATTATGAATGTTGAGAAAGAATTAAGAATTATCAGAGACTTAAAATAA**[T/G]**AAGATAAACAAGTGGTAATAAATTAAAGTAATAAGAGTCGTGTTATAAGAACGATCGATCCAACTTTAATATATTCGAAACGATATAATACATCGTTCCA

>scaffold14.1.1416040

AGTAACTTTGCCTCGTTTACTCAAATTTACACGCAATCGCACACGGGACGAGACGAGTGAAAAATTGAAAAGATGATTCAGAAATGTTTAAATATCGTTA**[T/C]**GTCTTGTTTTGTTCGGATCGAGGAGGTAAAACAGACTTGCATTATCCGCGATAGATTCGAATCCAACGATTCTTCCGAAATTTTTATTCCATTTAAATGG

>scaffold14.1.1440178

CTCCACGCGATTTCCGGCGTAACTTCCGATGACATTCCTCCACAGATTCATTCAACTTGTGATCGTGAATTAATTAATTATACTCACGCTTGCTATTCTG**[T/C]**ACGAAGAAGCGAAGCATCGATTCGTAATACGATAGAACTAGATTTATATGAATCTGGTAAATTCATGTAATAATAGTCTATCCATTATTTATATTATTTC

>scaffold14.1.467371

TTTTTATCGATGTTATTGCGAGCTCGATCGAACGATAAAAATATATTACTCGTATAGTGTAAACAAATAATAATCGTATAAAAAGATTAATAGAGTAATA**[T/C]**GAGTATTTTATTTTTAAAAAGTTCTTTTCCAAATAGTAAATAAAATATTTGCTACAATTAACAAATTTTACAAATTTACAAATTATTCCTCGAATAACTC

>scaffold14.1.911190

CGGTAAGCCTATTATATAATTTCATCGAAAATATTCCTAATTAAAAGCAATCGCTCTCCATGGAATTTTAATTGATTTCTTACTTTATTACCTAATTTAA**[T/C]**AGTTTTTATGAAAACGTTTATTAACGCGTTACCTTGTGTTTCTTTTTCGCAACCGACTAACCAGAATAAAAAAGAGAATAAATAAAAAAGAAAAATGCAT

>scaffold15.1.99656

ATATTTATTTGTAATTTCAACGAAATACATCGGCGTGAAATATTTCTGCGCAATCGCGAAAATCAATCAACGCGCAACCATCCGTATAATTTCCATATTC**[A/G]**TCCGAAGTTTGAACGTACAAACTGGTGGTCGATTTTAAAAAGATCATCGTATCCTCTAGAGGAAAAGGCTGGGACACGTCGACTAATCCGTCGAAATTCG

>scaffold15.1.213634

AAGGAAAAAAACTGAATTTAGAAAAAATAAATTATTGGAATAAGTAATAATAAAATAATAATGAATTCAAAAATAAATGGATTTAAAAATAAAACTTAGG**[C/T]**AATGCAAAAGATATATTTGATTAAGTTGATATGAATTATATATGAATTATATCAACTTAGTCAAAAAATCTAATAGATAGATAGATCAGAATTATAAAAA

>scaffold15.1.226879

TTATTTTTATTGATTAATTTATTATATCAATAATATTCATTAATAATTTTTTATAGATCAGAAATATAATATTCACGAATATAATGATAGAATCGTAAAG**[C/T]**AAATAAATAAAATAACGAAAAAAATGTAATGTATATTCATATGATAATAAGTAACAAATAATTATCATAAATAATCACACGTAACTAGCGGAATTTTAAA

>scaffold15.1.427569

AATCTTGTCTCGATTTCTTCTCGCTTCAGAACTGTCAACATTGAGAGAAAATAACAACTTTATTATTCGAAAGTTGTACAGTTTCTTCACTTCAATCAGG**[C/T]**ATCTACTTATCATCACAATTGTCAAAATATTCTTATTATTTAACAGAGCGATCAGAATTCAACAAAAGGAGCGGCAATGTAGCATAGAAACTGTATTCAG

>scaffold15.1.88535

AATATTTATTTATTCCGCCACATCGGACACCAATAACGCGTTTAACAATCGATCCAGTTCGATCGAAATCACCGAAGTTTCCGTTTGCCTTTCGAGGATA**[C/T]**GCCTCCATCACGATATTCCGGTCGCTCGGCTAAATGGATTAAACGGCCTCGTTTTAATATTTGCGATGGTGGGACGATTGTTATGAAAATAGAGTGGATC

>scaffold15.1.224534

TCTTTACATCTCGTTTAGGGTCGTAACTATATAAAATCCAAAGTGTATGAGAACGGAACGAATAATCAAACTTACCACATGCTTAAACAATCGTATCATC**[G/A]**TGATGTACGTTGTAAAAACATATCGATCGTTCCTTTCAATTCAATAAAAGGATGAAATTCCATTAATTAAATACCACCTTTTCTATCACATTTCGCTTTA

>scaffold15.1.236324

TAATAACATGATTGAACTACTTAAAAAATACAACCATGAAAATATGAATTTCAATTCTATATATTATATTATTATATTATATAAAATAAACCGATGATGC**[G/A]**TAATACCAAGAAAAATTTTCATATCATTTGTAACAATTAATGAATTTCTTTCACTGTATTTTCTTCATAATATTGGCAAAAAAAAAGTAAAGATCAATAT

>scaffold15.1.161474

TTCTAACAGTTGCCTAACCGTTTGCAGAGTGCACCTTTTTAATACCGCTGATTATCGTTTTAACAACGACACACGCATTATTACGTTATCTTATCCTCGA**[T/C]**GCCTTCCCCCCCGATTCTTTTTGTCTATTTCTCTCTTTTTCCCCTTTCGTTTCTTCCCCTGTCATCTTTCCATTCCATAGAAGGTTGTTTGGTTGTTTGT

>scaffold15.1.419903

TTAAAAGGGATTGAATAGATATATCCGTTGAATTATATTTGTCACGATTATTATTTTAATTCGATAAGAGGAATTAGCCAGCTAAGAATAAAAAAGAAAA**[T/G]**AAATCTTTCATGATGCACATAAGAGATCGATTAATACGGAAAGGATAAAAGTTGAACAATCGAGTTTCGTGCAAAGTCAACTCATGAATCGAAGGGAGAT

>scaffold16.1.269312

CGTTCATCCATTACCACGAAATTCCATTTCGAAACGGAATTCGTATGAATCACGCGAAGCTGCGACTGCGTGTTTACGTGTCCTTTCCACTGGCACAATT**[C/A]**TTGCTCGCTTCCTGCTTCGCAGAGAAACGGCCAAAAATGGCTGACGCTCTTCTAGGTAGTAGTGTTACCATTCCATCTTTTCGCGAGTAATTCCTGTATT

>scaffold16.1.319566

TGAATTAGTCTGTTTCTTTTATTTTTTTTATTTGAATTTTTATTTTTTATTGTTACGATCATATGATATATTATCGAGATATTAATTTTTAAAGAATTTT**[C/G]**TATCGCATTTGTTTTAAGAATTAGTGTTGCGATGACGAATGATCGCGTTGTTTGAAGTAGTGTTGCGTTCATGAATAAACGCGTTTTGTATAGTAGTGTT

>scaffold16.1.412552

GATTCTTTTAAGTTCGTTTGTGATGTAAGGGGACACGGAAGTTCTTTTGCACTGTTAACGAGAATTTAACGATTATTAATCGTGCGAGACGTGATTATGA**[C/T]**GATTGTAGCTGGATGCTAGAGTGGGGTATTTAAATTGCACGTGTATTAAAAACAAGAAAAGTGTGAGAAATCGTGTCTCGCAAACTTTTACGCAAAGATA

>scaffold16.1.472920

AGTTTTCTTACTCGAACTCGGGCGGAATTATGCCCGGGTTGTTCTCGCAGTCGAGGGGGTTTCAATCGAGCGTGACGTGCTTTCTGGTTAAGAAAAGTTT**[G/A]**CGTAACGATATGGCGATTCTTGGCGATGTGGTCAACGGTTAATATAATCGTGGATTTTTTATATGGACTCGATGATTTTTTATTGCAGTTGGATTTTTTT

>scaffold16.1.494021

AAAAAATATCGTTCGATTCATAAAATATGTTTCAAGAGTTTTCTGTGTAGATAATTATTCCTCTCGCTAATTTATTCGAATAATATTCGATATCGAGAAA**[G/T]**AAGGAGAGAAGAAGGAAAAAGAAGGGAAGAAAAAAAAAAGGTAATACACTCACAGCTACACAAAGAGTTAAAAGGAAATTTCGCGTTCGAATCAGATACG

>scaffold17.1.30158

ATAATTCGGTTTCTTAATTATGAATTATGGTCGTTTGAATATAGAATAAAAAATATAATTCTTCGGACCAAGCAATTTTCTTTTTGATTTTAATTTAATC**[A/G]**TTATCCAGTTAGATTGTAACATCGTTTCATAAAAGAGATACAAAAAAAATGTATCTCAAATATAAAAGTAAATTTCTCTATGAAAAATGAAATCGGAAAA

>scaffold17.1.742262

AATAAATGAATCTTTATAACTTGACTTCTCATTGATTGATTAAATTACAATTATTCAAACAATACAATAATTTTACACGTTAATGAATTCGTACTCATGC**[A/G]**TTTGTTCGAATGCAATAAAAGATACAATAAAACAATCGTCAATGCACGAAGTAAAATGTTCCGGTTTTATCGAAGCTATCTGTTTAATGGTCGATTAATA

>scaffold17.1.15731

ATCGGTTGCCAAAAGTTTTCAAGTTCACAATCTTAAAATCGAATCGGGCAGATGTGTAACGATATTGAAATCATTCAAAGCCGTGCAAGTTTCGTGGTTT**[C/G]**AACTGTTCGATATCGTAACAATCTTCTCGAAAGATTTTCGCATTAAGCCACAACTTTCGCAGTGAACGTTATCTGGGATACTGGGAGCAATAGATCGTTA

>scaffold17.1.754025

ATCCATCATCATGTATCATTCTCATCTTCACAAATACAATGCAAATTATACATAATAATTAATCAACTCTTTATGATTACAATATAATTACCGCAATACT**[G/A]**TTTACAAAACAAAATCAAACAATCACATAAATTAACCATTATAAATCTTTTTTTCATAGGTTGATGCAAGCGAGAGATCGTGAGCCATTAAGATGCATTC

>scaffold17.1.780724

TTTGTTTATTAGAGCTCCTCGAGGTAAATGAAACTTTTTAATATTTTTAATTTTCAAAATATAAATATTTAGTTTTTCATTTTTAAGAGTTATAGCAATC**[G/A]**TTGACAAATTACAATTAAAATTATATTTAAATAGTTAAATTACATTTTATAATAATTAAATTATATTTTAATAGCATCTAAATTTTAATCTTTCGTCTTA

>scaffold17.1.530838

TAATAAATTACGAATATAATTTTTATTTAAATTATCTCATTTTTACAAATGAAATATGAAAGTAAATGTATCGCTATTAATTCAAATCTTAATACCTTCA**[T/C]**GAATTATAAGCATTTCTAAATTTTTGTTGTGGTACTTAGTTGATTTTATTCATTTGCAATAAAGAAATACTAATATTAAAACAAAAAATAAGTTTATCCA

>scaffold19.1.426133

TCGCCAATTCGAGGTTCGATCCGGTTCCTGCACCCGTCGGAATGGCGTTGTACTCACGGAATTATAGTATATCTTTATGATATATTCTCGTCGAGCGAAC**[A/G]**TTTTGAAAAAAGGAAACAGATGTATCTCCCTAAGTTTCTTCTTTTCACTATCATCACATGAAAATTGTCATATTTTGCCCATAATAGATACAAAATTTTT

>scaffold19.1.1148690

TGCGAAAAGAACATTCAATGTTTACGCAGTTCAATCTCTCGATTTCATAAATACCACGATTAAAAATTTTTTTTCTTTTAAACAGCAATGATCTTCGATT**[C/A]**TTAGCTTTATCGATACATGAATATACGTGACGTTTGCAGTTGTTGATTATCAGGTAAGAAATTGCAATACTTTTTATTGTTCGTAAATGCAAGAATCGAT

>scaffold19.1.118824

TTTAATTTCGTTTGTATACTGCCTCTCGGTTACCACTTTCCCTCCCTTGATAGAGAAGAGACTTTCCTTTTCGCTTCTCACATTTGTTGTTAACGGGGAA**[C/T]**GGGTTGAATTTATTTTCCATTGTTCGAACGGATGGAAAGGGGGCTCTATGGCATTTCGTCTAAAATTTATGCGCTTTAATGCTGAGCCTTCGAACGAAAA

>scaffold19.1.1208932

TGACATCTCGATTTTTTCAATATTACTGTGAAAAATGTTTCGTATGAACGTTAAATGATATTAAGCGTGTTATAATATTATTACTTCATTCGTGAAAAGA**[C/T]**GAAGATCAACTTTGTTTCTTTTTTTAAATAAAATGCAGCTTAATATTCTGTAAAAATTATTAATTCAGCAGATATTTCAACTCTGTAAAATTTATTGCTC

>scaffold19.1.136862

CATGTTATATCGAATTTTTCATATATACAGAGAAAACCTGTACAAAACCATACACCATATTTATTTTCGTAGCCAATTCAAAAAATCCCACGAACAATTG**[C/T]**ATTTTACAATTATTCTTCGTCATATTAAACATTGTTTATCAGTAATGTTTTATTATAGATGACGTACGTTGTAAAGAATCATCGAATTTATTGTATTTTC

>scaffold19.1.1485537

TGTTAAAATTAATTTCTCAATGAAAAAATTTTTGATTGAAAAGATAAAATAATTTATAACATTTTCAAATTGAAGAAGTGGAAAAAAATTATTTAGAAAA**[C/G]**TACATTATGTTATTTCAAAATTAAATCTTCTAATAGTCGTAAATGTATTTTTGTAATTCTAAAATTTTTTTTACTCTGATTTTTCGATTTTATCATAGTT

>scaffold19.1.1502616

ACAAAGTTATTATTTATATACACATTTGTGTAATAATTTCGATTTCCACATTTTCGAATTGTTTAAATCAATTTTCCGACTTTGTCGGAAAAACAGATCG**[C/T]**AATAATTATTTAAAAAATAAAATAACCATGGCGAATTTTCATCAACACGATAGCTCTCATTTAAACGAGAATATAATGAAAAATTAAATTTGCATAAAAA

>scaffold19.1.207467

TCTTTTTTTTTTTTCTTTTTCTTTATTGAAAAGTTATCTCGTCCAGAGATTGGAGGATGATTGAACAAGTTTCTCTACGCTCAAGCAATTGAGATTTGTT**[C/G]**AGTTTTTAAAATTTTGTAATGTATTTGAGAAGTGAGTCACGGCTTAACGTAAAAATCTTATCTCTGAATTATTGCCGGATGAAAGAAATAAAGGAAAGAT

>scaffold19.1.416167

ATTGGGAAAATTTGGCTGTCAAAAGATGTTGGATTTTATTTTATTCAGGAGATATGAGAAAATTTAAAAAGAAAACACCATGAGCGTTAAATGGATCTGG**[C/T]**GATAAAATTAGTTTATTAGATATGAAATTAGAATACTGGGAGTTTTAAATGGATCTCGTGATAAGATAAGGATTTTATTCTTATGAAAGTGTAACACTAA

>scaffold19.1.427795

ATTAATAAATTTAGATATAATACAAGAAATTTTAGAAATTTAAATTGTTCAATATAAAAAGAGGAAAAAAGATTTTCTCTTTTAATATCAATATTTTTAA**[C/T]**GTTTTAAAAAGAAGAATAAATTTCTGAATCGATTCTTATCACATATACAATAAGTGCATGTTATTTCTCGAGAGCAAAATAAATATTTAAAGGGAAATGC

>scaffold19.1.483850

GTCAATGTTGTTTTACCATCAAGATTTAGGCACGATATTTTTACAAGTTCTCTCAATCTTATCTTTCAATTCGTTAACCTTACCATTGAACCAACAACGA**[C/G]**TTTATACTTATAAAATCACAAAAATTATTATTTGTATAATCATTGATCACTATCTTAAGAAAAATAAGTTTCATATAACCTTACATTTCATCGCCTGACA

>scaffold19.1.657028

TGTGCTGTTTCGTGTTTTTTTTATTAAAAAAAAAAAAAGAAAGAAAACGTTCAGATATCGTGATTAGGAAAGAGAGAGAAAGAGAGAGTGTGCGAGGAAA**[C/G]**TTTGTCTTTCCTGTTCGTCGCTTTTCACTGTACTTTCTGTTATGATTTTGGTGCGGTTTGGTGGAAGGAGCGAAGGAGCCACGCGAGGGAGATACAACCT

>scaffold19.1.1015661

TTTTTTTATTTTCATTTCACAGTCACGGAGTAGAAAGTCCATTTGATGAACATTTAAGAAGAAGAGTTCTCCTCGCTATATTGCATATTATGAAATTCCA**[G/C]**TAACAAGTTGTTATTTTATTTGTATCTATTTATATAATCTTTTCCCAATAGAAGCTTATATATTTTTTTGTGTGATAATTTTTAACTTTTTATAGAATAA

>scaffold19.1.1050016

ACGATTAAAAATTGTTTTTATAATCCAAGGAAAATAAGTTCTTTATTTTCTATCATTAAATTATAAAGTTTCCAAAAATATATTCAAAAAAACGTGTCTC**[G/A]**CTATTTTTATTTTAATAAATCTCAAAAAATTCCTAAAGAATTTATTTCGTTTTTTAATTTTTCTTAATAAATAACGCGCGTACACCTTCTACTCCATCAA

>scaffold19.1.1095656

ATACATTACTTAAAAATAATTGAAAATCAGAACGTTTTGTACGATGATCGAAATCCACCATTTATTCCATTTCGAAAATGAGATGTTGTCAATCATTCTT**[G/C]**AAACTATCTTAAAGTACTCTGGTATATTAATTATAATAGCATCCATTACATTATATTTCACAAAATTATTGTCAATCATTCCTATGTTTCTAATTATAAG

>scaffold19.1.1106031

TGTTATAGAATGCGGGAACGTAGTTTCAATTAAGATAAACGAATGATAGAATCAATAGTAAAGCGAAATCATTAATGAACAGGGATATTTTTCTATGATT**[G/A]**TTTTCTTTTTAATATTTTCAGATCATCAAAAAAGATCATAAAACTAAACTTTTCCACGAAAATAAGTAATTTGGATAATCTTTATGGAGGATTAATTTCT

>scaffold19.1.1137819

TGTCCTTGTTAATATTTTTCTCGAAAGGAGTTAACGTATGAAGAAAATTATATTATTGCGTCTCTTTTTTCCTGGAGATTCTTACGCAACAACCTTCAAA**[G/C]**TTATATATAGAGAATAAGACAAATTATATTATAGGTAATTATATATATATATATACCTATTACATCTAATTTTATATAAATCCGTAAACTTGTTGAACGT

>scaffold19.1.214994

ATCTCAGCTCTAACATTCATTCATTGGCGTGGAACTATTGCACGTTCGCTCACCACCCACGTACACCATCGAACCCAATGTCATCACATTAATTTTCATC**[G/A]**TGGCACGATTACAAGCTTTGCAGAAATAATATTATTCGAATCCGTGTCGGAAGTGTCGATTATTTTGTTTTCGTTTCTCGCGTAATTTGGTTTGTGTCAT

>scaffold19.1.22582

AAGGTGGACGAACTTCGACTTGTCTTCTGAACAAGTATTTCTATTTCTGATTTTCAAACATTTTTCTTGGCATTTTTTGTTAGAAAAATAGCGGGGGGAA**[G/C]**TTTTTGAAATTGAAAGTATGAAAATAGAAATGAAAACATTACTATTTTATTTGATAAAAATCTCGATGTATCTATTATAAATCTTTTTCATAAGGATTGG

>scaffold19.1.31836

TCCAAATATCAAATATCAAACGAATTACTAAAATACTTGGATCAATATTACTATCCATGAAATACGCAATCCTTCCAAACGACATCGTCAATTTACAATC**[G/A]**TGATCAATTTACAAATAAGCAATAAAAATAAAGAACATCCAATTCTTATTATAAAATTATATCGAACCAATCGCTAGATTAAACGTTCACATTCCTAATC

>scaffold19.1.39810

AATGGATCCATTCCATCGCAATTGTCTCGTTTGACCCATTGATACTTTCTCAATCACCAAGAAAATCTATCCTCGTGTTTGCATTTCTAAATTTTTGTTC**[G/A]**CCAACTCTCTAGCAAGTTTCAATTGTCCCGAGTATATTTGCATCAATTTCCAATTAACGAGTTTATCATCGCTCGTTATTAAACGAATATCTCTTACATT

>scaffold19.1.498818

GAGGATTTTTTTTTTTAACCTTTTTCCACGTGGTAATTATTTTATCGAATTTTATTCACAATCGTTCTAAATTAAATATTCAATGTGCTACATTTTTCAC**[G/A]**TTATTATTTCAATTATACAAACATTTAATTTCAAGCTAGTATTAATACTGGTTTCAGAAGTTTAAATATTTTCCGTATATTTAATCTTAAACGTAATCTT

>scaffold19.1.545815

AAAACAGAATCCCTTACAAAAAAAGAAACGTACAAACGCCAACTGCCCGATTATCGTATATCAATAGTATATACGGAACACATGCGATAAATAGCAAAAT**[G/A]**CCTGACCTCGTAACCCGTAGATCAGAGAGATGTGTTTGGCGATTTGATGCATTTACAGGTAGGCCAGCGATGTAGAAAGCAGCGCATAATAAAGATAAAG

>scaffold19.1.58016

CGACGCACATTGTGTTTCAATTTTTCTTTCTTATTTATTTCCATTAAAAATAATTTTACGTATTATAAACGATAAATTATCAGCATTGCAAAAATATTCA**[G/T]**CATATCTATTCAGGATGAATTTCGACTACGAACCATGATTTTTTCTTTAAACAAAGAAATCCAAGCACCGTCTATCTTATCGATAATCACATATTTTTCA

>scaffold19.1.670202

GTAGATCTTTATTTTTAATATAGCACAGAATGAAATGCTTGATTCGTTTGTAATAACAATAAAAGGACGAGTTAAATGAAGTTCAAAGCTTTAGCTTTTA**[G/T]**CTTTTTGATGTGCATTCGAATGATCGTTCATAAAGATATAAAGATTATTACAAAGTGAGCGAGTAGAGTTAATTTCTATTTTTAATGAACAGGAGGAATG

>scaffold19.1.690431

GAAGAAAAATCAAAACTCTTTTATGCTTTGAATTTGATAGAATCTTCGAGTTATTAAAAAGTTGTAACGTCTCAAATCTGAGGGAGCTTGTTGTCAATTT**[G/A]**TCTCCGAATGAAACTTCTTTCAAACTTTGAATCTTCTCAACTCGAACTTGAACTCCTTTAACATTAGAATAATTTTAGCAACCCCAAAGAGATTGAAGTT

>scaffold19.1.856048

AGATTCCTAATAAATATTTGTTTAATAATATTACGATGTCGCCATGTTTGACATCTATTTGATATCACAGTTTTCCTCTTTTTATTTATGTATAGTTAAA**[G/C]**TTGTCAGAAAAATTCTTTTTCTATGAGAAGAGTGGGAGAAAATAATATAATTAATGAATATTATATAAATATTAATCTCCCATTCGTTATTTGTTTTCCT

>scaffold19.1.1049906

AAATATCAATTATGGATTATATTCGATTAATATACCTCGATGTTAAAGTTACGAAAAAGTTATATGAATTCTTATAAAAGATTTTCTCGTTAAGATATCA**[T/C]**GATAACTGAACGATTAAAAATTGTTTTTATAATCCAAGGAAAATAAGTTCTTTATTTTCTATCATTAAATTATAAAGTTTCCAAAAATATATTCAAAAAA

>scaffold19.1.1211357

ATGGATAAGATATTTTTAAGGCGAAGAAAGAAGAAAGAATGGTAGAATGACTTTTATTCGAATTTTGTCGTATTCGGTTATGGATGGATACGGATGTACG**[T/C]**ATGTTGTGAATCGTGTATTTTAAATATATGATAGATAGGTACGAAATTATTATTATTATTATACGTGTTAAAATTGTTAAACGATATTTAAATTTTCTGT

>scaffold19.1.144668

GATCAAACTATACACTATAGTCGCACAGATTACGTAACAAGCCGATTAATCGATTTATTAATTAAGAAACCTTTTCTAATTTTATTCCTGGTATAACTCG**[T/C]**AACATGCTTACAATCGTTTCTCTTGGAATCTTTTTTCCAAATTATTTTTATTTCAACGAAATTTTTTAAACGAAACGTTTGGAAGAAGGAAAATTTATTC

>scaffold19.1.1842048

TCTTTGATCTTATTGCAAACATTAATTATTTTTTAATTTTACAATATTATGCGGAAAGTATTCTGTTCAAACAAAAAAAAGGCAACATTTTAGTTCATCA**[T/C]**GAAAATTTGTTTTTATAATCTCTTAAGAAATCATACAATATTGTGATGGATTTTTTAAAAAGTTTTAATTACAATCACAATAAAAAAAAACTTTTTACTT

>scaffold19.1.307142

ATATAATTTCATTCCGAAGAGTCGAAAATTGAACAAGTTATTACGGAGAATTTATTACAGAGAATCACGTGATAATTCGACGATCGATTTGTATCTTCGA**[T/C]**GGATTACCATAATTCTTCGAGAATTTGAATATTCGATCATGAAAACGAGGGCTGATCAAAGAAGATCCTTAATAACTGGTTAAATGCCTCGATTACGTGC

>scaffold19.1.376177

CATCCCACTTTCTATAATATAGAAAAATTGATTCGCGGTTTCGTAAGTCGCGAAAAAGAAGAAGAAGAAGAAGGAAAAAGAGGAAACGAATATAACGGAA**[T/C]**GATCAAGAGGTTAAGAGTATTCTATCCATGCGGTCTGCATCAATAAAGAAACAAACGTAACTAGTTCGAGTGCAGTTCGAGGATGTACTACACTTTCAAT

>scaffold19.1.419689

ATGATCGGATCGAGTGGTTAGGGGTTGATCGAATGAGAACGAAAGATCTTTTTTCTTTTCTATGATTGATGCGAGTTTGAAAAGAAAGAGGATAATTTGG**[T/C]**AGGGAATGTTTCCGTAATGGTTGATATTGGATTTTATGGAGTAGAAGATTTTTGGTTTGAGATTTTTGTTGGTTGCGTATATATTTAGTGTAAGTTCTAA

>scaffold19.1.423203

ATCACAGCTTTCCAAGAACTTCGTTATTCAAATTAACATAATAAATCACTTATATATTCTTTCTTTTTACTTTTCCATCGTTCGAAGATATTCTTCCTCG**[T/C]**AATTCATCCTCTCATAATAATATCCAGATCACTATACAACTCCACAAACCGGACACGCAGCGATAATCTTCGCCCATTCGTTTCAAATCAACGTCCCTAT

>scaffold20.1.1362532

GTTTTCCGACGAATCTTTTTACCGAGCGTCGATAACGTAATCTCAAAAAGAAAAATAAAGCGTTAAATCGCGAATGCTAAACTAATAGAAATCGATCTCG**[A/C]**TTTGTTTTCAGAGAACGCGAGTCGTTGGTGCAGGATAGACGGTATCTGGAGCAATTATAGCAACTATAGCCTCTGTCGAAATCTTCGCGAGCCAGCGATC

>scaffold20.1.1546484

CTGATGAATTGATCATGCCTACCTACGCTAATTTATTTGTCACTAATCTAATTTACTTGTTTCCGTTCTTAATATTTATGCGATCGATGTACGAATATTT**[C/A]**GATTTTCTTCCATATTTTTACATATTAGCTATTGCGTATTATATATATTTCAATACACAAAGTGCAATCACTGTTATCATTTCGAAGCGAAGAAGAACCT

>scaffold20.1.1552903

CTTCGTTTTCTTCCTCCTTTTGTTTTCTCTTTTCTTTTGTATCTTCACACGACGTTACGCGAAAATTTTATTTCGTATAACGTAACGTGTTATTAGGAAA**[C/G]**AAGATTTATGTATTATTGATACGATGTTGATGCAATGGAAAAGCGATTGGTATACGATACGATAGGGTGAGGATTAGTTAATAATAATTGACGAAATAAA

>scaffold20.1.1681378

ACCGTTGTGGGTTCATTCGCGCGGATAAATTAGACGTAACGAATTGTAAAAATGTCGAAAAGAAATCTTGGAAAATCGTCGAATTTTCGCGAGTGAAATT**[C/G]**TTGTTATCTTCTTTGAAAAAGAGGACGAAACGAGGTATCGCAAGTTCAGAGCAACGCTTCGAACTTTCCTCCTTCGTTTCGCGAAGGAAGAGCAAGTTAA

>scaffold20.1.1974297

CAATCACAAGATTCGTAAGAAATATATTTATATTTCATCGAGGATCAATTTTACCTGATATTGCCAATTTTCCTTACAAGAAAAGTATCGATTTCCCTCG**[C/T]**AAATTCCCTTCTCACGTTACCATTTCACCGATCCAGGTATCGAAAAGTCGAGAAAACATCTCGATGGAGGAATCCTCGACGAGTCGTGGCACCGATTAAT

>scaffold20.1.2012512

TATTATTTTATTCTATAGAGATATAAATTCCCCCTTCTCTCTAAATAAATTATTACCATACTAAATTAAACTTAAGAACATCTATAACGTTTCCAAAAAA**[C/T]**GATATCTACCTTGCAAAAATACAGGAAACACCAACGATTATTCACACAAATGACCTCTTCCCACTCGTACCGATATCCTCACCCATATATTTATTTTCCA

>scaffold20.1.1377333

TGATAACATTACAATAATATCGTAAATTATTCTAAGAGGATTTATGTCGCGTAATAGGATTTAAAGATAACAGAAAATTAATGTTAAGATTGGATAAAAC**[G/A]**TTAGAAAATTTAAAATAAAAGAATAGGATAAAAATTGTCCATTCGATTGACTTAAATGTCAAAAATATTTATAAGGTATATAAATTCAATGGAATACGTT

>scaffold20.1.1819510

TGAATATTTAATGTATAAATAGTAGATAAATTATATATTATAAATCGGTAAAAATCGGTACGGTAAAAAATTGGTCATCAAGACGATCTCTTTGATGCTT**[G/A]**TCAGTGGTTAGAATTTTCACGAGATTGAAAGGAATCGAATATTTTCATTTTCATCTTTGAATCTTTCAATTACTATTATTTAATATCATCGATAAAAGTC

>scaffold20.1.1255623

CGATTAATATTCGAATCTTTTTTTCCATCTACAACTTAACCACTTAAATCTTAATAAATTTAAATATTCCTTTTTCTTCTTCGACGAATTTCATGCAACA**[T/G]**CGTCCGCGTATATCAATAAAACTCGTGTAAAAATTTGCAACTCGACCCAAGTATTCTTAAACGAGATGTGTCTATTCATTTTTAAAAATTCTCAAGCATA

>scaffold20.1.1625293

CTTGAGCAAAGTAAAATATATTTATTTTACGGGGACGAGATAAATGATAAGGGGAGGAAATTATGAAAATATGAAATAATATATATATATATATTTGAAA**[T/C]**GGAAGATAAATCGAAGAAGATATCGTAAAAAGTTGGTGGTGAAATAAAGAATTTGTCGATTAACCGAATAAATAAAATGTTTGAATAGTGAGATCGGTAA

>scaffold20.1.1932017

AGTAATGCAATATATATAATTCCCTGATTTCACTGCATTCGATTGGAAAAACATGTCTCGGAGAGCATAAAATTATACGAGAAATAAATTAAAAATAAAA**[T/G]**AATTTAGCAAGTAAAATTCCGCATTGTAAATAAGTTAAATGGATAAACTATATTTTTTGAAACCATTTATTATAAGTTATTTTTTTATAATTTAATTTTC

>scaffold20.1.1960520

CTTTCATTGCTGAAATCTATAAACGTAGAAAAAAATTTGGTAGTGAAAAATATCAAATTATGATATATTAATATTTATAATTATCAACAAAACCATTGGA**[T/C]**GATGTTAATAAAATATCAACTCAATTCTTTTGTTATGATCGTGAGTTTTTTCACACTTAATTTACCTACATATTTCTAGGTAACCATTCGATTTAAAGCT

>scaffold22.1.1133048

TCAGATGAAACATCCCCCGGTAATCATATTTATACAGCAAAGTATCCAGCTCTATACTATTTTTCATCTCGATGAAACGATGAAATTTAATTTATCTTCC**[A/G]**CTCTAAACAACAATAACTGTGTTAAAGTTGGGGAGGAATCTCGAGATCGAACGTTAGATAGGTACGTTATTTACATTATTAATTACGCAATCACGTCGTA

>scaffold22.1.1462544

ACGAAAAGAATGGACGACTGTTGACCTGCATTAAAAATATATACATAAAAGAAAAACAATTTCATATTTTCATCGAATTTACAAAAAAATAATTCTCTCT**[A/G]**TTCCTTCAATTCCAAAATTCTATACGCATACTGTGTGAAGAAATAATAACTAGATTCCTCGACTAGGTTCTCGACACCTCGTCCATATCTCTACAAAAAA

>scaffold22.1.1124916

AATATTTGTATATTTGTTACCTGGACGAGAGCCTAATTGTTTAAGGAGAAAATATTATGCACGATAAGAAATCATACATTTTATATTGAATTTTATAAAA**[C/T]**AAAGAAGAAGAAATACTTGAGAGTAGTTTTATTTCATTTTAATGATCTTGAAAAAAATAAAATTAATTTTATGATTTACCTAATAAAATTGTTAAATTAG

>scaffold22.1.1242600

TTTATAACGTCATTTTCATTTACATTTTTTATTATTGAATATCGAGCATCGCCATTATATAGATATTGTTCTACTTTATTTGCTAATTAGACTTGTTTAA**[C/T]**GATGAAACAATTGTTTTTATTCTTAATTAATCTATTATAATGATTGGCGTATTGCTCTCAATTGATTATAATTGTTCTATTCACCATTTGACTGATCGTG

>scaffold22.1.1263032

TTTTTAAATCTCATATGAAAGAATCATGTACCACATATAATTGCTTAATCAGCTCGTAGTTATTATTATTTATTGATAACGACGATACATGTTGCGATGA**[C/T]**AAATATTCATCGATACATATTCCAATTAAAATTAAAGCACTACTTCGAACAAAATAAAAAAAAAGGACTAATCAATTTATTCTCCCCTTACTCTTTTTCT

>scaffold22.1.1297781

ATTAAATTTTGAATTAATATCTTTATATCTTTGTGCAGCTTTTTTTTTCTATATTCGAAAAAGTACAAACAAAATCGTTGTTGTACATTTAAAATACGTT**[C/G]**AACGTAAATTCTCTACAAGTTTAAAAAGCTCGTCGATTATTCTCCATTCAAGTGGAAATACTTTAATCGTTGATAATACTAATATTAATCACACGGTTTC

>scaffold22.1.1322300

TTATTTCGACCAAAATTATGAATGAAAAATTAATAAATATTACATAAATTTAAAAATTTTAATATCAAAATAAAAAATTGAAATATGAAAAATATGTAAA**[C/G]**TTTTTTTAGCGAAAAAAACTTCATTATTCGTGGTATTTGTGTTATGATTTATATCTTTTAACTATAGAAGCGATATTAACGAATTCTTTACCTTTGTCTT

>scaffold22.1.1430491

TATTCGAACCAATTGGAAGACAAGCTATAGATCGAATAATTGAAATTCGAATAATAAAAATGTCGATGTTTGACAAACAAGCGAAATCATTGAGTACAAA**[C/T]**GAAATCGAGAAAGAAACCATTCACAATTTTATAGAACACGTGACTGACGACTATTGTATATCAGTAATTTATACAATTAAATAAAGATGTACAATCAAAG

>scaffold22.1.815135

CAAGATAATGCATTGTATTTTTAATTTTATTGCGTGAGACCAATCGGCAATTTTCATCGATCATCGTAAGTTAAGTTCGGTTTTGGCGCTAAATTCAATG**[C/T]**AATGATAAGTAATGCATGTTATAATCTTTTTGAGCGAATAAAAAAGGCGATTTTTTGAGCTTCCATCAATCCTTTTGGGATTATAGGAATATTAAACGTG

>scaffold22.1.1418705

CGATACGATACTAGCGCCACGCGACCATTAACTGGTATCTTATGTAATTCAAATACATTATATTTCATACCACTTACTGATTCATATGACTAATAAATTT**[G/C]**ATATTACAAAAATGAAACATGAAATATGTTGAAATGCAAATTTTACTCCATAAAGGAAATAATAATCCCCAATTAATCCATCTTCAATTCGAACAATTTA

>scaffold22.1.1508609

TTTTTTAATATAATATTTTTGACAAAGTTTGATATATATATACGTTGTCGTTAAAATGATATCTTTAAACATTTAGATATCATTAGATAAAATTTTCTTT**[G/A]**TTTCGAAGAAATCGGTTAGAATATCATAGCAAGTTAAAATGATATCCATTGATTAAATATCTGTTCAAAGTTTTCCCAACCATCTGGTGAACCATTTTAT

>scaffold22.1.1513426

CTTTTTCATTATGTTCTCGATGTCTTTTCATTCTGGAAGACAGTAGACAGTGGATAATTTGATTAATAATAGCTTCCATAATTTTATGCATTTTAGATAC**[G/A]**TCAATACGGTAGATTAATAAACTATTATCACTTGAATTCGTGAAGTGTAAGAAAGAAATACAAGTAAATTCAAATTTATTGAATATTGCCTCAATTGGAT

>scaffold22.1.259869

TAGCTGTCATATCTAATGGCATGACAGTAGAATGATTGGAAATAATTGTAGAAGTTTGAAAAGAATTTTTAGTAACTTCTTGTGACTTTATTTTATTCTC**[G/A]**TCTGTGTCTGAAATAATTCCTAAATCTGTCAAGGAAATATTCTTATTTGATAGAATATTTGCTTTTAAATGTTCAGAATCATTTTGTAAAGATGTCCTGG

>scaffold22.1.432225

TTTTCTTATATCAAACTCCATTCATTTTCAGAACTCTCTATTCAGTAAAGATTTGAAGCAACTACTATAAAATTTTCTTTCTTCAATCTCAAGAATTTTC**[G/A]**TCACTTTCGTTTTTGTTTCAGAAATCTGGCTTCGTCTAAAAAAAAAAAAAAATTGAATTTCAACATTTCGAAATTTTTTCTTTCCTTTTTCAAAAATCTA

>scaffold22.1.716994

TCAACTTTTTTTGGTTACTAGAATTCTTTTTATGGTCTTATATTCATTTAATCTGCTGCAATTTCTTGCAATCTTGTTTTTATCATATTCATTTTCTGAT**[G/A]**CCTTTTATTCTTTTTTCGTTTCTATACATATAACTTGCTTTCTTCAGAATCCATATTATATTTCTTTTTATATTATTTTTTATTTTATTGCTGTACTTTT

>scaffold22.1.765104

AGTGCAAGAAAAAGAATCATATTCGTAAATATGGAAATTGAGATTTTCGCAATGTTGAAATATAAATTTATTACTTAAGAAGATTTGAGTCGTCTCTTTC**[G/A]**TTTCTTCAAAAAAAATTACATTTTAATTACTTAATTGAGCTTGATATTAAATTAAATCTAGGATTCATAAATTTTCTTTAATTTTTTACAAACAGTATGA

>scaffold22.1.877716

TACTTTTTTCCTTTTCTCTTTCGAGATCTGACAGAATTTGTTCTCAACGGTTGATTCGTTCGATGTTTGAAAAAAGAAAAAGAAAGAAATGGAAAATTCA**[G/C]**TCCGTTTAATCCTATCTCGGTTATCGGTTACGATGTCGGTGTTATTTTTCTAAATTATCTTTTTCCAAAACTGTTTGTTACATTCGAAACTTGATTCAAG

>scaffold22.1.946204

GAATTAGCTTTCAACCGATGACAGAAAAAATAGATACACTGATTTTTTTTTAAGAAGAATCTCAGTGTAAGCAGAACTGGGAAAAATTTTGCTCGAAATT**[G/C]**AAGAAACAAGCAACTGAATATACTGAAAATAATTTACTACTTGTCCAAAATATTTGAATTGAATTTGAAGATATAGATAATATCAATTTATTTGAAATAA

>scaffold22.1.1064186

TTTATATATTTTTGCTTTTATTATCTAACTGTTCTGGCTTTCGATTTAATTAATACTAATCCTATTATATTTAACGGATTAGCGAAAAGATAATAAATAG**[T/C]**ATGATATTTAAATTATATAATTTCCAGTAATAAAAAATTATTTCCATATTATAAATACGCGATCTTGCAAAAAATGAACAAGTAAATGAAATTTAATTAT

>scaffold22.1.1122970

TATGGAGCATAATCGATTATATATATTTTTTTAATATAGCTTTGCATTAATTAAGGAGATGGAGTAAAATTTCAAATTATTAAATATCCAAATTATTCGA**[T/C]**GCAACATAAATTTTCGAATTATTTAATTCTTTACAATATATAGAAAGACTTTGTTCAAATCTCCATCCATATTGTCTAATATTCAATGTCTCGCTCATTT

>scaffold24.1.391849

ATATATAATCTTCTACATAAAGTACATTTGCTCGATCGTATTTCGAAGCGAAATTTTATCTTTATTGTTATCGTTAAAAAAAAAAAAATCGTAAAATTAT**[C/G]**AAAATCACGAGAAAGAATCATCGTAACAGCGCGCAAATATTCGCTTTCCGTTTTCAAACACGCCAGTCCAGTCTAAAAAATCCTTGCGAAAACATATTTT

>scaffold24.1.434080

TCGCCGATTAATTTTGTTAATTGAGCATTAATAATTTTGTATTCAAAAAAAGTTGAGAATCATAGAGCGAATTATTAATCTCTTGAAGCATTAACGTTTC**[G/A]**TAGTCGGTGCATTTTATAATTTGAAGTGTGTATAATAGAATATAATAGAATGGATGAATAGTTGTATCATTTAAGATTCTCTCACGAGATTCTCTCTTAT

>scaffold24.1.434242

TTGTATCATTTAAGATTCTCTCACGAGATTCTCTCTTATGAAGAAGAAATCTAATGGAGCCATTTTATTGACTTAATAACTATGATTTCCTTGAGATAAA**[G/C]**TTTAATAAAAATTCAACGTCCTTTTGAGAAATTGTACGACAATCTTGATTCCTGAGAAAGATTCCTGAGAGGTGTATCGAGATGTGTACTTTCAGAAAAA

>scaffold24.1.444806

CAAATAATTGGATACGAATTTTTTTCCCCCCCACGAGAATTTTCGAGGAATAATCGAAATCGATGTGTGGTAAATATTTCATAGAATTCAGAGAATCGTT**[G/C]**ACAAATAATAATAAACATACGGACGAAGATATGGAATATAAAAGATAGTGTGATTGTTTTAGAATTTTGCAATAGTTTAGATTATTATGGAAATTGTTTG

>scaffold24.1.645725

TCAGCAATTCCCATAAGAATCTATGGTGAACTAGTAGAACGCTATCCTCTTGAGAAAGAACGACTTTGGATCTTCTAACGAATATCAAGTCGAAGTTTAA**[G/C]**TTCTATTCATACATAATAGATTATGCGAGAATATTTTTTTGGAAAAAAGAAGAAAGAAAAGGAAAATGTTTTCTTGATCCTCTTTTTTAAAAATTCTCCT

>scaffold24.1.661961

TTCTTCGTTTAAATCTCTCTTATGATTATCGATGTAGATCGATTTCGAACAGAAAAAGTCCATCTTAATTATCTCTCTATCTCTAACTGTCTTCGCTACC**[G/A]**TTTAATTGATACACGACCATTCCATCTTCGAATAACAAAAATTCGTAACGCTTCTTGTAAAATTTGTAAAATATCCGAAATAATATCAACGAACCTTCAG

>scaffold24.1.411900

GAATAATGAATTACGTTAAGATAAATATGAAAATTTTAACATATTAATTTAGAATCGTTTATTACAAATACTGACAGTTATTTCGCATCATTTCAATTTG**[T/C]**AGAAAGAATTTTTAATTAATATTTACCCCAATTTATCGAGTACATATGTTTAATTCTTAATTTCAAGAATGACTTTGTCTTTTTCTCAATCTGTGTATAT

>scaffold25.1.646887

CAAAAATATGAACCTTTTATTAGAATTAAATAATAAAAGAAATATTGATTTAACATTCTTCTGATATCGATGCAAAAATTTCTGATAAAAACAGATTTTC**[A/G]**TATTTAAGATCTAAATGGAAAATTTACTAGATTTATTACATTCTTATCTTTCTATGAACCTTTAAAAATTGATTCCTTGTAATAACTTAATTAGAATTAT

>scaffold25.1.833610

ATTTATTTAAAATTATTTAAAATCCTTCGCATTTTGCTTAATCGTCCAAGAATTTTAATCGTTGATATATAAATGTATAATGATAATATGATAATTTAAC**[A/G]**TTACGATACGTTATAATATTGGAAATACGACAATCATACTTTCAATGATTATTAAAAAAGAAAGAAGATCGAGAAAGAAAAATAGAATCTACGTTATTGA

>scaffold25.1.988699

TTTTTACTAAACTATTGTTAATTCAGAATTATATTATCTACAATTTATTTATACTATATTTTGTACTTGTAAAATATACATCATATTATAATCAATTAGG**[A/C]**TTAATATCTTAAATAAAATCAATTTTAATTTATTTTGATTTACGAGAATATTTATATTGAAAGTTTTTTTGAGTTTTAAATAAAAGTTATTTTCTCAAAT

>scaffold25.1.104011

TTGAAATTTGTATAATAATTGATCAATAGTAAAATGATAATGATTCTTTTGATTTATATAAGATTAAATATATTTATCTTTTATTTTTTAAATGCATTGA**[C/T]**GGAACCTTATCGTATTAAAGTTCAAACACGTTGGTTTATTTTTAGTTAAATTGAAAATTATTTGCACAAGTTACAATTCTTTAATTAATTGTAAATTTCA

>scaffold25.1.511290

TTCAACGATTTTATCGATCGTAAAATAATAGATTAAAATGGAAAGTCTTTGGTACTTTAACGAAATTTTTATAACATTTTTTCAACAATTTACGTAAAAG**[C/T]**AGTAAAGTTATTATATCAGTGTGACAACATTGGAAAAAAGCTCATGATCATAATTCGTAAAACTTTAATCGCATTAAAAAGAAGTTGAAAAAAAAAAGAA

>scaffold25.1.560110

AGAGATCAAGTACAGATATTTATGTATTTTAATAAGGAATAAATAGTTATTGGATATAATTTAAAATAATTATTTTGTTACGTAAGTCTAGTCTAGCTCG**[C/T]**AGTTGAAACAATACGCTTTTAAAAATTGACAATTTTTATTTTATAAAAATTGAGAATTTATATGAAGCTCGTACAAAGAATTTTATTATATTCCTTAAAA

>scaffold25.1.635136

GTCATATTAAGTAAGAGGATTTGTTAATTTCGTTAATTAATTTGTATTTTCTTTTTATTTTTCTTGTGCTACTTTCTCTTTGAGCTCTTCATTTATTGAG**[C/T]**GATATTGTAAGAAGCAAGTGGTGACTTGTCTAAATAAAGTTTCTGTTTTATTAAATTATCTAAAAATCGTTATTTTTACTTGTTCGTAATAATAATAATA

>scaffold25.1.770273

TTAATGCGAATCTCGTTTATTTATTTCTCGTCGTCGTGACTATTTCAACTTTTTTTTTTTTTCAAAAGAATCGAGTCAAAATACAATATAAAGGAATTTA**[C/T]**GCTCGATCGATTTTTAAGCCGGCTCATTTTCCGGCTGATCCGGTTTTTCCTTAATAATCTTGTTTTTCCATGCAATTTCTGATAAATTGCGCGAAATGGG

>scaffold25.1.252481

TCGTTAGGAAATGACTTTAAATTACGGAAGAATGTCGAGCAATTGAGTATACATATAAAAAATTTTCAAGAAAATAATTTCAAAGTTGCTCTATTTCGAC**[G/A]**TTACTTTTTATTAACGTATCTAATGATATCAAACGTTATCTCAATGGTAATTATCGAATACCAATTGATGCATAAAAATATAAAAGCGTCAATTTAGAAC

>scaffold25.1.392827

AAAAAAGTTAATATTGGGCTATAAATAAGTAAAAAAAGGAATTATTTTAACATTTTACGTATTTTTTTAATTATTTCAGTATTTTTAGAAAATATAATTT**[G/C]**AATATTTAAGGAATTAAAAATTTTGAAAAGTTATGATAGTTTCATTCGGATATATTTGTTATAAAGAATATTGAAATTATTATATTATCTTTTTAAAACA

>scaffold25.1.503968

ACATAAGTTATAAATGTTTCTGTAATGTTATATTACAATTAGCTTTTATCAAATATTTGATACAAATGTTACATTATTCTTGGAACATTCGAATAATTTC**[G/A]**TTAAATTGAACAGATAAAACTTTTTCAACTTCGTTCAAGTTTCAAATATTAATAGAAGCTTATTTCAAATAAAATGTCGTAAAGTTTATTTTATTAAGTT

>scaffold25.1.684881

ATTTTTATAAAATAAAGAAAATATAAACTTATTTTACCAAGAAACAAAAATCCAATTCTTTCTTTTCTTTGTTTCAAAGAAAATAATATTGAAAAGAATC**[G/A]**TAAATAAACGATTCCTAGAACATCATTCCAAGATAAACATTATTATATTATTGATAAAAAACATTCAAATCAAAGAAAATCATCTAAAAATGATATATTA

>scaffold25.1.477244

AAATTTATATTTCATTGAAATATTTTTTATAATTTATTTTATTCAGTATCGTATTTCAAAATAAAATACCATTGTAAACATTTTTTAATTTAACTTTTCA[T/C]GCAAAAAACTTAACAAAAAAAAATAATTTTATCATTAAAAATTACCCAGAGAATAGAGAAAACCGTGCCAATGAAATCCCTTTTTGTTTTATCCCGATAT

>scaffold25.1.621141

TCTGATTTAAAACAATAAATTATAGATTTATTTGTACGCATGAGATACATAAGATAAAATACCTTTTATATACGTTATCTCTGATATAAATATTACAGAG**[T/C]**AATTTTGTATTTTGAATAATAATAATCCTGATCTTGATGATTGTTAGTATGCAGAAAATCAGATAAATCTACTAGAGAGTTGTTCTAAAATTCCTAACTC

>scaffold26.1.1072854

AATTCATAATCAGTTGTGAATTCTTAATTGTTGAAGCTTGCTTTTGTGTTTCAAAATTAATTAATTGAGTCGAAATTAACCATTTTTGAAATGGAGTAGC**[A/G]**TTCGTATTGAAAGTTACAATGGTGACGATAATAAGAGAAAAAGAAAAGAAGAATATAAATTTTAACAAAACGAAAAATTTCGCGAAACACGAGTTTCAGA

>scaffold26.1.1128750

TTAAACGATAAAACTCGCGTTACTTTTATTGACTCGAATTTTAAAACTTTATTTCTACTAATTCGTATCGTCATCTGTGAAAATCTATTAACCCCCTATT**[C/A]**TTTTTACTTCTCCTTTGCTCAGTTCACTTTTCATTCATAATTCATACTGTTGAAGAATGATGAAACTGGAGTTGGAGCTATTATTGCTATTGGTAATCAA

>scaffold26.1.1249146

CACATAAATATAAAACTTGAATAATAATAGAGAACTCTGTAACAAAATCCAAAAAAAATATATTATCAATATCGAAAAATAAATAATATTATTTTAAAAA**[C/T]**AATAAACATTGAAAATTGGCAAATGTTCCGTTCCTTAGACACCAAAAATGGATCGAGAGTCCTTTATATAAATTTTATTCATACTTTTAACGCATATTTT

>scaffold26.1.1714281

ATAAATCCAGAGAATATTTAGTTCAATAAAGAATTATTATTACTGCAATAAAATTTGTTAAGATTTAATTTAAATAACAAATTTTCAATAATTAAAATTA**[C/T]**GGAGTTTATTAATAGAAAATATATAAATATACAAAATATGGTAGATATTTTTTAATTGATTTTGGATGAATTTTATGAGAATCGAAAGAGCCTAAAAAAT

>scaffold26.1.1746547

CAAGTGACGTGTTGCAAATTTCTTAAATGTTAAATGTACACAAAATGAATTTAGCGAATAAAACGAATAAAGATATTATTATAAATTTGTATAACATATG**[C/T]**AATATTTATATATATATGTAATGCACATATTTACATAGCATATATTATCTGAATCATTAGAAAGAGAAATAAAATGACTTATAGTGTCGTTTAATAAATA

>scaffold26.1.1784457

TGTTCGCTGTATTTGTATAACATGAGGATCCATTGTTGAAATTTTGGGAACCATTAGATATTTTCATTCAATTCTCTCTCTCTTTTTTTCATTTTATATA**[C/G]**TATTTTCGTGATACTAAAATTATTCGCTACAGATTTTGACTTTGTGTTAATCAAAATCTTGTCCTTTTTTCTTGAATTTATATTCTTGATACAAATGGTA

>scaffold26.1.1792564

GCATTTCTCGAAAATTTCACTTTTAAAATAAACGTGCATCTTAAAAACATGTAGGCAGTTTCTTGAAGGAAGAGGGTTGTAAAAATGGAAATGAGATCCG**[C/T]**AAATGGAACATATTTGAACGTGAGTGAATGCATATATGTATAATATTACGGTGCGTATATTAGGACCATATTTAAAATCTGGGAAATATTGTATGTATAT

>scaffold26.1.1867654

TTTTCATAATCAATAAATCATGTTTTTAGTATATTATAATATAAGTATAATATAATATATTATTTTGAAAAATGTATTAAATCAGTTATTATAGTGAGAG**[C/T]**AGTTTAAATAAGATAATCTAATAATTGGAAGTCATAATATGCTATTTTTTATATATTTTATTAAAATTATGTGTTATAAATGTTTAATTTAGAAACACCA

>scaffold26.1.393969

TCCAAAAAATGAGTTTCGAAAGAGTTATGGAATTCTTTTACATGGATATTTCGTTTGAACAGGAAGGTTTCACGGATCCTCCAAGTGTCAGTTCCATCAG**[C/T]**AGATTACTTCGCGGTGGACGACCTGGTGACGATGGAAAAAAAGATTACACCATCGATGGAATATTAGGCGGGGGTGAGTACAAAATGATAATTTTATATA

>scaffold26.1.706573

GTTATATACTTTGAATTCGAGTTGTGAAATAATAGAATTAAATATGTGAAAAGTATTTCAAAATAAATTTATTTAAATTAAATATCCTTATTAATAATTT**[C/G]**TAAAAGTATCTATCAAATAATTTTTATCAATAAATAACGATAATACATTGAATTAGAAATAATTAACGCAAGAAAAAAAATAACATATTATTCGACTTGT

>scaffold26.1.761387

GTATATATATGTAATGTATACATAATATTAGATTTCGAATAATGTATACATAACATTAGATTTTGGAAGGAAATCGAGTTTCGACTCAGTTGAGAACAGA**[C/G]**TTTCTCCGATGGAGGATGGTTTTATATAAAGAGATAATGTAAGGAGAGAATTTTTGTTTTTTATAATAATTATTCAAGTTTTTATAATAATTATTCAAGT

>scaffold26.1.808239

AACGATCTTTGGAATAGTCGGCATTAAATCGGGTGCACGCGTTGCAAAAGCTATTTCCTGGAATCAGTTTATCGTCATCAGAGCAACGCAAAGCAGACTA**[C/T]**GTACGTGGCGGAAATAGTGATGGATCGAATTGCATCGTCACGTCTTACATAGTCATTTATATCACAAAAGGCACTGGATCCTAAATTGTATTTGATATTT

>scaffold26.1.1006482

CAATCAGTTAAAAGTTAAAAGTATCTTTCCTAAATGATCATTAGACTGAAGAAACCTGTTGAAATGTGCAATTAATATAACAGGATTCAAAATTAAAATT**[G/A]**TTTAAGCAATTAATGATTGACTATACAAAAAAATGTTATATTTCTAAAATCATTAATGACTCAAAAGAGATTTCGAAGATTCGTCGAACGAAAATAATTA

>scaffold26.1.1143720

AGAGGAAAGGAACAACAGAAATAAAAGAATTTTCTTTTAAATTCAATTGAAAAACGCACGAAACAAAACAAATAGATAAAATAATAAATGAATTACCTTC**[G/A]**TCGTTGCTTTCATAAAAACGTAAAAAAAGAGGAATCGATTTAAATTCAGCGTCATACATTACATGTTTTACACGTAATCGGCTATTCATTTCGAATCCCT

>scaffold26.1.1231525

GATTGTATTGAATCTCTAAATTGTACTATGTATCTCCTTTGCACTTACTTTGAAATGGAGGATAAAGAATTAGAATCCAAACGAGGATAACACACGCGCT**[G/A]**CCACTTGCTCTTTGAAACAATTAGTTTCCATTAAAGAAAATCTCTTGTTACACATCGTGGAAAATTTAATTTTTAAGAAAAATCAAGTAAATGTTCCATT

>scaffold26.1.144081

ACGTTTAATTTGCAATAATTTTTTCAGCTGTTTCCGGAAATTGTTGTTGCATTTCCAATGAATCAAAGTATTCGATATATCTCGAATACATATATTTCCC**[G/A]**TTGAATATTAATCACTTAGTAATTTTTTTCAATCTGAGATATTTTTTATCTTCGCGAAATACTTGTATCTCATGTCGAACAAAATTTCTTTTCACGATAC

>scaffold26.1.1485148

TTATTAATTGTACAAATAGCCAAACTAACAAATTGTAATTTTTCTAAATACTATTTCAACGCTAAAACAGAAATTCTAAAAACAACGAGAAACATCTTTC**[G/A]**TATGAAACGAAACAACAACCAAAAAATATCGAAACGTAATAATCTACACTTCATCAAGCCTTTCGTCGGCTACAGTCATTTAGATATATTAATCCCTTCA

>scaffold26.1.1507458

TCGTTTTCATTTTGATCAAACATGTTGACGTTTCTTTAAAAAATTAAAAGCATTAATTTATCAAATAACGTATAAAAAAATTTTTGAAAAAATTGCAATT**[G/C]**ATCAAAATCGTGAAGAAATAATAAAGATCGCTTTTTACAATTTTTTTATCTAAGCCTATAATCAGAAGTTAAAACAATACAATTTTGTAAATACGTCAAT

>scaffold26.1.1547143

ATTAATAAATTTCACTACGAAATATCATAAAAATCATTTATGAAATTATATTTTTATTAAACTTTTTAATAAATAATAGAGAATTACTATTAGCGATTCT**[G/A]**TTAAATTATAATTAATTAAATCGTAAATTTTTTGATAATTATTGGCAACAATGTGAATTAAATCATTTTATAAAATTGTAAGTAATAATTATTAATTAAT

>scaffold26.1.1572849

TTCTGTTTTGTTCATTCAATTCTTCTCAATATTCGAAAAGAAACATTTCTACTCTCGTTGGTCAAATTTGAAATGGTCGACGACCATGAAAATAGAAATC**[G/A]**TGAAACCGTTAAAGCCACACATTTTATGAAACTTTTACGTCAGAATAAACTATGGATTTAATAGCTCATTGTAGATGAAACGGTTGAATTATTAATGAAA

>scaffold26.1.1700482

ATATAATATAATATTTCAAATTGTTGGATTAAGAAGAATATTTTTATATTATATTGTATATATAGATAGAAAATTTTTGTTGATAATGACATTATATTTT**[G/C]**AAAATACAAATAATAAATTAATGTAAATGAATAATAATAAAACAGATATGAAGTGAAATTATATTTTCTATTCAGTTGTAATGTAAAACATGTTAAATGA

>scaffold26.1.2106654

AAAATAAAATATTAGTAGCAACAAAAGTGTTAAATAGTTGAATGAATATAGCACAAGTCTATGTTATAAATAAAATCAGACAGACAAGATTAATTTTATT**[G/A]**CAATAAAAAAGAATATAATTTAATCAAGAATTTGTACCACGATTTCACAATGAAATAATGAATGACAAGCATAAAAATCATTCGTTTGAAAAATTTGAGA

>scaffold26.1.255038

TCCATCTTTTTAAAGAAATGTATAATTACTAATTATAAATTTTATACATATAAATAGACTGGAATAATTTAACAAAAGTCAGAAAAGAGAAAAAATTCTT**[G/C]**ATATTAATTAGTATCAATGCATCAATCATACAATAAAAGGAAATAGGTAATTTTTTAAAAATTAACTTTTCAAAAATATCATGTTTGCTTGATATGAGCA

>scaffold26.1.882640

TCGAATGTATTAATTTTTAATTTCATTAAGTTTCAATATTAACGATCATCAATTTTCCAATACTTTAAAATACATCTCTATCGATATAATCATTAAAATC**[G/T]**ATCAAAGTGAGCAAAATTATTATTAATATTTATTGGCAATCCATATGCTTAAACAAATTAACGAAAACTTGCATTCATAGTTGATAAATATCACTATAAC

>scaffold26.1.895297

CATCTCAGCAACCGATTGAGATATCGGAATAAAACAAAAACTGACCTCAACGGTATAATTTTCTCTACTTCCTGAGCGTGTTTCGATGACAAAATTCTTA**[G/T]**CTCTTACTTTCTTTTTTTTTTTTAATATGGAAAATTGAAATTTCATTCGTGGAATACAATGTCTGTTATGATATAAAATTTGGTTTAAAAGATTATAAGG

>scaffold26.1.951028

CATTGATTCTGATGAAATTTTCAGATCATGTAAATTTTGCTCCCTTTACAAATCATTTTTGCAACGTTTCTGAACTATTTTGTTTTTTTCAATCACAGAA**[G/T]**CTAATAAATAATAAATAATAAATAACAAAAGAAAAAATTAAAAGTTACTCAAAGGAAATGGAAAAATTAAATCGTTTCAAAATATATTCTTCTTTTATAT

>scaffold26.1.1016570

GCACCGCGGTGGAGAAAAGGGTTTAATATAGACTCATAAAGTAAGGTGAACTTCTATGCCTTTTTATTAAAATTCATGCAGCAGATTAAATCAAAATTAA**[T/G]**CCCAAATTAACCCTTTCACTTTTACCATCTTGCAATTCCATCATTTTCCAAATTATCAATTTCAAAAAATTTTCCCTATTCCTTCTAAACGATCCGACAA

>scaffold26.1.1058878

ATTGTACAATCAAAAAGGGAACAATGAGCGGTGAATAAGTTATAAATGAAAGCCTCTTTTTAGATTTACCGAATGAAGGGTTGAATTATTAGGGAATTCA**[T/C]**GGTTAAAAGAAGATAAAGGAAAACTTGAATACGTTGAATCTTCAATTCAACATAAATTAAATTCTACTTATAGAAGAATAATTCTCTAACAGATATCACT

>scaffold26.1.1172703

TAATTTTCAAAACAATTAATCGATTTATCTCCTACCGTTTACAAATCCATCTCTCAATCGTGTCAAAGATTAAACGAGTAATTTAACGAGTCGACTCAAG**[T/C]**GGGGGACGCTTAACTACAAAGGGCACTTTTGTGTGAAGTTTTTCGAATTTAATAAATTTACAAATTTCCATAAAATTAGCAATTATTGCAGGCATTTTAT

>scaffold26.1.1437570

TTTAATTTAAGGAAAAGTGAGTATAAGAAGTAATAAAAGGAAGACATTAAAGTCAAACAAATCGAACAAAGAAAAATTGCAATGCAACAGAGCAAATAAA**[T/C]**GAATTTCTTCCCCGCGAAGTTTAATTTCTTGCAAAACAGAAATAGTTAATAGAAAAAATAAATAATTAAAAGGTCAAAGAAGATAAAAGATTGTACTGTA

>scaffold26.1.1898375

CTTTCTCTCTATTGTCTTTTTTATTCTCTCTCTCTCTCTCTCTCTCTCTCTCTCTCTCTCTNNNNNNNNNNCGTGGTTCTGTAGATAGTCTGGAGAATTA**[T/C]**GTTAAATCATGTCAGGTTATATTGGGTCAGGATGCTTCCATGTATCATTTCATTCTCGTTTCTCTGCCTAACCTGACCTCATCGGTGTTCCTGCATTTCC

>scaffold26.1.2282349

AATATTAATATCATATTTATAATTAAATTATAATATATACTTTTTACATTACATATAAGATATAATTGTATGTATATTTTTTTTATATTAATTTTATTTG**[T/C]**ATGATATTTGATTTTATTTTAATTGAATTGAAATCCATAATCTAAAAATAATCAGTTTATAATTTGAAATTTTAAAACTTATTAAATGAAAAATAAGGAA

>scaffold26.1.37497

ATTCAAAGTGAGATAATAAAATCATTAGTTGAAAGTATACTTTGAGATCTTGTTCATTTATATTTTAAGAATTCTCGAAATCTTCTTGTAGTTTCTGTAA**[T/G]**AATGTTAATTGTGAAAAAAGAAGTTATGGAAATGATTAACAATATTTATCACATCCTCCACAGTTTGTAAAGTTGGAGAAAGGGAAAGAGAGAAAAAACA

>scaffold26.1.573717

CGAGAACATGCGTACACATGTATACGCACACGTGTATAATAATATACGTGTATGGTGCTTCCTTTCTTCCATATTTTTCTTCTACGTAGAAGACATCTTG**[T/C]**AAGTAAAATTAATTCTATCGAATTATATTGAATTATGTCGACATTAATTTTCAATTTTTCTCCAATTATGCATATTTCCACGCCACAATACACGTGTGTA

>scaffold26.1.595956

ATATCGTTAAAAAGGAAAAAAAAAAAAAGAGAGAGAGAAAAAAGGTATGAATATAACTGTAATTATCGTTGAACTACAAACGGGACTCTTCGACGTATTA**[T/C]**GTTCAACTTTCCGTGGACAAAGTGGGTCGTTGACGATCTATTCAGTTACTTCCAATTTTTGTTTTCGTTATTATGAACGCTTTGTTTAAAATTTTCAGCA

>scaffold32.1.133004

TAGGATGTGAAATGTATTATTTAAAAAAAATCGATGTTGATTATATTCTTTTTGATAAATTACCAGAAACGAAAAGCAGAACATGTAAAACTATTTTATA**[C/G]**TTTCGTAACTCCAATTTCATATAAGATTAACTCTTAGGATGAATACGAGTGAAAATATCAATCAGTGATATGACGACCAATAAAGAAAGCTTCATTATTA

>scaffold32.1.165052

TAAAAGATTAACAATTCCACATAGATTGCACTAGAATATATATAAAAACTTAATTCTTCGTTTCATTTTGCATGAATATGTCTTTGTGGAAAAATTCAGT**[C/G]**AAAATTCTCGGATAAATTTCCAGATACACTGAATTTCAATCTCTAATGAGACTTTTATTCTCATTAAAGCTCAACTATATCGATCAATATCGAAATATTT

>scaffold32.1.204210

TTCAATGACAGAGTGGTGTATTAATGGAGAAGAAAAAAAAAAAAAAGAAAAGAAAAAGATAAAGAAATGTAGAGGACAGAAAAAATACGAAAACGAATAA**[C/G]**TTAAAACTGGATGCTCGAATATTTTGATTCGTTTTCTCATGATGAATGAATTATTTTTGCGAAAATACAATTTTATATTGATTAATAAATTATACGCGTT

>scaffold32.1.27896

GAGAATTGTCGATGTAGGAATTGGTATTATTCTGATACGTTATGATATATGCAGTGAATGCATTTTAAAATTTTACTTCGTAATTAAAACTATGTAACTA**[C/T]**GGAAATATAAACAAAATTTCGTTTAGTAACAATGCGAATGTTTAATTCAAAATTATCATATAATATAAAGATAAAAAATATCACGATTACATAAGATTTA

>scaffold32.1.28175

AAATTGTTACGAAGAGAAAATTAATCATCCCAAATTAATTTTATAGAATCAACAATCAATTCTCTTTGTACGTCTTTTCATTAGCAAATTATGTAATTCA**[C/G]**TAGAAATTCATATTAAAGATTTAGATCTTTCACTTTATCTCTTAATTATCTTGTATTATCTCTTAATCACATTGTTGTATTATATCTAGAATAATATTAT

>scaffold32.1.356001

GTCCACCGACACGGATTGTATTCGAGTCGTGAAAGACTCTCGACATTACTGCTGCATACGTGAGAAAATTCGATATTTATACGTTTTAGAAGAGAGTAGG**[C/T]**ATGTTGAAGAAATAAATTTCTTGGATATTTATTATTTTCGATTAAATTTTTTTATTCTCATTCCCTTATTTCGATTACTACTTACGTATAATTCAGATTC

>scaffold32.1.437671

AATAATAATATTATATAATAAATTAAATATTCTTTTGTTTTTTTTAAATATAATCTAACATAATATATAATATATGTATATAATAAATAATAGAAAATAT**[G/C]**AGTTTCAAAAATTTGTCATAGAATACAGATTTGTTTCTCTTTTTTACATAAAAAAAAATTCTTATTGACAATACAATTACATCTAATACTTATTTTGTTA

>scaffold32.1.125762

AATCCGATCGAGTGGAATCACTATTACGAGTTTTAACAGAATTAAAAGCTTCATTGTAAAATTGTCAGATATAATAAAACCATAGAAAATATCTTTCGTG**[T/C]**ATTTCACATACACTCTCTAAATTTAATTTATCAAATATTTTCCACACGTTCGTCGTGGGATTATTATTTCAAATGCAAGATTAACTTTTAAATAAATGAA

>scaffold32.1.158629

TTATGAAATAATATGTCGTAAAGAAAAACACAACATGAAGTTATATTCCTATATAAAATAATGAGAAAAAAATATCATGTTTACTTGTAGTCAATAAATG**[T/C]**ACTGTCACAAGTTGATATCTTACATATTTTGAACACATATTGAGTATGATTTTATATTGTTATTCATATTTTTATTATTAAGAAAGAAAAAAAAGAAAAT

>scaffold32.1.261886

CATTAAGTTCTGGCTCGACGATCATTTTTCATGGTCAGGTGAGATAGAATTTTATATGACGCTTCTTGTGAATTGTTGAACAGTGTGAATAATTTGGCGG**[T/C]**AAATGATGTTATTATTTGATAAGAAGAAGAAAAAATCGAAAAGAAAATATTTTCGAATGGTTTACGGGAACGAATCATTTAGAATGATTGAAAAATTTGA

>scaffold32.1.289355

ACCATTGTCTGTCATCAACTATCATACTACGATCATCGAATAATGGCCATCATTCGAACCATAAACATCATTGTAAATCTTCTGATATCAAAATTAAATG**[T/C]**AATACGTTGATATTTATTGGCATTTATATTAGAAATTATAAATTATGATATTCGATGATAATCGATGATTATAAATTTACTTCAATATATTAAAATTTGA

>scaffold32.1.386882

AAAACGATAAACAACATATGATTTCGAAACATATTTTTTGTGAAAGCAAAGTAATTTTAGAATTTTCAGACAAGCTCTTTATAAAATTTGAAAAATTTAA**[T/G]**AAACGTGATTTGTAGATATTTAACTCTGAGAGCAGATATAACAAGAAAAATAAAAATACGTGCAATATTCACATCATCTCAAAGTTTCCAATAAACACAG

>scaffold35.1.3100144

AAAAGATTTTTATACAACCCCTACGGTAAAAAATTATGCTTTTATTAAAATAAAATAAATTTTAGCTTATATTAAATTATATATTTATATAAATAATCAA**[C/T]**GATATATGAATACTCTTGATAAATATTATATCAAATATTATTTTTGTCTGATTTTCTTAGCCTGAATGGGCAAGACCACCAAGCACAACGGATAATGTTC

>scaffold35.1.3147930

GCTTGAGGATAGTTGTTGAATAATTCTTCCTTGAATTAATTTTACCTCCTTGAAGGACCAATCCGATACAATGATTTCTAAATGAATTTCCTCTTTGTAA**[C/T]**GGAGATATATACGATTGTTCCATGTATTTTTCAAGAGTTTTCATACGCGATCTCGAGTCATTGTGTCATCGAAACCAAAAATAATTCTATCTTACAACGA

>scaffold35.1.3700086

CACAGTATTTACGTAGCAAATACCAACAATGATCGTCCACGTGTTTATTGATTTTCTTGACGTTAAAACTTAAAAATTAGAAAATAAAAATTGGAAGAAA**[C/T]**GAAAGTAGGAAAATGTTCTTTTGAATTGATGCATCGAATAATTCGAACCACGTACATGTGTGTATCGATACGCGTGTAAGAAGCGCGATAACCTGATTAT

>scaffold35.1.3500879

AAAGTAACTAGTTAAAAATCTCGAATAAAGTTACTTGAATTGATTGTTTCATTTATAAAACTGCTTGTATATAATAACACACATACATATATAATTGTTT**[G/A]**CCATTACAATTGCTATTACGTACCTTGATCGAGGTATAACTTTATTCGTGGCTTCGACTTATTAAATTTTTATCCCATCACTGGTTGTACAAAGAATATA

>scaffold35.1.3712639

TAATCGAATGAAATACCCGATTGACTCGATTATAACGGATCATGTACCGATATTTGAAATGTATTTCGAGCCTCGATATTCTATCCTTGGAGAATAGTTT**[G/A]**CCAACGAGAGAATATATTTCGCGTAATTCTTCGTATAATTAATGTATTTAAATGGACGTAAGTACGATGAGATGTTGTAACGTTAGTACTCCATACTCTC

>scaffold35.1.3510359

CGTTTCGATATTTAAAAATCGATTTTAAAGAAGATACGGGGAAAAAAATCGATCAAGAAGTATCGAATTGTATTAAACGAGAATAAAATAAGCGGCGCGA**[T/C]**AAATACGTTTTAATTTCAAAGCGAATAGTTTGGTGAAATTCCGATGGGAGAGAGTGCGATCGTTGCGGCGAAACCGTGTAATGGCGCGATGAATCGCGTG

>scaffold37.1.310734

AAATATAATGGATATTCATACAGTTAATTTGGTTAAAAGATTTATTTAATCATTTACGAAATGATTTCGTTCGAATTCTATTATAATTTTTAAGTTAATT**[A/G]**CTATGCAGAAGGAAAGCATTTTAAACGAGAATTGCGTTTAAATGCAATTTAAATCAAACTGCATTATACTCATCCTGATTCATTGACGTCAATCTATGAA

>scaffold37.1.51101

ATGGCATGCTCAATCGTGCACGGCTTGCTTTCAATAATACCTTTCAATAATCCGACGTTCGGCTTCACGCACGAACAATGACACTTAACGAGAGTTCGTC**[A/G]**TTTTAACTTTGCGTGCGCGGTTAATCTTTTATCGGCGAATTAATCACATGAAAGAGGCCACCTTTTTCTATATCGACGAAAAACTGAAAATCTTGATTTT

>scaffold37.1.194024

CGCATTTATTAGAGACTAAATTCTACCTGATTCTCGATTCTGACGTAATTCTCACTTTAACATTCGTAGAAACTTGAGAATATATCACAACTCGAAGATA**[C/G]**TTTTACGTTACTTTTAAAAATCGCAGAAATTCTTCATTTTGAACGTCTCTTCTAAATTTTGGCTGGGAAGATCGATCGATATATTTTAGATAATTATCGA

>scaffold37.1.195156

GATATATTGTCAGAAAAGAAATTAGAACTTATTATAGTTATTGGCAGTATAGAAATAAATCGAGGAAGCATTGGATGGATATACGTGTGATGACGTAACA**[C/T]**GAGTACTGCGAAAATGTGATTACTATATTGCAAATACAATCTCTGCAGATATGACGACATTCGAGGTGTCTCGCGTTGAGATTTTTTCAGACGTATCTCG

>scaffold37.1.244233

AAATTGTGATTCATCTTCAAAGTGGGGAGAAAAGGTGCAAAATTATTATTATGGTTCGTAATAAGCGAGTAACATTTTATTAGAGATAAAATATTAATAA**[C/T]**GATATTGACAAAATAATGAAACGGTGGAGAGTATAAAAAGTAATTCGTACATCATTCATTCAATCAGTTACGGTTCGTTATTCATATCCTTCGATAAAAC

>scaffold37.1.2639

AACATAGATTTAATGAATGAACGAATATATTTTTGTATGAAACATAATTTAATGTAAAGGAAGTGGATGGATATATATATATATATATCTCTAAAATAAA**[C/T]**GAGAAAATTGTTATTATTTTTATGATGAAAAAATACGTGATGAAATAATAAATAATAAATCACATATAAAGAATATCTATAAATATGTATCGAATATGAA

>scaffold37.1.310740

AATGGATATTCATACAGTTAATTTGGTTAAAAGATTTATTTAATCATTTACGAAATGATTTCGTTCGAATTCTATTATAATTTTTAAGTTAATTACTATG**[C/T]**AGAAGGAAAGCATTTTAAACGAGAATTGCGTTTAAATGCAATTTAAATCAAACTGCATTATACTCATCCTGATTCATTGACGTCAATCTATGAAATCAAT

>scaffold37.1.125958

GGGCAAAAATTATCGCGATGCTTTTGTCTTTACGTTTTATCGACGATTAAATTGTCCTTATAATTACAATCGTGCAGGATCGTTAAGTTTATTAACCGTT**[G/A]**CTTAGCAAAGGAGCTGTATATATCGCGAAAAATAAACGCAGAACCGTAACAAAAACAGGACACGATAAATTCCTCCCAATAAGGCTGTTCCAAATCCGAC

>scaffold37.1.223047

TAAATTATATAGCTCAGAATGCAAAAGAATTATTAAGCTGTGGCATATGATATGACCAATTTTACGTACAAAATTATGCGGAGATGAGACTCGATGTATC**[G/A]**TTAAATTTTATTTTAAAATTTGATGAAACCTGGATGAATTGACAATTTAAATATTGTTACAATGAAGCAAATTATATATTGCGAATTATATATTACGAAT

>scaffold37.1.442769

AAGCCAATGAAAAAAGCACTTGCCTTGACATTTTCTTCTGTCATAATATTAGTAGTATTATTATAAGTAATATTCCTTAAGAAATAGATTCATTTATTAC**[G/A]**TAATTTGTTTTAAAACATTATCAAAGTTTATCCTTAAACCTATTAAAATTAAGAAAAGTTTGAATCGCTACAACTCCGAAGATAACAACTTTCGATCAAT

>scaffold38.1.2399619

CGTCTGCCAATACAATTTGTAAAATGGAGAGGAGCGAAATCAATAATAAACTCCCCCCAAAAACGATCATATCGATACAATCGTCGAAAACAAATCGCCC**[A/G]**TTCGCAAAATATGCGACAACATCAATATACGATGGATTAATAATTATCCGTGGAAAATATTTTTCCACTGTGAATAATAACAATGACGTAAATCATTTCC

>scaffold38.1.2436925

GTATTCGTTTAAAATCTTCAAGAATTAAAATTTTATTAAGAACAAATCAAATTTGATGTAAATGGTTACTACTATTTTTCATAATTAAATAATATGGAAC**[A/G]**TAATAAAAATGAAAGATCCATAAAAAAGAATTATATAATGTAATAATACCAATTTAAAATCTGTACCATTTTCGTAACAAATAATGCTCTAGATATTAAC

>scaffold38.1.2832864

TTAATGCCATTGCTGCTGATGACATCCAAGGTTGAAGGAAGAATCCAAAAGAACTAAATATTCCAGCAGCAATGGGAATACCCAATAAATTATAGATACT**[A/C]**GCAAATAAAAAATTCAGCCTTATTCGAAGAACCGTTTTTCTCGATAAATCCAGACACGCGATAACATCCAAAAGATCATTCTAAATTATTATTATTATTA

>scaffold38.1.1745418

ATAAATTGTATTTTCTTTTACTAATTTAGTTTAAACGAAAGAGATAATTATATTTGTAAAATAGAGTGGCCATCGTGTGATTTAGAATTTTATTTGGCCA**[C/T]**GTTTTCAATAATAAACTCATTCTCATTCATTTTCTCGTGCTTATACTAAGCTATACACGAGAAGGGAAACATGAGAAGTATATAGCACGAATAAGACAAG

>scaffold38.1.1954819

TAAAGCGAGCTCTCTTCAGAAATTTTCTTTTCCTGTGCCTTTACCTCTCCGCTTCCTTTTCACTCTGACTAATTTTCTAGCGGCAGGGATTCTCAAACGA**[C/T]**GATAAAGTTTCTTCGCTGGTTGTCGATTTTTCATTTATTCGTATAATACTATTTTCACATAAATATACAGACTGTTTATTTGTCACTTTATTTATTATTT

>scaffold38.1.2261002

GTGGATATTTGATGATTATCGTTGGCTGACCGTGAATATAAAGTATTTTTTCTACAACGTCGCATTAACGATCGTTGAGTGCGTGATTATGAACAAGCTT**[C/G]**AACCTTTTGAAATTATTGCTCGCGTTTATTTTAAAAAAAAAAGAAAAAACAACGTATTAATAAATCGAGCTAAAATTCCATTTTCGATCGACGTCATCTT

>scaffold38.1.2326240

ATATATATATATATATTTGCATAAATTATATTATCATATATGTTGTTTCAAAAATTCAACAAATCAACGAATTAATCTTTTGATTTCCAATTTTTTTAAA**[C/T]**GTAATGTATAGTAAAAAATATTTATACATCTTATTTATTATTAATGTAATAATTAATCAATTTAATTCAAATATTTATAATCATACGATATTGTATACAA

>scaffold38.1.2406775

GAAGAAAAAACTTAAATTTGAATAAAGATAAGAACAAAAATAATATAATACAAGCATAAGCAATCGTTTAAGAGCAAGATGAAAAAAAATGGAATGTCGA**[C/G]**TTAATTGACGGCTCGTTTCATTAATAGCTCGACAAGAGTATTTATTATCGAGATTCGAAATGAGTCGATCAACTGCGAACGTTTCCATTGTGGATAAGAA

>scaffold38.1.2580263

TGATGCTCAATGGACGAACTGGACATCGAATAAGAAAAAAAAAGACTAAATCCGTCATTGCGAATTCACAAACAGAGTTCAACGAAACCCTGACATTTGA**[C/T]**GTATTGTATAATCAATTAGACATTGTGCAATTTCTCGTCGTGTTATGCAGCAAAGTGTGTATTAATTTTAATGATAGTTTAAATTATAATGAATAAATTA

>scaffold38.1.2611539

ATTTTCTTTTAAATCGTTGTCGAAGAGAAAATGTTCATCGTGAATTTTTAAGATATTCATCGTTTAACTTGACGAGGAAATACGGAAGTTAATTACTAAA**[C/G]**TTTTTTTCAATTTTTTCAAAATCAAATCATTCAATGCAAATATTGCGTAAATAACATAGAAAAGTAACAAATTATTTACGAAATGATATATAATAAAAAA

>scaffold38.1.2639615

GTCGATTAAATTGACCCATTACGTAACTCAAATCCTTTCGAACACGGCATTCGTTTGACTTTTGGTTCAAATGGTTATACACAGTGGTATTTTCGAGTGG**[C/T]**AATGAAACCAATATCGTGAATATGTGAAACTATTTTGTTGGTTCGTTGAGAAAAAAAATATAAAATCACGAGAAAAGAGCGAATGATTCGTTCAGTTGTT

>scaffold38.1.2690969

AGTTAACGTATATTTTTAGACTTAGCAGCGGTGGCGATAATGGTAGTAATAAGGTTCGCAACGCTGCAGTAGTGAATGCAACGAAACAAGCAAGAGGTGA**[C/T]**GCAGATGTTGTAAAGAAGCGCGAAGAATTACAAAGGAGAATAGAGGAAACTAGAAGGAAATTGCAAAGTGTATGCATTGCGATATTTATTTCATAAATTA

>scaffold38.1.1906613

GGTAAAAAAAAGAAAAAAAAAAGAAAAGAAAAGAAAAGAAAGTGATACAAAAATTTTTCGATTCTCTTAAAATTCTCAAAATTTTCACAATATTTTATAC**[G/A]**TTATTCTCTTTAACAAAGAGTATATATTTTTATTCAAAAAATAAAATTTGAAATTCTTCGAATAATTCGGATAATCAATAGAAAGACAGTAACAATAATT

>scaffold38.1.2024202

TTCAAATTTTCCCTATTGAATTTTATTAAAATTTATTTTATTTTCTTTAATTAATTGTTACGTATCCATGTACAGTGGTACGAATTTCTCTTTTCCTTTC**[G/A]**TCCACGAATAACGAAAGAAGCGAGCAAAAAAAACGAGAAACAAACCGCCAATCTCGTCACAATCGTGCGACAGTTAACACATCCTGTATAAAAAGAAATT

>scaffold38.1.2210971

CACACGTGTGAATGAAACGATGCTGAAATACCAATCCGCCTCTATTTTTTAATACAATCATCACTTATTACATTACCAACTGTGTAACAGCATCTGAAGC**[G/A]**TCAGTTAACCAAAAATATTTCCAAAACTGTAAAGTTTTATCTGTATAAACAAGAGATAACCACGGAGCACTATTATAGATATTTCTCTTTATCTTCCTTT

>scaffold38.1.2242482

AAATTGTCAGTAAATATTGTGATTAAAAAACTTTTCTAATATTAATATTTGCATGATTTGTTCAAATGATCTTGATAAAACACTTCTTTGCTAATTACTC**[G/A]**TTAATTTTTTATATTATCCATTTCTTCTAAATTAAATCTTTTATTTAATATTAATTAAAAAGCAGCAATTTGTTTTACATTCGAAGTTTGAATAAAAACC

>scaffold38.1.2433664

AAACAGCGCGTAAAAAATTCCATAACACGAATAAATAATAATCTGTCACGAAAGCAGCTTAACGTTTACACACACAGAATACCGAAAGGATGCTCTAGGT**[G/A]**CGTAAAAACTCGATGGGACGTAACCAACCTTGACCAATCGAGATAGAGACCGATTTTTCATGAAATCGCGGATAACGAATTCCTTTGGAAGAAGAAAGAT

>scaffold38.1.2439704

ATGACACGAACTTGATTATCTGAATTTTCCTTTCTTATTATCGTCGAAATGCTATCATTCCACTTTTTATGATATTATTAAAAATGTGAAATCTATCTTT**[G/C]**ATACAAAATAAATAAAAAATTAGAAAATTATTATTTATTTGTAACTTTCAAAATTGTTTCTAAATTTTTAATATTTAATATCATGTATTTTAGAGAAAAC

>scaffold38.1.2529980

ATATTTAAAAAAATATCACAATATTTAATAGAATAGATACATTTTTATAATTATCCAGTTAATATTTAAAAAAAAAGTTATAAATAGAAGAAAAATACAT**[G/A]**CTCCAACGAATGAATCATTTTCCTTCATATTTTTTCACATTTTTTTTTCCGTCACGAACTAGCATCCGAGACACATTTACCAATGATCAAGATGTCATTC

>scaffold38.1.2660404

CTTAACAATATTATCGATATATCGAAAACTAGTTTCTTAGAATGTTTATATAATTTATTTTAGAATCATTTATATAATTTATAAATGCTGTGAAACAAAA**[G/T]**AAATTAAAAATCGCCATAATCAAATTTTTCATTCAAACAACTAAAATTATTGAGAAGAAGAAAATTTCTTTATTCGTGATATTGTTAAACGTCAAGGATT

>scaffold38.1.2720446

ATATAGATACAATAGCTTACGAAAGTATTTGATTTATTTTTTAATGAAATGTTAATAAACAATTTTTTATTTTTAATGAAACTTTAAATATGTGAATAAA**[G/T]**CATAATGAATTGGAAATATGGAACTTCTTTTTCTTTTTACTAAAATATTTTAAGGAAGTAAAGTTAAGAAAGTTTTAAAGAGGTTTTTATAAAAATTAGA

>scaffold38.1.2766186

ATAGGTATATGTAATTATATTTTTCATAATTTTTCATTACTTAATAATTAATGGAGTATATTTAGTATATTTTAACATGAAACTATAATAAAACAAGGAT**[G/A]**CTTTTACAACATACGTGTTTGAATTGTAGTTATATTTAAATTTTTGGCCAAAACTTATATGCTCGTCCATTATATTCATATAATTTACTTTCTTATGACT

>scaffold38.1.2799336

ATTGTTTTTATTTAATAGCACAATTATCATAATTATATATTATAATTACTTATATTTTTTAATTTCTAATTAAAACTTTAATTAATCTATAAAATTAATC**[G/A]**TAAATTATTAAATAATAAAATTAAACGTCGATCAGTAGTTAATCATTATAATCATTAATAAAAAGAATAATTATATGAAAATTTTTGAAATAAATAATCA

>scaffold38.1.2801219

TAATCCATTCTTCCAAAGTGCTCTTTAGATTGCATTTTATGAATATAATCGACTATAAGTCCTCTTAAAACTAATTATATGTTCAAAGCATTTATGTGCT**[G/A]**TTTAATAAAAATGCCAAATTTCTATCTAGAAATTAACATGTCATAAATTTAATATAAAAAGAATAATATCAAGTATTACAAATACAACAAATATTACAAC

>scaffold38.1.2832212

ACAATATAATTAAAATATTGATAAAAAATTTACTATTTATAAAAAAAATTACATATATTATCAAAAATATAACTCACATGACATGACAAAAACTAAATTA**[G/C]**TAGTTTTATCATAATTTCCATTTAAATAAACATTCTTAATATTTTTGTTAGTTTAATACAATATTAATGATGTAAATAATAAAGAACAAGCACATAAAAT

>scaffold38.1.1859150

GGAAAAATAATAAAAATAAGTTTATTTTTTTTATCTACAGTTATCTATATTCACATATTCAAAGAAAATCAGTTATACTATATAATTAAAGGATATCAAA**[T/G]**CATAAATAATTCATATTTCTTGAAAATGATTTTCATAATTTAAGAATCTCTTTATTATTTGAATATTTAGTTAACAAATGAATGAAATCACTTTTAAACT

>scaffold39.1.1807009

AATAGTACGTATTTCAGATTTCCAATTTACGTACGAGAATCAATTGAGAAAAATTTGTAATATTTTTATACATTCGTAAGAAAATAATTTTAATAAAATT**[A/G]**TTGAATTATCAAAATATATCGCAAAAATATTCTACTATTTTATTTCCAAATTATTATCCTATTATTCAAAAACTTAATTTATAAATATAAATTTCTTAAA

>scaffold39.1.2112306

ATAAAATCTAATTAAAAATAACGCGATATCGTTTTGAAATTTCACTATTCGGCTTCGACTGGAAAATACGAAAAAGTTGGATTTCTTTAAATTTTTCTCC**[A/G]**TTTTCTATTCGATATTTTCATCTATTTATTTTTTTCTTTTCGATTTCTGTTTATTATTCTTCCACTTCCTTCTTCTCTACCTTCCTCTCCCTTTCAACGT

>scaffold39.1.269954

GAATGATTGAATAATTGTGTATTCCAAAAATATTTTTAGAAGATGTAATCTTTTGATACTCGATCATTTATTTAAAAAAAAACTGATTATAGTATTCTCC**[A/G]**TTGTAATCAATTGGTAAGAAAAAAACAACTTTTTCTCAAATAATCTGTAAAAAGTTTGCTGAAACATGAACGATATTAAACAAGAAACTTTATGAAAAGT

>scaffold39.1.393445

GAGACGCTCGGCAACATTCCAACGGGAAAGTTGAAATCGACGTTTTACTTCGAATTTAATCAATCTTGATTTAATCGATTTTTGATCGATCCATCTTCAT**[A/C]**GTATTTTTTATTACTTCAATAAAGATGTATTTAGAATTTTATATTTGAAATTTTCTTATTAAAATATATTATCAAAGATGATGGAGAATTTGTTAGTTTA

>scaffold39.1.1181389

GAAATATTTCGTTTAGATTTCCATGATTCTAACTAACTATTTTTCATTTTTCTACGATAATTCTCGTTTCCACGAATAAGAAAACTCTCGAAGAAAAATA**[C/G]**TTGGAGAAAAAAATGTTTAGCATAAAATTGGACACAGATTTATAAATTCACTCATCATCGTAATAATAATTCTTTCGTATAACAATAAATTCCTGAAAGA

>scaffold39.1.1377323

ATTGGCACGTATGTAGTTTAATCTTTCTTCGATCCAAGCAGATTTTATGAAATAGAAATGATCGTATTAAATAAAAGATATAAAATTTCAAAACATATTT**[C/A]**TTTAATTATATTTATCTTGTCTTTTTCAAGTTCACGAATATTTTACGATCTCAAACTAAAGAATAATAAATTCGTTCCTTTCTCTGATTTTATAACAATA

>scaffold39.1.1696448

CCATTATTTTCCCCCCCATCATTTTAGACTTTCTAAAAAAAATCCTAACGATCCTTATTTCGATTCCAATCAGATATTTATCCGATCTGATCAAGTTAAA**[C/T]**GAAATAAAAAACGTTCAATACGACTTCAAAAATCGGAAACAGTCCCCCTCCCCCTGTTTCTTGAACAGGATGACGAATATTGATTTTTGGCGAGTACAAA

>scaffold39.1.1733136

GCCCTCTATTAGCATGGTTAGGCGATATATTTATTTTAAAAAGTAATTTCATTAAAGTGATATTTCATTGATGAAAATAAAACAAAATTATTGGTTTTGA**[C/T]**AATAATTTATTTGAAACGTCTTGCGTGTTATATTAAATATAGGAAGAATATTGTAATGTGTTCGAAGTGTTTAATTTGATGGTTTGAATATTGAATTTAG

>scaffold39.1.1826056

TAATAACGGAGAGAATGTATTTCGTCCTTCTTTTCACATTCAATCCCCGAAGTTCTGAAATTTCTAAAAAAATTTAAATCCACGCGAATATCCGAGCAAG**[C/T]**ATAGCGAAGTTTAATACGAAACAAAGCTTCGATGAGAGAGAGAAAGAGAGAGAGAAAAAGATTGAAATTCCGCGCGAATATTCTTAAACCGTGTATCGTA

>scaffold39.1.1841158

GTGAATCTATAGTTATGATGAATGTAAATAATAATTTATTTAATATAAAAATTGTGAAAACACAACATAAATTTACGTTTTAAGTAATTTTATTAAATAA**[C/T]**AAAATATGGGTCGTCGTTCAATAAATACCACAAAAAGTGGGAAATATATGAATCCCACTGATCAAGCACGTTAGTAAATAACATAACCTAAAAATTTATT

>scaffold39.1.1862219

TCTCAGGACACATTATATACACAACCAATTTCTGAACATCAGGTAAGGTTGTTAGAAACTAGTTGCTTGCTAGCATTTCATATTTTTCTTTTATCTGTGG**[C/T]**ATTGAATAGAATTTCAATTCATTTTTTGAGAAATATATTTTTGTTATGTTTTTGAAGTTAAATTATATTGTCATAGGTATCAAATGTGTCTTCTCAAACT

>scaffold39.1.2113440

GGTGCATCGGGGCCCTTGTTACCTGATAATTGAATAATCAGATGCTCGAATCGTGATAATTCGATTCCGATGCACTCGATTTCTTGGATGCACAAGAATG**[C/T]**AAAGCGTTCTTTGTATCCAATTTTGAGTATATTTTAATTATATTATTTAAAAAATATTGGAAATAATTATTGAGATGTATTTATCAATTTATTTTAATTA

>scaffold39.1.2177974

AAAAAAAAGATATTGTATATCATCGAAACAAATTGTTTCGCAAATTGGATAAAAAATGAACAGATTTATTGCAGATTTTCCCATTCATTATCGTTTCGTG**[C/T]**AATGATTTTATGAATTGATATTATTTTTTATAAAATGTATCGATATATTTTTCTCGTAGAGGCAATAACGAATCGATACAAAATGACAATTCAATAATAC

>scaffold39.1.2237076

ATAATATCGAACGTAAGTATAGAAAAAGAGAAACTAGTCATGTGAAGAACTTTTTTTATTCTTTATATTTTTTTTTAGAATGTGTTAAGGATTTACAAGA**[C/T]**AATCGTGATTTACAAGACAAACGGTTCTAACAAAAAACAAATTGTTGATTTAGATACATATTGTTTGTTCGAGTTCAACCGCGGTTCGTACCCAACGCTA

>scaffold39.1.2367479

ATCTCAAGATTGATAACGTGTTCGTTAATAATTAAAATAAAGAACCTACGTTGCTTTATAATACTAACCGTCAACGCTCAATTGGACCGAGCGAAGACGA**[C/T]**ACCGTCGCGACGCTATCTAAGAAAAATTCAAACGCGACTACTTGTAATATTCTACAATTAATATAGATAGTAATATGTAATACATAATAGAATACAATAG

>scaffold39.1.301184

AATATTAATATTATTATGTATTTATTTATATATATATATACATATTTAACGTTTAATTAGAAAGAAAGATAAATATGTATGAAGCAATAAAAATTTAATT**[C/A]**GCAAAGTTAAATTTGTGAATCTACTTTGAAACTCGATGTTTTAAAAAAAAATAGGATTTGAATTGAAACAGATTTTCGATATAATTATAAATAAACAAGA

>scaffold39.1.451739

CAAATAACATTGCTGGTCAACCTGGAAGACAACCACTAGTATGTTATTGTTAAATAAACTCATATTTTTTAATTAAATATTTATTTATTTATTTATTAAA**[C/T]**GAATAAAACCTATTAATAAAATATTTACATGTATTAAATTTAATATTTATATATAATTAAAATAGAATGAAATTAAACAAAATAGAATAAAAAATAGAAT

>scaffold39.1.896051

TTTCTCCAATTTGCTAATTTCAATTAATGATTGAAATTCTTTCACATACCGATACTTACACTCTTGGTATAAGTGTATAAGAATTGTAAATACGATTTTT**[C/G]**TTTTTACTAGATTTTAATCACGTGATTTTATTTACACACGAAGCTTTGTTCGAAAATGAATTAGTCTTATACATTTTAAGCTTACCAATCATTTCAAAGT

>scaffold39.1.1319476

CTAAATACAAGTGCAACGCTCGAATGAATAATTGATGGTGATAATTTGAAATAATAATATACGATGATGATGTTGTACGTATCCATGAATATTTATAATC**[G/A]**TTTTGTAGAATTTTAATTTAATTTGTAGAATAGTACGTGTTACGAAAATGAAAATTTATGTCATCGGTAGAAAGATATCTGAACGGTTAACTAATTTTAA

>scaffold39.1.1343995

TCAAATGTAATATACCTAACGTTTCAAGAGCAATATTCAAAAAAAGTTTCATGAAGGAATTGCGCAATAATATAATATTCCAAAAAACGATTATGATTGA**[G/T]**CATATATTTATATAATACATAAGAAGAAGAGTTAAAACGTTATGAGAAAATTATATAGTTTCTATTGTAAAGAATTTTTAATTAAGAAATCATATAATCT

>scaffold39.1.1355167

AAATGGTTATATAATTAATTAGTTACTTAAAAATTATATTATTCATTCGATTTTTTTATCCGATAAGTATTCTGAGAAACAAGCGCAAAAAGATTATCTC**[G/A]**TACTCCTAATTTTCTTCTAGAAAATTATAAATATTTCCCGATACGTAATAATAAGCATTGCAATATTTTTCCCCCTCCCCTCCAAAAAATCTCACAAGTT

>scaffold39.1.146366

CAATTATTTTGTTCAATTTGCATATTACTCTGTTTATGCCCAACTTTGGCCAATATCGAAGGTAATCTTCGTTCTATATAACGTCTTCATCGGCTAAGAC**[G/A]**TTTTCCTAGTGTAGTATCATAATGAAAAGCAATTCTTTTACTACCACGATCATGATATTTCTACGAAAATAAAATTTTTAATTTTAAATACAGTAATTTC

>scaffold39.1.1485764

ATGAAATCGAGCCGTGTCTCTTGAGAAGATACTTGAAAGATACGACAAGTATGCACTGTTTCGTATTACAATCTTATTTACCATCATACAAATTTAAATA**[G/C]**TATAATTCGGAATTAAATTATTTCGCAATTTCACTTCTCTCCATTATTATTTCAATCATTTTTCTTATTCTCCATCGAAACGTTATCACTATAAACTTCC

>scaffold39.1.1508136

CAAATTAATTTAAAAAGTCACCTGTTAAATTGAAATTAGGTTTCTATCCGATTGACTGGTTCGTTTTTTAATTATCCCATAATTTTTCTAATCATCCGGA**[G/C]**TTTTGTTTCACGCGCATTAAAATATCCAAAACACATGTAACGCGAAAAATATATATTCGCATGGAAATCCACAGTCTAGTTATAACCTTTCGTTCAACAG

>scaffold39.1.1558439

CCCCCTTGTCAATTTGAACGAACGAAAAGAAATCCATTCTCACTTTTCACTTTCCTTCCGATCTGACGAGAGATAATAACTTCGACTAAATATTTCTATT**[G/C]**AATAGAATACACACGTGAGTGCATGAATATGTTTTGAAAGTTATAATAAATGGATATAATTTTATCCACATTTTTCTGCATGAAACAAAATTCAATTGTA

>scaffold39.1.158459

ATAATAATAATCAATATTCACGTATCTACATATAATTTAAATGTTTCAACTTTAAGAGGATTATTTTACAATTTATTTTACAAAAATTTCAACGACGCAT**[G/C]**ATTTTCCCACTACATTCGAATTAAGAGAAATCGAATTCACCAAGTTTTGACCAAGTTCTTCGTTTCAACTTAAACTCGTCCGATTCCGTCGCGGCTTGAT

>scaffold39.1.1606909

TCTTCAACCATTCGAGATCAACCAAAGCGCAGAAAGTATATAAAGAAAAATAAAGTGAAATTCCAGAAGGGAAAACGTCAATTTCTTTCAACGTTTTTTC**[G/A]**TGATGTAAGGATTTGAATATCACGTGACTAACCTATGGAATTTCCCTTTGCACACACAGGGTGAAGAGATTAGGTTTCTTCGGATGGATGGGCCGGCTGG

>scaffold39.1.1617617

AATATTAACTCTCTAACTTTAAGTGGAAATGATATCCTGTATAGAAAAAGAAAAAGGAAGACGATTCTCATGAATTATGTTAAACACGAGTAAAGATAAT**[G/A]**CTGCTTGAGGATTAAAGGAAGGGAGATTTGATTAATTCAAATCGGTGTCGAATAATTATTTATGGTGACAGAGAAAAGAGGAAAAGAAACTGTCGCTCTC

>scaffold39.1.1679058

TCATTGCGATTTACGCACACAGTGGTTGGGTTAAAATTATCGAAAGCCATGTATTCTCTTTGAGAATACGAAGGATAGAATGTTTTTCGATTGGAATCTT**[G/C]**TTTTTTAATGACAAATAGAAAGTAGTTAGATGTATGATATAATTTAAAAAGTACACGGAATAAATTAAGAAAATTTGGGGAAAATATTAATAATTTAGGG

>scaffold39.1.1959475

CCCCTCTTTGGTCGTTCTATTAGAAAATACTTTGCGCTCTCGATAGGAACATTTTTCTTTTTCTTCCCCCCTGCGTGCATGCTCTCCCCCTCCCCTATCT**[G/A]**TCAGTCAAACAATGAGAAACAAGATCGACGCTATATCTTTTCTTGCATTGTACGTATGTACGTGTATATAGGAGAAACAACGTTGGATGACGAAATGAAA

>scaffold39.1.219012

TTTCCATGTTGTAATAACGTGTCGTTACTCGTTCGAGATCCATAATTATAATCACAAGTATCGGATAAAGATCGTATCGATACGATGAAATATTTAATTC**[G/A]**TAATGTGATTGAACGTTAAGTTAAAGACGAAACGAAGAAACTCGATAACAGTTATCCCCTTGAAATTGGTCTCGTTTCGAGAGAATCTCTGTTTCCGTAA

>scaffold39.1.2203506

GTAACAGAAAGAATTTCGATTAATAATATTTTTTAATGATGGAAGAAATGATGGCTGACTAATTATAAATATTTATTAATATAATTATTAATATTTATTC**[G/A]**TATGTAAAATAAATTGCTCGTAAAGAATTTATAAAAAGATTAATGAAAATGATGATTTTTTAAACGGTTTAACTTTATAAATGGTGAGCTCAAACTCGTC

>scaffold39.1.2226089

TAATGATTAAATATTACATTCGTTTATGTGTATTCGTTGGAAAATATTTAATTTGAGATATATAATTAAAATATCTGGAAAAATTGAATAATAATAAATC**[G/T]**AAAAAGAATTGCATTTCATATTGCAAAATATACAAAGTATATTCTCAGCTATTTTCAAATGTCTTTATCTCCTTTTATGAATCTTCTCAATATTATCTCA

>scaffold39.1.2289986

GTGATTTTTCTGCTCTGATTTCTCTTAAAAATCATAAAAAGGATTTAAATTACTATCTAATTTTCAGATATAAAAATATTTTCCAATTTCAAATTCATTC**[G/A]**TCTCTCATTCATCTGAAATTCTTCTCGATCATTTCGTCACTTCTTCACAATTTCGATTCTTCCTAACGACCTATTCGAAAAGATTCATCTCGTACTTCGA

>scaffold39.1.2330980

TAATCAATGTAAATTATTATTTCTTATAAAAAAAATTTTCTTTTCATACTTTCTTTGTCTTTCATACGATAAATTTCAGAAAATTCAAATTAAAATATCT**[G/A]**CCAGAGTAATAATTTTTCATTATAAATGATTTAATAGACTTTTAGAGAACAACAATTTGTATCGATTAATGCATAACATTTAAAAAAAAAAAAACCTCCC

>scaffold39.1.244617

AACCAATCGATATTTATCGAAGCCAGGATGAGATAATTATTTTTTGTTATTCTTCTTCTAACAAAGAGAAAATAAATAAAATCACACAGCTTTCGATCAA**[G/T]**CGTGTAACAAGATGTTGTTAAAAAAATTTGTGTTATCAAATTGAATTATAATTTTCGTAAATTTGATAAAGCGTTATATAATTGTAATTCCCATTGAACG

>scaffold39.1.280167

TTCTTCTTAGCCGGGTGGAACCTGACAACCGCCACAATTTTATTATTCTCAACGTCCTCGCACCTTTTGTCTATATCTATATACATCTTCCTTAGGAGCC**[G/A]**CCATATATCGGCGATCGAGACGTTTAACGTTGGTTAGAAGTTCGATAATTGGTCAACAAGGAAGATTTCTATGCATTTCGTTGCGCTTTTAAGATAGTAA

>scaffold39.1.386152

GACAAAGAATCGCAATTAGCCCAAAATTTCGAATACGAAAACATATGTGTAATATATAATACTCCTTATTTCTTCGATTCGAATAGCCCATAAAGGCTAC**[G/A]**TAAATCCGCGAAAAAATATATAACTCATTATTAATTGAAAATCCAGCTCAAAGACAATTGTGTATCCAATGTAAGAAATTGCTGATACTAAATTAACAAT

>scaffold39.1.390226

CAATGGAACATGATCAATCGCGAAATGTACAGGAAATACTAGGCAGCTCGGTGTTGTGAAAAAAGAAGAAAAGTAATGCGTGAATACGATTCATGTGAAA**[G/T]**AATTAATATTTGAAAAACGAATATTGAACTTTTGGTAAATTAATTATATTGAATTAACGAGGAAAGTAATTGTGACAGTTAATTACGTGGAAAAACGAAT

>scaffold39.1.515197

TTACCCTTCTTGTCCCTCCCATTTGTTCCCTTTCTGTATATCGTATTTTTTTGCGATTCATCATATTCTCTACCACTTGTTTTTCTGTTAATATTAATAA**[G/C]**AAGATACTTTTATTCAAGAGTTTTTTTTTCTAAACAAAAACTTCTTTATCTTGTTATAAATTTCATTGATAAAATTCATTAAATTGCCATTATAAAAATT

>scaffold39.1.818822

AATGCTCCAATAGATATCTCGAAGGAACTGTTCAGTATTCGAAAAAATATTTCGAATAGAATTTATTTTGTTCCGAAGAGAGATGGTCTCATAAGAGAAC**[G/T]**ATAATATTTCCTTTATTTTTTAAGTTTTATTTCAATTAATAAAAGAATATTATAATAATATTTTTTATGTATTCAATTGCGAAAACAAATAATTTAAAAT

>scaffold39.1.1112018

TGTGAATCTCTCCTTTAAAATTTCTTCTTTTCATCCTGTAATCGATGCTTCGTATTTCGAAACAAAGGAAGGGAATTTTAAATGATAATATTAGAAATTG**[T/C]**ATTATTTTCATTTAGTTGGTTGGAAAAAATAATAATAAACTCTTTTTTTATAAAAGAAAAAATTGTAGTTTCTCCGAAGAAGTATAGATTACGATAAAAT

>scaffold39.1.1743854

CTTATTTTACATGTTGCAAAACACGTTAAAATTAAACGGAATCATATTCACGAATCAGAATGCTTCGTGGTTCGTCATGCGCGACACTTGAACGGAACTG**[T/C]**AACTAACTGTAACTCACAGTAATAAATTACAATATTCGATTAAAGATTGAGTGGTCAATCGTTATCTTCAAGTACCACGATCTCTCGCTCCGGTGAAGAT

>scaffold39.1.1836406

CAAATATTACATTTTCGTTATGTTTTTGCCAGTTAGAAGAATTATTTATCGGAAAATTTAATGTAAAAATATGTATAACTTTAGGTTTAGATAAATTATA**[T/C]**GATATAGTAGTTATATTCTCTTTTTTATCTTCTTTAATAAATTGAAATTCATCTATTAAATTAGTACTTGGCACAAATAGAGAACTAACTTTTATTTTAT

>scaffold39.1.237935

TAAAAAGAAAAGATAATTTTGCATATATTATTACGATTAATTTTTATAATGGCACGTCAGCATTTTTTTTTCTTATTAACGAAAATAAAACTTTGCAAAG**[T/C]**AAAAATTAGTTGCTTGAAATTAATTCAACTAATGTTATATATTTTTGGGATAAAATATCTTTTATAAAAGTTATTCAATCTCTTAATTGGGACTTAATTC

>scaffold39.1.685938

AAAAAATAAAATTAATTAATTTTTCGAATAAATAATTAACATAGCATGAAATTTATTTAAATAATAATTAATTAAGCATGATTTACAGGAAAAAGGAATG**[T/C]**AATTTCTGAAAATATTTTAATTTACATTATAATGCATTTAATAGTAACAAAATTCAAAAAACAAATATCAACGTTAATGTTTCCTTTTTCTTTTCGTTTA

>scaffold39.1.761493

GAAGCCTCTCGTCGCGCAGATTATATTTTACAACCGATTTATTCAATTTTCTTCTTCATGTCCAATTGCACTTTTACCGATCTTCCGTTTTGAAAAATCA**[T/C]**GGCCGCTGGTCGAACAGGTAATCAAATTATTTTCCTTCCGTCCTGTTGTCTCTATATTTTCTTAAATTGTTTGCTTATTATAATCAATTCAAGCTAATAT

>scaffold39.1.926972

AAACATTTTAAAAATGTCAAATATTATTAACCAAATATAAAGAGAGAAAAATTGCTCATCGATGATATAAATAACGAATAAGAAACATGTATTATTCGAA**[T/C]**GATAGTTTAAATCAAAACAGTGCAAATATCTTAATTCGAAATAAATTGTATGAAAACATATTAATTTGTCAGCAAGAAATAAATTACTATTATTCCTTAC

>scaffold40.1.23035

AATAGCAAGAAGATTGTAGGAAAGGCTTGGCTTTTGATTTCAATATGAATTGCAATTTATTAATGTATTATAATGGAAATGAAAAATTGTTTTGTACGTA**[C/T]**GATGTACGAATTTTGTAAATTCATGTTAAGTGTTTCGATTAGTGTTGCGAAGATATTAAGAAAAATAAGTGTAAAACGAAGCTTGAGAAACACTGGATTT

>scaffold40.1.121676

AAGGAATGTATGTGTGTCAGGTAACGAAACGATAATTATCTAACGTAAATTAACCATAACAGAACGAAAGATTTAATTATAAGACTCGGATCAATGAAAC**[G/A]**TGCGTGGCAGGATGTGCGGAAGAAAGTTAATTTCGTTCGACAAGTTTGAGAAAGAAACAAGCAAACGCGGATTTTACATAACACTTGCATCGAATCTATC

>scaffold43.1.297870

ATTATTTAATACATTTAATAATAAAAGAGATTAACTATAATGTCAGATATTAAATATCGATAAGATAGCTAAAAATCTAATTAATAAAAAGAAAATATCT**[A/G]**CTTTATACCATTAAAAGGAATTCATTAGAAATGTTTCTTCTTCATTTTCTTTACTATTCCTGACCTTGCAAAACACAAACCAAAAGAAAGTTTTTATTCG

>scaffold43.1.455047

ATGAATTATATCTAATAATTTTTACAAATTTTACAAATTTTTAATAGATATTAATAATTTATTTTAAATCAGCAATAATTAAAAATATTGTTAAATTATT**[A/G]**CATTATCATTATTCATTGAAAAATAAAAGTATTACAAATACTAAAAGTTCTGTGACTTTAGATTAATTTTCTACATAACGGATTTCTACAGAAGTCAGAA

>scaffold43.1.48837

ATTTCTAATTATACAGGTGAATAAATGAACGTATGTAATTCCTATTTCTTGCACAATATATATTATACATTATTATAAGTTCTTTTCTCGATCCGATGAA**[C/T]**GCTTTATCAGATATCTCACAGAAAAAATCAATTATCAAATATTTTTTCTTCGTCATATTTCGCCAAATATATTATTATATATAATTATATTAATTATTAT

>scaffold43.1.53637

TCATTTTTTGATTATCTTAATTATTATATTGTTAGGTAACCTGTTCTCCGGGATATGTAACGCCTTTGGAACCTGTAAGAAAGCCGACCTGTCGGGAAAA**[C/T]**GGAATATGGAGTGCACCCCCACCACCTTGTAGATCATATAAAGATGTTTAAAGAGGAAAAAAAATTGAACTTTAAGGAAAACTTATCGATGATAGCATTT

>scaffold43.1.636552

ATTGAAAAATCGCGTTTTACATTAGTGAAAAAAAATGAAAAAAAGAAAACAATCTGCAACAAATATCATTCTTTCATACTTCATATTTTCGATGCATAGA**[C/T]**AAATAAGTCTTTAAATGTCGCAAGAACGTGATTCCATCGCAAAAATTTTCTAAACAATAAAATAATTAACATTTCTATATGCCATAGCTAAATAGCATTA

>scaffold43.1.355990

TAATATAATTAAAATTAAATAATGTATTGTAATTTGATATGATCTTAAATAAATAATATAACGGAGGTTTTATTGCAATCACAATTTGATTATTTTATTT**[G/A]**CAACGACTAACGCAACATATAAATTTTCATCGAATTTCAAAACAATCTTCATTCCGCTTTGACAGCGAACTTTTCCTAGACACGAGTCTATTGAAAATGT

>scaffold43.1.819045

TTAAATTTAGATTTCATGAGCTTTTATCATTGAATAATATTTTTTCTTCGAGAATCGAATAAGACGGCGACAGGATTAGAAAAAAATGCAATATTTCTAA**[G/C]**TCGATGAACTTTTTCCTATCGATGAGAGATGGAAAATATCGGAATACAATTGGTTTTATTCGTAGAAACGAATCATCAGTGCCACGTAAGGTTCCATAAT

>scaffold44.1.158412

GTTGCCCCATCTCGACTTGTAATTACTCTTTAAATCTTTAAAATTCTTAAAATATCTATCTTTAGATATTTTTGGATTGGACACTTGAAAGTAATCTTTA**[C/T]**GGATTAATTTTTTTTTTTATTAGCATATATAATACTTACAGAACTTTGTACGAATAAAATTATCTTTGTTCAAATCGATAATTCGTAATCTATCCATCAT

>scaffold44.1.281451

GGAACGAGATATCTATGAACGTTGATTCAAAGTAAGTGTGTCGATACGATGCCAATAAAGATTTGGATTCTTTTTTTTATCGTAATTAAAATCATTCGTG**[C/A]**GGAATATTCAAATAGAGAAATTATATTTATTAAACCTATTAAGACACGGTATTATTAAGTTACAAAGAATTGCGTAAAGTTTGCTTCGTTATTCTTCAAC

>scaffold44.1.60480

ACCTCGAACCAAACGAAATAAACTAAATTTCCATAGAGGTAAACCGTTATGTTGAAATGATTATCAAATTTTGGATTCACACAATTCGAAATATCAAGCA**[C/T]**GTATAGGTTTACATGAGAAATAAAAATATAAATTCGTAAATTTGTAAATTTCTTTCGATACTCGAGATTATACAAGTGTCTTCCTTTCAATCAATCATGT

>scaffold44.1.154116

TAATCAAGATTAAACCTGAGATCGAGAAGATTTTTCGTATGATTTTTTTAAAAGAGCTACAGAGAATTTCGTTATAAAGTAACGATTTTTCAAGTAAACT**[G/A]**CTTATTGTATCAACAATTCCATGTTGTATAATTTAAAATTCTTGATTAATTTGATTAATAAAGTGATAAGACGATATATAAAAATCTAAAAAATTTGGAT

>scaffold44.1.297203

GAATGTATCTATGACGAATTAGTTATTATCTCGAGCAAAAGACAGCCTTCAAAAGATCGTGGAATGCGGTTTTCTTTCTAAATAAGAAATTCATGGATTC**[G/A]**CTCCCTCGCTGTTATTTATCTTGCTTTATAGTTGAAAATTTTGAAATGTGATAAGTAAATAATTACGTTGAAATATTATTTTTCAATAAGGATTAATTTA

>scaffold44.1.80770

TTTATCGAACGATACACGATTTGATCGATCGAAAGAAAGGAAACTGATCCTGCAAGAATTTTCCGAATTGTTAGAATATATATTATCACAATTTCAAGAA**[T/C]**GAAACGTACAGAATAGATATGGAAAACGCATAAAACACAAGCTAAGACCCAAATTAATTATATAACTTCCTCGATTGGTGGTTCAATAAATCCGAGTAAA

>scaffold45.1.1135895

CCTGAACGACAGGGGTGAGCAAGAATTAAGGCTAGCCCGTGGCCATGGAAAAATCGACTCAGCACTAATTTTTTATATTTTCTGGTAAACAATTGGTTTC**[A/G]**TTTTGTATCCTTATTTAACATGTATTTATAGATAATTGTGAAATTATTTTATAAAAATAATATGGAATGAAAATGAAGGATTGAAAAAAACTCTTATTGA

>scaffold45.1.11523

TTTGAAATTTAAATTTGATTCTGTCCTTTTGTGGATTATTAAAGGGGGAGGGAAAAAAATGTATAAAGCACGTGTAATGAAAATGTTATAAATTCTCGTT**[A/C]**TGATTAAATCTGAATAATCACGTTATTTCATGATTACACGGCATAAAGTTAAAATCTTGAAAAAGAAAAAAAAAATATACAACCGATTGATCAAAACAAA

>scaffold45.1.1397587

TAATGCGGGCGCCGATCGTTGTTTTACTGCAGACAATGAATTTCAAATCAATTTATGCAACTACGATCGCAACCTTCGTGTAACTCATTGACGCCTACCC**[A/G]**TAAAACCATCCACCACGTTTATCATCCTAATTACGCTAAGCGTAAATTTATTCCCGCGAATAATATTTATCATATCCTCCCTCAGAATTAACGTCATCTC

>scaffold45.1.1519363

GGAAGAAAGGGATGGCAAATTCCTTGGGAAATATTCGAGTTACAGGTGAAAGAAAAGTCGTTAAAGAATCGCTAAAGAAGATTTTTCTGTTCTTGATTCC**[A/G]**TTCTTCGTAATCTATGTATATATAATTTAATCAATAGGCACGCGATATCTCCTTAAATGTTTCTTTCTAACAAAATTCTTTGAATTATCACGCAGCATTT

>scaffold45.1.1758007

TCATCTTTCATTCATTCATTGCAGCATTCTACTAATGATCTATGATGCAATGAATACATTTCAAGTAACAAATATGGCATAATTAATGATTTTATTGTGC**[A/G]**TTTCATTATAACGTTCATATTTCAATGTTTCAAAAATTAATTTAGAATACAAAATTAATATTTATCAATTTAGCTCACGACCCTCAATTTTTTAAAATTC

>scaffold45.1.203027

GAGGCAAGAAAAAAAAAGGTAAGGATAAAATACACGTGTGCATCACAATGATACAATTGACCCTCGTCAATTTTTGTTTCAGAAAAATATGATTCGATCC**[A/G]**TAATCGATCTACAAAAATAAAAAAAACGATTAAGCATACCATGTAATGTCAGGGACAGGATCGGCAGTGAACATAGCTTTGAACATTCCAGATTTCCCAT

>scaffold45.1.3372

CTGTTTCTCGTTCCAATATTTCTTTTCCAACAACGAGTCAGAATAAAAATAATATTACCAATTATTCCTAAAATCTTTCTAACACAAATACTTTTAAATT**[A/G]**TTCTATTTTAGATATATAAATTACAGGATTACATTATAAATTTCGTTTTTTACAATTCTATCGACGGAAACGTATATCAACTGAAATATGAAACTCGAGG

>scaffold45.1.656728

CAAGAGGGAGTTGATTCAATTCCCAAACTTTAATAATTTAAACGTCCATTATTAAATATAATTTATATGATTTATAGTATTTAGAGTAAATTAATTGCTC**[A/G]**TACATTTTAAATATTTCTTGCATATCACGTGTATACGATTTTTCTACACAATTTAAAATTTAAAAGCTAGTAATCTGAATCTTAAATTTAAGGTTACTCG

>scaffold45.1.752023

ATGTATGAATCTCTTCATAAGCTTTTATCATCTGTAAATTTATCATAACATTTAGAGAAGGAACAGATAAAAAATGAAGAGATACAAAGATATCATTGTT**[A/G]**TTGCTACTTAATAAAATAACTCGTGCTTGGAAAACATTATTAATGCAGGATGTGCATTATCGTTTCCACTCTGTTTCTTGCTTATACTTCGTTACGAAAA

>scaffold45.1.900946

TAATTTAATCGGGGGATCGATTATATGGAAGGTGGCAAGGTAATCGAAATTGCGAAAGTGTTTACGATTCGAAGATTTTCGAGTAAAAGGCACTATTTTT**[A/G]**TTTCTTTCTCGATCGAATTTTGTTGACATTTGTCGGCAACTTCGTAGAAATAATTAATGATTTTTTTGTACAAATGTAATCATCTCACAATAGTTTCAGA

>scaffold45.1.913845

GACCATGGAGCAAGGTAATGCATATATGCATTTTGAAATAAAATTCAAGTTTCTGTGGAATTCTTTCTCCGTTCGATGATTTTGAATGATTTCAAAGATC**[A/G]**TTTAGAGAATAAAATAAAAATAAAAATAAAATTCATTGAACGAAACTGATAAATCCTGCAATCTCTTAGTTATAAATAAAGTAAAAGATAAAAGATTTTA

>scaffold45.1.1164379

CTTATTGCTTATTCCATTCTTCGTTTTGATCGACGTATCGATAAAACTCGAAGTAAAAAGCGACAGAAACAAATTTCCCTTATAAAAAATTCCAAAGTAA**[C/G]**TACTTATCGATCGTTCGCAGCTAAGAAAATCGAGCCTTTTCGATATTTTTGTTTCGAGATTTTTTTTTCACTCATTGAAAAATATTGCAATCAGTATTTT

>scaffold45.1.1190745

AAATTTTATTACACATTGTTGAGACACACATTTGATCGAAAACGTTGAACAAAAATTCGTTTACTATTATCGATACATTTTTGTCACAACAACGTTCCTT**[C/A]**TTATTTATCAATAATTGCATCAAATTGGAAAATTATTTCATATACTTGTGAGAAAATCCATTCCTCTATTAATTATATAAATCATCCGCGCGAGAAATGG

>scaffold45.1.1295851

CATTGAATTTAAGATTAACGAAGCGATATATTCGTTATATTAATCAATTATAAATATATATATATAAGTTTAAACAAGTTCATTGAATAATTCAAAGTAA**[C/T]**GAATAATTTGTTCGTTTCTATTGATATTCCTATTAATTCACTATTTTTTTTAGAAGATATTCTCTCCGTTAATAAAAGTGTATATAGTTAAATAAACAGT

>scaffold45.1.1509115

GAGCCAAGTACGAGAACAAGTTCTTGGATTGATAACGCAAAGTTTCGTCAAAATCTGCGTGTTCCCTTCAGACCTGAAAAGCAGAGCACAATGTTAGAGG**[C/T]**AAAAATTAATATTTTGTCGAACGAAAAGAAAATTAGAAGACGTAAGAGAAGGATCGATGTAAAATTAAACGGCAGAGTTAAACGAAATTATTTAAATAAT

>scaffold45.1.1659052

TGACATAATGTTTTATAAATCGTCGAACGTTTAGAGATTGAAAATTGCACGCTTTTAATAATTGAACGAATTTTGAATAATGATAAATTTTCTATTCGAA**[C/T]**GGAACGTGTGACAATTCATTAACGTTTAATAAATTTTCAATATATCCAATGAATTTTCAATGAATTTAATGAATCAAAATTAAAAAGAAAATATATATTA

>scaffold45.1.1689749

CATATTTACATAATTCAGTATCTCCTTAGTTTGAATAAGAATCAGAATTCGTAGAAATAAATATATCTCGAAATGAAAAATATTTTAAGAATCGTTCGAG**[C/T]**AATTTTCAAAATTTTAATTTCAATCTAATTTATTCTTAAATATTTTCAATAAAATCCAAAAGATAAAATTGTCCACAAACATATTTACTCTTCTAGGAAC

>scaffold45.1.24291

CAATTATTGCATGAAGATTTTCTCGATTGATTATACGATGATAGATTGAAAGATTGACGTGAACCTTAAATACCAGAGAATTGCACCAAGAGAATTATGA**[C/T]**GCAAATGGTCAATGAATTTCCTTTCTCTTTGAATCGTCTTCTCATCAGGACTTAATTCATCTTTGTACTTGTGTTACCTACGGTGAACTGATTCCTTTCA

>scaffold45.1.267974

TTCCAGAGAAACGATCGTTTTATCTAATCTTGCTTGCTGATAAATATATACGTTAAAAATCGCGACTTTTCCTTCGAAATCTATGGTGAAGACCGTGCAA**[C/T]**GACGAAGTTCTTGGAGTAACTGCAGAATAATCACATTTCTCTTTACCAAAAAACGATTCCCCACTTCTTGGCTAAATTCTTTAAAAATTATTCACTTTTA

>scaffold45.1.321689

GTTCTATTATACGAATAGATTTAGTCTTCCCTTTGATGAGAAGGGTCCAAGATAGAATCATTATGCGATCTAAACAATCAATGATTACATGTACCCTTTG**[C/T]**ACCCCAAGTGCATTATTTATTTCATTGTATCATTTTTTTTCTTGATTAATCAATGTTTTTGAGCAAGAAATGCTTGTGTGCAAGAATGCAATCGTTACAG

>scaffold45.1.649470

TTACCTTAAGACGGCTTGCCCTGCTTTTAACGTATCTATTGCAATTTCGAGATATACGAGAGGTTACAATTTTAAGATTGTATTATGCACTTGGTTGTTG**[C/T]**AAATTTTTTTTTAAAAGTATGTACAATTTAAAGGAGAATCTCGTTGATTATAATTTTCAAAATTACTCGAAGGATGTTTCTTAAATGTTTTTTAAATGAA

>scaffold45.1.778936

AAAGCAGTCCTAATTCGGTTGCAACTTCTATACTTTCAGGATTCTCGCTAAAAGAACCAAAAAATAAGATAAATTTTTAAGAGTATTTCCAGAAATTTTA**[C/T]**GATTATCTATATTTTTAAAAATTTTGCGCCCAGTGAATTTTGATAAACAAATTTTTATTCGATTAGTATGTTTCAAATATATAAAAAAAAGTAACACATG

>scaffold45.1.933054

AGTTCTAGGGAATATATTGCAAAAGTAAGATGTCTTTCGATATTAATCATTTTTAAATTTATTATTTTTTTAATTTTATATTTTATATTTCTGAAAGAGA**[C/T]**AACATCAGAAAGTTTCTCTTGTTTTTACATAAAATAAAGTTAAGGTAGAGCATGGAACGTACCTCGCGTAGAATCAACAGAAATTTTTTCGAAATTTTTA

>scaffold45.1.1089705

TTACAATTTATAAAATAAGAAAAGCAGTAATGGAAAAAAAATAAAGGTAAAGCAAGAATTAATTATTAGCGCCATCTCTTTGATATCAAAAAATTTTTAT**[G/A]**CCATCTCTGAAAAATATACAAACTTGAAATATATTGAGCATTCAAAAGATGAATGCAAGTCATCAATTTTATATCTTTGTGCATAATTTATTTTTTTTTC

>scaffold45.1.1204651

CGGGACAACGCCATTGAAACTTACACGGCGCGGAAAGATTATCGGAGAATCGTTTGTTCGATCGCGATCCAAAGCGTTGAATATTAGATCTGGCGAAATT**[G/A]**TTCTTTGTTACTTGTGTACGTTTTATCGATGAATAGATCGAGATAATATCTTGTTTATTTTTGAAAGTTGGAGAAAAATGGAGTTAGAATAATAAAGTTC

>scaffold45.1.1231842

GAGAGTTATGATTTGATGCTTTCTTCCTAGAAAAAAAAAAGTCTCTCAAATCATATTTTATGTCCACGTTTCTATTCGCTATTCTATCTAATCTCATTCT**[G/A]**CTTAAAAACGGAAAAAAAAAAGTAGGATTGTTTTGGAAAAAAAAATTGTGCTCGTTTCTATGCAATCCGGACTTCTTCTAGCCTGATCTTCCGGATAAAA

>scaffold45.1.1296297

AATATCGTTACTAATCAATGAACGTAATTATTTTCATAAAAATAGTAATCAAAATATATATTTCCAGAGAACGAAGAATGAAAAGAAATTATCCCGTAAC**[G/A]**TCTATAATTTGAAAAGAAAGAAAAGTAATTAGTTCTATCGTCTTATTTTTACATATTTATATATAAATTTTTCAAAAACTATAACAGCAATAAAAATTTT

>scaffold45.1.1730949

CGATTGTTTTGTTTAGGTAGAGAAAAACCGAGTGAGAGAGATTTTCCAAGGCATCGCCGGAAGTGGTTGATCGAGGGTGTAAGAGAAACGAAGCTTTTCT**[G/A]**CGAAGATTGATATTAATGGGGCAGGGTTTGAGAGTTAGGTTTTGGTTAGATTCAATGAGATTGAAAAATCGAATTCTTCTTTCGTCTTTGTGTGATAATA

>scaffold45.1.343054

TCTCTATATATACACTTTTATTTATTCAATAATATAATCAATAAAATAATAATTTGTATACTCAAGAAAATAGGGTCGAATAATAGATCCCCTCCAAGGC**[G/A]**TCAAAGATCCAAAGGAGAATGCAGGGAACAAGTGTATTTCTTAGGATCGTCGAAGAATGGAGTCGCAACCTTTTAACATCACATAGTTTGCCTGGTTGTT

>scaffold45.1.707864

GTGATGCTTTATTATCGTTATATTGAATCGAACTGATCGGTTTCAAAATATCTCTGTTCGCAAGATCACGCGTTGTTAATTTTCTAATCGAATCTCTTTC**[G/A]**TCCAATTGAATACGACTGGGCAAAGCTAAAGATTCTTCTTGAATTTCCTCATACAGATCTCTTCTCAGACCTCGTGGTTGTTTCAAAGAATTCGATAAAG

>scaffold45.1.747609

GGTGAAATTTATGAATATCTCGAAAAATGAAAGAAAAATTGTAATAAAGAGGGAAGGATCTTTTTGTTAATTGGAAATATTGAAAATCAAATTTATCTGT**[G/A]**CCTAATTAATAAATAGTTGTCTGAATTAATTAATTGATTAATTTTATTAACTTTCTCAAAAAGTTCACGAACTTTTAACATAGTTGCAATCTCGTCTAAA

>scaffold45.1.863127

TATAAAAGTATGAAAAAGATGAACCCGACTCTTTGAAGAAAACTTATAAATAAAAAGTCTCTTACACTCAATTTTAAATATCAAATTATTTGCATAACAA**[G/C]**TTGAAAATTTAACTTTACGTATCCCTCTCCCCTGCGAACTAAATAACAAAATACTTCCTCGAAACTTCCTCGAAATTCATATCCTCGAAAATGAAATAAT

>scaffold45.1.1283446

TTTAAGTACCCTGAATTAATTAACCATTAAATCATTATAATTCATTTCCCAAGCAATTCTTACAAAATTGCATACACCTTCCTCCCTCCATAGATTCTAC**[T/G]**AAAATTTTTACAAAAATTTAAATATCTCGATTTATTTGTTAAAATCAATAGCCATTACTATCGTTCGATTTAATAAATTTGTAAATCCGAAAGGTTAGTT

>scaffold45.1.1284012

ACGGTCCTTGATCCACTCTCAAAGGAACTCTTTTTGAAGGCAGAAAATTAAAATTATTTTTTATTTTAATAAAGAATCGAATTCTATGGAAAAGAATTAA**[T/C]**GGGGAGAAGACGTTGATATTGTGAAATTATTGAAATTTTTTTTAATTAGACGAGAAACGAAGAACACGATTTTCGTGGGATTAATCTTCGTTTAAAGTTT

>scaffold45.1.1794524

GACCAAGTTTTGTCGTGCGATTGCTTTATAATTCTCTTCCTAGTTCAGAAGTTTTGAAATTTTCTTGGGAAAATTTTTCTGAATAATGATTACGACGATA**[T/C]**GAAAGATAGACAAGAGAAAGATAGATGTACAGAGTTATAAAAGAAAGAGTTAAAAAATGATTTAAAAAATGTTCTGATACGCACGAATGGAAATATTTGT

>scaffold45.1.1810928

ATTGCAATTCGATTTAATATTTAAATGAATTTTAAAAAGAAATTTTTGAAACATCATTTTATGAATCTTTTTTTTCAAATTAAAAGAAAAAATTTTTTGA**[T/C]**AAACAAAGCTTTTTCTTATTTTAACCTGAAAACAAGTTCGTTCATTAATATTGTCGTGATCAAAAACGGTCAACTCTATGATGAAATTTCATTTACATAA

>scaffold45.1.637207

TCTTGAAAAAATAATGAGCAACGTTTTTTAACAATTAAAAAAAAACCAAGAAATTTCTTAGAGAATTTTGAAAATTAAAAATCTACCAGAAATATTTCTA**[T/C]**GAAGAGAATTACTTATTAATATCTATTTCAATTTTTAAAATAAATCTAAATATATCTCGATCAAAAAATAAAAAACAATATCTCAAAATTTATCCATCCA

>scaffold45.1.668423

TACGGAAAACGATTATATAATAATAAGAAAATAATAATTAAACTTATATTTGATCCGATCGCCAAATGAACGAACAATTTACGAGAATTAAAATATATAA**[T/G]**AATTAAAATAAAAAATAATAACTATTATAAAAAAAATTTCTTTCTAATTTTATAATGATTGAAAAAAGAAATGAGTTTTCTTAACTAATAAATAATATTG

>scaffold45.1.760070

TTAAACGTAAGAAAATATAACCATATAAATAAAAACTCAAGCACAATTAGATACAATTATTAAAATTTCAAGCTCCACTGTATTTCTGCTTAAATCTTTC**[T/G]**ATCTAGCTATTTCGAAAGAAACGACCAATCATTAAACGTACCATTTCGAATCGCCGTTAAATCTTAATCCGTGAAATAGTAGCTGAAAGAAATCGAACAC

>scaffold46.1.1152356

TTTCTTAACGTCTAAACGCTACTTATAAAGCATCATTGACGTAATAATTGTTTTCGATTTCAAAAAAGAATATTTCAATCTGACACAATTCATTTGTCTC**[A/G]**TTTTTTTTCTATGACAAAAATAAGATATAAAAAAATATATTATTATTATTAGTCTTTTAAATGTCCGATCCTTAATCCTAAAAAAAGATTTTTACAATAT

>scaffold46.1.1767754

ATCATTTAAGTATTTTCCGATAAGCAAGATAAAAATATGTTCATCGTGGATTAAACAGATTGCAGAACTCGAAAGTGTTTAAACGATTGTAAAAAATATT**[A/G]**CGTAATTAATTTATTCCCAATTAATCAATTTGTTAATGTTTAAGAACGGTGGAGATAATTATGAATAAAAATAAGACAGAATTGATGATATTCATTAGGG

>scaffold46.1.1821058

TACTTGTGAGAAGTATAACCTATATAAATCTCATTATCATGACTTTTGTTCTTCGCATAACCTATAAAATTTAATAACTTGGCTGTGCCACACTTTTCTC**[A/G]**TTTGATATAATGCAAATGATTAATATTAACAAATTAACGGAAGACATTGATATTAATAAACATCTTCTAAGAATGAAACAAACAAAACAATTGATGAATT

>scaffold46.1.744261

ATCCTGTTTACGAAAAATAGAACGTGCAATGGATATAATTTTTTTTCGTGAAAAGAAATTTTTTTGAAGTTTACAACTGTCGTTATTCGTGACGTATTTG**[A/C]**TATCTATTTTCATCTCTAGTTTGAGAATTTTATTATTATAAAAAGAATTACCGAAGAATCAAGTAAAAAAGATTTAAGCTCGCAACCAATTCCGTAACTT

>scaffold46.1.1054433

TTCCATAAGATTATTTAATTTCGATTTTTCACATTTTATCGTTTAAATATCAAATATCTTAATACGAACAATTGTAATTATTTAATTGAACCAGTGATAA**[C/T]**AATTGGACGTTTAATATCCTTCGAGGAATAATTCAATCGATCGAAGAAATGCGACATATTTTATCTCTCATTTTTCACGTCTTTTTCCGAACTGGTAACC

>scaffold46.1.1100424

CATATTTTCTATTAATAGAATCTTGTATATGTGTTTTCATGTTTTCAATCTCTTATTTTAATGTTTTTATCATAGAACACAATCGAAAATCATTGTTGAA**[C/T]**GTTTTACAATCTGAAAATGATTGAAATCGTGAAAATTATCGTGAAAAATATTTATATATCTAGTATCAATAATTTAGTAATAATAATAAATTAATTGACA

>scaffold46.1.1207525

TTATAAGGAATCTTGATTTATCCTTTTCCAAATAAGTTGGGTAATTTTAGTTATTTTTCTCACCATGTATGTACGTATATGTTATATTATTTTTTTAAAA**[C/T]**GTATTTTTATGAAGTAGAACTATAAAACCTTTATTGATTCATCGCAATAAGAATGTCAGAGTTCTTCTAATTTTGGAATCCATTAGACCTAGACTCAAAG

>scaffold46.1.1287733

CTGTGTGTGTGCGTGTGTGTATGGAACAAGGTGTCTAGATGGATAGGAATAGTGGAATGAGTGGTTGTTTGTCATTGCCAAATGCAATGATAGTTTCTAT**[C/G]**TATGGACACGATTTCTTGAACGTAGGAATTTTAGAATAAAAAAAAAAAAAAAGAAAATCAGAATTTGAAGTAAAATTTTTGAATCTGTTTATTAAAAATT

>scaffold46.1.1675540

TATCGATCAATCTTTTCATCTGTTTGAAATATTTTTTGATAATTTATCTATTGAAATGAGGATTGGAGATGAAACGATATTGTAATTTTATGATTCGAAG**[C/T]**AGTGGCTTTTTTTTGTTATAATACAATGTTATAATACAATGAAAAAAATTATTTTTGCGACAAATATTCAGAAGTAAATAAAAATGAGATAGGCTGGCGA

>scaffold46.1.1729587

TATCGATATTAAAAAAATAGGATCTACACAGTCTTTTCAAATAAATATCAATATTTAAAATTACAAAATTATCAGAAGGAAAAGAGAAAAAAAATTATTG**[C/T]**AAATTATTTAATCTGCTTAAGATAATTCGCAACAAGATTGTCAATCATCAGCTATTTTATTTCGAACAAAGCAACAACAACATGTGCACACTGCATAAAA

>scaffold46.1.1858581

TACGTGTTTCGAGTCTTTTTCGAGTTTATCCATCGAAAAAATAAAAAAAGTGTCTTGTATCAAAAGATTCTTTAAGTTTGAGAAACATATTAGAAAGAAT**[C/G]**ATAATTAAATTTTAATCTAAAGTTTTTCTTAAAAGGAGAAGAAAATTTTTTAAAAAATATAAAATATTATAAAAAATTCGAAGACCTATACAATATTAAT

>scaffold46.1.2072192

TTTACATTTATAAATAACAATTCAAAATATAAATTTAAACGATTTCATGGATCGCTGAAAACACAATTGATAATGAAATTTGCAGTGTACTTAAATTTTA**[C/T]**GGAATTCTCACGATTAAAACTTGTCATTAACACACACGGAACTGAAAAATTGTCTGACTCTTCTCTGCGTCAAATAAGATTCCATACGTGTGGCGTCGAT

>scaffold46.1.2616468

AACGATCAAGTAGTCGTCGGTTAAATGATGATTATTGGTTTCGTTTCTTATGTTGTAATTTGAGAAATTTTCGTGGAATTGTTGCAAACTTTGTCCAGTA**[C/T]**GTTGAAGTACTTAGTGGCATGGGATCGTTTTAAATGTTTTTTTATAATAATGTTTAAGCAGTTAGGAATTATGGTCTGGTGACAAGCGATCTGAATCTGC

>scaffold46.1.784702

ATCGATTTAATATTAAAATTTATATAATTTGATAATAAAGTAATAATACGTGATGTCAGAAAATTTAAGTTATGTAATAGGATAATCATGATAATTGTTG**[C/T]**AAATTATTTGCATCTAGATGTAAGATCTAATGAAAAAATTATTTTTATATACTAATGCAACGGAATTATTGATTTTATTAATCTATGAAAAAAAATCTTG

>scaffold46.1.793969

CTAATGTCGTGTCGAGATTTTCCACGTCCAATAAAATTACGAATTATGGAATTTGGCACGTTTCAACGAAATATTTTAAATTGCCTGTAAATTATTCGCA**[C/T]**GTAGCCAGCTAGGTGAGAGTATTATTTTTATATAAATTGATACAGTATGCGGAGGTCGTTTACTCCCGATGACCCTTGTTACCTGTGATTTATTGTTATT

>scaffold46.1.1021897

AATTCATTGCATTTAATATTTAAGAGACGTTTTCTATTTTAAAAAAAAAGAAAATTAAATCAAAATGGCAAACGTGAAAATATTCATAACGAGTGCAATC**[G/A]**TTGCAACAAGAGAAAAACTTCAGTTTACAAAGATGAAGTTTTCGTTCGATCATCCTCTTCGAGAGCTTTTTCTTGTTATAAATCGAAAACACGTACTTTA

>scaffold46.1.1089694

AACTTCTTTAGATCAACAGAGTCAATTATATGAAATGCATTATAAAAAGACATTAGAAACAGAAAAAAAGAAATTTGAAGTAAAATATGATCAATTAATT**[G/A]**CTGAACTTGGTATCATGCATCAAAGTGTGGAAAAAAAAGAATCACGTATGAAAGAACTAGAGAATGATTTAACACGACATGTAAGTATTGATCATTAAAT

>scaffold46.1.1100465

TTTTCAATCTCTTATTTTAATGTTTTTATCATAGAACACAATCGAAAATCATTGTTGAACGTTTTACAATCTGAAAATGATTGAAATCGTGAAAATTATC**[G/A]**TGAAAAATATTTATATATCTAGTATCAATAATTTAGTAATAATAATAAATTAATTGACAAGTTACATAATTTTTTTTTATATAAAATAATGCAGTCAGCT

>scaffold46.1.1128008

TGTTAAATTAATGAAGAATTAGTGAAGAACTTAATTAGGTAAAAAAAAAAATAATTAATACGAATTTTAAATAACTTCAATCAGTGTCAGTAAAGATAAT**[G/A]**CTTAATAATTATATAATTATGCGAGATCGTTATATTATTAAGTACTAATTGAAGTGATAATAACGATTTACTTTTAGCGAATGTTACTTTTAACTTCGAA

>scaffold46.1.1302358

TGTGATATACGAGATTTGATGTTGTTTAGAAAGGTATAAATGAGTTATAGGAACATTTGATCCGATTGAGGTTAGGTTTGAATGGAATTGAGATTGACCA**[G/C]**TTTGGTAATGATATATACGAGATTTGATGTTATTTGTTTTGAAAGATATAAATCGGTTGTGGAAACATTTGGTCTGGTTGAGGTTAGGTTTAAAAGGAAT

>scaffold46.1.1530218

ATTATCATTTGAACTATATTTCCGTAAAGTTTTCAAATTTTATTATTTCTTTCCTGAATCATTGAATGAATACGAAGTTCAATATAATTCCATGTTCCAT**[G/C]**TACCTAACATAACCTGACTAATTACAACTTGGAAATTACACTCTTCATAAAAAGTGAATAAGAATTTAGAAATTCTGATCTAATCCAATTAGAATATTAC

>scaffold46.1.1561237

TACAAATTATAAATACCCTTAAAATAATAACGATATATCGCAATAAGATTTAATTGTATTCTATTAAATCATTTCAACAATGAGCACGTATCTTATCTAC**[G/A]**TACAGACATTCGGTCTGAATTTAACAACCATCATGAGTGAACATCTCTTTACACTTATCTTAAATATCTGTGTATAATACAACGATAAAAAATTATATTA

>scaffold46.1.1661034

ACAAAAATATCGAGTCAGGTTTTTAGTTATTAAATTATACGCAGTTTGAGTTTGTCTTGAGGACCAAGATGGAAACGGAGATGGAATAACATGGAGCGCC**[G/A]**TACAGCGGCGATGTGACGAAGATAAACGCTATCTCGCTTTATCTTCGTCTTCTTATCCTCGTCTAACGTAAACTTCAATTGCTTATAATGCTCGCTCGTT

>scaffold46.1.1760276

TTCTGTTCTTTTTTTTCTTTCCTAACCATATATACTGTAATTTAAATTTCATTGATTATCAATTTATAAATATAAAATCGAAGATGAGAGTTTTCAATAA**[G/C]**TTGTTATCAATAAGTAAACTTATATATATTTTTTTAAAATAAAAGTGATATAAATTACGTAATGAAAGATAGAGAGAATAATCGAAGAACAATTCGTTGA

>scaffold46.1.1953826

AGATCGATGATAAACAAGAATAAATGTCATTCTATATTTTTTTAAAGCAATTATTATTTTAATCAAAATTTTATTACTAGTAAAATTTATTAGATTAGAT**[G/A]**CAAATTTGAATTATTGAATTATTGAACTACTTTAAAAATTTATAAAAAAATCTTTAGAATTGTATTGAATACGATAGTTATTGTGTCAAATAATTGAACT

>scaffold46.1.2115160

TTAAGTGAAAAAGTCAACTTGCATCAATGTCTTAATTTTAAATCATCCCGTATTATTAATCCTTAAACTTTAAAGAAATTTGATCAAAAGAAAAACCTCT**[G/A]**CCAAAATGGATAGCGTTTCTTTAAAGAAGAAATCGGTCTTTTTACTCGAATCATTTCTTTCACCTACGAAAAACAACGTAATTATAAACAATAAACGATA

>scaffold46.1.2235771

CTTCGAACCCGTAATGAATGAATTACACGAACCATAAATTGTTTATATAACAAACGGAATAATATACAAGTTTTCATTTCCGGATTTGTATCCCGACAAA**[G/C]**TTGTCGAATCGAATCTTCGTTACACCCTCCATTATTCGAAATCACCCACCACAAATTAAAACAAAAATAAAATACAAAAACATCCACACGTGTATCGCTC

>scaffold46.1.2528012

GAAATTTTATAATCCCTTCGGGATTCTTTAGCTTTCTAGGGAATTGGATTAAGTTTATTTTTTCCCCCCATCGATATTAATAATAACGTATAATTTACAC**[G/T]**ATTGTTATATTTTTACATCGCGTAATACGTATTGTTCCACACGAAGTGAAATTTTCTTTACGAGAACCATATTTTCAAAATCGTTTAGACGGAACGAATA

>scaffold46.1.2616567

ACGTTGAAGTACTTAGTGGCATGGGATCGTTTTAAATGTTTTTTTATAATAATGTTTAAGCAGTTAGGAATTATGGTCTGGTGACAAGCGATCTGAATCT**[G/A]**CTTAAACTTTTCCACGTCTTTTTCTTTGAAACGTTTTCTTTGAAAATTGATTTTCAACGTGTCGAGATATAAGATATTATTTAAAAGCATACCGATGATA

>scaffold46.1.2921717

AAATCTCGAACCAAATTTCAAACTGTCATACTGATTCAGCTCTATCAAAAATCTCCTTTATTTCAAAAAACAAAAGAAATTGAGAATTCAAAGATTATTT**[G/C]**ATCGTTCGAAAATCAATCATTCGAACCAAATCTTAAATTATATCGTTCAAACTCCGCCGAACCATTTTTAAAAAAATTTTATCAAAAAAGTCAAGCTTTA

>scaffold46.1.2970465

ACGAGTCTCTTTTATAAAATTAAATTTCTTCAGTCAAATGTAAGCAGTATTCCTTTGCCTCTCGTCTTACTTGAGCTGCGGAAATGAAAGTTTTTCTACC**[G/A]**TGCGCATATATTCGTTTATTACATCTTCAGAAACAGATATTACACCTAGTATTATACAACGAATAATAAAAATATAAATAGAATAAGCAAATTTTCGTAC

>scaffold46.1.800664

AGTCAACAATTTTTTCTTCCTTGTTAATTAATTTTTTATCCCTTTTCTTTTACAATAGCACGTGAGATTTGATTTCAATCGTGGAAATACATTTTCACAT**[G/C]**ATTGAATTGACCAACTGGAAGAAAAAAGTTAAAAGTCTAATAACATCACGTAACATCAGAAATCATACACCGTATTATTCATATTACGCATAAATAAAAT

>scaffold46.1.1109055

ACTAAAACAAAATTAATTATTAATTATTTTTTCTTCAGTACTTTGACCCTCAATCGTTTACACTTTTATATACATTCATCATAATTTTTCTTACGTTTTA**[T/C]**GATATTTACATTTTACTATTTATTAACCTAAAAAAATGCAAATTTTCGCAAATCAAACGAGAATTTTTGATCGCTCTTCCAACTTGGAAATTTCCACTAT

>scaffold46.1.1845726

TCTCTTTAAAGAAGAATTGTTTCAAAATAACGTATTGAAACGTAACAACGTGATATATATATATATATATACGTGAATTTAATCATCTATCTCAGACGGA**[T/C]**GAGATTAAAAAATTCTATAAAAATATTCGCTACATAAATAATTAGACATTGAAGCAAGATATAGAAGTTGCGACAGCAAGGTTCGTTCAACCTTTGTATT

>scaffold46.1.2209912

ATCACGGATCTTCGATTCGATAGATGGTGCATTAGTTTCTCTTGAATTTTAAGTTATTTTTATGAAAAGCCGCATTTTAAGATCGACGTATTTAAGTAAG**[T/C]**AATTTGAAAGATACATTGCACATGTATTTAAGTAAGATAGACGATAATTTTAGAAAAGTTTCCTTTGTTTGAAGATAAATATTGAAGAGAGATTTCGCTC

>scaffold46.1.2371564

TGAAATTTCAGAATTTAAATCGTTTAAATTTTCAATATTTTTACAAACAAGAGACCTCGAAATTTCCATTATCATTCTCTCGATAATTAGAATTTTACCA**[T/C]**GGTAAAGATTAATGGAGCTTTACATTGTTTGTGGAAGAAGAAAACTTAAGAGGCACATATCATTTTCAATTGTGGAAACTTGAATTTAAGGAGCAGAACT

>scaffold46.1.2512774

ATTATCGAGTGTTGATCATAACGTGGAACCGTGAAACTGCGTTACCAAGACAAATTGTAAATTTGTTTAACAGCAATCTTTCGCTCACGAACAATTGAAA**[T/C]**GATGGATTTTACATATTTACATACGGAAATAAATCGTTATCGTCTCTTTTTTTATTTTTTTTTTTTTCTCGAAACTCGATATAAATTTTATTCTTCATTT

>scaffold46.1.2835115

TAAAACCTGAAATCGCCGCAAGTTGAAAAACGCTTCGCCTTCGGCCGTGTTCCACGCGTCGAATTTTTCTCTGGTGAACGTATTGAATTAATTCGCCAAG**[T/C]**ATTTCGTGCAACACGCAGAAATTAATGGGTATCTTGACGATAATGAAAGCTTTCGTGCTTCTTATACGTTAGAAAATGAAAATGGACACTGCGAAGATGA

>scaffold46.1.2969391

CATGATCGATCACTTGTGCAATACCATAGTGACACGATCAATTTTTACAAATTTTACAAATTTTCTTTATTCCAATATACAAATTAATTGTTAAAATTGG**[T/C]**AAAAATAAAGGCTATTGTAATGGACAAAAAAAAATTTTTAAAAACTTTAATTATCTAACTGTATTCTGTATTAGACGTGCAAGTGTATCTAAACAAGAAA

>scaffold46.1.707014

TCGTTTTCTCATTTCAATTTCCAAGAATAATTCTCTCTCGTATAATTTAGAATTATTAAAAACTGTTTTTAAACTGATGTAGTAAATGGAAAAAAGAAGG**[T/C]**AGAAGAGGAAACGAAATAAAAGGTAGAAAATATGATTTATCGTCCACTTTGTCTGCTTTTACGCAACAAGACGTCACGATGATTGCTTTTCTTGGTAAAT

>scaffold48.1.2014649

GATCGATTGCTGTTTCGAGCTTTTTGATTAACACGTTCCAACCAGCTGGGACGAATTTATCTTTCTCCTACTGCGTTTTTCTCCTTTCTTTTTTTCTTTT**[A/G]**TGCCTTCGAATACTACAAGACACTCGAATCGTTTTATCATGGTGGTTAATTTTATGATAGCAATCTTCAATTTTCTTCCTTTTTTTTTTTGAGATTGTTA

>scaffold48.1.2288588

TAAATCTCGAAAGGTTTGAATGTGATAACGAATCTTTCAACTCCACTTTTCTAATATTTAAAATCGAGAATAATTTTCGAATCTGCATCTCAGACTCTGC**[A/G]**TCTCTCGGAGAATTTCTCAAAAAGTAGCGGAATTTTACGATAATTTTCAACTTTGGTAGAATCGTTTAATCACGGATACTTCTAAATACGTAAAATTCAA

>scaffold48.1.368360

CAAATTCACGAAATTCTTCGAAACGAAAGAGCGTTTCAAAATAAATATCTATAGAAAGAATCGATTCTATATTTTTATTTCATCCCTTTCCACTAGAATC**[A/G]**TTCCTCCCACGATCAAATACAATTTCGAGAACACGGAGAATCGAAATTATAATCGCACATTTATCGCGATTATTCCAAAGTTGACGAGAGATCGTATTTA

>scaffold48.1.999137

TTTGAAAACAGAAGAAGTTTATAGCTATTGTTTATAGATTGTTAGATTTGTGGAAAATTCGAGAAGATTCTTGGCTTCTGTCCGATCTTCTCTTGGAATC**[A/G]**TTTTTGAAATTTTTTCAAAAGAAGGGTTGTCTTTGAACGTTGTTGTTCAATCGTTCGAAACATTTGAAAGTAGAAGTTTATAAGTTCTTGTTCATAGGTT

>scaffold48.1.1649272

TTAACAGGATGTAAAGTGTTAAAAGAAAAGAGTGTAAAGAATAATTAAATAAAGTGAAAAAATTTTTGGAAAAAATAACGATAAACGGGAAACGAGAGAA**[C/T]**AAAAAATAAAGAATAGAAGTAAAAAGCATTTGTGCGTGAAAAGGAATTCAGGGCAGACGAGAGAAACCGAGTTTGCAAAGGTGGTGGTTGCGTAGGATGT

>scaffold48.1.1944997

AATTCGTCATTCTGATTTTTCTTTCTTTTTTTCTTTTTTTGTTACCGAGGAGAACAGAAAATTTCTTTTTCCGAGGGGAAAATATCTTTTATTAATTCCG**[C/T]**GGAATTAAAACTCTTTAAAATACGGTAGAAAAATACGTGGTTAAGGGGATAATTAAAATATGTGAACAAACGAGATGAATAACGAGAAAAAATTTTAATC

>scaffold48.1.2025448

CCTTTCTCATAATCACGTAACGAATCGCTCTTCCCAATAGTTACAAATTCTAACAGAACTATTCTAAACCATTCCTAAAATCCTAAAACAATCTTCCCAA**[C/G]**ATCTTTGCAAACCCTCACAACACTCCTAAAAAAAAATAACAATTCCAACGGATCCCCAAACGGAAGAGAACAACAAATTGGAAGAGAAACGACAGACCTT

>scaffold48.1.2345651

CTTGACGAGAAAAGAGATAAAGACGATAAAAATATTCAATTTTCAATTTGACAATTCGCGCTAATAATCGTTAATGCTCCGTGTCTTTTGTTATTATAAG**[C/T]**GTTCGATAAATTGATCTTCGAAGTACGGAGATTGGAATGGGAATATCTTTACTTTAAAGAATCGTGAAGAATAATTTTATCCGAATTAATTGGATAAATT

>scaffold48.1.292080

TTATAAATCCTTTGATTTCGTCAAGTTAAATTTTCCATCCGTATTCCATGTATTCCGAATAATAATTTGAAAAGTCCCGAACTTTTTTATTTAACGAATA**[C/T]**GAATAAGGATCTTTTATAAATTTTTTAGAAAAAGGAAGAAGAAGAGGAAGAAAAATAAGCATAACGATTCACATTCACAAAAAATTACCCCGTACTTTCG

>scaffold48.1.359429

GAGCTATTTTGTATACAATCGAACCATGTGTTGAAAAGAGTATACTTTTGGAGCAATGAGATCAATTTATTTCGTAAAAAATTATTTACGATATAACGAA**[C/T]**GGAAGAATCAGTAGAGCGATATTTATCCATGCACGAATTTGATCTATTTTTATTCTAGAAGTTACGCTTATATCGCTTGCGTTGATACGGAAAACAAAAA

>scaffold48.1.481967

TCTATTCGGTAGCCAAGTTTTTATAAACAAGATTTCCAATAAGTTGTATATAAATAGTTTTATTAAGTTAATATCATACCTTTAGTTTAGGTGATAAAAA**[C/T]**GAAGTCGTAAAAAATTTATCGATATATCGATTACCACTACGATTGTATTACTATTAACTGTCACTAATTGATCATCAAAATTAAAATGGTAGTCACGCAT

>scaffold48.1.765372

ATTTTATGCGAAGTTTCAAGTTTTGTAAATATTGTAAATTGGAAAGTAATCGAGATGAAACAAAGTAATTTTATAATGTATGTATATTTCTATGTTATTT**[C/G]**ATATTTCAGAGATTACATTGAATTATTTTGGTAAATGATGATTAAAATTAAATTTAAAATTTGAGAATTTATCATTGATGGGTTTTAATATGAAATTTGA

>scaffold48.1.808465

AGAGCGAACCGATCAATCGTCATTGACGGATCGATGTTTTGAATAGCCAAAACGATCAGATATCGTTTGTGGATGTTTCTCCAAGTATTCGTGCTCGTAA**[C/T]**GTGATTTTTCTCGAAGATTTCGAGGATGGAAAAAGTTAAAATTTTATGAATTATGAATATTTATTTATTTATTTGATATAATCGAGCGTAATTATGATGT

>scaffold48.1.860796

TTAGAAGGGTAATAATTTATGTGTGAATGTACTCTTTGATGATAATAATCTAGAATTTTAATAATTTTTCGAATGCACATCGGATAATCTTTCTTTGATT**[C/G]**TTCGAGAAAAAGAAAAGAACAAAAAATTAGAAGAGTTCTTTTTAAGAGCCGTTTTAAAATGTTGAATAATTCACAAAGAGAAATTTTTCTGGAAGAAAAA

>scaffold48.1.964586

AGGCATAATAACGATTATCAACAGATTTGCAGAAACGCGTAAAACATAAAAATTCAATACATTTCGCGTATGTACATGTAATATATCATTAAGATCAAAA**[C/T]**GTTTTCTTTTTCGATAATTCTCTTAATTCTATCTAAATAATTCTATCTAATATAATTCGAACCAAATATATTTTATATTTAAATAACAATCCTAACAATT

>scaffold48.1.1121649

ACGAAAAGAAAGGGAAGAAATTGAAGTAAATGAATGTCGATTAATGATCGTAAGGGTCTTGGTCAATTTTTTAAATTTCAGAATCTATTGTATTAATTAC**[G/T]**CCTTCTGATCTTTTATCTTCTAATTACCAAACGAAATTAAATTCGATTTAATTTCACAATTCCGCCACGTATCATATCCATCTTATTTCCTTTTTCAAAA

>scaffold48.1.135255

TTATCTCATAGTGATTTAGAAGAATTGGCACAATTAAGCAGTGTCGAGGTAAAAGAATAAACATAATTATTTTTCTTTCTTATTTCTATTTTCTATTGTT**[G/A]**TTTAATTAGATAAAGAACAAAATTATAGTTGGAATATTATATTTCTAAATATATTTTTTCCCAATAATTATTTATATTCTCATTATTTTAATATAAATTG

>scaffold48.1.1397779

GATAAATTACTACCCCTTTCTCTATTAATCCACATTGATCTCTTCGCAAAAAAAAAAAAACAATTATTGGACAACAAATTTTGCACGATACAATTTATTC**[G/A]**TCCGACAAGAAGAAAAAAAGAGAGAGAAAAAAAAAATATTAAAAATTCTTCTAATAATCAAATTTCCACGGTAGAGCGATAAATTTCAAAAGCGCATTTT

>scaffold48.1.1456142

CATCCTCCACGCGATTTCAACTACTCGAATAACTCGCAATGGAGTTCGCTCGCTCCGCTTTGGTATTCGAGCTATTCAAGATTTTCTTTTCACTTTGTTC**[G/A]**TTTTATATAAATACTCCAACTGACCGTTTCACAATTCGAATCAATTATTTCGATGTAAATAAGTAGCTTAAAAAGTGGGAATGAAAAATCTCTTTAACGA

>scaffold48.1.1605889

AATATTGTGTGCGTGTTCATATCATAGAGATAGATAAATATTAAATGATAATAAAAATATAAACTGAAATGCTAATATGAACAAAAAATGAAAAATAAGT**[G/A]**CATGATTATTTTAAATATATTTTAATTGTAATTTTTTGTATTTTTTTCATTTCATGTTATTTACGTATACCGATCTCTTTAACAATACAATTATATTTCT

>scaffold48.1.1867279

CTAATTATATCTATATAATTAAGTATATATTAAGTATATATATAACAAGTCTTATTTTATTTATTAATATGTTCTATTTTGGTATAGTTTGTTACCAATT**[G/A]**CTTATTCGCTGATCATTGTTTTTTATTTCTTTTTTTTAAAAATAATTTGTTTTCTTTTCATCGAGTAACTTTTAATTAATAATAGGATTAAAATTGATCA

>scaffold48.1.2009057

CATTTCCGTATTTAAATAGCTCGGCAGTTAACTTGTGATTGATATGAATTTTCGTGTTCTAATAATTTTTACACCTTTATATATTAAATTTTACGTAAAT**[G/C]**AGCTGTTGGAATATAATCCGAGAATATATCCGTATTTAAATAGCTCAGCAGTTAACTTGCGATATAAATTTTGCTGTTCTAATAATTTTTATACCTTTGT

>scaffold48.1.2025455

CATAATCACGTAACGAATCGCTCTTCCCAATAGTTACAAATTCTAACAGAACTATTCTAAACCATTCCTAAAATCCTAAAACAATCTTCCCAACATCTTT**[G/A]**CAAACCCTCACAACACTCCTAAAAAAAAATAACAATTCCAACGGATCCCCAAACGGAAGAGAACAACAAATTGGAAGAGAAACGACAGACCTTCCCTTAC

>scaffold48.1.2257422

AGTGGCTGTGGTATTCGGATAAGTAGCGCGGGTTGATTATGGTTGGAGAAGTGGATTAAACCAGTGGATTCGAACAAAGTGCAAGTGTAAGCCAATTATT**[G/A]**TTGGTTGACAAACTTATCCCTTTTAACTTAATCTTTATTTTCGTTATGCGATTGTACCGTTAGATGCAAAAAGGATCTCGATAACAAGTTTTTATATCTT

>scaffold48.1.2283635

TACGAGGGATATATACTCCGATTCAAATTTCAAATTAAATCAATTATTGAACAAGTTACGAATCACTCGTTTCTTTATACAACAACATTTTTTTATTCTC**[G/A]**TCGAAGATAGAATAAAAGAAAGTAATGATATAAAATTTGTTAGAATTTGTGAAAGAAATTCCGTTAATTCTCTTCTCTCTCTCTCAAAGTTCAAAAACTC

>scaffold48.1.2358022

AAGCGGGGAACGCCCGTGAAGATCAACGACCCAACTTGTAATTCGATTCGACGCGTTAGAAAGTGAGTTTTAATTAATACGACCGATATCAGCGAACAGT**[G/A]**CAGTTTCAATGGTAAATCTGAACAAGATGCGGTGTAGCAAGAAACTGTCGGAACTTGTAATTAAAGTTCGACGAATACGAATCGAGCGTCGTTGTTGCGC

>scaffold48.1.2552589

GTGGATTAGAACGAAACTTTTTTGCAATTTCTTTTTTTATACAGAGCAGATACAGAGAGATGTGTAATTTCTCGAAATAATGTATCGATCAAACGGTTAC**[G/A]**CTCGGAAGATAAAATTTATTTACATTAACTTCGAGAAAAGTATTTATAATTTTAGCGCGAATCAAGATTTTTAAACGTATAATTTCCTAGCCTAATGCAC

>scaffold48.1.346553

CCGCGGAAGATTTCTCTCTTTCTCTTCCATTCTTTTCTATATAGCAGTATTATAACCATTCCGTGGTCGATTAATACTAATCTATCTTGCTTTTATATAC**[G/A]**TTTGAATGGAGAGTATATTTGAATACGAGCATAACGAGCGTTTCTGTAATAATATTTTTATGAAGATAAATATAAATTTTACGAAAGAATAATATTCATT

>scaffold48.1.665337

ATGCATTGAAAGTTCCAACAAGCTCGGTAGAATGGGTAACAGGAAGGGGTGGAAAAGTGGAAAGATCGTTAACGAGTCGTTTAATTGTCTTGGATTAATT**[G/A]**CCAAACGAATCCACGTTCAGGGTAGAAATGGAAGAAATTTCAATTAGGAACGATGACCGTTGCAGCTGTATCGTTTATGTAATTGGTATAAGAATAAGAA

>scaffold48.1.989947

TATAAGATTTTTTTATAAATTATATCTAAGAGAATACACAGTGATATGAATATAATGGGGAAATTAAATCAGTAAACACTCGTAAATGGCGATTTAATCC**[G/A]**TAAGGCTTTCGAATATCGATGACAGTCTTAAAAATAATTAATCTTATCTCGAGGGAGAGCCTATTGTTTCCCAAAGAAATCCCTTTGTAATTAAAAGTAT

>scaffold48.1.1388523

GTATCCATAATTCTAAACTCTATACCAAGCGATCAAATAAAATTCTAATCGTATTCCAGCCAGATTCTATTTTTAATTCTTATTAAAATTTATCCATAAA**[T/C]**AAAATAATAATATAAATATCTTCTATCCATCTTTCGATTAAAATTCCACGAGAAAGAGAAATCGTTACCAATTAAGATACCAGTTATCAATCGAATCGTT

>scaffold48.1.1953491

GTGTATCTGTCCCTCGAATCGAGGAAGATCGGTATCGAAGATTGGATACCTTCGAGGTAGTCTTATCGATTAAACGCGGCTTTTTTCTTTTTTTTTTAAA**[T/C]**GAATTTACGATTCTCATCGAGAGCACCTTATCGATAATTTTTCTTTCCTCTTCAATTCGTTAGATCCGAGTGAATTTTCGTTTCCATTGTTATTCGTCGA

>scaffold48.1.2116989

ATTCTACTATCACTATAGATCAATTTATTGTATTATGTGTTCAAATTCAAAGATTTACAGGTAAATAAATATTTAAAAATAAATAAAAAAATTAAAATAA**[T/G]**AAAAATGTTATATAAATTGTTGTATTGTTATATAAATGTTATATATAAAAATATTAAACTTTATATATATTATTGATATTAATATAGTTTTTAACTTCTT

>scaffold48.1.2265963

TCTATATCCCCCGAAAATACTTGAATTCTTTTATCACTTCTTATCGAAGCGTTATAAATTTCGATTCTTTTCGAACTAAAAATTCCCTCTTTTTTAACGG**[T/C]**ATTTAACAATTTTCATTCGTATCAAGTGTCGCAAATCAATTTCCACGTTATATTTGAATTCGGTCAATCCATTCGATTCGTCTTTAAGACCGCGTGTTAA

>scaffold48.1.328098

GTGAGAAAAGAGGTGTTATCTGAAAGGAAGCGAGGAAGAAAGGGTAAAAGGAGAGGTCCCATTCCACGTGATTAAATTTTCCAGTGGCGAAAATCCAGTA**[T/C]**GGAATTGGCACAGATGGAGGAATCGTAAAATCAACCGGCTCGAGAATCCGATTAATCGCCGATTACACGCGGATGTAAAAATAGTAGAATCAATCGACTC

>scaffold48.1.560931

TAGAACGCACTCCCTTTACTATTCATGTTCGTTTTATTTCAGAAATATTCTTTTAATTATTTTTATTCGAATAGATTTAATATTGAAAATATTTCTATTG**[T/C]**AAAAAGTTATTATTTTGAATGTTGCAACAATAATTATTATTGCTTTTTAAGATGCTAAAAAAAAATTTAAAAACACTTTTTATACGTTTTATATATTGTC

>scaffold48.1.938432

ACGATATTTCTTAATAATAATCACAGGTGATTTCAAATTTATTTACATTATCGAAAAGTATAAAGTTAAAATCGAGTTTAAGATTTAGGAGAATCAAGAA**[T/C]**AAGGGAAAATATTTACGAAAATGTTAAAAATAGAACGTCCTTAGAAACTGTCCTTAGAAATAAGCTGTGAATAGGAGAAGGAGCGTTTTCAATTAGAGCA

>scaffold50.1.198520

AATAATGCTTCTATTCATATAGCTGAAATTGTGCAATTCGTTTAAGGAGCATGAAAACGAAATAAAACATCTTCCATAGCTAGCACAATATCATCGAGTC**[A/G]**TTATGGTCTATTTTGGAGAACAAAGTTAGGGGCAGATATCCTCCTCAGGCTTCGCTGAAGGCACTTGAAAAACTATTATATGAGAGAATAGTATAATATC

>scaffold50.1.273298

TCAACAATTATTCGAAATCTCTAATTGAGATATTGATTCTCTATGAAATTACTACGTCATATTAATTGCATATGTACACTTTGATTAATACAAAGCTATT**[A/G]**CAAGTTATTAACTATCTAATTAAATTCTACAAGCGTCGGCTATAATGAAATCGCTATAGGAAAAAAGAGAATCAAAAAAAAGTGAAACGATAAAATATTT

>scaffold50.1.528871

AAAAATAAATAATCCTTCGATATATAAGTTTAACACTTTGCATGTAGCAATAAGAGGCAATAAAACGTTCTTAAGTATTCAATGAGGATATAAGCTTGAC**[A/G]**TACGAGGAGGAATAAATAATATAATATATAATGACATATCTAATAATAAATCGATTATAAATGTTCAATAACATTGACATTTAAGCGTGTATCGATGAGA

>scaffold50.1.371857

AACATCTTTCTTCCACTATTTTTCTTTGAAATCCTATATTTTTTTCGTCCTACACTTCAATTTCGTAGATAAAGTACAAGAAAAATTTTAATTTGCAATG**[C/T]**AATATAAGTAATAAAAAGCATAAATAGAAAAATGTTAAACTCAAAATTCGTTAAAATTGATATTCTTTTTTTTTTTCCATAAATTCTTCATATATATACG

>scaffold50.1.459627

CTCGTATCTGTCTCGATATCTTTTTCTACGCTTCGGTAGATAACTGTCAGCTAGAATAACTTGACAACTATAAGATATATAAATATTCTGTATCGATAAA**[C/T]**GGAACGGATATATTCATGGTTCTTCGCCATGCCATTTTATATCATGTGGACAGATTATTAAAATCGAAGAGAATTTTAAAATGGAGAATTTGAAGCAGAC

>scaffold50.1.464739

ACATAATTGAAATACATTATATATAAAATAAATGATAATTTTTGTTTTTTGATATCTAAAAATAAGTTAATTTTATTATTTATTGATAATTTTAGCAAAT**[C/G]**TTGACAAATTTATATTTATTTAATTTTTTCTAAATTTTCATAAAATAATATCATTTTAGAAAATTTTTAAAAATATTTTTTAGTAAGCTTTAATGAAAAT

>scaffold50.1.470796

CGCGTTAGTTTGCCCAGCGAACAACGTATCACGTGACATACTTATTGCAACATACAGATATTTCCCATCTAATAATACACTATATTTATTTATTAATCGA**[C/T]**GAATTAACAGACAAATAAACAAACTATGTGTTTTGTAAAAATAAATATTAATCTATAACGTAAGGAATTTTTTTTCTTCAGTGATTCGAAGAAATTCGTA

>scaffold50.1.57740

TAGTAATTATAAAAATATATATACTGATTTATAAAAATATTAAATAAATAATCACACAATGCAAACAAAGTTCAATGTAATAATATAATATAATTTTTTG**[C/T]**ATCTTGAAAATCAAAAAGATTATATATATAGAAAAAATTATTCAATGTTGAGTTTTACAATATATTAAAAAATCATTAAAATAGAAGATAACGACTGTTT

>scaffold50.1.297890

ACGAATTGGCCAAAATTTTGTAGAGTTTTAACCTAAAATTTAGAATATAGGACAATTAATGTTTTTATGTCTTTCCTGTCATTTGTAAATCTCAATTGAC**[G/A]**TAATTAATACGAAATATTGTACATTGAATGCGATGAATCGAAACAGAAATATAATTTAAATAAAAAATAAATATCGTTCGACAAAATGTCACTACATTCA

>scaffold50.1.366581

TGAAAAATTAAAAAATTAAAACATAATCTACAAATTATTTTTTATAATATCACATTAATTTAATATATATTATTTTATTTAGGATATTTCTTCAAAATTT**[G/A]**TTTATTTATTCTTGTTAAATAATTTTAATTCTAAATTTTTATATGAAAATAACTAGATTCCAAAAAAATGTAATTAATTTAGAACAATTATAAATTAATA

>scaffold50.1.374551

TTTTTATATTTTCACAAATTAAACAATACTGATCTTTCTTTATTTTATCGATCATACCACATTCGTTGCAATAATAACATAATTAAAAAAATTGCCGTTT**[G/A]**TTAATTATTAATTTAAAATAAAACTGTCTTTGTCTACTTATTAATACATTTTACGAGTAATTTTCAAATATTAAGATTCCAATTTTAAATTTCAATATAA

>scaffold50.1.399419

GTTATTTGTGCACGAATTTTTCTAAAATTTTTTTAGATAAACGCAGTTATTAAAATGAAAATAAAAATGAAAATATGATATTTATGTATTATGTATACCT**[G/A]**TTATTTGAAAAAAGGAAAATTAATTTTTATTAGGAAATCGTTTATTGTAAAATTTATATTCTATTATCAAATTTTTATTAAATAATTAATTTTTTTTAAT

>scaffold50.1.759763

GGAAGAGGGGGGAAATTTGGACCAGGACTGCTTCCGCTGTTGGGATCCAGGTTTGGTGGAGATACGAAACTCGTGAGTTTTTTTCGTGATTTTTATCGTC**[G/A]**TTGAATCGGTGTGAAAAGCAGAGAGTAGAGGAAATATTCGAAATTTATGCAAATAAATGATATAGCAATTAAAAATTATTTCGATATATAGGTTATTATT

>scaffold50.1.355347

AATCAAAATCGCCCAGTGTTGTTGGTGAAGAACTAGATTTAAAGGACATTGACACAAAGGATATTGAAAAATCTAGATCTTCGAGTGTTACTATAGAACC**[T/G]**AAAGAGGCCTTCGATAAATCAAAGTCGCCCAGTATTGCTGGTGATGTTACCGATTTGAAAGATGTCGATACAAAAGATATGGAAAAATCTAGATCTCCAA

>scaffold50.1.750077

TTAAAAAATCGCTCGATTAAAAGATTCTTACAATCGAATCATATTCCAGATGGGAAAGAAAAAAATACAATAATATTTTATTCGAAATGTAATTGAATTG**[T/C]**ATTATACAATAATAATTGAAAGGTAATTAAACATTTATAATGGTTATAATTGCAATCAACAGAGTTTCTTTAAGATATATTCAATTATAACTATCTTATC

>scaffold51.1.329287

TGTATTTAAGAATGTATTTAATCTAAATATATAGTTGAAAAATATGAAGTAATGAATATACTAAAAATTATATGATTGAAAATATAATATTTAATTCTTT**[A/C]**TGCATAATATTAAAATTTTTATTATATTTGAATTCAATGCAGAATATCAATTTATATTTCTTTTTTAATACATACCGACATATATTTTAACGATGATACT

>scaffold51.1.171425

TGTAAAATTCCTTCAAAATTTTCTTTCATCGATCGTGAATTATTGAATGACTAAAGAGAATCCTCTTCGCATTAACAGAAGATTTCGTATTATAGAAAAA**[C/T]**GAATTGAGAAGAAGCGAACCAGAGTTCGTAGGAATAAAATTTCGATTACCATTGCTAATAAGATATACCAATTTTTATATTCAAATCGAGATTATTCAAA

>scaffold51.1.210396

AAAACAAGGCTTGTCGATTTTTTTCCAATCCGATCTTATATTGTACTTTCAATTAAATTCGAATTTTATTAAAAAAAGGGAAAGAAGAATTTAAGATGAT**[C/G]**AGGACTGAAATTTGAAAGATTCTTCTTTGGAGAATAGGTGTGTTTTAAATTTATTAAGGTGTCGAAAAGAAAAAGGTGTTACGCCAAGGAAGAAAGTTTT

>scaffold51.1.185745

CTCGCGTTATTCGTAATCGAGGAGGATAAGTCCATCTCGTGTTTCCTGTATCCCCTTCCTTGGTTGCGTTTAAACGAAATCGTTTAAACAAAAGTTACTC**[G/A]**TTTTCGGACAAGATGCGTAACAACGACACAGCTTTCCTGATTTTTACGTATTCTCGCTTCGATCCTCTTTGAGAAACAAACTTTCTCGGCATCTCGAATT

>scaffold51.1.253098

ACATTATATAAATTACTTCTATTCCAGAAGTTTTTAACTTTCAAAAATGATAAGAAAATACTAGTCTACAATATGACTAGGATAAGTCCGTTTTTATAGT**[G/A]**CTAAATGAAAAATTAAATGCTTATATTTTATATAACTTTATTACAGAAATGAAATAACATTTATTCTGTACAGATTATTTTAAGAAACACTAGTCTTTTT

>scaffold51.1.152527

ATACTTTGTATATCGATACACGAAGGAAAAAAGGTATATTTCGATAATTTCAACAAACTAACTTCTCTTCTATTTTTCTTTCGTACGTAGAATCGCATCG**[T/C]**AGTAAACAAAGACACGTTTCGACCAATAAATTCAAAAAACACATTTCCATAGATCCAATCACTTTCCAGTAAGACACTCAGCAATAGCACCCAGCAATAA

>scaffold51.1.38668

ATTTCGGAAAAGCTTTCAGAGAACGAAGCGAACCGTGGTATTAGGAGTATTAAATAAGAGAAATTTCATTTCGAACGTTTCTATCGAACTATTTTGAATG**[T/C]**AGAACTCTTTTCGTTTAAGCGAAATTTTAATAACGATTGTTAGCTGATCGGGCTCGCATCAACACGAATATATACATATAATTTATCGATTACAACTGTG

>scaffold54.1.221225

CGCAATTAATTGGAAGATACGGAAGAATGATGATGTAATTGGACGTATTCTGTTAGGTAATATTTTTGGATGAAGACTCTCGAAATTCATTGTGACGGAA**[C/T]**AATTCATTATATTTATTATATTGAAACGAATATTTCAAGAGATCAGAATGGAATGAAATTTGAATATAAAAGTTTGTGAGATCGTGAAACTGTTGAAACG

>scaffold54.1.607094

TTACTGGTTCAAACTTTCTCGTGTCAATTTTTTTTTTTTTGGAATTACATTACTAATTTAAACGTTGTTGTGTTAATTTTCTTTACAGATTGGAACAGAA**[C/G]**TTTTTTTTATTTCTTCTTTTGTTTTTTTTCTTCGTATCTTTTTAAAAACAGAAAAATTAATTCTTTGCATGCAAGCAGTGCACGACACAATGCATCACGT

>scaffold54.1.109956

AAAACTTTTTTTCTTTATATTTTATTTTATTTTAATACTTACTTCTTTACAAAAAAAAATGTTGATTACTAATAGTATAAGTATCTTATATACATATTAT**[G/C]**TTCATTAATAATTGTATTTTTAACCTAGGTTATAATAATTAATTATTGCTGTCACAATTATTGATGAAAAAAATAACCTAATTATTTTATAATTTCCCGC

>scaffold54.1.122199

AATTGTTAATTGAAACATAGAATATAGTTGAATTATATAAATTTAGAATAAAGTAATTATTCTATATACATTAAGATTAGAAAAGTTTAATTTATCTTAT**[G/C]**TTTGATTATTATTTTAACATACTTCTGTAATAAATAATTAATACAAAACTTTCTTACAACATAATTTTAACTTAGAAAAAAAGAAAAAGAAATTTACAGG

>scaffold54.1.160002

ATGTATTAAAAATTAGAATAGATACAGATGTCAAGATTCCACTAATAATATTATAAAAATATAAATTTTCACTAGACTTAGATTTTTTCAGCCTGATTAC**[G/A]**TCATTTTTACGGAGAAATTATATAATAGATTTTATAAGATCGTTAAAATTGAAAATTTAGGTTAGAAACGACCAATGATTGAAAATATCGATAAAACTGG

>scaffold54.1.192026

CGATGGAAAAACAGATTTTTCCAGCTATGCCGATGCACTTTGGTGGGGCGTGGTGAGTGAAAATTCGTTTTTTATTATATTTTTTAATGCACGTTTATTC**[G/A]**TTTTCTTTATTATTCGTTAAATTGCAAATGTTTGTGAATTGAAATTTTTATTAGCATAATTAGAAAAATATAAATCTATAAATTCATTCTTTGAATGTAT

>scaffold54.1.301034

AACATTGTTTTATTATGTAAAATATATATATATATATATATATATGTATCTATTTTCATATCTAATAGAATGTTATATTGGAATAATTTTGAAATATTCT**[G/C]**AAGATCAAATGAAAATTGATATATAGAAAAATGTCATAATTTACTTATTAATTATAAATGATTGCTCGAATATAGATAGAATTGTATTTTACTTTATCTA

>scaffold54.1.649417

ATCGAGCCTTGTTGCCTCGTGAAGGAAAAAAAAAAAAATTATATTAATAGACGACGATACATCAAAGTTTCGCATTTTCGCATTTCGAACGACTGTGCAT**[G/A]**TACACGTTCGAGAGTGAACATAGTCGAAATAGCACGGTAAATTTTCAGAGCAACCGATCAAAGTCGATCAAACTTTCAAACTTTGACAGCGAAACTTTCC

>scaffold54.1.233364

TCAAGAAGCTTGTCTTTGTTAAGAACGATGAATGAATTACAATTTGCATAAAATTTGTACTATATGATGATAAAAGAGGTGTGAAACTTGGATTACAGAG**[T/C]**ATGTTGATCACATTAATTCAAATTTTCTCATTTTTTCTTATAAGAAATTATTAATCTTCAATACTAGTCTCTGATATTTAAGACTATAATGAAAAGTTAC

>scaffold54.1.253317

TTAATAAATGAGGTAACCAAAAATATTCTGTCATTGCTTTTTTTTATGAAAAAAAAAAAGAAAAGTATAAAAAGAATAAGAGTCAGAGTAATTTAAAAGA**[T/C]**GAGGATATATTTAATAAAAATAATGATACATTTCAACATAGACATGTATACATCGCTTAGAAGACAACAAATTTATAGCACGAATTTACTTTTAACGAGA

>scaffold54.1.867151

AAAATTTTCTTTCCCCCTTTTTTCCCTCTTGTTTCAATTACTACTAATTCTTAACGAGCCACCCTGTATATTTCGTTCCTCCACGTTCAGCCACCCTACT**[A/G]**TTCCCCCTCCTGGTCGTTTTCAATTTTTCTCACGAAACCGGCCTCACGCCTGGCCTGGTGGTGCACCGTTTAACGAGATCAAGAATAAATAGCGGCGTAA

>scaffold63.1.255096

GGACGCGCAACATCTTTGAGACGAGACATCTTCAATTTTTCAACTCTGTAAGCGTTTGCCGTGTATAAGATAAAAAAGTGATGAAGTTTATTTCCATCTC**[G/A]**TTCTTCGAATTTTTATGTCTTAATTTTCCTCTTATTTATTTGATTTAAAGAATATTGATGTTTTAACAGAAACTTGAATAGCGAATAATTCATCGAGTAA

>scaffold63.1.209006

ATATTTTCCAAATAGCCGGCGATATTATCCGTTGCACAAGTTCCGTAAATTGTAAAAATCTCTAACTGAATAAAATTACTTCAGTTTTGAAGAATTGAAA**[C/G]**TTGGAAAAGCTTTTAATAACAACGATTTAATAATATCGATTATATTCGATGATATCTTTATTTCTTTATGTGGTTTTCCTTTATGTAGAGCATATCGCGT

>scaffold67.1.142783

TACTCAAAATTATTATTTGCATAATAATAAATTATATGTTCAGCATGAAAATTACCTGTCATTATATTATCATTATATCTTTATCTACTAATAATAGTAA**[C/T]**GTTTATATTCTAATAATAATAATAATTATATTTAATATTACATTTAATAAATGCTTACTCACATAAATTTTTCGATAAAGGAAAATTGAATAATACACTG

>scaffold67.1.250757

GGGAAACTTGAACGCCGAGATCGAGACATTTTAACTCATGCTTCCGATCGAAGCATCACGATTAACCTAGCGGATTCAATAAAGAGATAAAAGATGCGAA**[C/T]**GTTCTCCTTCTTTTTATTCTTTTCCATCCGTTTAATATTGAATTATTGTCCAGAAAAGTGCATATAGGAAATTGATCAAAGTATATGCAATTAATTCGTG

>scaffold67.1.48600

TTTTTATATCAGATAATGAGTTTCTTAATTTTAATTTAATTTTAGTATGATTGTTGTTTAGGTATTTTGTTTTAGATTTTACACTTAATTCTAATTTAAA**[C/T]**AAAATTATTCAGTCAATTAATATTGACAATGATGAGTTTAATTCAAACTGAAAAATTTCATAATAATGCTTAATATATTTAATTCATCGCGTAATTATTT

>scaffold67.1.80022

TTACAGCGGTTCAAAGTTTTTCTAATTTTAATAGGGTTTAATCGGATTTACGGTGAATGATTAAAAATTATTTTCTTTTTACACGACGATATTTCAGAAA**[C/T]**GGGAAGTCGTATCAAGATAAGTAAAAGGATTTTAAAAGGAAAGATTCTATGTTTTTAGTGATCTCTTATTCATTAATCAAAAATCATTATTTTTGAACTT

>scaffold67.1.93846

TATGATCGTCTTAAATTATTTATCGTTGTTTATATCGTACAATCATTATTTATTAATATAATTAAACATTATCTCGACAATCGAACTGTTTTATTGGAAA**[C/T]**AATTCATGGTTCCAAAGTTTATTTTTCTTTCTAAGTCAGAATACACGAACATTTACGGAGTTCTTTTATCAACGACGTATCTATTTGCCTAGCTATGGAA

>scaffold67.1.68096

TGCTTGAGTTTGAATACCTAATAAATTCCAAAATTCAATTATCCCAAGAACAAGCCTTGAAATTAAAATTTTTTAAAATTTCATTCTACATTTTCAATTT**[G/A]**TTTTTGAACGTAGAATAACCACTTATACTACTTAATCCCGTTACACTATATACCTCTTTATACCTCTGTATCTCTATACCATATAACATAAATTCATATT

>scaffold67.1.17431

GGCTCACCGGCCTGATGGTATCACCGCCTCGATTTTTATCACCGTCGTTAGCAAAAAAATATTTTACATAGAATAGTTTTGGAAAGGATACGTTCCGTGA**[T/C]**GTTACCAGTTTTGTAGTTTGTTTCGAAAGAAGAAACGCGAGGATCATTAACGATTTTTTAAATGGGATCGTATATTTTTTATTCCTATTATTATTGTATA

>scaffold67.1.205316

CTCCAGAGTTCTTCGACGCCAACACAAGTTAAATATCGTAGAATATTTGTTCAACGAGATAATTGTGTTTAGGAAAACATTGACTCGAATTTGTTTGCCA**[T/C]**GAGTATAATCTTGTTGATCGTGAATATTACTTATTGAAGTACTTGCATGCTTTGCGCGATAAGTATTATTGTATTGAGATATGTCAATAAATGCATTATA

>scaffold67.1.183163

AAATGGAAATAAAAATTGATATTCTTTGTTTAAAAGAAGAAAATTTTTACGGTTTTTCTGATTGCATTGCTTTATGCTTATTTGGCGTGGAATATCAAAA**[C/T]**AATATTAAAACAATTTTTTTAAAATATATTAATAAAATTAAAAAATTTTGAATATTAAAAAAAAAACAAAAAATGTTTAAAATAATATAAAATTATTCAA

>scaffold67.1.199329

CTTGATCCTTGGTCGTTCGAGGATTTTCCTTTGCCCTTCATAACTCTCTTCGGATAATGGAATGTAGAGATGATTACGTTGAAGACATATATAAAAATTG**[T/C]**AATATTATTGTAAATTCTTTGTATTTAATGGAAGAGGATTATCTAAAGATTCGAAAAGTTAATATCGGATTTTATGCAAATATTAATATTGATAAGTTAC

>scaffold70.1.1552922

AAGCTTCTTGAAAAAGTTACGCAACGTTCATTAATCAATCTCGTAATCAAATTTCTGCCTCGTAATCCCGGCAAAAATTCATTCATAATATATATATTTC**[A/G]**TTAAAAAGAATTATCGCGCAAAGAACATTTCCCATTAATTTATAACTCTTTATTGGCCAAATCCTCTTAATGCAATCCTAGTAGCATTTTACTGGATGCT

>scaffold70.1.1846404

TCTTAAAAATTTCCTATCGTCCGAAGATTTAAATGAAGAGGGCTTTTCGTGAAAATTAAATAAGAAGAAAGGAAATCAAGAATAATATACAGAGTGGAGC**[A/G]**TTCTTAAAAAAATTAGATTTAAATGAAGAGGCTTTTTTTGTGAACATTCTTAAAAATTTCGTATCGTTCGAACTAGATTTAAATGGAGACTTTTTTTGTG

>scaffold70.1.2137689

TTTTTTTAATACTCACGGATAAAGGATAAATAAATTAAATAACTAACGTTTAACGATTTCTGTTTCAGGTAAGTAGCCCGAGATGACGGTAATCTCGAAG**[A/C]**TGTTGAAAATGTTTTAAACCATTCTGCGGTGGTAAGTGATACTTCGGATCTTACTTTTAATCACCGTAATCACTCGTCGTTTTCCTCTCGTTGTCGCAAA

>scaffold70.1.246056

AAGTAAGATCCTCTTCTGCCAACAAATTAAATACGACTGCAAAAACTAGCAGTACTACAACTACTGTCAGTACGGCTAGCAACAGACCTAAAACAGCTCC**[A/G]**TCTAGTGGAACTGCATCGAAACCTCGCATGTCACTTAATAAGTTACCAGCGATCGATAAACAAGTAAAGGAAACTGCTAACAAACAAATTTCTATGGGGC

>scaffold70.1.2559154

TTACGCGAAGTATCACGTACACGCACGCTATTAAATTTTTCACATTTTTACAAAGATAGCAATTTGAAATGTAATATGGAGACTAGGAGAAATATATATT**[A/G]**CTTAGGGATTACAATAATATTTTACATTTGTTTACAAATGTTTTACCTCGTAATTATGATATAGTGAAAGTACAATTTATTGTAATATACGAAAACAAGT

>scaffold70.1.289623

AGAGAAGAGGAGGTCTTCTGATATTTGCGAGTGGAAGATTTGGCTTAAATTTTTTTTAATAATTAATAAAATCGTGTCAATTTTGATTTCCAAATTTATC**[A/G]**TCATTTATTACATCGTTAGTTTGAGAATTAGGTGGAAATTTGTGGAAGTATTATGGAAAAGGAAATTTCTGATTAATTTTTAATTTAATCATTTTATCTT

>scaffold70.1.379130

TATACCATTATATACATATTATTTTCACTTAGGTGATAACACAATTGTTCAAAACTCAATATATTGTTTAAAACTATAATGTTAAAAACGCACAAAAGTT**[A/G]**TCTTTTTACAAAAGAGATACGATCGTTTAATTAGAGCTACTTTAGTGCCCTCTACGAATAGATGCAAAAGCATGGTGAACCATATAAGTTCGCGCATGTC

>scaffold70.1.1674390

CAGCTTGAAATGAGTTTTATATATCCTACTATCCAATTATATATCCTACACAAGGGGATTATCCAACGATGTTTAATCTTTGTAATATCTCGTATAACAT**[C/G]**AAAAGAAATCGATGGATAATTTGATATTAGACAGATAATAAAAATTGTCGAAATATTTCCTTTCCCTCTCGAAATATATTTCGTTGTACTGTAAAAGAAT

>scaffold70.1.1761330

TTCGAGGAAGGATGTTATGTGTAATTATTTATTTACGATATATATATATATATATTTTTTCTTTTTTCTATCCAACTTCAGAATGATCGATCGATAGATT**[C/A]**GGCGAAAAATGATGAGTTGAGGATGAGTTGGAGAGAGAAATATGCTTTGTGTTACGCTATTCAATAATACACTACGCTTTTGTGAGTTTATCTTATTCAT

>scaffold70.1.2025094

ACCGTTTTAAATTGCAATACAATGCGTTTACATTTAACAACTGTTGTCGTTTATCAGTTTTTTCCTTTTTTTTTCTTTCAATTACACCGGTTGTTGTAAA**[C/T]**GCAACGCTATAATGCTCGAACTATAATGGTCGTAAATTGCTGTTCACGCATGTTACACGTGTTGTCAAAATTTATTTGCTTTGTAAATTATGAAATACGT

>scaffold70.1.2367212

GTAAATTGTAATGTTAATCGATGAATGGTAAGACACGTTAAATCATTGCTTTTGCAATTTTATTTCTACTTATTAACTGTTTTCGTGATTATTCAACTTG**[C/T]**AATCCCAGTACGAAATAAAAAAAATTTGAAAAATCAACACGATAGAAGCATACATATATTGGAAGCATTATTTTGTTAAAATTGATTAAATGAAGATTGA

>scaffold70.1.2416379

TATTTTTTATATGATGTTTTGATTATGTTATAATTTCTTAAAAAATACAACTACAAAATTTTATATCTAAAATATCTTAACGAAAAAAAATTATAATAAT**[C/G]**ATGATATTCATGAAAATTTAATAACTATCACATAAATGTCACTAATTAAATAATCTATTATATAATCAGTATTGTATCAGTTTTTATTCTATTCACTTTA

>scaffold70.1.2664419

CAAGTTGTGGAAAAATGTTCAATTTCGAGTTGTATAACGGAATTGACATAAAACGGAATTGGTTAAAGGTAAAAAGAATTCTCAATCTATTTTCTTTAAA**[C/T]**GACTGCTCTCCCAACAACAAGGTCGAAACAATTAGCATGTTAAGAATATTTTGCGATACGCATTGCATTATACCTCAAGGTTTTGTAATTCACGGCGCTC

>scaffold70.1.2692291

AATGTCAACGTAATTGATTTAATTCGCGTACGTTAAAATAACAAGCGATTCCTTTTTTCGAGCATAGACAATAATGGATTGAGAATAGGAAAAATTGTTA**[C/T]**GGAATTTTAGTTGGAAACAGAATTTCGAGAACGCAAGAATCTATTTTTCTCGTGCACGCGTCATTGATGGAATCGGGAGTAAAAGATTCATGGAACGGAT

>scaffold70.1.281406

TTGAAAGTTATGATTAAAATAATCTTTTGTGTATAAATTATTTAATATATTGCATGAAAATTGTAATATTTAATTTAAAATTATTTTATGTTAGATATTT**[C/G]**TTATGTTTTACGAAAGATGGTATATAAGTTAAAATAGTAAAGAAAAAAAGCAAGAATTTTTTATCAAATCGTAGATATTTTGTAGATTTTTTTGTAATAT

>scaffold70.1.2980986

TCGTTTTGTATATGCAAATTTGTGTGAGTGATCAAACGTAGGTAGGTCACTCTTGGTGGATAAGTCCATCTTAGAGATATAGCAGACAGTTACTAATTTA**[C/T]**GGTTAATATTCTTTTTATCCTTTGAGTTATCGTCAGAATCTTTTTACGATTATCGACTGAATAAATTTCACAAATAAATGAGCATAAAATGAACAGAATC

>scaffold70.1.3011110

ACTATCTGCCCTCTTTCTCCTACACTCATTATCTTTTTCTATATTCACATTCAAAGAGCAAAGGAAACTTAAAATAAGTTTAATAAGTTTCGATTGCTCG**[C/T]**AGTTTAAGAACTCTCTATCCTCTTTCTCCTTCGCTCAAAAAGCAAAAGAAACCTAGAACTTTGATAACTTTTCGATTGCTCTTCAATAGAACAAATTTTA

>scaffold70.1.3150311

TCGATTCGCATTTAAGACATCGTAAACAAGTTCGGAGGTATTTACGTATATTTATATCTCTTCCCGGACAAATTGGTTATTTTTGGTTAACTTTGGATCG**[C/T]**AAAATGTAAGACGATATCGCACTTTCGTTTGGAAATTATTATTTCATCGATTGATATAGATTCAAAAGTTTCTTTCCTCTTTTTTTTTTTCAAATTTAAA

>scaffold70.1.3386224

TAATAATAATAATAAATTTTGTATTGTAGGATATAAAAAAAACATGAAATTCGGAATATAAAATCCTATATTATATCAATATAAAAGTTATAACAATATG**[C/T]**AAGATGACGATTACAAATGAGAGATCAATCGTTTTAGATGATATTTTTTTCTGTATTTTAGTGAAAATTCTAGAATTTTTTGTTTTGTTTTATTTTTATT

>scaffold70.1.3567740

TTTAAATTTTTCTTAGAATCCAAACATCCTGTGTGTAATATAAAAGTTAAAAAAGTTTGTAGAAAAATATAATAATTCGAATATTTGAAAAGTGAAAAAA**[C/T]**AAAAGTAATAAATAATTATAAGTATATCGAATTGAATACTGTAGTATCATACTTAACAATCACGTGGCGCTAGTGTCGGGTTACTAATTGTTATAATATT

>scaffold70.1.3633420

ATGTTTAAGAAAAAAAAGGAGAATAGTGTTGTTAGCTTGGTTATGTGAGTTTGTATTGGTTAATTATTAACACTAAAAAAAGAAATGGAAAGAGATTATG**[C/T]**AGTGAAAGAGAAAGGAGATTTTTCATTTTTAGAAAATTAATGTAGAGAAAATTAAAGAACGTGATAAAAGAAGAAAATTATGCTATATTTAATAATATAA

>scaffold70.1.966120

AAGGAATGAAAACTATAGATACGAGAAAACTTCATGAGAAAATGTAAAAGAACTTCAAAGACGATGACGGAAGGAGTGGGAAGAAATAGAAAGTGAGAGA**[C/T]**GGAATCGAAAGAAAGAAAAGAAAGAAAATAAAATAGGAAGAGTTGACACCGCTACGAGAAACACGTTGCCCGCAGAAATTCCATTCCCGTGTGATTCGCT

>scaffold70.1.97949

TATAGATTATGAATAGGTTTCTTAAAATTATATAATTTCATATCGAATGGAATATGCATATAAAAAGAAATGAAAAAAAAAGAGAGAAACCTTCAATTTA**[C/T]**GCGATAAATTATTGAATTATAATATTATAAATTAATTATAATTAATCATCAAGTGACAAATTATAGGTTATGAATTCTAATAGATTTCTTGAAATTATTT

>scaffold70.1.107170

GAAAATGAATAAAGCATCAACTCGATATCCTTTCAAAACAGTATCCAAATTATCTTCTTTTTCCCTGGAATTTTATTTCCTCTTCAAAATATTGTAGTTT**[G/A]**TTCAAGCTTGCTTAAAAGAAACCTTATTTTCTCTTTTCAAAAAAAAAAAAAACTCGAAGAAAAATTCTGAAACGAAAATAGATATGGGTAAATACGAATG

>scaffold70.1.1345330

ATTAATTATCTATCTACATAGTTTCATGGAAACACATTATGTTTAATTTAAATCATTTATTTATAAATCGATTATTATTATGTGAAAATGATTTAGATAT**[G/C]**ATTGGAAATTAATAATGTATGTTTCTATTAATTCGATTCGTATATATTTTTTAAAAATTATATTTATATAAAATTAAATGAGATATAATTTATTCCGTGT

>scaffold70.1.1459506

TCTTTTATTAGAAGCAATTTCTTAATATCTACAGAAAAATCATGTTCTTGACCTAAAAATTTAAAAATTAATTAATTTTGAATCTTGCTAAATTATTTCT**[G/A]**CATCTACAAATTAATACCATAAGAAATTATGAGCATTCATTTAAAGAAAAAGTGCAATTGGATCAATACACCTAAAATAAAATCCACCCAATCTCATTCA

>scaffold70.1.1487918

TATATCGCGCGAAAGATATGATAAATTCGATTTATTCACGTTAAATTATTTTTTTCCTATCTTGTTATATTACCTTTAACAGCTCAAAATCGTTCTTGCT**[G/A]**CAAACTTCTCGAAATTAAGTAAAATAAATGAAATGAAGAACACGAATAACTTTTTGATCAAAAAGTTAATATTATAATTTGACGGATTACACCCATAAAG

>scaffold70.1.1620471

AAATGCAATCATCACGATCCTCTTTTGATTCTTGGAACATTTTTTTTTCTTTTTGTTAAAGTAAAATACTGTTAATTTTGCCAGTTGTAACAATTGTATC**[G/A]**TAGGGATAATTAAAAATTTTAATTTTAATTCCCCTCCCATACATCCTATTATGATAAAAGATATAAAAATTGAACGTGTTAAAAGTATTAATTTTTCAAG

>scaffold70.1.1885512

CGAGATTTCGTTTGCTTATCGCCTGTATCTTTGTGAATATCGTCACAAACGTGGAGAAGAAATCGAAGGAATTTAAATGAATTTAAATAAGTTCATCGTT**[G/C]**AAAACGGTGATAGCGATTGCAAATGATTTATAACGAGATCGAATAATAAACGTTTAATCGCTTAGAGTAACTTTTAACTCTGATATATTCAAGCGTCATC

>scaffold70.1.2268326

GTTATGTATTATAATCGTCAAGCGTCGAAAACGAAACGTGTTTTATTTGCCCTAGCGTTTATATTATCAAAATTCAATTTTTCTTTCCCGGCAAGTATAA**[G/C]**ATGCAACAAGTATATCGAGTAAAACTCGATAAATTTACGAGGCGAATCGAATTTCAATCTAGGAGAGAGACTAAAAGTAAAATTGTGAATGAGGAACGAA

>scaffold70.1.2383981

GTTAGAATTAAATCATTTTTTTTTCAATTATTTTTTATTACATAAGATTAATATAATCTTATAGATTAAGATAATAAAATCTAATTTAACATTAATATTA**[G/C]**ATAACGAACATGATAATATTAGATAAGAAGCATATAAAAAATAACAATATAATTTTTTTAATTAAAAAAAATATTGTCGAGACCAAGAAAATGATTTACT

>scaffold70.1.3257082

ATTAACACCATCTTAGACGTAGATACTTCGAATCGTTATATCTTGGCAACGAATTGAGATAACGGGATAAGACAAAAACTGATTTCAACGGTACGGCTTC**[G/A]**TCTACTTTTTAAATGGATCTCGATGACAAAATTTTTTACTTTATTTTCACAAGAAAAGCTGAAGTTTTTCACTCGTGATTTTGCAATGATCTTTATCTCG

>scaffold70.1.3631691

GTATAAATAGGTTTGACCCACTTTTTAACTCATGATAATTATAAAAATTGCAAGAAGTTTCTATTTGTTTCTAACTTTATCTTTCTTCGTTTCTGAATCC**[G/A]**CCATTTTTAATTATTTAGTTTCAAATTATAGGAGATGATAAACAAAACCGGTATAACATCGTTCGAAAATTGCAAAGAAAATTGAAAATCGCAGTTACAT

>scaffold70.1.3989814

CTGTTCGAATTTAATCAATTTTCAAAGAAAGGAAACTCTATCGTATCCTCTGCCATGATTGAAACGCTCGACTCGTTCGAATCTCCTTTCAAAATTCGAC**[G/A]**TCGTATATCTCGCGCGTATAGATTGCGACAAGATTACTACAACTGGTGGCCACGACAAGAACGACGAGGGAGTAGGTTCCAAAGCACACCCCTTTGCCTT

>scaffold70.1.59697

TACATATATAAATATACATATATGCAAATATTTATTATTGAATATGATATGGAATTTCACATTCAAATATGAATTCTTTATTTTCCATCCCGTCAATATT**[G/A]**CTCTTTTTGATATTAGATTGAAGTTTAAACGAATATTTTTTCGAAATTAGAATTTCGAATAGGTTAGAAAAAACAAATATTTAATTTTGATCGATAAGAT

>scaffold70.1.786878

AAAAGATTACGTAGAGAATAAGATAAATTCATATTTCAATGAAATCCACTTGCGCTTAAAAAATCAATAAAAAAAATAAAAATTAAATAACTTACAAATT**[G/A]**TTTATCAAAAATTGAATTCCAATTGACAAATTACAAAATTACGTTGACGGTAATATCCGTAAGAAAATGCAGATATTTTACGTCAGAAACGACAAATGCA

>scaffold70.1.1508324

TTTCAAATATATCTACTAAAAGATTTATTTTAAACTTAGATACACAACAATGGAAAATTCTCGCATTGGAGTAGCGTGGAAAGAAGCCAAGACGTTTCCA**[T/C]**GTGGAGATAAACGATAGATCCTATCCAAACTCCATCGATTTCGCGATATCTATTCAATTTAAATCGAGAACTCGCGATATAACGATCGAACTCGAACTTT

>scaffold70.1.1669568

ACTTGTTCATCAATCACGAGAATGAGAGAACAATCTTCTGTTAAACATTAAAAAATGAAAATTTATCAAAAATAAAGACATTGGTGTATATTTATAAATA**[T/C]**GGAAAGAAGTAAGTATTTCAAAAAAGAACAAATTTAAAAAAAAATGAAATTTATCAGAGACTTAATAAACTTATTTTAAATAATTACTTATTAAAATGCT

>scaffold70.1.2447067

TTTCTTATTTCATTTATTCAAAACTTTAGAGATAAATATAGTATATAATTGCTTTTTTTTTTGAATTTTATTATGCTTAATAAGATTTAATATTATCTCA**[T/C]**GAAGATAAAATTCTTTTGAAATTATGTATTTTTTAAAATAAACTGTATAATTATCTCACAAAATTGAAAGTAATTGAAATAGCTTAATTGAAGTAATTGA

>scaffold70.1.349282

TTGTTACTTTTACTTGAATATCAGACGAAGATTTCATCGTGAACGCGGAACTTGGAATTTTAAATTGGAACGTGTTAGGTTAAGACTGACTACGTAATTG**[T/C]**GATATTGATCGAAGTAAAGTTTAAATTTGACGATACCCCCAGTATTGTCTCACGAATAATTTATCATTTAAAAATAGTGGTTAATCCACGAGTTACAGGT

>scaffold70.1.3741351

GAAGCAATCATACGGTTGTACTTACATCAAAGGGGGAAGTGTTTACATTTGGAGCTTATCAAGTTTGTATTAGTATTAAAAAAAATCCATTATTTGATAG**[T/C]**GATGATAATTAAATAAAATAATTAATATTTTTATATTTTTCTCGTCCTCGTTAGAAAGGGCAATTGGGCATAAATTGGTGGAATGGACAAAATGAACTTT

>scaffold71.1.1441022

TTTTAATCATTTGTTTTTAATCATTGACTTATTGTCGATCATTGGGGATCTGTAAGATGAGTTCTGAATCATTTATTTGATTTATTTGATTATTAAAATT**[A/G]**TCATCTAAGCAATCAACAATAATGTTTTTTAAATCTTAATATTAATCTTAATATTAATTGTGAACAATATAGTTTAAAATTTTAATTGTTGTTATTATAT

>scaffold71.1.1464055

TTTTTTACAAAAAAAAAAATAAAAAATATTATAATAATGCACCATACATTTATGCACATTTACACAAAAATCCATCATTTTGATCGAATTTTTCCAACGT**[A/G]**CTTGAAAAAAATTGGCATATATTGGCATAAATACTGGAGAACACTTTCAAAAACCTTCCAAAGATAGCCAGAGTTAATTATCAATAGAGTGTGACCGTTT

>scaffold71.1.1628873

AATTCTATTTTTATCTTATCTTTAGCAGACAATAGTGAAATTTGTCGATAATCGATTATACATAGAAAATGACCGTTTATTAGAAAGACATTTTGATGTC**[A/G]**TCAGAATCTAATGATTATTTATATTACTTGTTGAGTATTATATTATTTTTGTTTCAATATCTAATTTTTTTTTTTTACTAAAGATTGAATTTAAAATTTT

>scaffold71.1.1780129

TTAAAATTTTTGTATTTATTTTTATACTTCTAGAATTAATTCTTCAAAGATTATTTACATATTTATAAACGTATCTACCATAAAATAATTAATTTATCTT**[A/G]**TTTGAATATTTACAATGTTGTTTGAATATTTAATTTTCTTACAATTAAAAATTAAGTCGATTGGTAGTTAGATTAAGTTTAAGCATATCGATTATTAAAT

>scaffold71.1.1796010

GAAGAAGCAGCGGATAAGGAGTAAGCAGAGAAACCGATCGTTGTCAGGGAAACGAAGGAAATCTGCATTTTGTCAAACTGTCGCTGGAAAGTTGGTGGCT**[A/G]**CGTCTGGAAAGGGGTGTGACAAGAGTGAAAGGTGAGGAGTTGGGGATAGAGGAAGAAGAGGAAGAAGGTGGAAAACAAAAGAGAAGAAAAAGACGAAGAG

>scaffold71.1.459251

CCATTAAATGTTTCCTGAAACAAGCTATGCAAATGGATTAGTATAATTCAATGATAAATAATTATTTAATAATTTTCCATTTCCAAAATTGAGCTGAATT**[A/G]**CATGAAACTATACAAATACAGTTTATTTCAATCTTTTAATAGTATTTTCAGTAACATTTTGTTTAGAGGATGCTGAAAAGAAAAATAATAACCTGAACAA

>scaffold71.1.53980

CGAGAGATTAGGACGAGTAAATATAGTCGTTGTACGTAATTGCATCTAAATTTTTCTTCCGTCTATATATTCGTTTTCTTGGATTGCGCATGAGAAAGTT**[A/G]**TCGCGAATTCGTTCGATCGAGACGGTGCACGATCTGTAAATCACCGGATAAATTCTGTTCGAGGTAACGATTATCCTATAAATTGAATTATAATCACTTT

>scaffold71.1.785188

AATATATAAACATGTTTATAAGTCATTAATAATCACGTGGCGCTATCATGTTATCTTTGTTCATTATAGTTATTAATTGATTAAAATATTTCTAAAATTT**[A/G]**TCATCGTTAAATTTACATTTCTAAAAATTTCATCAAACCTTTCTATTTTTCAGTAATTTTCAGTTATGTCTGGTACTCTAACTCCTGTCTATTTATGGAA

>scaffold71.1.795727

TCCATATCAAATTCTCATAACACATAAAATTTTCGGTAATTATTTACGAAAAATCCATCAGATAATTCTCAAATAATATTGTGAAGATTTTACTTAATTC**[A/G]**TCATATTAAAAAGTTATTAAACAAAAACGTTTCCTTCTTCGAAATAATTGTAGATCAAAATAGTTTATTACTACGGACAATTAATGGCTACAATTAAGAA

>scaffold71.1.825071

ATTTTTGTATTTTTGATTTTTTTTCTAATAGAAATTTTACCGACGTATTTATGAAATTTCTAAATTGATTAAAATGAAACCAAATATAATTCATTTATTT**[A/G]**CTTAATTATTTAGATTATATCGCTATTTAAAATAAAGAAAATAATAAAATAAAGAAAAAATTTTGTTATCATTTATTGTTAGAACATTCAAATTACATCG

>scaffold71.1.1143705

TACAAGTGAATAAATATCACACTGATTATTACACGGAACATTGTTCCAGTTTGCCAGGCGTTACTTCATCCGACAATTATACATTAAATTGATGCAATCG**[C/T]**AATGACAATTTTTTTGAAATCGTCATCACGATTAAATAATTTTTCTTTTTTAACAATTAACATTCCTATTCTCCCTTCTTTATGCATTCGTATTCATTTC

>scaffold71.1.1194801

TATAAAAATATAAAATAATCATGAGCTTTAATTCATAACAATTTCAATATTGTTATAAAATTGATTAATTTTGTTTTTTAAAAATGTTCATTTTTCACTG**[C/T]**AATTGAATTACATATACATTATGTCTGTTAAATCAAAGATTGGTCATCAACTACATACATCGGCCATCTATACGAGAACAAATAAAACACATTTTAAATG

>scaffold71.1.1315577

TTCGTTATTAAATCACAGTTTAGAAAATATATATGTCGCATTTATCTCTGATTTTATCCGTTGACAGCGACGATTGTAAACAGTGACAATTAATGAAAAA**[C/T]**GAAGAGAGATTTAAACATTTAAATAAATTTATGTTTTTATCGGTATGAAATTACTCGTTTCACTGAATTCAAAAAAGATTTCATTGCTCATATCAAATGA

>scaffold71.1.1339274

ATTTGCATTAGTTTTAGAGAAATTGAAAAAAAATATTTTAAAGCCGTCCTCGATTTACATCAAAATATTCAAAAATTGGTCAATTTTTATTAACAATAGA**[C/T]**GAATAAAATAGAAAATAAAACATAAATTATTGATTTTTTATTGTTTCATTAATAAAAAAATGCAGTAATAAAGCAGATCTAAAAATCTGTATAAATCTGT

>scaffold71.1.1423811

GAGTTTCTGCATGGTTGAGAAAATATGCCTTTCAGCGTTTCTGTCTCTCTCTGCTGGAATGGGTGTTATAACGATCCTGCAATTGTCATAGAATCGATGG**[C/T]**ATGGTAGAATATGAACATTTTACGAGTTCTCTCTCTTTGCCAGTGATATCATAGTTTGTAAACAATGTCGTTGAACTTGTCGTAGAACTCGAAACTAGAG

>scaffold71.1.1430129

TTTATATAGATATAATATGGATTAAAATGTAACGAGTTTGTAAATTAAATTAATTAGAGACGATATGGATTTTGATGTTAATGAAGTGCAAAAGGATGAA**[C/T]**GATGATATTCTTCTCTTCCGTGTTTCTGAAACTTTAAATACTTTTGTTTTGATCATAGATGTATCAAAATAATATTCTGAGCTCTAGATATATTGTAATT

>scaffold71.1.1533516

TTTAATAAAGAAGAAATTCATCGAGCGAAACATCTTTCAATGGTATACGATTGTTTCGATTGAGGTTTATTTATTAAGTTATAAAAAATTGAAATTTAGA**[C/T]**GAAGTTCTATTTGTTTTTTTTCTAGAATTGATCCTTCAAAAATATATTTATATATTAATAAAAAAAATAATAATAATTTTAATAAGTGGTATATTAACCG

>scaffold71.1.1555544

ATTAATTAAAAAAAAAAAATGGCAACCAAAATTTTTTCTTTTCAAGTCCTACATTTTTCAACATACTTTTAAAACAATTTAATTATTTTTAATTGTTTCG**[C/T]**AATTTAATTCATTTCATTTTAATTTAATTTATTTTAATTTAATTACATAAGAGATACAACTTTACCGATAATATATATATTTGTATTTATTTAAATTTTG

>scaffold71.1.1594367

TTAAAAAATGTCGATTTAAGTTTGACAAGTATCAATAACGTAATGTTAATTGTCTTATTAATTATTCACCAATTATTTGCCACATTTTTGTGAGTAATAA**[C/T]**GCTTGCTGGGCTACTTGGAAATCAGTTTTATCGCGAGATATCATTCGTGTCGGCAGACAGTTTGGAGACAATGGGGGATAAAACGCACGCCGCGAACAGG

>scaffold71.1.1597786

TTTTCTTTGAGTATTGAGTATTGATTATTGAGTAATTCAAATAACGTGGTAATTGTATACACTAATGTCGAATAATTATTGTGGGCTCGAGTAGTTATAA**[C/T]**GATAATCAAATGACCAATAGATTCGAGTAATTGAATCCAAATAAAATCCAAAAAATTTAATGTTGATTATGTAATATAATAATCGTATGAATCTTATTCT

>scaffold71.1.1623041

GACATAATGATCAGTCTTATGACTATATTACGATAATATAGTTGAATTAAAGATAAAAATCTTCAATAAAAAAAAGTATAGCGATAGCAGAAATTATTTG**[C/T]**AAATTTTCAAAAATTATTATGTAGACAATGTCATTATTATTGGAAAATATCTTCTTATTAAATCATTATATATTGGTTTTGAAGCTTCGTATATATATCG

>scaffold71.1.1658744

TTGTTAAAAGTTACAACTAAATCTATACATAGAATCCTATTATATAGAAATAATTCAACTTTTTAACTGCCATTCCAGATAGAAGACAGAAAACGATTGA**[C/T]**AAGAATTAGAACGAATTGATAAACGAAAAGATTAATCAAACTTGCCTCTTTATAGAAGGAATCAGATAGTTATACTTCATAGCAGAAATAATGTTAATGA

>scaffold71.1.1663931

ATTCGAAAACTTGATTCTTGAAACGAAGTTCAAATTATGAATAAATTGATTTACATAATTATAAAATATTCAACTGGAAAATATCTTTCTTGATACATCA**[C/T]**GAAATTCAACATGTGAATAATATCAATAATAATAGATGATATAAAATATTGTAAATAATATTAATGTAATAATAAAAGATTATAATTTTAAAAAAAGTAA

>scaffold71.1.1668807

CTGTTTGAAAATTAACAAATTGAAAATTAATTAGATTTTTATTATATATAAGATGTATAAAATTAAACGATAAAATATATAGAAAGATAGATTTATACAA**[C/T]**AAGAAACGAAAAAACTTTTGAGACAATCTAAATATCTCTCGAAGATTTTTCGACATTACGATAATTGAGACAAATATTAAAAACTTCACATTTATAAATT

>scaffold71.1.667282

TGTCGAAATCTTAAGAAAATAAATTTACTATTCATTATCTCTATATTATATAATAAATCTCTTAAGAAAAATTTCCTGAGAAACGGAGAATTTAGGATTG**[C/T]**AAAAACAATCGATGGAATTAAATAAATTATAATTATGAAATCATAATGGAAATTTATGCAGAAGTGCTTATCTGTTAATTTAAATGTTATACATGTATAT

>scaffold71.1.718802

GAGCACCAACCACTTCAGAGACAAAATATGGTTAGTAATTTTTTAATTTTTTTTCTTTTCTTACAGCCGAACTAGTATTATTTGCAGTCTTTTTTTCAGA**[C/T]**GAGAAAAAATACATGACGTTATATTATCCCAGTTATTATAAATTTGACAGACTCCATATTTGTCTTTGGCCATGTAATAATTATAGTGGAAATAATATAA

>scaffold71.1.750497

AGACAGAACAAAGGAATAATAGAATATAATAAGCAAAATATTGTATTGTTTTCATTTAATTAGATTTAGATTATAACCTATCATATTAGAATTTTTTAAA**[C/T]**GCACTTTAAAATACTTCCGTGTCAACATAAACGCAAATTTTGAAAAAACACAATTCATTTTATCTTTAGATACAGGTAAAATATAGGTCATTACTACTTA

>scaffold71.1.1228562

TATGTCTTCTTTATCTTTTTTTATCTTCTTTGTATATTTATATTCCTTTTTATAATCTTGACTAATTTCCTGGTTATATCGGTTTATTATCTAGACATTA**[G/C]**TTCAAGTTCAAGGTTTAGAGATATTACAAATGTAGGTGTATAAACCAAATTTTATTTGCAAAATTGCTCTTAAAATGATATCACAATTGCCTCCAGCGAA

>scaffold71.1.1235724

GTATGACATAATTTGAACGTATCGAAATAAAAATTTATGCAGTATAAAAAGTACGCATCGTTCATTTATCGTTAATTCTATAAATTGTTTTTTTTATTTC**[G/A]**TGATTATTATAATTTTTAAACTGCTATTCTTCCATATTATGTTGAATTAAACGGAATAATATATAAATGATAGTCAAAGATATAGTATTGGAAGAATAAA

>scaffold71.1.1332739

CCGGTATCAGCTAATTTGTTTTGTAACAGAAACGAGAGAAATTTTGGGATGATTTAAAAGTGATGCAACATCCACTGTCCATGCAATAGTTTTACCGTAC**[G/A]**TTATAACGATTAAAAAATTTTAATTTTTAATTAAAAACAGTTAATGTTTCGAAACATTTACATAGTAAATGTAAGCCCAATCCCAATATAGATAAATATA

>scaffold71.1.1396891

TCTATCTCTCTCATATTATTGTTTTCTATCTTGAATCTCTTATAACTTAATAAATAAAATTTGCATTTTTGCATTTATCATAACTTTCATACTCATTTTC**[G/A]**TTTTTAATATCAATTGTATTCGACCAAAAATTCAAAAGTAACTTTCCTCCTTTCTCCTCTTTCGACTTTTAATTCCACGAACGAATCCATTCACGGACGG

>scaffold71.1.1523479

AGTTTTCATTGTATAAAATTAGGGATCTCGTATTTTTGTTAGTTTGTACAAACTTTTTGTAACTTTCCATTTCATTCTTCCGTTTTCTTTTTCGTTTAAT**[G/A]**CCAATGATTTGATGATAAATTTTGTGTAAATTTTGTCACTTGTACAGCACTCTTTCTAATTTCTTGGACATTTTGTTTAAAAACATTAATTTAATTTTAT

>scaffold71.1.1628847

GGCTGCTGCATTTGCCAGAGACATGAAATTCTATTTTTATCTTATCTTTAGCAGACAATAGTGAAATTTGTCGATAATCGATTATACATAGAAAATGACC**[G/A]**TTTATTAGAAAGACATTTTGATGTCATCAGAATCTAATGATTATTTATATTACTTGTTGAGTATTATATTATTTTTGTTTCAATATCTAATTTTTTTTTT

>scaffold71.1.1782806

TAGAGATTTTTTTTAAAGAATCGATTCTAAAAACTTAAACAAAACTTGATACGAACAAATATTGTCTTATTTTTTTACATTTAAATCATCTTAAATTTGC**[G/A]**TTAAAAATATATTAAATAGATTTATCTTATTCTATCTTCTCGTTTCGTCTAACACAAATTTCAATTACTTGTAATTTAATCAATAAGCCTCAATCGATAT

>scaffold71.1.231341

TGCGACCGATTTTCTCCGTTCTCGACGAGTGAGAAGCCGCCGATCGGCCAATTAATTTCGACCGTCAATTTGGAGAAAAAGAAATGGCTCTTCTTTTGGC**[G/A]**CTCGATTTTCGAACGCGAACTTTGTTTTTTTCTGACCTCCTTCTCGCTTTTTCCTCTGTTTCGAATGTCAAACACGTAGCGTGAAACAGTGCACGAAATT

>scaffold71.1.268985

AAGATAATAGGATCAACTTAACTTAAGATATAAATATAATTTTGAAAACTTTAAATTGTTAGTAATTAAATGGTAATATCTTAGTGTTATTGTAAATCCA**[G/T]**AAGATACTAAAATAAATGAATTTAATTGATACGAAAAAATGTACAAAGTCCTTCTTTCTTGACTATAAAAGTGCTGCGGATAACTGTCGCAAGATCAAGA

>scaffold71.1.366683

TGGAAAAATTTCTCGAGCGACCTCCTTGTTATATTCTTCTAATAGAAAGTCAAAAATTTCAATTTAAGTTTAAGAATTTTAAAAATTCCAAGAATTTGAC**[G/A]**TCATTTTTAAGAATTGACAAAATTCCTAAAAAAATTTCTCGAGCGATCTCCTGTCAATGTTATATTCTCCTGATACAAAGTCAAAATTTTAGGAAATTTT

>scaffold71.1.454790

CATAATTGGTAGTATCATCATATTACGAAGTAATTTTATTTATCTTGATTTAATTTATAAATCTAATTTTTCAAGTATTATAACGATTCATTAGGAAAAC**[G/A]**TGCAGTTTATACCAACCATTTACGACAATCAACGATGTTAGGTTAGATTCAGTTTTAACATAACTAATAAAGTCAAACTTGATACTTTTTTTGCAATTAT

>scaffold71.1.492735

ATACTAATAACGTAAACTCATTCCCAGATTCGTCCTCGAATCGTTAATTTCTTTTATCAATCATGGCTAATTGTATCTTGATAAAGTTAATTCGGTATTT**[G/C]**AGACGAATAATCTCATAACTTTAGGAAAAACATACTAGACTTATTATTAAGAACTAAAACTAGATTATCAGAAACGAGAAATTTAATCGTCTCTGGATGA

>scaffold71.1.501178

AGAAAGACAGAGGATATATAAAAGTATTTTTTTGTAAAAAAGATAATAGAAATCAAATTTGAAATATATCTCATTAAAGAGTAAGTGAAAAATAAATTTA**[G/C]**TTAAATTTATTAATAATTTCAATAAGTTAAATCAATATGTTACAATTTTTAAATATGTTTTGAAAAATTGAAAATAAAAATTTTTGTTCACATATAAAAA

>scaffold71.1.510378

TTTAATGTATAGCGTTAGTGTCTAATAGTTAGTCCTTTAGTTATGCCCTGTTAATGATATTTATTGGTACAATTATTGTTTCTTTTCTTATTTTTGTGCC**[G/A]**TATCATTAAAATAATATAACTGACTCTATTAAAAATCAATTAGAAAATCATATATACTAGCACATAAATATGTATAATGTTGTATCCTGTTAGTACTAAT

>scaffold71.1.656253

AAATCTTAGAAGACAAAAATTAATAAAATTATTATCGAATGAAATAACAGATATTAAAATAATATTTATCTCTACACTTTATTATTCTCTAAAAAAAGTT**[G/A]**TTATTTTCTTTTTCTATATTATTTGTCAAAAGGAAACAATGAAGAATATTTTTCGTTTCAAGTTATTAATTAATTCCTTATAGAACGATAATAAATCAAG

>scaffold71.1.92484

TCAATCTGCAAAAATCCTCAAAACGGTTAAATTACAATAAAGAAAAAAGAAAAATAATCCCTTTCTTATTATACTCGAAGCAATTCTTGAAATCTCAAAT**[G/C]**AAATTTTTGAAAAAAATCTGTCGAATCGAATAATATCCTAGACAGGGATTCTTTGCATAGTTTTCTACGAGGCTTAGAGTACAAAAAGAGAGATGGAGCA

>scaffold71.1.1411889

AAGAAGAAAAGAAATGAAAATTATGAAAATTTAAGAAGGAGCACTATGATAGTGCAAGTGGAAATGAAATTGTAGACGATACCAGAAAATTCTAGGAGTA**[T/G]**CATTTCTATAATTTAAGGTATAACTATGATGGAAAATTTTTGATGTATGATTTATGAAACAATTTTATTATTTTGAAATTGAAGTAAGAAATAATTATAT

>scaffold71.1.1596688

GTCCGAATTTTTCGTGTAAAATTACTTATTTACTTATCTAAAGATATATCTAAAGATCATAATAAATCAATAAAGAAAAAATTTATAAAAAGCGATGAAA**[T/C]**GAGTTTCGAGACTCGACAACGGTCTCTGATTCGCCTTGCTTTCTGATGATCACGATTCGACGAATGTTCCGTCCTTGATGGAACGTTGTTTGACGAGATA

>scaffold71.1.230117

CGATCTTTCGTGGATATCGACAAAATGGCTGGCCGATCTGGCAGTATCGTAATAATACATGTTTTTATCGTGTTTTTATCGCGTTTGCGGAAAATGTAGG**[T/C]**GAATAAATAGAATCTTATCGCTGTAAAAGAAAATTACTGGAAAAAATCTATTATCGTAACAAAGTTCGAAATCTACCAATCTGTAGTCGATCTAATCAAT

>scaffold71.1.488664

TCATATATATTTGCATAAAAATCTGCAATTTAGTTTAGATAAACGTGCTAATCATCACTAAAAATTAACGTCTAATCTAACAAATATTATTGAGAATAAA**[T/C]**AATGATATTATTATAGAATGATATTAAAATATGTAATTAAGAATAATATTGCCAAATAATAAATACAATTATTGATCCTATGACATACGTCATCATCCAA

>scaffold71.1.577716

ATAATTTTTATTGTTTCAGGTGTATTTTAGGTAATTTAATCAAGAAAATTTCTATCCAATAAATAACCAAATTTACAAATTATACAAGTTATATATTTTG**[T/C]**AAATTTCTTGATGATATTTAGTCGGTATCCAAAGAGAAATCGTGTTCGGGATCGGGCAATGAATGCAGTGCGCGAGGAAAGGGAAATAGTTATTTTTTAA

>scaffold71.1.747576

AAACCATTCATGAAATCAGAATATGGAGAAAATAATGTCCAATATAAAGGGTAAATATAAAAAAAAATTTTTTAATTTTATAAAATAATTTTCTATTAAA**[T/G]**AATACGTACTTTTTATTAAGAAATTCAGATATCCAGAATATCCAGATTATTTCAAGTTGTCTCCGTCATCTTGCTTTTACCTGAAAATTTAAATTGCTTA

>scaffold72.1.1152578

TATAATAAATCTTATTACTCCTTCTTTTCCCGATTTCAAGATATTGTCAGATTTATACATGTCCGACATCTCAAAAACTTTCTTCTAAATGATTTGATTT**[A/G]**CCATAAGAAATTATAAGTTTGAAAATATTAATTTTTCGATCTACGTTTATTAAAACTCTTTAAATACTATCTTCCTGTTTAGATTAGAAATTGAAATCTT

>scaffold72.1.1339939

ATCGTAACTTGTATCATGGAAATAAAATATAATTAAAAACTAGGATTAATATACATCGAGGATTATTTAGTTATTTTTTCGATATTCATATTTCAGTTTT**[A/C]**TTTCGATAAGTGTTCTTGTAAATTTTAATTTGATAAAAATTATAAAATGATAGAATCAAATTTATTTATTATAGATTTATGTATATTCAATGTAGCGATA

>scaffold72.1.1355183

ATCTGAAATTTTGAAAATAATAAATAGTTACGAATAAACCATCAGTGGTACGATATATTTAAGATCAATCGGAAACAAATTCCTTATCAAATGGAATGTC**[A/G]**TTAATTATTCGTATTAAAGTTTCGACAAATAGGACAAGAATATTTATTTAAGAAATAAAAAAACAGATTAATAGTTCGATCTAATCTAACTCTGAACCTG

>scaffold72.1.1407836

GATAAATTCTTTGATATCTTTTGATACCTTTTATAAATAAATAAAAATTCGAATATTATTTACGAATGATTCGAATCAAAAAATTAAATGGAAAATAATC**[A/G]**TATAAATTTTTACATGTTTAAAGAAAAGATTATTGCAATTAAATCAATTAAACGTAAAATTGAAATTAATATGCATATGATAGACGTTTTCGTCGACAAT

>scaffold72.1.172562

ATTATTGAAACGAAGAGAATCGATCCAACAGTCAGAATCATAACTGATTCAAGATTATAATAGAACGATTATTCAAACTAATTAAGCGGGAAATCCGTTC**[A/T]**GATAAAAAATTTTCAGATATGAATACTTTGAAAATATTTATTATTATCTTTATAATTCTTCTCGAATTAGTTATATCTTATTTATATACAGATACTTGGT

>scaffold72.1.403161

AATTTTCACAATCTATTCATGGTTATAATGGTTAATATTTTTCCACTTTTGAACACTATTCTCTCCTCTGAATAATTCCTTGAAGTGACACAGATACGTC**[A/G]**TATCGTTCTCTCTATGTCACAGGCACGCATTACGAATTCTCTGTCGCGTAAAGGCATAAGAACGTTATTTTTTCATTCGCCTAGCTTCAGTGCAAAACGA

>scaffold72.1.625676

ATGAATTTGTAAATGAAAAGTCGAACGTACCAGCAAAAGTTTCGTTATTTTATCTTCTAAAATTGCGTGTTATATATGATATAAAACAATGAATTACACG**[A/T]**CGTTTTCCTTAGAATACAAACAATTGTACGATTTAAAAATACTTAAAAAATACAATTGTTAAGTAATAAAGTTATATACTATCATCAGTTGGTAAGAATA

>scaffold72.1.887067

AAAAATATTTAATTCATTGTATTCATTATATATAATATATAAGCGCGATATTTACGAAGTTTCAGGACGATTCTTTTTAAGATTGTACTACTTAAATTTT**[A/G]**TCTAGTTGCCACAGTGTTATTACAATTAACTATTAAAATTAATTATTACAATCTGAATTTAATTTATGCTAAATTCTAACGATATACATAACTATATGAA

>scaffold72.1.1004868

TGGATTTGATATCCTTTTAGTTTTACAATACATTTTAACTAGTTTAATATATTGAATTATTTTTTGTTCATAATATTTTATGTCTTATTTCTATTTTAAA**[C/T]**GAATTTATCATTTTTCTCAAGTATGTATTAACTTTATTTATTTTTTATTTATGATCTAATTTCTTGCAGAATTGTCATACAATTTGCAGTATATTTAAAT

>scaffold72.1.1252970

CAATATTACTATATACAATTTTATAAAAAAAATTAAACAAAACCAAAAAATTTGATCGAACTTTTGTCTTATTGAATATCGTACAAATAAAATGAAGGTA**[C/G]**TTCGTACTTGGAAATGTAAATTATGAAGACGAGAATGCGCACGTCTATTGTAAAAATATGGCTACGTAATCCGCCATTTTTATAAAGCGGGAAGACTCGA

>scaffold72.1.1275239

TAATAGATATTAAAATAAATCATGTTTCCAACAACGAGGACAATTATCCAAATTAAACATTTTTAATATTTCGACAAATAAATATTTATCGATTAAATCA**[C/T]**GCATTCTCGTCGGTCATTGGAATATTTTTCACCAAGATCACCAAGCTCGAATCGAGGCGTAAAATTGAAAATCTGTACCCGTTTATCATTCGTCGGCCGG

>scaffold72.1.1410340

ACGAGAACTTTGTACGTCCGCTTTCAAACGTTCTTCCAATTCCTCTCTTTCGTATTTTGAAGGTCGGGCTAAAATAATTCCAGTTTCATATATTTGAGAG**[C/T]**GAATTAGACTGCAAGTAATCGTACTAAAATTAGCGTTAATGATCGTGACCAGCCGATAAAATTTATCTATACATAATCTTGTACGTACAATTGACTAAAG

>scaffold72.1.1497121

ATAGAATCCTTACATCACATATAGCTTTTCTATTACACATTTCTTTAAATTTCTTCTTAAAATTTCTTTAAAATTATTACCTTTCTCAATATATCACAAG**[C/T]**AATCGATTAACTATAAACGATTATTAATCTACTTATAATTACCAAACAAATTTAATATATATTTATCGCAATTAAATTTTTATTACAATCATTAAAACAA

>scaffold72.1.716725

GTTAATTTGTAAAAGCACAGTTAATAATTAGTACAAAACATGAATATTGAAATTACGAATTCTTTTTTTGGTATCCACTCTATATATTATAAAATGGATT**[C/G]**TTAAAATTTCAATTTTTTTTCTTTCTTTCTTAAAATATAATTTTTTAATGCTACAAGAATAAAGAAAATTTTTACGCATATAAAATGTTCAAATTTTAAT

>scaffold72.1.74531

CACACAATGATGATAATGCAATTACATATTTACAATACATTCATTTCTTTCATATCTAATATGCATTTAATGTGGTCAACATATTTCCTGGACAGAATTT**[C/A]**GTTCTTTCTTCGTATTGGCGGTTTTTTGGTCAATGAATCGAATTCGTATATCGATTACAATCCAACAATATTTTCGTTTTTTTTTTTACGGTTTTGGCTA

>scaffold72.1.780211

AAATGGGTATTGAAATTTTAATGCGATTTAATATATACTGCATATATATTAAGATATGCAAATACGTCGATATGAAAGAAATATTTTAATATTTAATAGT**[C/G]**AATGGCGAGTTCATTCATAAACAGCAATGTGTAATGTAAAATTAGCATTTATACATATGTATGAATCTATCAGGGAATAAGAAAGTTAATATAAATAAAT

>scaffold72.1.91591

TTGTTTTCAGTACTTTTTTCGTATCTCGAGATATATATTTTTTTTTAATACATCTTTGAAGAAAGTCAATTGATTATTAATCTGATGAATAGAACAATTA**[C/T]**GATTCTATAGCGTATTTTGTGCTTGGAAAAATTTTTGAATTAGATTTATTAAAAAGATCTATTAAAAAAAAAATTGAAAGTGATTTCAAAATGTTGTAAA

>scaffold72.1.1033003

GATAATTAATTAATTAATAAATAACACATCGATCAAAATTGTAATTAATACTAACACACGTTCAAATACCGTTTTACGAAGTAAAATGGATGTCCACTTC**[G/A]**TTGTACTCCCATTGTATTTTCAAGCTTCTGTTTCTTTCATGGGAAATAGTGAGAATGTACTTTGTAGATAAAACAAAGAACGACAGGTTGGTGAAAGCAA

>scaffold72.1.120768

AAGCAATCAATTGAGTTTATAATTGACACGTATATTGTGCAATCTTTTGCATAAGAGAAACGATACAATTGAATTTACTTCGAACAAAAAGCATTTCACC**[G/A]**TTCTATTGCATGTTTATTTGCATGTGTGAACAAATACTCCTCAAGACGTTCTTATTCACTATATGTATCCTTATGTTATAATATCGATATATGTATAAAC

>scaffold72.1.1217464

TCACATGAGTAAAGACTAGTTGAATCTCGGAAAAACGGAAAAGACTATTGTCAATATTGGCCTATAGAATAAAAGTAATTGAAAAGTTATTCAAAAGAAA**[G/C]**AAACTGAAGTGATTTCATATGATTTTGTTATGATGCTTTATATTAAAAAATTTAATGTATATGATTTCAAAGTAATAGCATTATTAAAGATTAAGTGATT

>scaffold72.1.1341206

GAGCAATCCAATCTTATCATGCACCTGTTGCAATTTCAGTACTTTGTACAGTGTGAAACATCTTCTCTTGAACTTTCAATATCACTTTCAATTTCAAAAT**[G/A]**CACTTGTTGCAATTTCGAAACAGCTTGAGCTTCGACTTCATTTTGAGAAAATTTCTTCAAATGAATTTACTATAAATTTATATTACATAAAATCATATGC

>scaffold72.1.170928

ATTTAAAATTTTAAAATATTTTCTTTTTCAAAAAATGATGATCTACTTGTATTAAAATTATTACAATATTCTTGATTAATCAAATTATCCGACATTTTCT**[G/A]**TCTACTATTATTTCGTTCTTTCTAATTCTATAATTAATATTTTTCTTTCTTAATTATATTTATTGAAATTAACAATCGTTAGAGAGTGATTATATATACG

>scaffold72.1.389888

CATGATGTTTGACTTGGATCAAAGTCAACTTCGTAGCTTACAAAATGTACTCATAATGCAATTTTAACCGTATTTATTTTAATCAAATGATCAATTCTAT**[G/C]**ATTGTTATAGCGATTCATTAATTAACTTCATTATGGTAATTTATTAACAATGATAATTTTTATATCGTATTCTAATTATTATTAATTCTATATCGATAAT

>scaffold72.1.409504

TTTTTTCTTCGATAAAGTTATAAAAAAAAACATTCGTTTCAAAAGTTATAACGAATATATAATTTTTCCTAATTGAATTGCTCTTTTTTTTCAAATTCTC**[G/A]**TAATGTCAGTAATCGAAGACAAAAATTTCAAAAACGATGCAAGGAAAGTACGTTTTCTTAACTACGTAATCTATTTCCAAACTGTCGAGTCGGCTGTGTC

>scaffold72.1.496888

ATCAATTTATATTTACTATTAGAATATCCGCTGTACTGTAATTCTCTGTGCAAAAAAACTCTCCTCCAAGTGATTTCCAAATTCCAAATTCACCTCGAAC**[G/A]**TCCCTCGATCCTCGCATCGGAGAGAACGGTTGGAATGGAACGATAACAACAACGATGGAATGGATAACAAAAGGAGGGAGGGGGGAGAAAGAAAGAAAGA

>scaffold72.1.501887

TGTAAAAATTATCGACAATTGTCATTTTGAAATAAAGTAGAACATTATAATTATGTTTGAAATAATTTTTCGAATAATTCGATCTGGGATAATTAAATTT**[G/A]**TTACGAAGATATTTTAAAATTTAATTTTGTATCCAATTAAAGATCTTTTTCTTGAATTAATGAACATGATATTAGTATGTATTATGTATGACATTAATAT

>scaffold72.1.624938

ATGTACATGATTTCAACATTCTGAAATAAGTGCCGTGTAATTTGATACAATCTTCTTGCAAGAAGACATAATAATACATTTGGACATTCATTTGAACAAT**[G/A]**CTGTACACAATGATTCAAGTTTAATGTAGAAGTGGAACAATTGTTTTAATAAAAAAAACATCATAATTGAGTTTATATTTAGAATTTATATATTTTCTTA

>scaffold72.1.642299

GCTGAATCAATCAGTGAAAAAAGAAATATATGATTACGTTTAAAGAAGATGGAGAACTTTAAAATTTTGTACAAAAAAAGAATTATTATCGCCATGTTAT**[G/A]**CAACTTTCGTTAATAAAGATATTTAAATATTTCCTGTTTGAAAATTTCATCGAAATCGATCGACGAGGAAACGAACTACAAATGGATTATAATGGAAAGA

>scaffold72.1.836842

CAAGTAGAAAATAAAAATCGTGCCTTTATGTTTTTTGATCTTTCGATCAATTTAACTGGTTGATATTATATATGGTATATATAATACGCGTAATGTGTTC**[G/A]**TTGATGTTACGTCGCTGCAATTAAAAAAGAATTACGATTTTTAGAGAAAATCATTTCGCGAGACGATATTTTGAATCGTTATGAGAGCGAATGTAAGGAT

>scaffold72.1.1094497

ATAAATAATACATATAGTTAAAAATTTTTTAATTTATCGTTAATTTTCTTTTCATTTTGTTTCACATGATCGTTAATATTTTAATTAGTTGTGCACGATA**[T/C]**GAAAAAGAACATAAAATATTCAAAACATATTACTCATTATGGTACTTGAAAATCTTTTCTTTCCTTTAATCGAATATATTCATTCAAATTTTATTTTGCA

>scaffold72.1.1126311

AATGCAGAGAATATTGATTAATAAATGTCGCGAAAAATAAAGGGAAAAAGAACAGTTTTTGGTAAACTTTATAACAATTTTATATTTATTGAAGATTAAA**[T/C]**GGTTTAAAAATTGTATACACGGATCGATCGATATAAATTGTTCACATTTTAATACTTTATTATCCTTGATATTAAAAGTATAATATTCTAAGGATCGATT

>scaffold72.1.112851

CAAACAATAATAAAATTATTTTTCAATATTTCGAGTAGTATAGAATTCTATTTCTTGCACTACATTTATTAATTAAATAGATTGATAAACAAACAATTTG**[T/C]**AAAAAAATAAATCGCAAGAAAATTCTTTCTCACTAAAATAATGCTACTTATTATATACAGAAATATAAAGCATAATAGTAAAATATGTTACATTCGATTA

>scaffold72.1.1217520

TTGGCCTATAGAATAAAAGTAATTGAAAAGTTATTCAAAAGAAAGAAACTGAAGTGATTTCATATGATTTTGTTATGATGCTTTATATTAAAAAATTTAA**[T/C]**GTATATGATTTCAAAGTAATAGCATTATTAAAGATTAAGTGATTTTACGATTTGAACAATTTCGATCAATTTCGAAATTTCTAAAAATTATACAAATTAT

>scaffold72.1.1251441

ATGAAAGAAAGAATTAGATGCCGAAATAATCCGGATATCGTTAACCACTATAAAAAAAAGTATTGTAACCTTTTCTATTCTCGCCACTAAAAGAACGTTA**[T/C]**GCCAAACATTTTCTTACGAGTCCTCGCGAATTCATCCATTTTTACATAGAACGTTAATTTAATTCTATAATAGCAATGTTTAAATTATGGGCCTACTAGA

>scaffold72.1.232428

ACAAAATGTAGAGAGATAATTAGAGAAACACATTCTTTTCTATTATTTATAATTTTTAACGAACAAAAATTATCTTTTAATAATGTTTAAATTTCTTAAG**[T/C]**GAATTTGATTAAAAAGATTTTTTTATTTAGAAATGATTATTTTTACTGATTATTTATAAGTTTCTATTCACTAATTTTACTGATTATTTATAAGTTTCTA

>scaffold72.1.389383

ATGTTTTAAGTTATTTATATCATAAATGCAATATACAATGCATTGGAGTAAGATGCCGAATTGTATTTGAGAAGATAAATTTTTATAAATTAACATTTAA**[T/C]**AATACATCTTTTTATAATAATATACTAAATAACAAATAAAGATTAATTTAATATAAACATGAATATAAATTTTTCATAAATTTAATTAGAAAACCTTAGA

>scaffold72.1.446481

ACTAAGGAATTTTACTTATACATTAAAAATTTCCAAACTTTTATTTTTACGTTCTTAACTTCTAACTTTTTTTAATTTTAATTTTTATTCGAAGATGAAA**[T/C]**GGAAAAATTGTGTTCTTTCTATTCCACTCATTATTATTGTATATAAAATCATCTCCTACTCAATTATTCATTGCAAGACATTCTGTATATTTTAAGAGGA

>scaffold75.1.397292

TTTTTATCAACACCGATTTTAAATAGAGATTTATTTTATTCATTAATAAAATAATTTATGAATTTTTAATTACTTAAACGATACTTTTTACTTTTACTTG**[C/T]**AGAAAAGTAAAGCAAAGATTATTAGCAAAGATTGGATATGGAAAAATACATACTCATAAAAAATGAATCTAGATTTCAGAAGTGTCAATATTATATTCAC

>scaffold75.1.522353

TCTTAATATTTTGAACTTGAACTTATTTTTTGATATAATTTATTGATAAATTTATTTTTTTTTATATTTCAATTTAATTTAATTTCTGCAACACGATTAA**[C/T]**GTTATTCCATAAATTTCAGAAACGATATTCCCACCGGTATTCGGCAGAAGATTGCAAGCTCAAGTTGTGAAACGTGGAGAACGAGTCAATATGGAGGTAG

>scaffold75.1.105626

TAACTCGTATATTCCCGTCTCTTTTCACCATTCTACCTCTCTATTCGTTTGTTTCATTCATTGAAAAAGACGAGCATGTAATTTCAGTTTCGAATCTGAC**[G/A]**TCTGAATTTAACATAAATGATTTTTTGCTTGTATGATTATTCAGTCTGTTTTTCGATTCCATGTTGATTTTTTTAAAAATTAGACAAATTTTATTGACGT

>scaffold75.1.22808

TTCTTCTTTGTCTTATTATTGAATAAAGAACAAGAATGTAGAATTAAGTGTAATTAATTAAATATGTGATATTATTACGATATTTACTAATTACACTATT**[G/C]**AAATTACACGAAACAATTATATACATATTTCGAAAGATTGACAAATTCATGGTGTAAAAATTCGAATAATTATATTTTTAATTAAGGATGAGTTAAAATC

>scaffold75.1.281625

TTTATTAAGTTATAAACAAGTGAAATTTTCGTTATAAACGAAACGAGACAACGAAATAGAATAAAACAGCTCTATCTCCGACATACCATTGCATTATATC**[G/A]**TTTTGTTTCTTTGCCTCGCTTTATATAACACAAACTTGAATTGCTTATAATATAATAAATAAGACTGATATTTTTGAATACCAATTTTTGTATTTATTTT

>scaffold75.1.482872

ATTTTTATGGGTTTTTTGATTAATTTTTGACTGATTTTTGTGTAGAATAGTTTTGTGTGCTTTTTGCTAGTTTTTTCCCTCGTTTTAAGATTAATTATTT**[G/C]**AGTGTTATTATCTGTTGTTTTTTGAAATTGGATGAAATACGGATATTTTAAAGATTGAATTTCAATGTGTTTTATTTTCAAGATAAATCAGCAGTAATTT

>scaffold75.1.65822

TCTTTTCTAATTGATTAATTAAAATCTAATTCAATTTTAAAATAGATTTTAAAATTATTTTTCTCAATCAAATTATTTTGTAATGTTTCTCCTTCCTCTC**[G/A]**TCAACAGAGACACGAGATAATTCGTTTAATTTCATTTACTTACGTGACAAATAATTATTTTAATTATCCGTAAATAATTTGTCCGATGATTGAAATTATA

>scaffold75.1.73319

CTTCTTCTTATTGTTGTTATCTTATATTGGTAGGATAAGAGAATTATTGTGTTTCTTTTTTTTAGCATCAGTTTCTCTCAATTTTATACGATCTCGTTTT**[G/A]**TTTGAAACATTATATGCAAGACACGCGTGAATGTGAATTTAATCATCAAAGATATTTCTTTGCAAGTTTTATCGAAAAATATGTATTTTGTTTATTTATT

>scaffold76.1.283535

GATTTTTCATCCATATTAAAATTCTGCTGTAATAACCATTGATCAAATTTTTTTTTAAAATTTTTTCTTGTGATGTTTCAATTGTATTAATCGAGAAAAC**[A/G]**TTTCTTAAATGGAAATAATATTTAATAATTCGTAGTAACAAAATATTATATGTTGTAATACATTTCCAAAATCAATTCATAAAAATGACGAAATAACATA

>scaffold76.1.403976

CTGAACCAATTATTTCAATACAATTATTTTACGATAAATTTTTCTGCAATCATGAAAAGAAAAAAAGGATTACTTATTTGATTTATTTATCTTCTTTACT**[A/G]**TTATTTTCTATTTATTATACTATAAAAAAAATTATGTTTCAAAAAAGACTGGTCTAAATATAAAATTATAAAATATAAATATATTTCATATCTATCATAT

>scaffold76.1.420287

ATTATTTGATTATTCATTTTATTTAATCAAATTATTTTAGTTCTTATTTGTTTTATTATTATTATTTTTTTAGTAATCTTTCTAAATTATTTCCAATGAT**[A/G]**CAACCACTTATTGATGTGCTGAAAGAAAATCGAGATTTATGGGAAGAATCTAAAAATGTATTTAAAAAATATATGGAAAAAGGAATGAAAAGTTTAAATA

>scaffold76.1.423659

ATGTAAAAGGTAAGCAAATATATTAATAAATATATCTCAATCCCCTAATTTATTTCCTTTAAAATATGTTTGTATGTATTTTTATATTACTTTGATATTT**[A/G]**TTTAATATAATAATAAATTTATTGATATTAATTAAATTGTGAATTCAATTAAAATCAATAAAAAACAAATTATTATAATTATACATATACGTAATTGAAT

>scaffold76.1.506219

TTTGTTAAGCCAATTTTTGGGAAAATTAGAATTTTAATAAAAAATATATTTTAAATTGATATATTTCAAATTTTATTTTTTAAAATCAAAAACTATTAAT**[A/G]**CAAATTGATTAATTTTCACTATTAATCTAGAAAAGAAAGAGAAGATAATATTACAAAGAACAAATAAGATATTTCAATGTATTTAAAGGAAAGCTTTTTA

>scaffold76.1.822442

CGGGGAACGAGCAGCAATATGATGAAATCGAGTGAGTAAACGTTACTGGGATTAGTCAGACTTTGCGCCGCTGGTTTGGATTGTTGTTTAATTAGTATTC**[A/G]**TCGTTCAATATTTGTTAAACGATACGAGAAAAATTATAATAACGATTCTTCCTAATTTTTGAAATAAAAAAAAGATGAAATAATTTTGTTTGCAAATTTA

>scaffold76.1.482782

TTTGTTAATTTATAATTCTTAAAACAAATTAGTGGTTTTTAAAGATAATGTATAAGATAATATAATTAATTATCTTATACAAATTTTAATTATTACTTAG**[C/T]**ATAAAGTGAATTTAATTTCAAATTCAAATAGAATAATTGAAATTAGAATTTTTTTTAATCCGTTAAACAGAAACAAATTTTAAGACGATTCGAAACACTC

>scaffold76.1.725888

AATAAAAATATTCGATATTATTTCACAAATGAATTCATATAGCAAAAATAGCAAAAGATTTATTTAAAAAAAAAGAATTAGATCGTAATAATTGATAATA**[C/T]**GATTATTCTCTTATTTTTCTCTATTTTTATACTGTTAAGAATACGTTGCATGCTTTCACTTTATCACAAAAAAAAATTATATTTCTGTAAGAAGTAGTCA

>scaffold76.1.730376

ATATAAATCAAAAAAAGTATGTCAACTAACAAAAATACCACAGAATTTGAAAATTTTAAATATTAATATATTAACATTAAACAAATTTAAATTGTTTTCA**[C/T]**GCTTTCTAATTTTTAATCAAACAAATGTTAAATAAAATAATTCAATTCAATTTTCTAATCTTGATTTTCTATTTCATTATTATGAAATTATAGCATTAAA

>scaffold76.1.45689

TTCGAACGGTTATTCGAGCAAGAGAAGGGGGGATTAAATATTTTTTTAAGAATCCTTGTTTGTAAACGTATTATTTTAAGAAAAGTCACCGAATCAGGCT**[G/A]**TTTTATCGCGTTTATATCGAGAGAACTTAGTTTGTATAGTTTTATTTCTTAATCTCATAATTGCTTCACGTTGTAGAGCGTTTCAAAATCCAAGGTTGAT

>scaffold76.1.62182

CTGTAAAGTTTCTTAAAAAGACACGCAGTCGATGCTTCTTCCTCTTATATACATTATACATTCTCACTTACTTTCGAGAACTTTCGGTAATCGCAATCTT**[G/A]**CTCAAAACCCTCTCTTCTCCCCCTCACCTCTTGGAGGTGGAGAAAAAGAAAGAAAAAAGGAAATCACAGAGAAGGTCAGTGAAACGTCAAATTCAGACAG

>scaffold76.1.644259

TAATTAGATTAATTATCACGATTATCATCTTGAAACGAATCGATATATCTCGATATCTCGATATATCTCGAAAATAAATCAAAATGAAGGCAAATAATTA**[G/C]**TTTGCTTTGTTACATATAGAAAACAATTTTTTTCGAATTTTTTAACGATTCTCGAAACGAATCGATATATCTCGATTTGAAAATAAATCAAAATAGAGGC

>scaffold76.1.720972

AGAATATTTCTTAATATAGTAAAAATTAAACTCGAGTGTTTGTTTCGATCGATTTCTAGAAAAAATAAAAAATTATGATCATTATAGAAATATTTCTTTC**[G/A]**TAATATCTTTGTTAAAAATTACAGAATCGATATTTCGATTGATAATTAAAGAATCGAAAGTGACGAATATTGATATTTTTTTAATATAATATTTTTTGTT

>scaffold76.1.728714

CATTTTCATCCATGATATATAATCTTAGATATTTTGCATGCTGTAAAAAATTAAAATTTTGTATTATATTTGTAATATTTGTAATATTTTTCACTAAGAA**[G/C]**TACAAGAATGCGTAAGATTAAAATGTCAAATTGAAACTCATAATTTAAAATATAAAAATCAACAATAACTTTAAATTACTTAAAAATTGTAAGTTTTTAA

>scaffold76.1.732243

GATTATTAATAAATATACAAAATGTTAATATATTCGTATAACACTAATAAAAACAAGTGTAAAAGTCGATATGTAAAAATCTTAATTAAAACTCGATAAA**[G/T]**AAATTTTCAACTGAAATTTTTATATATTAACTTTTGCATTTAGAATCAATCTTCCAAAAAAATTCCACAAATATATTAAAACACTTTACGTAAATGACGT

>scaffold76.1.85474

TTGATGTGCATCAATTAAAAACAACGTGAATACGTGTTAAATATTATTTTATTATGAAGAAACAAATTGAAAAATGAATTGTAATCTAACCTAATTTGTC**[G/A]**TAACCTATCTTAATATTATAAATTCGATTGTTTTTCACAAATTCAACATAAATCGTATCGTAACTATAATATTCATTTATCCAATGATTTATAGATAATC

>scaffold76.1.407273

AATTTGAAAAGATACAATATATTAAATTTTAATTTTTTATAATACTATGTTTTTTAAATAGAACCTCATATTTTTATTATATTCATTATAGATTTAATTC**[T/A]**GCAATAAAAACATATGCTTTTTTAAAAATATTGCATAATTGATGTTAAAATCTTTATAAATAAAAATTTTAAATAAAATGTTCTTTGAATCAATTTTTAA

>scaffold76.1.616240

TCTATCTATTTGAACAATATTTCTGATACAAATTTCAAAAAATTTTTAATGCACGATTTAATAATAAATAACCTAACCTAATCTAACTTAATTTTTAATA**[T/C]**GATCAACAAGAATATTAAGAATTTAACACAAGAGCATTTGTTTCAAACGAAAATATTATATTCTTCGAAATTATTCAAAATATAACAAAAAAAATATATT

>scaffold76.1.798584

AGAATTTTTAATGCTTTCAAATATATTTGCATTTCAAAATTTGTATTCTGGGAGATAGTGCATTCACGAGTAATTAGAAAAACATCAAAGCTAATAAGAA**[T/C]**AGATTCTCTGGAAATAATAATTACGAGAGACAATGTTTAATTAATTTCAAAATTATATCTTCATTTCAAAATTTATAAAACAATAGTTATAATAGTTTGA

>scaffold76.1.86338

AATATGCGCACGCAAATATCATTCACAAATGAATGTCGGAATGGAATTTCAAATGGAGGATCGAAAAATAGTTTAATAAAAATAGTACTATAATTAAAGA**[T/C]**AATATTAATTAATTTTTCCTTTGTAATTCGTTATCCTTTATGTTCATCTATTTATATTATTTATCTATTACAATTTATCTACTTACATTTAAACTTTTTA

>scaffold78.1.1131742

TTAACACCCCGTGTCCCATCATCTTTCAACTTTCGGTCTGCTTATTACACATGTGGATAACGTGAAAAAGAAAAAGAAAAGAAGAAAAAGAAATGAATTT**[A/G]**CATATCCAGGAAAAAGAAGGAAAGAAAAAAAAAATTAATCATCCTCAAACTTACATCCACTCGAATGAAAAACTCGGCGACAACTCGGCCGCGTTCGTTC

>scaffold78.1.1132750

GTCTCGAAAATAATATTCGTGCTTTCAATCGATTAATTTTATTTATTTATTCTTTTAAAAAATTCGGTTGATATGTGTGCAATTAATGTAATGTTTCCGG**[A/T]**GATTTTTTAGTAAAAAAAGAAAAAAAAATAGATGAAAATGCGCTGAATTTATTATTACAATGTGATTATATTGTTTAATTTCCTACGCAACGTACAACTT

>scaffold78.1.1506527

TACGTGTTGTAATTGTTTATTCCAATTTCGAAGATTTATATAAAGAGCGGAGATTTTTAACGTTTTCATTCAAATAATCCCCTCCAACTAATTTTCAAGG**[A/C]**TCAACACTTTATTCACTTTTCAATATACATCCAAACACTTCTCTCCCTGATACTTAAAAACAGAACAATATGGAAACCCGGCCATAAATCTTCGACCGGG

>scaffold78.1.1801547

AACACAGTACGGAAATTATGAATGGATCTGCGACCACGTGATCTATATCTTTTCTTGGAAATACATATTACATTTGCGTATTATGTTTTTCTAGTCTGTC**[A/G]**CTTGTCTGGAAATTGAGATAGGTCAGAAAAACGAGAGAGTAAGTACGAAGATGAAGATAGTAAAGTACTAAAAATTATCTTTTCCTTTACATGATGATTA

>scaffold78.1.196892

CATGTTGGGGTAAAATGTTGGAACATCGATTAATTAGAGATTCTTGGGCTAATTTTAAGCAACATTTTCCTTTAATTATTAACAAAAAACATTGCCAATC**[A/G]**TGCATAGAGTTTCGAAAAATATGCGTGGAAAATCCGTTGAATATAATATATTTATCGAAAAAAATTATCTTATTAAATATAGGACGAATAAATGGGACGA

>scaffold78.1.2375476

TTTTATCTCGGTGAATTAATTAGATAAGATAAATGATTAGGGATATATTATCACGTTGCCATCAGTAGTTTTATTTTATTCGAGTTTTGTTCGAATGTTT**[A/G]**TCGAATCGATTATTAAACGTGGATGATTAATAATTTATTTATTATCTTGTTTCGGTAAATTGGTTTCAATAGTGTATGAATTTTATCGATATTCGTTGAA

>scaffold78.1.2423617

CTTTCGTTAAAAAATTCTTTTGAAACCCTGCAACTTATTTGCATTTTTCTCAAACTTTCCTCCAAGATTCTTCTTATATCTTCATATATCTAAAAATATT**[A/G]**TTTCAATTTATAGATCTCGTACATAATACATACATTATATTCGCAATCTCTATTAGTTGTTCAACCTTCTAATCTTTCTTACAAACATTCCAAGAAGCTT

>scaffold78.1.436126

CGAAATTTTAATTAAAGTTGTAAATTAATTAAATTCTCTTGATTCTCCGATCAAATAATAAGAAACAGTTTTAAAAAACATTACCTTCGTGTAAAGTTTT**[A/G]**TCATTTACGAATAGAAACATGCATCATTCATTTCAATTCATCTTTTTATCCACTCCTTATGTATTTCACGATAATCCTGAAACCAACAATCCATGATATT

>scaffold78.1.634890

CGAGTTTCTCGATCGAAATATTTATTACAACACAACCGCATCAACTTCTTTTTTCAGATTTTACACGATAGTCGACTTTCTGAAATTAATCAAAGACAGT**[A/G]**TTTGATTATTAATAAAACTTCCACCCCTCAATGGATATCCATTTTATTCCACTTTCGATTATGTTTCTCTCGAGTCAGCTTGGGAATGATATCGCGTACC

>scaffold78.1.799383

AATCTGAAATAATTTTTTTATCATTTGATGATGATGTAGATTGTAATGATGTTCTTGCATGTAATCTATTTTCATCTTTTTTTAATGAAACTTTTCCACT**[A/G]**CAATCTACTTCTCTTTCAATAATATTAGAATCTTTTTCTTTCATTTTATCAATATGTTTTTGATATAAAACAGTATATCTTTTTCGTTTTGGTGGTATGT

>scaffold78.1.1483678

AGCGAAATTAATTTATCCTATCTTATAAATAGATAGAAAATCAATGAGACACGAAATATTAGAGAGAAAGAAACAATCCTGGAAATAAATGTTAATCGAA**[C/T]**GATAAAAATTATAACGCGTTATAAATTGCGTGTAATCGTATTTTTTCTTTAACGATTAAAAATGCGGTTTTGCAGCTAACTTATTTCAATTATAGTTTCG

>scaffold78.1.1513173

AGAGTCGATATAGAGCCGAGGATTGAATTGTTTTTTACCTGTTTGGTATTATTTTATTATTACCGTTAAGCTTGTTTGTTGAAAAAATTCGATTATCCAG**[C/A]**TTTCAAGATTTATTTTATTTGTTTTTAATAAGTTTTAATAAGCTACATGGTTTACAAATGAATCTCAAGTTATTGGCTACCACGAAGAAAAAAAAAGAGA

>scaffold78.1.1531017

AGAAACGGATCGAGCAACCTGTCCACAATTGTCAATAACAATTTACATCCGTGTGTTATCTGAATTTTCGATTTCTATCTAAGAATCGTAACGAAAATAA**[C/T]**AAAGAGAATGTTAAAAGATCGAAGCGAATTCCAAATTGAAGACTGAGCTATATCCATAATTCGACCCCTCCGCCTCTGTTAAATATTAATAGAATCGAGA

>scaffold78.1.1739984

TCAATATTTCAATATATTATATTTCAAATGATAAGAAAAAGGATAAAATACATAACATTGAGATTTAGAAAATTACCTCTTGTTGATAGAATTGTAATAA**[C/T]**AAATTTAATATAGCGAAACTTCTTATATTTGTCATTTCTTAAAAGTTTCTTTGGTGTCTCGGTTTCTTTGCTCTGTTATCGACTTGGATACACAGAGTTA

>scaffold78.1.1877804

AGTATTGGATTGTACAATTTTTTTTGTGGAATATGAAATTAAAAAAATGAAATTTGTCAATCACGAAAATTTTCGTATATAAAAATATTGAGAAAATTTT**[C/G]**TAAATGTTAAGAATTTTTTTTTAGTGAAACAATCTATTGTAGAATATTACGGATTGTATTTTTTTGAAATATCGATATGAAGAAATAAAGTTTAAATTTT

>scaffold78.1.1942871

ATTAAGAATTCGTTTGAGAAAAATCCACGTCTTTGAATAATAAATTGATCTTCGACGTCATACGTTACTTACATTACGATTATTCGTTACTACATGCAAA**[C/T]**GAGAAATAAAACGAAGATATTATAGTAGTAGAAATATAACATCTCATTGCTACTAACTTAATAAATTAAATTTGTTGCGAATTTAAATACTCAAGTCGAG

>scaffold78.1.2148826

TTCAATTATATAATCGAGATAAGTAAATGAGAAGTACGCGTCGAATAGATAATAAATATCTTGGATATTATGAAAAAAAATATTTAATCAAGACTGCAAA**[C/T]**AGTTCCCTATTCGTATTAAATAGATTATTTTACATAATATATTATATGTAAAAATTTATAAAACATTTATAGATAATAGATAGTATTGAATAAATTATAT

>scaffold78.1.282638

AGATATTTACATCTTTTTTTATATGATATTCTGGTTCTTGATTCGATCTTCCATTTCATTTCTTAATAGTTATTACCATGATCGTGCAATATTATACAAA**[C/G]**TTATCACGGTTAAATCATGAGTTGCCCTGTTATAGATATAAGGGTTAATAAAGGTTCTCGATTGCTCCTACATGCACGAGCTTTCATAAGTACACACTTT

>scaffold78.1.363654

AAAGGCAATCGTCGAACGAAGTTTGTTCTCTCTCTCTCTTTGAAAGATTTTGTATCGTTTCTGTCGAATCTCGGTTGCGCTCCAGAAAAGAGGGATAATT**[C/G]**TTTGGAATGGAAATAAAAATAAGAGAGAAAGGAAAATTGGAGATATTTATTCTTTTTTTGAATTATCGAGAGAAATCGGCACTATCGGTGCAAATTGGCA

>scaffold78.1.598211

TTTCCAAAATATATACAATTACAATTTATATTTCATTTTTATTTTAAAAATTTATAAAATTTATATTTAAGTCTTAATGAAATAGAATAAATAATATTTA**[C/T]**GTTCCTGTATAACATTAAATTTCTATATTAATTTCATAAATTTTTATTATCCTTGTAGCAAACATATTTCTTTGAGTGCAAAGGACTATATATACGTATA

>scaffold78.1.658240

TTCTAAATTATCTTCTAAACAGTTATAGGTTTTGAAAGAATAGACGATATTAATTTGTAAGTTTCGTCTCTTTTTTAAGTTTTTTAAATTTCTTCTAAAT**[C/A]**GAAATTACTTGATATTTTTGACCGACCAATTATAATATATTACTTAGGATATATTCAGATACCGTGATATTCAGAGCCACATCATTTTCTGCAAGTTTAA

>scaffold78.1.1024385

CGTAATCCGCAAACCATAAACTATCCTCGTTCTCGATATCTAAGTTTAACGTTCTACCGTATATTAAAAATTACGAAATTCCTTCGACGGTTATTACAAC**[G/A]**TTTACCATGGTAAGTTATCGTTTTTCCTTCGATTTGATTCTACTGTAAAACTTTGACACTATATTTTCCGAATGATCGGAATAATATATTATAAATATGT

>scaffold78.1.1676627

TATTGAAGATTCCATTGAAGATATTTCGGTGAAATATTCTAAATACATAGTTAAAGAGAGCAACATGCAACAATCTTATCTACAAAAGTTCATTGAATTT**[G/A]**TTCCAAATTATTTTGCATTTATTACATGTAACGAATATGAAAAATATAATATATAATAATAAGAAAAAAAAAAGAAACAAAATTTATCAAATTCACGTTA

>scaffold78.1.1805960

GCTTCAAAGCGAAAAAATAAAAAATAACATTATCATGAAAATATATAAGCAAATTAAAATAAAATAGATAATAAAAAATTACTAAAAAGTAATATAATTA**[G/C]**TATACAAAATCACAAGAAAATTATACTTCAACATTGCCTTCTAATAAAAATTCAAATTATAATCGATTGCATTATAATTTAAATGCAAGAAAATTCTTTA

>scaffold78.1.1826182

ACCCGCGGTAACCAGACAGAATATTGCCATCAGCCATGGCGTCATCTCGACGATGGCAGATGTTGATCTCTGTAAACAGAGAGTTAAATACAATTGGCGC**[G/A]**TCTTATGTTTAATTCAATAATCTTTACCGCTTGGATTAGTTAATGATAAAAAAACTAATGTCGCATCGATCAACATTGGCGCTTATAAATTACGAAAGAA

>scaffold78.1.1970666

AAAACTAGATCGCATTTCAAGTTTTACGTTTCCAGTTTTGCGTATGATCCACTTTCACTCGTTGCTTTTCCTCCGTATACTTAACGTAACTAAGCTAATC**[G/A]**TCTTGACTATTTAATAACTCAGAAATATATTTTGTAATTTGTTAAATTTCTTTGAAATAAATCAGATTAGCCTCTTCATTGTTCTTGAAATTTTACATCT

>scaffold78.1.2357560

ATTTGGAAAGTGAAAGGGGGAGGGGGATTGTCGAAAACGAAAATTATTAAGGAGGTATATAGAATTTGGTAGAATCTGAGAAAGAAAGGTAATGAAGAAA**[G/C]**AAGGGAAGAAAGTGTTGAGGATAATGGCAATCCAGCTGCGAATACTTCAGACGACTCGTTCTTCTCGTTAAACTTACTTGTACTTGGTCATCTCACGCTG

>scaffold78.1.2369405

TTTCAATAATGTCAAAACTAATACAAAAATAGGTCATCCTGGTTATTCCAGAAAATCATTTCCAAAAATGGTTTTGAATATCCAAGATGCTGGCATTGAT**[G/A]**CAGCTTTTCAAGAATAGAATTTTGAAAATAATCATAGCGATATTCATTCACATTCAATGAAATGAACATGTAATAATTTTGTCAGAAAAAATTCAACGGG

>scaffold78.1.2433935

TAAAACCGAATACAAGAACAAAAGAAAACAGGAACGCTCCAACCAAACTCTCGTAACTCTCGAATATCTTAACTTTCCAGTTAATTCTAGAATAATTAAC**[G/A]**TGCCAGTTAAATCCTTCGATACGACGAGCTGATAATTCATATATTATATTGTATTGATGAAAAAAAAATACCAAACAAAAAAAAAGGAAAAACATTTATC

>scaffold78.1.402073

TGTGACTCTCACAATCTTTCTCGCATACTACACAAAAGAATGGTCCACTACTTGGCACATGATTATCTATAGTATGCAACAAAACACCAGTCAACTTATC**[G/A]**TAATTTTGCAAACATAGAGTACATTGATACTGTATTACATCATCTTCAAGTTTGATTTCTTTTATTCTTGTTATTGTATCATTCAAGCTACCTAATATAT

>scaffold78.1.842159

GCTGTACACCACGGTCGCGCGGTTTCAAATCGATTCGTGGAACGATATAATGTATATATATATATATATCTAGATCTGTATCCGTGAAATTTTTATTTCT**[G/A]**CACGAACGAGATTCCGTATTGAGACGAATTAAAAAAGATTTATCGATATTTTTGTAAGAAGGAAAAAAAAGGAAGAAAAACAAATCGATAGAGATCCAAG

>scaffold78.1.1127330

GAAAGCAAAAGCCAGAAACAATTAATCCGTGAAATCGTAAAAGTTAATGTAATACTCGATGGGGCGTTTCCTATATTTCAAAAAAAAAAAGAAAAAAAAA**[T/C]**AATCGTTTCACGATAAATTTATATGTAACAGCGAGAAACATTACATCATTTACATCGTTTTATTAAAAAATACGTATATTTTCTTTTTTCTAGAAAAGTA

>scaffold78.1.1966989

GTCAAAATATGACAAAAATAATAAAGTGAAATATTAGTCAAAGATACGTCTTCAATATTAACCTCAATATTAATTAACACGGAATTGATATTTTTAAAAA**[T/C]**GTTAAATAATGAAGTGTTATTAATTTAATATTATTTCAGGTTTTACTATTAATAGCTCTTCCTGCGTGCTTGGCACAATTTACGATACAGGGACCCAGTG

>scaffold78.1.2420834

AATATAAATATTTTCTTCTCTGAAAACGAATAAAAATGTGTAATTCTTTACGTAAACAGTTTCTTATTTAAATGAACGCTATCATAATTCAACGTTTCAG**[T/A]**CGAATCATGGAGAATTCTATATTTTATAATTTAAAATACAATTAACATGTACGATGAGTGAACATTGATAAAATATGATTTTATTCGATGATATTCGTTG

>scaffold78.1.524961

ATGTGCGATGCTTTAATAACTAAACAAGTATGTTGTTTAATACACTATTTTCAAGTATAAGAAATTTAAAATAATCATACTATTACATTTTATTCATTGA**[T/C]**AAATTTTTTAATGAATAATATTAATGATATATTTTAGCAAAGAAAAAATTAACGATGATTATAAAAAAATTTGTTTTTCACAATAATATCAAAGATTTTT

>scaffold78.1.663093

AGTACTCTCCATCCGGATTTATACATCCCTTACGATTTTATTAGCGATAATACTAGATACCGTAGCAATAATTTTTCTAAAGAATCACGCTGTTCAAAGG**[T/C]**ATTGAAGAAAAAATTATCACTTATTCTCTTCGCAAGCACGTAAGAACTCGATGAATATCGATAATGCTTATCCAATTGACAAAAAGTTTGCCCATTCTGA

>scaffold78.1.924511

AAAAAGAAAAAAAAGTAGAAAATTATCCCCAATTACATATTTCATTTATAACGTTAGAAAACGCTGGCATATTTTATGAAAAAAACGTTAAATGATCAAA**[T/C]**GTACTCGTAGAAAACATCGTTTTGAAATTTTAGATTTAGTTTTCGTCCTTGAATTTTTCTTTTTTTTATATTAGTCCAAATTCGTGTTTGTCAAAGTAGG

>scaffold79.1.814004

CGTAGAGAATAAAGTTTGTCAAATTTTTAAAATAGGAGTTTGAGTTTGTATTATAATATGAATGATGTACAAAATCGTTTTGGTAATTCATAAGTAAAGG**[A/C]**TTGATATTTTTATCTTATATCTTAGTTTATTTATTTTCTTCTTTACTCAAATATATATGTAAAATAAAAAAATGAAATAACGCACGTTCAAAGGTAAAAC

>scaffold79.1.792645

ATTTACTTTAGTCCATTTTGTGATTTTTGAAAAAAAGAAAAGATAAATTAAATCGTATTAATCGTAATTTTAAAATATTAACGAAGATTTACAATATTCA**[C/T]**GTTTATGATTATGGTATAATATGGCATTAAAATGGTAAATACAAATATGAGATACTTGAGCAATTAAAATTTTTTGAAGATTTTAAATTATAAAAGTAAT

>scaffold79.1.802540

AGATTGAATTTAATCGAATTGAGAATTAAAATAATAATCTATTTCGAGAAGAATAAGATGATCCCGATTTCGAAGATTATTAATCGGAAATTATTAAAAT**[C/G]**TTTGAGAATAAAGCGAGAGTTAAATCGATACAATTTTATTTGTAATTAAATTCGGGAACGAAATGAATAATCTTTTCCATGAACGTAAAATTAATCAAAA

>scaffold79.1.1027946

CTACTCGAAAATCGATTTTATCATTTTTATTCTCTGAGAATTTCTAAATTTTACGGCATCAAAACGTATAATTATTGATATTTAATTTTTTTTCAGAAAT**[G/C]**ATAAACTTTTAAGCAATTAATCAGTTCACCTCTTGGGTAACGTAAATATCAAAATATATATATATATAAAATGAACGTTAATAAAGATATATTTATCCTT

>scaffold79.1.340160

CGTTTTCTGATGGGACATAATAATTACATAATAAAATCGCGCTTAGTATCCTCAGTTGTGATACTCTTAAAGGAAAATTCCTCAAACTAAATTGATTAAC**[G/A]**TTAGGATTATATCGCGCTCTAGCCAAGCTATCTCAAAAAAATATAATTAATGAATGTCAATTAATCAAAAATAATGTCTTAACAAGCAACATCATTTTAA

>scaffold79.1.605787

TTGACTGTTATTCAGGATATTATTTTTGTTCAACGTTTTTTTTTTTTTGAAATAATTAACATTGATGACAAAGGAGAAAGATGTATTATGATCAAACAAC**[G/A]**TAATGTATTCATATAATTTTTTTGTGATTATATTATTTTTTAAATTTCAAAGTGATCTTCAATTTTTCAAATGGAATATTATAAATTCTTTTATACAGTG

>scaffold79.1.607206

GTTCATTTTTTTTTTAGTTTGGAAAAAGTATTCTACGATATTTAATTAGAAATGAATTTATTGTTTTAAATATTTTTTTTTTTTCATATTCGTTATGCTC**[G/A]**TTTTCATTAAATAATTTATAGAATTTGATAGAACGATTTATATGTATGTTTATACTCTAAATAGACATTCCAATTCAATTATCTTTGTACAAAACAATGC

>scaffold79.1.655362

AAATAATAAAATATAAATGTTCCAGAGAAAACGAATAAAAATATATTTTTTCTGCATTATATCATTAAAAGCAATATATAATATTTAAAGAATAATATTT**[G/A]**CATATTCAGAATTTTTAAGTATAAAATCGAAAATCTGTCAAGTTGATGAGTTCCGTAAATTTAGTGTTAATGATTATAAAAATCATATTTACGATGAAAA

>scaffold79.1.659633

GTAGACCGTGGAACGATCGGCGAGAGTTTATTGAAATATCTGAAATTTGAGAATTTTTAGAATTCTGACGAAATTCGATGATTTGCACGAATTGAATTAA**[G/C]**TTTGAGCTAAAATTAATTTTTAGCAAGGTTCAATGATCTTTTATATATATATAAATGCTTTTTTGATGTATTAGTTGTTGCACGTTATTCATTAAAAATG

>scaffold79.1.664526

CTAATTTCAATGAAATATACATATAATACCTATTTAAATAAAAGAAATGTTTAAAATTTTTTCATAACAATTTTTAAAAATTTTTTATTCAGTAAACTAA**[G/C]**TTGTTTGATCTTTAAAAAAAGTTAATCAAAGTTCATTCTTAACAAAAATATATCCAAATCTTGAATATCAAGCAAAATCGAATGAGTCTTTTTAAAATAT

>scaffold79.1.727311

AGATACGTTCTTTTTTTAATATAGTTTATTTTATAGTATGTCATTTTTATTCTAACAAATGCTATCATAAAATCATCCCAATAATATATTTTATGAACAA**[G/T]**CGAAGAAAATACATTTGTGACAAAGAAAATATTATATATTGTTCTATTTTATAAATGATATAGCGTGAAACATGTCTTATAGGTGTGCATTAAATCAACG

>scaffold79.1.773661

ACTCGTCAAAGTGACGGTGTTTCAGATCGTTTTATCTTTTGCAGCTAATTCATATCCACCGTGTCACTTTAATGATAATTATTTATTTACAGTACAAATA**[G/C]**TATCGAGGATCGAAGAGATTGATAAAATGGTGGGATTTACTCTCGAAATTATTCGTCAAATTATTTTTCTCATCAATCTATACACAGAATGTTTTAATCC

>scaffold79.1.867157

TGGAAAAATCATTAGAAGCAAATTGTTTTATTCGATATATAAAAATTAGCGTTTCGATATAGAAATTAAATTAACTATAAAACTATTATCTGAACTAATT**[G/A]**CCGAGATAGTTCGAAGGATAATTATGTTATAACAAATTTATAACTGTAATTTTAAATATAATATACGTTATACAAAATACAATATAAATTTATAGATTAA

>scaffold79.1.900051

AAAATTGGAAAAAGTTATAATTGAAAAATCTCATAATCATTAAAAAACGAAATGCAATTCATTTGTAAGCACATTATTCCTTACATATAAAAATATTTAC**[G/A]**TAATTTGACAATCAAAAAAAAAATTTCGTTGTAAATATATATTATCTTCTTCAAAATATGAAATAATTCATATAAAAAAATCAGTGTTCCATTATCTCTT

>scaffold79.1.998812

ACATTAAAATTTCTAAATTCGACAAAATAACTGATTTTTTTACAATTATAAGAAATATATGAGTATTTTCAGCAAAAATTAATGCAATTTGTAATCAACT**[G/A]**TTTATATAGATTGGTAATTCTACAGTTAACAATAAAATTTTCATGCATATAGAATATGATCTATCATAAACATTTTCCTTTTACAATCAATAAATCTGAT

>scaffold79.1.1104224

ACAATAAGATTTCCAAAAATAATCACACAAACATCGGTTAAAGTTGGACCTGGAACATACGAAATATCGGATGTTACTTGTCCTGATAGGCATCTTGGTA**[T/C]**GAAATTAAATAACCTATAACACAACGTTTCAAGAATACGTAAAATAAAAATTATATAAAATATAAAATGAAATTATTTGTATAGCTGAATATACTCCATT

>scaffold79.1.813987

TTATAGTGATAATATAACGTAGAGAATAAAGTTTGTCAAATTTTTAAAATAGGAGTTTGAGTTTGTATTATAATATGAATGATGTACAAAATCGTTTTGG**[T/C]**AATTCATAAGTAAAGGATTGATATTTTTATCTTATATCTTAGTTTATTTATTTTCTTCTTTACTCAAATATATATGTAAAATAAAAAAATGAAATAACGC

>scaffold79.1.828684

GGGAATCGAGCTTTTTCATTTTTTTTTTTATTCTTGGCGAGTGAAAAGTTAAGTTAATTTGTCAGACTTCTCTTTCCAAAAGTATCAATTAGTAGAGAAA**[T/C]**AGTACATTCATGTATTCCAGAGATAATATTGGATTCTGAAACATTCGATAAACGCAATAAAGCTATTTTTGAGCTGATTTTAGAATATTTTTAGAAACTT

>scaffold81.1.109906

ATAATAATTTTTGGTATATTTTTTTTAAATTTTAATTTTTTGCTCACATATTTCACGCCAATTAATTTGTCCATTTATTTTCTTATAATTTAACGGCTAA**[C/G]**TTTTGAGAAATGAAGGAAGACTGATTGCGAAACTTCACGTTCATCCCTGAAAGACAGAAAGATACGAATGAAAATGATATTAACAAATCGAAAAGTTAAA

>scaffold81.1.18686

GATATATTTAATAAAACAAAAGATTTCTTTTTAATTTTCTAATTTTTCGCAAATGAATTTTAATCGATTAATAAATCTAGAGTATTGATTTTCTTTTGTA**[C/T]**GGTATCAAAAAGTAACATTGTATAAAAATAAGAAAAAAAATAGATTCGGGTAGGATGCGATCGATTCTTCTCTCTTCCTACAAAATTTCCATAATTAAAT

>scaffold81.1.192404

AATGGATACCAAGTTTCTATTGAAATTACAATTATAATATTTCACAGACACAGGATAGTATTACTACTTGAAAGTAGTTTAAGAAATATTCCTTTATCAA**[C/T]**GATTAAAGAACATTTAAAGTTCAAATTATTTTAATTTTACAATTTATGGGAAAGATATTACAACTCTGTTAAAAGAGTATCAATCTATCATCTTTATTTT

>scaffold81.1.24497

TGCTTTATTTATTCTTTATTTTAGAAAACTTTTTTGCCAATCGGATTTTCCGTTCTTAAATTTCTAAATAGGATTATCTCTTCGTATCTTTTTGTCGATT**[C/A]**TTTCTCGGAAGAGATCTACAAGTTCAAATATTAACTTTTTTCTTCGTTATTAAGAAGAAGTGGATATGGATGATTTAAATTTGAACCGCACCTGCGAGTA

>scaffold81.1.347441

ATCTGAACAGTATAAGAATAACAGACTGTTCTCATTACGATTTCTTGTTCATTGATCGAGCTTCAAATTATGATGAGGCAACAAGGGGAAAATATTTTCA**[C/T]**GGCAACATGACTCTCGAAAAAAATTCTAATTAACCCTTGTCAACATCACAGACTGATTATACATGTATCTGTTAATTAATAGTCACCAGTGTTGCAAGGG

>scaffold81.1.469434

AATATTTAAATATTATCCTAATTATAGTAAATATAGAAAGTTGATAAATGAAATTCTATACGATTTATAAGGATCACTCTCAATATCAGTTATTCTAAAA**[C/T]**GAGAACTTCATAAGTAGGTCGAATTAGGAATCGAAATTTTTAATATTATAAAGAAATTGATTAAACTGATTGATCTTGAGAGATTAATCACCACGTTTGA

>scaffold81.1.198065

ACGTCGTCCATTCTCTTCCCAGAAATAATCACTTTCATAGAGAATCCTTTATCCTTTTTCATTTATTGATCAACAACATCTCGTATCTTACTTACATCAC**[G/A]**TTGAACAACTTTCAACATCCACCAACCAACTTTATTAAAACAATGCATCCAACTCGTGCATCCTCAACATTTATCATTTTGCTTCCAAGATCACTTTTAT

>scaffold81.1.134157

ATAATGATAGAACCATAAGTCGTCGCAATCTAAAATATTATTTTATATCCAATTTCAATCCTACTGATCCAATAATGAAAAATGTTGATAAATATTAATG**[T/C]**AATGACAAGAGAAATCCAATCATTAATTTTATTTCTTTCAGAATCCACGGAATAGTGAACAATTTACTAAGCAAGAATGCGACTTGTGCTACTTATGATC

>scaffold81.1.145673

TTCATCCCTGCAAAATATGAACGATTAAAAAAGTGGAATGTTCTGTACACATCTGTCCTGTCTCTATTTATGAATCCTGTCGTCTGCTCCTTTTGGTCAA**[T/C]**AGGCAGAAAGCTCGAGACCGATATCGACGCGTCATTCATTGCAGACATTTTTTTTTCTTCAATGCATAAAATGACAATACTTGATTCTATCAATATCAAA

>scaffold81.1.5983

TTTTCGACTACCATCGAATATGTTCTCGTATAAAGGTTACGTTCTTCGTGACAGCCAATAAGTGTATATTCCGGAGTGATATGTGACTTGGATTTTGAGG**[T/C]**AATTAATTGTGCAGTTGATGAATTCAAAATCCAAATATTTATATCGTTTTCAAAAAATTGTAGAATTGATATAATTGTGGTAAAGTTTTCTCTGTAAATG

>scaffold82.1.1569049

TTGCAAAACTTGGAAAGATATATGAAAGATGTAACGAATTGTAAATAAAACTAATATACTCTTGTGAAAGAAACTTGATAAAAATTTTTATAAATTTTCT**[A/G]**TTCTTCATTTAGTGAAATCTACGTTACCTTCCGAAGATAAACCCTAGATTACTAGAGACCCTAGATAACCTTATTAAATCACACATAAAAAATTATAATT

>scaffold82.1.1808239

TATATATAAAATACATATAATAATTACATACTTATATCTAATTAAATATATAATTTAATTTTACATAAAAAGAAAATTCATCCATATACAAATTGTATTT**[A/G]**TTAAACAATTATTTATGAAATATCTTTGGAAATATGTTTTTGATTCCTGAGAGACCAAGTTTATTTACCAATTTATAAACAATTTAAATTGAATCAAACA

>scaffold82.1.2296715

TAGATTATATAAAATTTTTTTTAATATTTATATAATATTTACAATTAAATAAAGAAATTAGGTTAGAAAAAAAGATACCAAGAATTTCATAAATTAAAAC**[A/G]**TTAAAAAAAACAAATTAATGTTATGTATATAATCATTTAAAGTTTATGCTAAAAGTTAACAAATATCCAAAGCATGTATTGTTTTTCTGTAATTTTAAAA

>scaffold82.1.1360970

CTTACGCTTAGTTATGAATTATGTGATATTACTCACAAAACATTAATAACAAAGGTTAATAATTTTATTAGAATGTATGGTCACACGATAGTAGAAATAA**[C/T]**GCCATTACGTCATTCATTTATAAAAATTATATATTCACATTTTTTAATTTTTTAAATATTTTAGCAAAGTTATTATACAATTATTTTGATAATTATTAAA

>scaffold82.1.2148867

TTCCCAATATTGGATCACGAAATGTGATGCGTCAATCTTCCAAAGCAGTGCTGAAAAATAAAGAATCGCAAAAAATTCATTTATTCATTCCATAAAATAA**[C/T]**AGAAATTTTCTCAAAATTCTCAATGAAATGTGATCAATCTTTTAAAAACAAAAGAATCGCATAGCATGAAAATTCATTTATAATAACTAATTAATAATAA

>scaffold82.1.2325973

AAAATGGTATACATGATGGGTTTGTACGAAAAATTTTAAATGACTTCTTTTGGATAACAGAGTGGCAAATAAGCGAAAATTAAAATATATTGTTGAAAAT**[C/G]**TTTGGTTAAATCGAGTATTAGAAAATCGATTTTGCGATTTGATGTAGGAAATTAGAAAATCGTGAACAAATTGAGAAAATGAAAGACCAAGAAATTAAAC

>scaffold82.1.2426126

CAAATATTCATAAACATATTTTCAATTTAAAAATTTTCATATTTATTTATTTATTGGGATAATAACATAATACAAAAATCATAATTTCAAAGAAATCGAA**[C/G]**AAATCGAAGAAATCTGAACATTTCCAACTCCTGTAATGCAATTTTTAAAAATATTATATATAATAATTTTCATTAAAATCATTACGCAATAGCCAAATTC

>scaffold82.1.2485164

AGAATGGATTAGAACCAATGCAATAAACGTATAAAAATATAACAAATGTTTTATTTTAAGATTTTAAATGTCTTAATGAAAGTTCTATATTTTGAAATAA**[C/T]**GAAAAAACGATCATTTATAAAGATTCTCGAAAATGTTATTTAATAAAAATTTTATTTTGTTACACAAATTTTAGCAAAACATTTTAGAAGATTTAAAGAA

>scaffold82.1.1374732

AATATTCTCTAATACTTCGTTTAAATATTTCATTTTATTCTAATCAATTTAATGATATTGTTCGAATTATTGTAATGTTATAATTTTTATTATGTATAAC**[G/A]**TTTCATTCATTTTTACTTTAATTTAATATATTTTATTACAGATATATAATATATATTTTTGTATTTTATTATCTTTAAATAATATACTATCGAATAAAAG

>scaffold82.1.1496071

CGATAAGTTTGATGAAGTAATAATTTATTATCGTTAATTCTGAAAGTATATTCAAAGAATATTCTATGATTTTAAAGTATCGTTGAAGTGATTTTAGTTT**[G/A]**TTTCGATCAAATTGTTCATAACGCTTGTTAATATTTTTTTATTCACTGTATTTATATTCGAATTAAATATTACATAAATTTTTTTGAAAATTTATTTCTT

>scaffold82.1.1860951

ATAATAAAAGAAATATTAATGAAATAACTGATAAAATAAATCGATAATATATTTTTATCAATGTTAATAAATTGTTATGCAGTTAAATACGAATTCATCT**[G/A]**TTTACGTATTCACACTTTTATTCTTAAACATAAAACTTGTTATAACATTGAAAGAATGCTTTTGCTTGTGGACCTAACTCAAACACTTTACTCATTTCAG

>scaffold82.1.2190092

TAAATGGTATAATTAGATTCGAAAGAATAGGAAATTCTTACGGAGTTATCAAGCAAGAATAATTATTAAATATATAGTTATTAAATAACAATAAATGAAA**[G/C]**TATAACCAAATCATATAACATAGATACTTTGGAAAATAACTAATTAAATAAAGGTTTTCATTTAAATATAATAACCATTTAAATATAGTAACAATTCTTA

>scaffold82.1.1571022

CCGAATATTAAATATCAAAAATTTTCGTCTTGTGGCAAAATATATTTTGTAAAATTCAAATTTCACATATCAAATTTAAACTTAATAATCCACAGTCTAA**[T/G]**AATTACTATAGAAGTAAAAACTGGCATCTTTCCAAATTCTTCTCGTTATACAACTCGTACACAGACTCGGGGAAAACTCGCAAGGTACCAACTTGAGAAA

>scaffold82.1.1622787

TCTCCTTATACCAATTAACTTTCGCATACAACATTCTTTTCGTGACATTCTTTTCTAAATTTCATTATTATTTATTTCACATTTATTTAAATAAAATAGG**[T/C]**AATTCAATTATTGTTATTATAAAAATAAAACTGGTTCATCCAAACGATAAATAAAGAGTGAAACGAATTTTTATCATATAGCTGGAACATTGAATGTGAA

>scaffold82.1.2443204

TTTCTTGATTCACTTTAAATTTTTTTCTTTTAAACTATATATCGTATTTAAATAATTAAAAAATCATCATCCTGTTTTTTCTATGTAATCTTCAATTCCA**[T/C]**GTCGAACCTTTGGAACTCAAGCTTTTATAAGACTGGTTATCGTTTCCTTCTATTGCATTGTAAAGATCTCTATAAATTGAAATAATTACTTAATTCGAAT

>scaffold83.1.1282663

TTGCGAACGATTATGCGAGGTTAATTGATGCAATGTAATTTCTGATAAAAAATTCATGTTCGAATTTTCTCAAAAAGTCATGCTTACAGTCTCAAGGTCC**[A/G]**TACGTAATGAAATAAATTACATTAACCTAACGAACACTTTATCCTTTCCATCCATTATTAAACCTGTTTCGCCCTTTATGCATTACAAATTATTACTTTA

>scaffold83.1.130257

ATATTTTCTATATATACGTTCGCTTAATTATTTTTTAATTTTTTTTTTTCATGAATTATAAATATACCCTCTCATTATTTATTCATCTTTAAGATCATTT**[A/G]**CGATCATACATCACAATAACACACAATCATATACGATATACAATTATATACAATTATTTAATGGAAAAAGCTGTAATAATATCCTATGTTCACTTCATAA

>scaffold83.1.237039

TTAAAACAGTCAATGATGTCTACACATTTCAACTATTAAAAAGTAGAATTTTTAGAAAAAATCTAATATTTATAAAAAAATAAATATTAATAAAATAATT**[A/G]**TCATTAGATTAACGAAATTCAATATATAGAAAAAAAATATTTTAATTAAAACTAAATCATATATATTATTATTAATTTTTCATTTTAGAATTTTGTTAAT

>scaffold83.1.640563

ATCGCGTGACAGTGACAATGGAAATGGCCGCATATAACTGTTCGAAACCAGAATATTCTACGCAATTTAATTAATAATATTATTAAAGAAATTAGGAGTT**[A/G]**CAAATAGAAAATGCTAATAAATTTTGCGTAATAACAGTAAATTTGATAATAAGGCAAGTAATATTTTATAAATAAATTTAATAAATTATAAATCATACGT

>scaffold83.1.1071686

CGCAAAAATAATTTTAATGTATAAAATATATCATATTATAATTTATTATATTTCTTTTCAATTATTTAAGAAATATCATAAATGTCACATATTTTTGAAA**[C/T]**GAATTAAAGTAAGATATTTTATCGCGTGTCATATATTAACATTCAAAATGAAAACGCACCCTTAAACGTTTGTTTCGTATTAGTGCGCGCGCATGCGCAT

>scaffold83.1.1217492

TTATTTGAGATCGCTTTATAAGATCGAGTTTATGTGATATCTATACATGAAATATGTGTCTAATGTGATATGATTGAAGAAAAATTAACTTCGTAACGTA**[C/T]**GTTGAAGGATCCTTTTAAAAACTTTTCATTAATATTATTGACTAGATAGCTTTTTTTTTTTTTACTATATAATATTTTTGATAGATTTTTAATATCACTG

>scaffold83.1.1300652

AAAAGTGAATTGAAGTTAAAATAAAAAAAAATAAAAAAAAGAAAAAAGAGAAAAACGATCGATCAAGCAAGATTTCCTTCCTAATGACTCTGTCAGTGCA**[C/T]**GGGGTCGGATAAGTAGAATAGGATCAATCTTGGTCGGACAAGTGGAACACACTGGTGGCCCATGCGACAAGTAGAATTAGATTGTCACCGGACGGACAAG

>scaffold83.1.1324006

AATAATTTAAATGTTCTTAGTAATTGATTCGATTGAAATTATATACTCGATGTGATATGTGTTTCTTCTCAAGAAAATTGAAAATGATTTAATAAAAAAA**[C/G]**TTTCGTGTAAACATCATTCCGCACTACGTGTAAAACTTCGTCGTAAAATCTATTATCTAAAACTTCCATTGAAGACCATTTATCCATTTATCCTTTTCAC

>scaffold83.1.1379855

TCGTGTTCGCCGTTACTCGTCATTTTTCGTGACGCTTCGTGCGAATTCGGGTCACGTGAGTGTTAGCCTAAACCGCGCGATTATTCATTTTGCGATGGGA**[C/T]**AAAATCGGTCATTTTCGTTATTAAATAACCGACACAAATGATAAATTTCGCGAACGATCATTGCACTTCGAACAAATTAAACTTTACATGATATAGTTGA

>scaffold83.1.1379957

AAATCGGTCATTTTCGTTATTAAATAACCGACACAAATGATAAATTTCGCGAACGATCATTGCACTTCGAACAAATTAAACTTTACATGATATAGTTGAA**[C/T]**AGTTCAATCGAGTTTAATTTAATTAAATTTCTCAATTTGATCGTGTGAAAGATAACTTATTTAATCGAGATTTTATAACAGATATTTTAAGATTTATTGA

>scaffold83.1.305205

GAATGATAAAAATAATATTTTATGATTATACTCATATATTATTCACATTTGTAATTTATTGAATAAATTTTAATTCTTTATTTTAATCATAAAAGATTGA**[C/T]**AAAAATCATTATTCAAAAAATTTTATCAATATTAATATATGTAATAAATTCCTTTAAAATCGTTAAAAATAAAAATAAAAATAAAAGTTATGAGAGGAAA

>scaffold83.1.604005

AATAATATTTATATGTTACGTTATTTTCTTTTGTTATGTAAAAGATATTATAATTAAATTATACAATACTTTTGTTATGGATTTACACTTAAAATTCAAA**[C/G]**TTTTATTAAATTCTGTGAAATCAATAAAAGTATCGTCGAGAGTTTTTGATGTTAATATTTGTAAAATTAAATTATTACGATACATTGAAAGTGAAAGAAA

>scaffold83.1.638831

ATAGATCCATATTGAATATGTTAATAAAAAATTTTATTTTGTCAGAACTTTCTACAATTTATGTATCAAAAAATTGAATTCTAATTTTAACGGACTCAAT**[C/G]**TATTTATTACTTCTATTAGATTAATGAAACAATAAATATCAAATATTAAATTATTTAAAGAAAAAATTATATATATATATAACATTTAATATACTTTTCC

>scaffold83.1.658873

CGCCACCTGAAACATTCGATTCTTTTTTAAAAATAATTCAAAAATTTTCTATCGCATCTAGCATTTAATTTACTTTCACGAAATTTTATTCTCTCGAACA**[C/T]**GAACCATTTACAATTATCGAATTACAATTATCGTGATTCTTAAGCAATTTTCGTAAATGGAATACGTTTTGTTATTACAAAAAATTAAATACAAGCGATA

>scaffold83.1.710645

GAATTATATATTGTATGATATAACTCTGCAAAAATTTATATTTTAAACGTGAAGTATAGATAATAATTAATATATAAAATAAGATAATTATTTAGATCAA**[C/T]**GGTTACTTGAATAATATAATAAATAATGATACTTATCTCACATAGTTGGTCAAAAATAAACTTTACTTATTTGTTAACTCTAATATAAAGAAAGAAGATA

>scaffold83.1.1185534

CGTGACGTGCCTATTGGTCGTACGCAATGCTACGTTAAACTGTTATTTAGAAAAAACTTTTTTCGTCAACAATGTGTTTCACATATCTACAAGAAAACTC**[G/A]**TCTTCCCAAACAAACGGCCTAAAACGTCATTTCTCACAAGGGTCTATATATTTATACGTAGTATTACATTTCCTCGTTGCAGCGCGATTCGCTTTTCAGT

>scaffold83.1.1195791

ACTCGTCTAAAGAAACGAAAGAAATGTATCTTGATTTTTCTAAATTAATATATTTTACGTAATTGATCACTTTTGCTCCTAAATTATTGCTACGAATATC**[G/A]**TTCATTGAATTACAATAGAACTTCGATTATATGGGTGGAACCAGATAGATATAAATCCACCGATTCAATTTTTCCTTAATACTTGCTAAAAATATGGCAA

>scaffold83.1.1283807

AAGAAACTGAAGTTCATACAAGAGAATACGTGAAACGTAATTATATTATTAGTAATTTATTGATAAACCTCATGAGAAAGATCAAATAATAGGAAGGTTC**[G/A]**TAAGTAGGGTAAAGATTAAATTTTGATATAACTTTTTACTTCTTATGATTCAACATCTAACTAATTAAGGGAACAAGAAACTCTTTTATTTTTGGTATTT

>scaffold83.1.1344731

GGAATTTCGATTTCTTTTTAATCTAGATCCTCGTAAATTTCCTTCAATTCTTAAGGAGTGAAAAAGGAGGTGCACGAGTGGCCAAACATCGTTCAACGAT**[G/A]**CTAATTAGCACGTATATTAGCAGATTTATTATCCATTAGCTCATCTAGTTATACAAGGTGAATCCTTGTACATATTAAAAATAAAAGAATAAAAAACAAA

>scaffold83.1.144967

CTTTCGTTAGTCAACAGATACGTCGAAATTTTTCTTACTGTTACAGATGAACAAAAGATCTTTGGAAGAAGATGATGAGTAAATTGTGCGTGGCTAAAAC**[G/A]**TAAATATTGTAGAGATATTTTTTATTTATCTATTCTATTCGAATCTCGGTAGAATTCAATGGAAATTATAAATTTAATTGGACAACTAATGAAATCTCTG

>scaffold83.1.236253

GATGTTTAAGATAAATGAATTTAAAGATTAGATTATACTCAAATAGTAATACTCACATTAGTTTTTGTATGTATATACAAATTCACTTATATTGGTAATT**[G/A]**CATTTTATTTTGTATATATTTATATTTTTAATGTAAGTGATGACTTAAAATATAAATTTATAGTATGACTCTTAAAAATTTATAGAGAATTTTAATTATA

>scaffold83.1.236290

CTCAAATAGTAATACTCACATTAGTTTTTGTATGTATATACAAATTCACTTATATTGGTAATTGCATTTTATTTTGTATATATTTATATTTTTAATGTAA**[G/C]**TGATGACTTAAAATATAAATTTATAGTATGACTCTTAAAAATTTATAGAGAATTTTAATTATAAAAAAAACATATTGAAATTAATACATATTGAATATTT

>scaffold83.1.242280

TTTTGAAATGGATATTTCGTTGAAATACGCAGTCATCTTCGTTCGCATTTATTACATTTCGATTAAACGAAAAATAAATATTCTATCTATATCGGATTTC**[G/A]**TCAATTTAAAATTTATAAAAATATTTATCTACAACGTTCACAATACTTCTTGTTCAAGATCATTTCAAACGAATTCTCTTATCCCTTGAAGTATTCCACC

>scaffold83.1.44117

TCAGAAATCCCCCTCCCCTCCGCTGGACTTTATTATTGTTATTTTTTTAAATTTTCAAACATACTTCCAATGTTTTTTCTTTTTTTCTTCTTAGTTTAGT**[G/A]**CGCCACGATTGAAATTATATATTTATCTTTCGTGATGAAATTTTTAATTAAAAAAATTTTAATTTTAATAACGTTCGTATCAATGATAATATTGAAATTT

>scaffold83.1.510707

CTTAACGATATCTCTGTAAGGTAAATGAAAGCATTTTTCAAATATTCCTCGGACTTCATCAAACAGACTTGATCTTAAATTTTATTTATATAAAGTAATA**[G/C]**TTGCTCGATCGTTAGAACAAAGAAGATAAAAGCTGAATTGACGACATTAGTCGAAAAAAGAATCGCAGCAGGAAGTAGTTGGCATTTTGCGTCGCAGATG

>scaffold83.1.598238

CGTGTTTTATAATATATCAAAATATAATTTTAATAATATTCCTATTTTTTATGATATCAAAATCGTTTCATAATTGAATACAGTAAGGTTATGTTTAATT**[G/A]**TTTAATTTTTAATTAATTATGTTTAATTAAAGTTTTAAAAAAATTTTAAAAGATTAATGGATCATCTGAGAAAATAACTAATCCTATGTATATATTTTAC

>scaffold83.1.941613

ATTTATTCAAGATTATGACGTTTCTAGAAAGTTAAATGTGTAATACGAGAGACTGATTATCCTTAACCTCCAGCATTGTTCAAATTATACTTCAATTACA**[G/C]**TTCAGAAACAATCTCGTAATTATAATTTAATTTATTTTCACCGTGAAAAAAAATTATCGTAAATAACAATATCTTTATTCATCTTGCCATTGTTTCTTAT

>scaffold83.1.150354

TGATTTTTGATTTTTGATATTTTATACGATTTAATTGTTATTATGAAATATATATGATAAAAGGGAATTTATAATCTTCGATATATCATTAGTACGATCG**[T/C]**AGTAAATAATATTCTTTCAAAAATAAACATATTTATAATTTAGCTTAAAAAAAAACTCGATTAAACTCTATTAAAATTAATCAAACTTTAAACCTCTATA

>scaffold83.1.452111

GACGGAAAATAGATCTTGGATGGAAATGGAGTTTGCCTTTGCGTGAGAAATGTATACCGGGTGAACGAAATATAAATATAAATATCATTTGGCATATATG**[T/C]**AAGATGTTTTATTAATTAGTCTGATCTTTGTCTTTTCTTTACAAAATGGAATAATTTTCTCTTTTTACGTTAATCAATACAGTTTCGTATTTCTTGTGGA

>scaffold83.1.598582

AAATTTTCTTATAATTTATAGTCCTTTATATATTGTTGAAATATATAAGTATCACTTACTTCGATTATTTTTCTGAGAAAACAAAATCATAAAAATTTCG**[T/C]**ACAATCTAAAATTTTCTTTATCATATAACAATTAATAACTAATTAATAACAAACTATATCAACAATTATCTAATTTTGTCGTAATTTTCTCATAAACATA

>scaffold83.1.601931

CCTCCATGGCTTGTGTGCACCGCATTTCGTGTCCGATGCGTGACTAACACAGTGGTTTATTGCATGTTAATACGAATCCGGTACCACCAGTATTTTTTCA**[T/C]**GAGATAATCTTGACTCACCCTTATAAACAGGATATGTATATTGAAAGAACGAAGGATTTTAGGAAATTTGGTTAAAAATAAGGAAGAATTTTAATAAATA

>scaffold84.1.263154

TCTCGATAAATAAAATAACTCTTATTGATGAAATAATTACAATTAAAGGGGAATATAATCGTTAATCGAGAAATTTATAAAAATGAGCAGATGATGTTTC**[A/G]**TAAAATGATTATATTTCCTTTAAAAATATTTTCATAAATAATTTTGCAATTTCAATATGTAATAAATTTATGGCATTAAATGCCTCATAATATCATATAA

>scaffold84.1.285957

AACGCGTAATATCATTAAAATATTATAATAAATAAAATAAAATAATTAAAATATTTTGCATTCAACGTATATATAATTTTTCAAATTAAATTATTATTAT**[A/C]**GTTATATGTATATTTAAATGTAAAAATAGGAGAAATAATATTTAAAAAATATCATTCTATGTTAATTAAAAAATTTTAATGAACTCGACGTAAAATGTAA

>scaffold84.1.194275

TAACGAGACCCGGATTGCATACTGAAATAAGCTTTATTATTTTCTCTAAACTATAAAGTTTTTCGATTGAATAATCAATCCTGCGTATTAATTGAATATT**[C/G]**TTCTATTTCCAGCATTGTAAACATTTGTCTTTCCTTTAATATTAAAAGCCACCCTCGTTCTTTAAAAGAGATACGGTTATACGCGCGAAGAATAATTCGA

>scaffold84.1.254139

TTAATATTTCGCGCATTATGGAACAAAAAATATTTGCTCACGTCCTGGCCAATTTTATAGTTGTCGTAATTAACGCGTTGGAAACGACGTTGAGTATGAA**[C/T]**GAGAGAGAGAGAGAGAAAATTCTTTTGGAAGTATCGCTTCGTTTCATTTTCCCGAAAATTCATCTCGCATCTGTTCACGGAATAAAATTTAAAACCTTTA

>scaffold84.1.397133

ATTGAATTTTCTATTTTATTCGTTTTTGCTTTGATTCATTTGTCTTGGTATTTCGTTTCAACAAATGATGATAAATATGACAATGATATGTTCAAGAAAA**[C/T]**AAAACATGAAATATAAAGAAATAATGTAATATTTTAAATTATGTCAATATAATATAATAAATTCAATAATATATAAAATAATATTAAATAAACACAAAAA

>scaffold84.1.414095

TCTTTATAATGCATTTTTCTTATATATAATAAATGTCCATATTTATAAAATTGTTTAATTTTTATTTTAATTGTAAATAAATGTCAAAATGTAAATTGAA**[C/T]**GAATGCCAGATTATTTTATAATAAATGTGTTTCAAGAGATCTCGTTGAAACGAACGAATCGATTCTTTTTTTTTTTTTTTTTTTTNNNNNNNNNNAAATT

>scaffold84.1.665427

TGATTAAACGATTAAAATACCGAAACGATCGAAGAAAAATTGAATGAAATTAAATTTAAATCGTTTTATATCTTATTATTTTCAAAATTTATTCGAGAAA**[C/T]**AGAGGGAACAATCGAAGTGCTTTTTCATTTTATTCCAATATTGCCGAGATGCAAGGAATATCCAAAGTGTCCATAACGTTTAAGACGGTACGGAAATTAT

>scaffold84.1.281761

TTTTTATTAAGAACAAAATGCTTCAAGTAGTAATAATGAGTTTGTTGTTTAATTTTTTAAATTGGTAGAGTTTTTTCCAAAAAATTTGAAAGAAACGAAC**[G/A]**TCGAATAAAAGATATACAATTTAACAAATAAATTTTAATTTTCCAAAACAAACAGTTTTAAAATTTTTAATTATCTATGTTTGTGTTCAAATAGTGAATT

>scaffold84.1.574251

AAATGGGTCACGCGCTCATAAGTTGAGAGAAGAATGAAGAATCGTGGCATAAATAGAGCGCGCATCGTCATTGATCGCTTTTCATTGATTATTATTTATC**[G/A]**CCGTGGGAGTACCACGTTAAGTATAAAAATATATCGTTTATCGATTATCGATCGATCGATCGATCCTTCTTCGATACTTGCCCGTTTATTTTCAATAAGC

>scaffold84.1.182595

TTCGAGTTCCTTTAAAATTTAACTATATTCGTAGCTAGATCATTTTATGAAATTCGTACAATCCTTCCTATAATCGCGAATGTATTTCATAGAACCTACA**[T/C]**GAAATCCTTACCTTATTTTAATTATGCAAGATTTCTATCATAGTTATCTTGCTCGATATATCAAAGATTAATATTCTTGACATTTTTAACTAACGAGTAA

>scaffold86.1.166425

ATTATATCATTTCATAACTCGTACAATTATATTATAAATAAGTATTTTCTACAGCTATCATAAACTATTACTTATTATTTCCAATATCAGTACAATATTT**[A/G]**CATTAATAATCACTTATTGATAATTAATTACTCCCAATACAATATTCGTATCCAATAATAATAACAATATTTTTTTCATAGTCATACTCAATAATAACAC

>scaffold86.1.136808

CCTTTGAAACTCGCACTTGTGCCACTTCTCGACTCCCCTTTCTCAAAAAAAAGCAGCCGTTCTCCAAATCACTCGCACAAACACTCCTCCCGTAAACACC**[G/A]**TGCCCCGCGCCATCAACATCTATCGAAGGAATTAAAGCGAGGAGACAAGAACACCCTCGATCCAGCCCGAGTTTTAAACCCAAATACTTACGCCCCGTGA

>scaffold86.1.193966

GGAATATCTTAGAAGAGATCGAACCTACCAATGTAACACAATTGCGTTGAACAATAATATATGCGACTATCCTACGATCCAAAAATAATCACAGACGATT**[G/A]**TCATCTTTTGAACCCCTTTTAAATTTACAATACACTAAATTTACAATACTTAAATTATTGAAATATCATTCAAAAAAGTAATTTGCATAATATCCTGCTT

>scaffold86.1.144701

ATTTCTTGAAACAAAAATCCCATTCAACCAGGAGTATATATAGCAAAGAAAAAAATGTTTCAAAGAGTAAGTAAAATTCTTGATAATAATTTTTAAGAGA**[T/C]**AGATTAAATGATTTTCGCATAACACTGTATATAGTTATTCTTCTGTGAATTCGTGTCATAATCGCGTATCTTCAAAATGATAATCCTTTCTTCCCGAATA

>scaffold87.1.1198265

AGTGACCAAGTTAGTCTATCTCTTCGGAATGATAAAGAAATCTCGTTCTTGCGCGTAACGACAGCATCCATTGTCTTGTTTTTCGTTGCCACCCTTCTTC**[A/G]**TTCTTATTTCCTCTTCTTCCTCTTCTTCGCTTTTCTTTACAAACTTGAAGCCGCAGAAAGATCTATATATCGATCGAACAGTTTCATAGGATAACTCGAA

>scaffold87.1.1219777

ATTATATAAAATAAACATAAAGTAAAAACGTTTTTAATTAGATAAAACTCGTCTTTAAAATAGACGAAAAGAAAGAATATTTTGAAAGCTTATAAAAATT**[A/G]**CTTAAAAAATATCTTTATATTCCAGTCTTATACTCTATATTTCAATTACATTACATTTTATAAACATTAATCTCCGGAAATGAGACAAAATTATCTTAAA

>scaffold87.1.1606800

CCCATGCAACTTGACTTTCAAATCGAACGAGAATCAATGAATCCACATCGCAAAGAACGTTGGTTGCATAAATTCTCGTGGAACAAGATATTTTATCTTT**[A/G]**CTTTTTGAAAGGCAATACACATTTCTAATTAAACTTTAATTCGTCCTAATAATACAACATAATAATATATTAATAATCCATTATTATAATTTCCGTATTT

>scaffold87.1.694045

GTGAGAAGATTATAGGACTCGAGAATATAAATTTTTGTCCAATTTTTGTATCGTAAAAATGGTAAAACTTTTATACCTTTGCAATTTTGATTTTTCTGTC**[A/G]**TTATCACTGTTATAATATTCCGCGAAACAGAAGAAATTACACGTATCGTAACGAGTATCCACTTTTCTTCTTTTTATTTTTGCACTTAAATTTCAAATAG

>scaffold87.1.850099

TAATATTAATATTAATCTTCAAAACCATATATTTTTTTCAATCACATATATCATTTTAATGATTATCACATTTCTATCTTTATTTTATTTTTTTTTCTTC**[A/G]**TTTATCTTCTTTTCTTTTTATATTTCGAATTTTTCAGATAGATATCTATTCCCTTGATTATCTACATTTTGTCAAGCGAAACTCGCCTACAAAAGAATCT

>scaffold87.1.1123093

CAATAATTACCAGATACCGAACTACTAAACTAATGAGAAATTACGAATTTCAAATTTTTCAGGTATTCCACTTATTTTTCAAAAAGAATTAAAAATTCAT**[C/G]**AATATGAATTTTATGTATATTTTGTAAAAAAAAAAATATATATATATATTTTCTACGACTCAATCGTATAATGAATTTATTATTTTATAACAAATTCATA

>scaffold87.1.1449555

TTGGCGTAAAATTATAATGATGCATTTTATTTTAATATATCATTTATATGCGATATATTTAAATATTTATCATCCCAGGAATATTAATATCATCCATATT**[C/G]**ATATAATAAATGATTATTTTCGTGCGTTTAAGTTGATAGAACGATTTGCCTAGTTCTGATTCCTCGTAAATGTTATTACTCAACAAGATTGGAAACACCT

>scaffold87.1.1798763

TCGGCGTAACTTCCAGTTCTTTGGAACTTTGAACAACTGAGTTATATCGATTTATCTGAAAGATAATTTCAGATTCTTTGTTTCAAATTTCAAAAAAGAA**[C/G]**TAGCTGTTGTCGCGATTGTTTTTTTTTTTTTTCTTTTGAACAAGTAATGAATTAATATCGAATCGAAAAATGTTTTTTCACGGGATTATCATTTCATCAA

>scaffold87.1.609477

ACGGTTCGTAAATATCACCAAAAGTTAACCACATAATTTCGAAACAATGCGTGAGAGCATAAATTTTGAATTTCCACGACATTACGCGTAAATTATTACA**[C/T]**GGTCTGTTGCATAGCGGTTTTGAATTTTCTTTGACCTCGTTCGAAAATTAAACATTACTTCATGCAATAAATGCAACCACGGTATTATGTAGAAATAGCA

>scaffold87.1.1052843

AAAGTCATTTTTCTATTTCCAGTTTCATATTACATCATTTCATCTTTCTATTTATACTTTTTATAACAAAAACTTGGTCTATGCATTTAAAATTAAATAA**[G/C]**TAGATAAATTATTAATGTTATTACTTTGTAAATAATTAGAGAATGTAAAACGCGTAAGTTCTTCTTTCCATTTCCAATTACTTCAAAGCATAGTACTTTC

>scaffold87.1.1221798

TATACTTTATAGAATTTTCCCTTTCTTTTGTTTCTCTTTTCAATTTTGACTAATTATTGGGAAATGGAAGAGGAAATTTATTTATTAATGAATAGTTTGC**[G/A]**TCGTAAACTTAAATGATGAAAAATAAAGATAAGAAGATAATAAATTATAATTTTATAACTTTAGGTAGATTTAAATTTGAGTTCAAATAGATTTTATTGA

>scaffold87.1.1226471

TCTACATTTTTTACTTCTTTTTTCAAGAAAAATCTCATTCTCTAAGCTCCTTTACGTCTCTACAACAGACGCAAAATAACAATATTTTACATATTATTTT**[G/A]**CAAGAAATTTAAAACTTTCTAAAAATCTTTTTACATATAAATTCTTTTAACATTTCTTTGAAAATTCTATAAAATACCACGAACTAATTACCTATTGAGA

>scaffold87.1.1445188

AACAATAATCAACAATAATCAGCATAAAACTCAAAAAAAAAAAAAAAAGATTAATACGTAAAACGTAATATATTCGATATAAATTTTCATTCCAACCGTT**[G/A]**TTCCTTTAATCGATTAAAACTCTTTTATGTCGTACGTGTGCAAAATATGCAATCTCGTAAAAAAATGTTAAAAAGAGTACAAGCGGACGAAGTAAACCGC

>scaffold87.1.153476

TCAGGAAATTATTTCCTAAATCAACCGATAAATTTTCATAAATAAATTTGATGATATTATTCAATTTTAATATATTGAATATTTCGAAGTGTAGGAATAA**[G/C]**ATATGATAATGAAATACTACTTATTGCAAATGAAATGATGAAAAATTGGTGCAAGTTAATACATCGATTTATCCAGATAGATAGTGAAATGATTTGGAAA

>scaffold87.1.474426

GAAGATTCGATAAAGCAATTTTTATTAAAGTTTCTTGTAGTTTTATTAGAATCTTTTTTTTTTTTTTTGTTAAAATTTTTCCGTTGAAGTCTGTCTTTAT**[G/C]**ATTTTTTTTAGAGAGGATTAAATTGAATATTAAAAAGGATGTGGATTCGATTTCTTCTAATTTTAAAATTTTAGAATTTCTTTTAAATTTACATGAGTCG

>scaffold87.1.994324

AATTGTATGATTTTGCCAAAAATAATTGAGGAATAAAATAATTATTAACCTTTTTATTTTGGAATTATTTTGAAATTTTGTTTTAACAAATAATAATTTT**[G/C]**AGTGTATATATCAGAATTTGTATATAATAATTGAATACCTCTGTTATCAACAAATTTGCAATAACTGTCGATACGTTCGAACTGGAGGATCTTTCTAGAT

>scaffold87.1.542065

TTTGTATATTTTATAGTATAAATTCAAAAAGTCAACAATAATAGTCTTAATTTCTTTATATCGGTTTTTATATCTTATATTTTATGTGTATATTTATTAA**[T/C]**AAATGGCAAGTGTATATAAATAATAAATAGAAGTAAGAAAATTATACTTTTAACGTAAAATTTATTCAAAATTCTAGCTCTCAAAAATCGTGTAAAAATT

>scaffold87.1.557142

ATACGTCATACATGATCCAATTATGAATTTTTCTTTTACTTTATAAAATTTTATTGTCAAAATATGTGAAAATTTGATAATAGAAGGCAAATAATTTAAG**[T/C]**AATTTAAGAATAAAATAATTAATTATTCATGTTAAACAATTTGTATGTCGATAATCTATCCAGTTCTGTTAATATTTAATTAGTAAGTAAAAGGAGAGCC

>scaffold87.1.797648

TTCTCGAGAAAAGATTTTTTTAAGAAAATTTCTAGTTTTATTTCATCGATTCCATATCGCAACTTAAGCAATATGATATAGTCAGAAAATAAGCAAATGA**[T/C]**GGAGAAAAGTTGTAAATTCGATGATTACTATCCTTTTCCCGAATTGACACTGAAATTACACTGTAAATAGTCGTTCGATTAGTTTTTTGCCGTTGCAGCA

>scaffold88.1.188263

AATTCTCGAATTCTCTGGCAATAGAAATGATAAATGAGTTTTTTTTAATATCTATTTTATTTTTATTGATGATAATGGACGAAAATTCTGATAATATTCC**[A/G]**CTGTGTTTCTATTAAAAATGAATCTTATCACTTTGAGAAAGTGAAATTAATTATTTTCGCTTTTTTTAGATATAATTTTCAAATTTATCACACTATTGTA

>scaffold88.1.212994

GAGAAATTTAATAGAGAAGTTTAACAATAAGAATTGGAATTGAAATAGAAATAGAAATTCTAAATGAACAATTTAGAAAAAAATTATTTCTTTATTTTTC**[A/G]**TAAAAAAGTTTCGTACTTTTGATAAAATCAAATTTATTTTGATTTTAAATAAAAGAAATATATTTTTAATAATATAGATATACAGATTTAATATAGATAT

>scaffold88.1.61311

ACAATCATAGTTATATAAAAAAAATTATAAAATAATATTTTCTTTTTATCCGTATAATCCATTGAGCTCTCTCTCATAACCGTTCTATCAAATCTATATT**[A/G]**CCTACTAATTTAGCATTATTAGATTAACATCATTCTAATTATATAAATTTCTAAAAATATTTTCTTTTTATTACAGAAATACTGTTTTTAAACGGATCAT

>scaffold88.1.360089

TTGTATGATTTTGACACGTGTAAAGAACACGAAGTGGTGACTTCGACATGAAAAAACATTAAGCAAATTTGTTAAAAATTTACAGTCTCATATTTAACGG**[C/T]**AGCAGTTAGAGCCATACAGATGGAATAATTGAAGTACGTAAATAGTTGTGCAACGAAGCGTTGAAACATTGTGTTACGATCACTTTTACATACTTCACGA

>scaffold88.1.367394

ATTTTCCATTTTATATTGTTCATTGATATCTAAAGTAATTAATAATATCGTAAAAATCTTAATTCCAAATCAAAATTAATAAACAAAATTTATATTTATG**[C/T]**AAAATATTTGGTGAAATCTTCATCGATTTTCAATAAACCATATATTCTAAACAAAATAATGCGGAGGAGTAATGTGAAAAATAGACCTAAATCTTCGACT

>scaffold88.1.493531

AATTCATAATTTAATAGATTCCTACAAATAATCACATAAAATTTTCTAACTTTATGAAAAGTTAAACGATGCAATTAAATATTTTAATTATATTTTGATT**[C/G]**ATAAAAAAGAACAAATGATTGAGAAACACTGGACTAAATGAATGCTGAGCCTTTGTATTTGAGCCTCTGAGCTTGGAACGTATGTAAATTCGATCCTTTA

>scaffold88.1.96424

ATTTGATGGTAACTTTAACATATTCTGAATTTTATTTCTACATGTTTTTAAAGGTCTTCGTGTGTTTCGTCACTTTCAATTTTGTGTAAAATCATATCGA**[C/T]**GATTGATTAATATTGTAATTTCAATTTTGGAACGATATCTTTCACGATAAATATCAAAATTCTTTCGATTTGATTTTTCAGAAAATATGAAAAAATATCG

>scaffold88.1.145174

GAGGTAACAGAGAAAATATGTGTAATTATTGCTGTGAAATTTCTTTTATGTTTATCGTCCTTTGCTTTTTAAGTGAAATTCCATGCATAAAAAAGAGCTT**[G/A]**TCTTCAATCGAATGTTCATCCTTCTCCTCGAATGCAATTTATTTTTTATTTCATCCGTTGTGTGGTCAGAGAAACGAAATTTCGTTTAGATCTCTCTTCT

>scaffold88.1.379827

AGTTCAAGGATACAACGGATAAAAATAAATTGAAAAGATCTAATTAATGTAACTATACGAATTCACTTTGACAGAGAAACTTTGACAGAGCATCATTTTC**[G/A]**TGGAAGTTAAGGGCAAGCAGACGATGTCTAAATTAATTGTACATATGATACGAGCCGGTGGATTTGCCTTAGACTTAAATTTTTGTTCAAAAAGATACAG

>scaffold88.1.553657

AAAAATAAAACCTATAACTTTGAATATGATTTAGAAGCATTCTACAGTTCAAATCGACTTGTTCAAGGCTTTTATTTTCGACATTTATCACAGTTGTCGA**[G/T]**CACCATGCGAATATAAAGTATTCAAAGCATGATTTTCATGCTGAATATTTCGATCAAAATTTTAAAAACTATTGTTAATGCCTATTATATCTTCGAATTA

>scaffold88.1.96319

ACTTATTGTCTCGTGGCTATTTCGCAAGAAACTCAAGAAACGTTAACTGAAAGGGTTCTACCGATACTTGAAAAGTTTGTTGCTAAATGTTTATTTCATC**[G/A]**CCTGATTTGATGGTAACTTTAACATATTCTGAATTTTATTTCTACATGTTTTTAAAGGTCTTCGTGTGTTTCGTCACTTTCAATTTTGTGTAAAATCATA

>scaffold88.1.110873

ATGTGGATATAACGTATGGCTACCTTATTTTTCATGAATGTATTTTAATTTCGAAATTACGTAGAAAAAGGTATTCGTAATAATTTTATTTCTGGATTAC**[T/G]**ACACGTACACGTTTTATCATAAAATTAATTTGAAAGATGTATCATATATATATTAATGAAGATAATTCTCCAGATCAATCTATTATATATTACGATTAGC

>scaffold88.1.155373

AATCGAAAGATAAACGTAATACTCTTTACGATGTTTACCATTCGAGTAAAGATGTTCCGGATTTTCTACACGTATAAAAAGAAATCTTGGAAATCAACGA**[T/C]**GGGATGGGACCCGTCCTCCCATGTTGTAATAAGAAACACAATTTTAGTGGAATTTATTTAATTGAAAAGTTTAAATTGTATTATTTAATCTTTTCAACCT

>scaffold90.1.185569

TTCTAGAATTTATAATTTACTCGAGATTATAAATTTGATCAAAATAAATTTAATCGATATTTTTCAACGAGAATTATCTTTATTATTTTATGTTTATTTA**[C/G]**AATGATAAAGGAACGTTTAATAAGATTTAGAAATTTAGATAAAATAATCAGAAATTTCCAAATTATGATTATCCGTTCATAGTAATTCCTAATGAGTTTT

>scaffold90.1.215468

ATCCCGAGAAAAATATATTCGTCGATATATCGTTTACGTAAAATACGTAAAATTTGCATTTGCATAAAAATCCTCAGTCCATAATCCAATATGCATAAAA**[C/T]**GACTTTAAACGTAACGCGATGCGACAACAAAGTTTACACGTTCCCCTCAGCTTAGAACCTTTTAAGGTAACCACTCGATTACTTAAGAGAGGACTTATTA

>scaffold90.1.132451

TTTCCTCTTGATTCGATTTTCTCGAACTTTTTTTTTTATTTAGAGACCGAAATAAAATTTCTCTTATATATTTTCACTTTAAACTTTTTCGAGATTCGAT**[G/C]**TTTAATTTGTATGTAAATAAATTACAAAATACATATTGTAAATTATATCTTTTATGTATTAATTTCTTCAGAAAACGGTTTATTTTTTCCTTTTTTTTTG

>scaffold90.1.156941

AGGCGAATCGGTTGAAAAATAGAATGCTATGGGTATCCAATATAAAATCGAATTCCGTGCAAGCAATTGGAGAATTGTTTCAATTTAAAATTTAAAATTT**[G/A]**TTGGTAAAGATATAGTTTAGCTATTTTCGTGAAGAACATACAAAATGTGAAAAAAAGCGAAGAAAAATAATCGTTATTATCAGGATTATACTAAAAAGGA

>scaffold90.1.200582

TAAATATCGATAAATCCCTCGACTAAATGGATAAAGCAATTATCTAAAAATTCCAAAGAATATATTAAAAAACTGATAAATCGTTAAATAAGATAACTTT**[G/C]**ATTTAAACTATCGGTAAAAATTTATTCCAGGATCGATTAAAATTGGAACACGCATCTTATTGTTCGAGATCGAAAGATTATATGTGAAAATGTAATATAT

>scaffold90.1.207117

TTGTCACCGATAAGTTTAAATATTTTATCACAATGGAACCACAATGCGGTAACTTGAAATTTTCATGTATATATATATACATATATATCAATGTGAACTA**[G/C]**TATCGGAGCGGATTTCATAAATATTAATATCAACGAAAGAAAATTCATTCCTCTCCACCACAATTTTGTTCAGAGACATAAATATATTTCCGCATGTACA

>scaffold90.1.153481

AATATTAAATTTGTTGTACAATTTGAATCAATTTAAATGAATATCAATTTATATTTAGAGATTCTCTAAGGGTAAAAGTTCGACGATGCAAACTTGAAGG**[T/C]**AAAAATAAATAAAGTTCATTTTATAAAATTTTAATAAATCACGAAAATTATACAAATTTTAATATCTATTTCTTTATGATCTTTTTGTTAATAATTAACA

>scaffold90.1.24409

AATATAAATTAATTATTATTCTACGCGTGAAAATAATGATAATAATGAATATTTGAACAAGATAACACGTAAAATAACTTTCTATCAATAATTTACTGAT**[A/G]**CTCCTTCACGAGGAACAATTTTTAACTTGATTTTCTCGAAAACGAAGGTTCAGACAAAAAAATAATATCCTTCTTTTTCACATCGAATCATCCTTTAGTT

>scaffold91.1.174766

CTGATTTGTTATATTATATCTATAATATATTGTTGTTATTTTAACTATTATTTAATATTAAATAAATAAAAGAGCATTGAGAAATTGTTGTGTGTAAATC**[A/G]**TGCGAAATGTTAATATAATATCTATCACTGTAAATTAAATAAAATTACATCTTGATATCTGGAATTTTTTTTAATAAATATTGCATTTTTATAAATGTTC

>scaffold91.1.206027

GTATTTTTTTTCCTTATTATTTATTCGTAACTAATTGATCCATTCTTCACTATAACCTTTATAAATCGTTTGACATTACAACTTTTTACGATTATTCGAT**[A/G]**CTTTAAATAATAAATTATCGAAGTAACTTATTGAAATAACTTTAACTATTAAAATTTTGTCCATCTCTGATTTGTCGATACGCATTTAATTATCGAGTCT

>scaffold91.1.235866

TCCATTGCACAGATTAACTAACGCAATAAAATTTGTACGATATAAAAAGCTTGAATTTAAAATTTAGTTCAACGATTCGATAATCTAGTAGAAATTCAGC**[A/G]**TGAATGTATATCTATTTTTTAAGAAAAAAGTTATTGCAACCTACATAGCTTAATTGGCGCAGCAATTTCCTAACACGCGAAAAACTCGGATTTAAACTCT

>scaffold91.1.155824

AATACTATAACGATTGAAAATCAAAAAGTCTACAGTTTACAACTTGGACCAATTCAATTAGTGATCCAGGTAGTTATTCTCCAAACGAGATCCTCCATCA**[C/T]**GCAAACCATAAAACAATCTCGAAATGTTCCGTGTGGCTTGTAATTGCGAGATTTCCAATGGCGGTTCGCATAAAGCAACATTAATTGTCCAATCAAGCGG

>scaffold91.1.169729

TCGCATGAGTAATTTAAGGCGATAGAATTCAATGAATCGCCCAATATATGGACCATAATCGAATTTACAACCAATCATAACAGATTTAGCATTAACAAAA**[C/G]**TTATGGAAGCCATTTCATAGACTCGACATTAGGGTTCCCCACTTTGGCCAAGTTTTATCAAAAGCATCTATTCGATGCATCCTCGAGGAACGAAAAATTC

>scaffold91.1.127152

GATCGATAAGAAGGGATAAAAGAATGCAATGCACAAATAGGTGCCGATAAGTTATATTAATCATCCCCGATCCTGTTATTTACAAGAAGCCATAATAATC**[G/A]**TTTTTCTTTACACGATAACAGAGATAATTTCAAAAAATTTCAAATACAACGCATATTTCTTTATAATATATTTGATATAACGATCAATATTTACACATTA

>scaffold91.1.135403

TAATTTATCAACCGTAAAAAATATTATAATTGTTCAAACTGAATAAATATTAACAATAAAATTTTCAGTTGCAGGAAAGAATAAACAATGATTACATTAA**[G/C]**TAAATAAAAAATATTATTAATTAAATATTAATTCAATCTTGAAAATATTAAAAGATTAAACATTAAACGTATTTTAAATCGTACGTTTCATCAGTAACAG

>scaffold91.1.146192

CGATATTAAAATATATTTTTCTTTATTCCTAATATTTAGATCTTTAACTTATAATCTAATAATCTTTATATGGAAAATACTGTCAAGAATCAAAGTACTT**[G/A]**TTTGCAAAAGATTAAAAACAATGTGTTTTCCTTCGTTCAAAATATCTGTTCGTCGTGCAGGAAAAGAGGGAAGAAAGAGGAAGATATCTCGATTAATTTA

>scaffold91.1.21688

GTTTTTAACTTTAAATAGATCCAATATATTTTTAAACATTTTTTATATTGATCTATACTTATTAAAATCTTATATTCTAAATTTTTTAATTCCTGAAAAT**[G/A]**CTAATCTCTTCAAATAATAGTATCATATTTATAATGTCAAAAAATTTGACTAATTTAACCTGTCATCATCTCATCTTTCGATTTTTCTAATAATCTGTTT

>scaffold91.1.62684

TTCTTTTTGAGAACAAAAGATAAGTTGGACGCGCGAAATGAAATTTATTCATGCGAGACATTATTTTTAACATGTTAACATAACACGTTCCTGTTCTTGT**[G/A]**CATCATTAGTGCCTTAACACGTACCATTTGTACAATATGAATTATATTTCTTTCTTTTCTTGTACAAAGATTTATGAAAAATATTAATATTTAGAAATTT

>scaffold91.1.174483

TAAAAATTTCAATGAATGAAATTTCATTCATATCGGACAAAATATCTTAGCACAGATTAAAGTTCATATTCTACGAGGAAATAGAGTCAAGACACTGGAA**[T/C]**AGAAGGTAAAAGCACGATTTAGAGTTAGATTTGAATTTTGAACGAACAGACAAACTATTTGGATAGCCACCAAAGGAAATGCCAGTTTCTCTTGTTAGAA

>scaffold91.1.221074

ATTATTTGTACTGTTGAGTTATTTTCTTTTTATAAATCGTTATAAAATATTCGCGAAAGATCCACATTAATCCAGTTTCTAAAGCTTTAAAATAATAATC**[G/A]**TTATTTGAGACACGAAATCAATTAATTCAAAAACTTTTACCAGAAATTGGGATACAATAAACAACTTGTAGCACGAACTTAGCACAACATCAACAGTTCT

>scaffold92.1.237425

ATTACATTTTGAATCACTTCTGCCTTTAAAATTGCTTTCGATTCAAACAATTTGAGAAAAATACCCGTATTGTGGTAAATCTACCAATTGTAACGAACAA**[C/T]**GAGAGAATGATAAAATATAATAGGATTATTGTAAATTATTAAAGTGAAATATTATTTCATTCGAGAAAATAATCCAGAAAAAAAATCATTTCAAATATCT

>scaffold92.1.36210

AACTTTCATTAATTATTTCCATCATCGAAGGATATCAAAAGGTTTGATTATTCAATTTAATTAATTCAGGATGAATTATGGACGAATAATTATTACCGGA**[C/T]**GTTATAACACGAATGATCGACACAATTGTGCGTACTATATTATATAATAATGTTGATTTCGTTATAAGTTTCCGATTTCGAAATAAAGTTGCTCGAATTC

>scaffold92.1.27715

TATTTAATTTCAAAGAACAAAACTACGAAATCGATTGATTATGTATCAACAAAAAATATGCATGTTAAAAAAATCAATTTTAATTTATTATAATCCAAGC**[G/A]**TTGTCCTAATTACCTAATTAGTCTAAATCTTTTTGATAAAGAAAATTACTTTTCCAAAATTATTTTATACCATACAAGTTTGACATACAACGTCTTTCTG

>scaffold92.1.55411

TTCGTATAATCCTTCCTTTTTTTTTTACAGATCCTTCCTTTTACAAAACCAGCATAATTATCTGTGAAATTAGTTGAAAGAAGGTGAAGAAAATGTTTCT**[G/A]**TTAGCATATTATCTATCAAATAGAAAAATCCACAATACACGTATCTTAAATACATAAGCAAATAGATACTTAATTTTTCGAAAAACTTAAAAGGAAAAAG

>scaffold92.1.69160

AGGTATATTAAGAGACAATGTTTTTTCAAGTATTGTAATTTTAATTATTTTTATATTGGCGTCGTTTAAATAACGATAAGTCATGTTTTTTTCTTCGTTT**[G/C]**TTATTTATAAGTATATATTTTAAGCTATACACGGATACTTAATAGCATATACGTATTGTTATGCTAAAAATTAAATACGTATTAAACGTATTAAATTATA

>scaffold94.1.146375

ATATCGATAACAATTGAATAATACAAAAGATAATACTAGCTTTTTTATAATTAATTAATTTATGTATTAAACTCCACTTTGTCCATATTTAAAATTATTC**[A/G]**TTTTCCAAAAATCTTAATTGTATAATAATCCATTATAGGCTTTATTTCATAAACACAAATATTAATCTATAATAATATCACCAAAATACATTAGATGAAA

>scaffold94.1.182753

TTTCTTGTTATCTTTATATTAAATTTTGATTTTACATAAAAAGAATCATTTAAAAAAAAATTAATGAAAGTAAATTAGAAAAGAAGGTATAATAAAGAAT**[A/G]**CTTTAAATTTAAAAATTCTTTTCCCCATCAATATTTGCAATTAAATCAAATTATATTTCAATAATTATCCATTAAGGTCAAATGTAAAATTGTAAATATA

>scaffold94.1.121163

CATATGATTAAAACTTTTTCCATGGAACTAAATATTTCAAACTAATAATTGGAATGAACTAGGTAATTAAACTAAAAGTGAAATTCTGAATTTCAGAAAA**[C/T]**AGACTATATTACAATTTACAATATTCAATATTATATTCTCATTTAAAAAAAGTTTCCAAACGGAATCACAGAATAATCCTATCAATTAATCCTTCAATTA

>scaffold94.1.146298

ATGATGATGAAATTGTACAAAAGCAATTATCCTTGTGCCAGTTTTACTATTGCTAACTATTTGAAAATCAATAGTAAATATCGATAACAATTGAATAATA**[C/G]**AAAAGATAATACTAGCTTTTTTATAATTAATTAATTTATGTATTAAACTCCACTTTGTCCATATTTAAAATTATTCATTTTCCAAAAATCTTAATTGTAT

>scaffold94.1.56212

CCTCAGCTGATCCCCATTCAACCCATTCTTACGATACGTAGTATGCTATTTTTAATGCTGAGATTGATATTTCTAACATTCGTCGAAACGGCTGATTTTA**[C/T]**GGTTGCCTTTCGAGTCGAAAGCTTGAATAAGTAAAATATTAATCTTCTCTGTTCTTCCGTTTCGAATTCTTCCATTTTCAATTTCCGCGTTTCCTTCGAT

>scaffold94.1.123634

TAAAACTCGATAAACTTTACGAAACATCGTTAAGTCGGATAAAAGTAACTTTTCAGCAACTCATTTTATTATCTAATATATTAAACGATCGTCCAACTTC**[G/A]**TTCGAGTGGTATACATTTATATACGAAGAGAGAAAAAAAATCCGATACGCGTAAGAGGTTTACGATCCTTTCTCGCTTTTATGAAACGGAATTAAAACGC

>scaffold94.1.176466

TCACATTATTTCAGAAGAAGAAGAAGAAGATTAAAATTTTCCAATATATTATAAATTAAGTGTTATTATTTTAGCTTGAAAATAGAAATATATGCAAAAT**[G/A]**CCACAAATATAATAATAATAATAACAAATATTCAATTAAATCAAAATATCATCAAAGTATCAACAAAGTAAGTTTGTTTAATTGTTCAAAATTTGCATGC

>scaffold94.1.62037

TAGTATTACAAAATTAATTATTTTGAACAAATAACAAGTTACATATTAACAAATACTGAATAATTTATTTCATGAAGTTACAAATAAATTATTCAAATAC**[G/A]**TTCATTTACTTTTCGTGAATGAAAAATGAAATGCTTCGTGAAATGTTTAACAATCATTTCAAAAATAAATTATAAATAATTTTAAAAGCAATGCAATTTA

>scaffold94.1.67170

AGAGATTATTATGTATAACAAATCAAATAAGAATCGTTTAAAATAACTTTTCTCATATTCTACTTGTTATTATATTGTTTTCTTTTCTTTCAATTTTATT**[G/A]**CGTTAATAACTTTCAGATAATCTTTAGATTTCTTATTATCAACAAATTTGAGATTTTCATTTCAAATCCCTCATAAATTCATCGAATTTACAAATCTCAA

>scaffold94.1.175315

GATGTTGAACATTTTTGAATGGAAGAGGAATTTAGGGTATATAGGAGATGTCATGTGTAGATAATTTATGTCTGATTATTCGGCTTGCTTTAATATTTAA**[T/C]**GTCAATGATTCAAATATATTTAATGATAAAAGTCACATTCTTATATCGCGAATTCTTATAATAAATTTATATGATAATTTATATGAATTGAATTATTATA

>scaffold94.1.175342

GAATTTAGGGTATATAGGAGATGTCATGTGTAGATAATTTATGTCTGATTATTCGGCTTGCTTTAATATTTAATGTCAATGATTCAAATATATTTAATGA**[T/C]**AAAAGTCACATTCTTATATCGCGAATTCTTATAATAAATTTATATGATAATTTATATGAATTGAATTATTATAGAATTGAAATTAAGTTGTACTCTATGT

>scaffold94.1.205581

AAAATAATTGATTATAAATATTACAATATAAACGATACTAAATTAAATATCCTTTCAAACCACGATTAACAACAAATCTTAAAAGATAAAAATTGCAAGG**[T/C]**ACGAGATTTTAATTTTAAAAAGATAGAAATATCCATGGTATCGTATCATCCATTTCTAAAATTCTTGGGTTAGAATCAGAAAATTTCATTCCAACCTGGA

>scaffold96.1.254916

GCTGTACATGACCTCTCGTTTACAACGAGATTTTATACTGCATAGTTAGTCATCCGGACATTTTTTGTTTCAGTAGATAAATTTTTTTTAACTAAAACAT**[A/G]**CAAATTATTAATAAATAATTATAAAAATTTTAATATAAAATTAGTGAATGTAGCGATATAAAAATATTGTTTTTTACTATGCGTTATTATTCAAACAAAT

>scaffold96.1.196137

AAATACGAAGATGCGTCGTTCTTCATTGAAGCAAAGATAAAAATGATTCTTTGTTCAAAATGAATACCAGAACGGTAGAAATATTTTGTCCATGACAAAA**[C/T]**GATGTATATAGAATGATTTTTCCAAAGAGTTGTTATAAACGTACGTGGAATGATTCAAATTCGAATGACGATCAAACGTTTTAGTTTTAATCGTTTGATT

>scaffold96.1.254970

CGGACATTTTTTGTTTCAGTAGATAAATTTTTTTTAACTAAAACATACAAATTATTAATAAATAATTATAAAAATTTTAATATAAAATTAGTGAATGTAG**[C/T]**GATATAAAAATATTGTTTTTTACTATGCGTTATTATTCAAACAAATTTATAATAACTTTCAACATTTTATATCAAGAATAATATTTCTACCCATCTCAAC

>scaffold96.1.255773

CAAACGTATATACATACGCGAGATCGTAAAATTGATCGAACCGTTAAGATTCACAATCGAAATATGTAAAAAAAAATATTTTAAGACTGTTCAGAATTAA**[C/T]**GTCAATTCAGAATTAGACTTTCGGAATTACTATATTATGGATATACGTAAAATTATAAATTAAAATGTTAATTGATATAACTCGAAGTATACTTACTCTT

>scaffold96.1.506597

TATTATTTGTAAATATAAATACGATACGTTCATTATATAATATTTAGCCTCTTATAATTTAAAATTTTAAATAATTTTAAATAATCTTTAGAAATTTAAG**[C/T]**GATTTTGAATAAAATTTTTCTTAAATTTTTATTGATACTTTCTGATAATTAAAAAACATACTATTAAGATATTTGTATCATTATTTGCATCATATATATT

>scaffold96.1.515672

AAATTCAATTATAATTTAATTAATTTATTTTTTTCAAATAACGATACTTTTCTTAAATTTAAATTTTTCAAATAATTGTTATAATTGTATACGATTTTAA**[C/T]**AATTTTTTAATTATATATTTTTACATAAAAATTCTATGATAAAATTTTCTGTAATAATAATATTTTCATAAAATATTATATAATAATATATTATAACTTT

>scaffold96.1.394977

TAATGAAATGCAATTTCCTTTAACAAAATTTTATTTTTACCGTCATTATTTAAAAAGATTTTAATTTCGTATTCCGTCGTCGAAATTTTGGATTTCTTTT**[G/A]**TTTAAAAATTTCGATCTTTTGGCTGCCAAAAAATTCCAAGGAAAATGAAAAATTTTACACGATAAATAAATTCCAGATGAATAAAATCATTTTAAAATAA

>scaffold96.1.491625

GTCGTAGCTTTTAGGGATTACGTTTGAATAATTTAGGATTATTTTTTTATCGAGAGGGATAACGCTTCTTTATCTCTTAACTCTTCTTTTTCGTTTATTC**[G/A]**TTCATATAGGAATCCTTTCGGTTTTAAAATTTAAAAAAGAATCTTACGTAAATCGAATATTTCATCGAAATTAGAGAAATATTTATTAATCACGATCTAT

>scaffold96.1.800174

ATGGAAAATTTGTAAAAATTTTTCCCTTAAAATTATTTTCTATATACTAGTTTTTGAAAAAAAGAGAAATAATAATTGAGAAAAATTCAATTCAAAATTC**[G/A]**TTTTGTCAAAATATATCGAAATATTGAGTTTTTTTAATTCAAAGATTCAGATACATTTATTATCATATATCTGGCTGAATTTCATTTAAATTAAACTCTT

>scaffold96.1.228991

AAAATTTATCTTCGAGAATTATTTTCCACTAAAATAGACATTGAGAGTGAAAATTATTATGTTATCGAACATACTTGCACTTTAAATAAGTAGATTCCAA**[T/C]**AACACGCGTCACCACTATAGTTTCTCCTCCCCCGAAAAATATCTCCCAAGGTGATTCCCTACTATTCCTACGTGTACCTCGAAATACCTCGAAGCTCCCT

>scaffold96.1.359173

ATAGGGCCTGATCTGCTTGATGTTCCACTCGAATTATTGCTTTCTAGGCCATCTAAAATATATATATATAAAAAAAAAATTTTTTTCTATTCTAACAAAA**[T/C]**AAAAATAAAATTATCATCAACAAAATGCAAAGTATTTATCTGAAATTATTATATATAAATAGTCCAGGTAAATAAAAGGATATGTGTTTCAGCTAAATCT

>scaffold102.1.81571

GCGAGCATTGCATTATTGATTTGATTCGGGGAGGGAGAATAGAGAATGAGTCTTGTCACTTCAAAAGATGAAAAAGAGGAAAAAGGGAGGAAAATAACAA**[C/G]**AATAATAATGATAATAATTGATAATATTTACGGGCAACGAGTGTGTAAATAATAACACGTAAATACGTAGGCACGTAAGCTGGTTTTAATTTGTAGAGAG

>scaffold102.1.235736

CTTGTTTTTGAAGATATTAAAAAATTTTTTAAATACAAGTTGAATGATTTCGAGGTGGGTGTAGTATGATAAATTAATTTTTTTATAAGTGAATGCACAG**[T/C]**GATCATAAGAAAATCGATTCCGTTTTATTGAAAAGAATTTTATATTTTTTAATACACGTATAGATATAGCACATTATTCTCTATATAAAAATATTGAAAT

>scaffold103.1.750291

GTCACATCGTCGGTTCTTTTCGTGCCGTCTCTCTGCTATCCATAATACGTACTTAGTTATTTAAGTAGTAACTGTACAGCTCGACTGCTCGTTTTGCTTT**[A/G]**CAGAGAAACCTTATTTTCTAATTATATTCCTCGAAGACTCTTCCTTCCTTTTTGCTGCATTTCTCCTCTCTTTTTCTCCCTCCGTTTCTTTTCCCTGTTC

>scaffold103.1.760704

AAGAAAAACCCAACTCCGTTTTCCTTTTAAGGGGAATTTTTCCTTTTTTTTTTTAAGTTTTGAACGCCGAGAGGATGATGAAAAGTAATATTATAGGAAG**[C/T]**AAATATTAATCCATCGTTTTAGTAAAATTTTAATCTGTAAGTTTTAACGAAGCTCGGGGAAAGAGGAAGAGGAGGAGGAGGAGGATGTATTTTAATTCCA

>scaffold103.1.554300

GCGTGGAAAGGATGGAAATCATCTCCTCTTGGAATTTTAATTCCAATTATTATAATCTAAGAAAAGTATTTCTTACGAGATTTTTCTTTCCCTGATATTC**[G/A]**TTTATTTCTTCGTAAAATAATTTTAGAAACAATTGACGAAATTTCAATTCTTCCATCTTGTTATTTATTAAATATTTTCTTTTTATTTCTTTTTAATATT

>scaffold103.1.767333

TAACCACTTATTGTATGGATGTTTTATATCAATTTATATATTGTTCATTCAGAGGTATAGCGTGGTCGTAAATTATTGTACGAGTCGGTGATAAGATTAA**[T/C]**GTTACGATGAATTTTTTTTTCTTTTTATGTCGTTGGGACATTTTTAGTTTTTTCGCTAGAATAATTTTACGACCTCGCCATTCTGGGTGTACTATTAACT

>scaffold104.1.506005

TATAATTATATTATAATGCAATTTTATATGGAATAAAAGCTTTTTGTTATAAAAAAAAAATTAAAAAAACGATTAAATTATAGAAAAAGTAATATTGTAA**[C/T]**GTTTATATAAACTATAAAAATTTATATGATTTATTTTTCACGAGAAACAGAAATTTTTTTATGAAATTCTATAATGAAATGAAAAAATATTTTCTTTGTT

>scaffold104.1.708494

AAATAATTTTAAATACTTTTTGACGTAGATTTGATATTACTATAAAATAATTTTGTTTTAATTTATTCAGATATTGTATTTAATCAAGAGGATGATTAGA**[C/T]**GTTCATTATAACGTGGATCTTTTCTTACTCATTTAGTTATCTAGTTCATCGTTTAATGATTTCTCTAGTAAAATAATTTAACCGTAAGTGGAAAATGAAG

>scaffold104.1.735223

CTAAAATTCCAGTTAGCATATCTACAGTTTATCCACTAATCGATACTGGATATAAGACTGTCGTATCTTCGACGCCTGTTCCTGTGACTATAAGTCAGGA**[C/T]**AAATATCGTGCTCAGACTCAATCCAAATTTGATTTTGGAATAAAAACTACTCAACAGGGCGAATATATTACTAGCACACCACCAACAGTGTTCAAGATAT

>scaffold104.1.927925

AGAATTGAATTTTTCTAGAATTCTTGTGGACATTCATTTGGAATTCTATTTTAAAATTCCAGAAAATATTTTTTCATTCGATACAATACGATAAAAATCA**[C/T]**GTATTTTATAAAACTGTTTTTATAAAGTACATATCGCGACCTTTGGATCTTGGAGCGATCTAAAAATCTATCTCTATCTCAAAGATACATGATGTCACTC

>scaffold104.1.982313

TTCCAAATCTTCGTACATCTTGATATTTTGATTCTGATAAAATATAAATTTAAATTTTTTTCTTTCTTCGAGCGATAATTATTTATTTAAATGGGAATTG**[C/T]**ATTTTTGAAATATATTTGAAATTCGCGATATATTTCATAGAATTGGATGTTTCATTTTTTTGACAATTCTTGGTTTTTAAATATTTTGGAGATATTTCTT

>scaffold104.1.989435

AATTAATTCGCATCCAATTTGCGATCAGAAAATGATTCTTACTAACAATTACCGACATTGATTAATTGTTTTAAGTAATAATTAATTCGATTCTTGTGCG**[C/T]**GGGAGTATAATTATGAGAATTGAGAAGGATAATTGACGCGACTTCCATTCGTTTCTTTTCGTTCGAACTCTCGTTATCGTTACATTGATCTAATCGAAGA

>scaffold104.1.662450

ATAATTTAAGAAAAGATTCATGCTGGAACAGGATTTAGTAGAGAATTGATGATTTGTATTGATGAAGAAATGAATAAGTTGAATAGGTTTTGTAATTTCC**[G/A]**TTAAATGAACGAAAAATTTTTCATTAAAATGAGGGAGAAAGAAAAGAGAAATAAGATCGGTAAGTTGAAGTGTATAAGTCAAGGAATTTAGAATCACTGC

>scaffold104.1.686956

TAATTCTTTTGAAAAAAAAATAGTTTTTCTCTTTATCAGCAAATGATAATAACGAGGAAAATTATTTATTATTATTATAAAAATTGGTCATAAACATTTA**[G/C]**AAGAAAAAAAAATATTTCTATTTCAATTTTTCCAAAATCAAAGATATCATTGAAATTATATAAAACGAAATACATCGAATGAAAATGAAGAACAAATTTT

>scaffold104.1.886732

GGTTAAATAAATATTTTAAAATACGAAACGTACATAATAAAATTTAAAAGATAATTCTTTCATTAAGAATAAAAGATACAAACTATCATTAAAGAAGAAC**[G/A]**TGAAGAAGAAAGACATTCTACAATAAAGACGTGGCCATTCTTCCATTCTTCGACACGAGTCCAGCATTTTTCTTGAAACGTTTGCAATACGTGCCACGTT

>scaffold104.1.979555

TGAAATTCAGGGGAACGGGAACGATATCGTTCAACCTTCGAATTAAAATTCACGATTAAATTTATTCGCGTTTAACTTGCTCTCTCTTTTTTTTAAAATT**[G/C]**TTTAAATTTTCAGTAATATTCTTGTAAACTCATAAGCTCCTTTCGAAGTCGGTTGCGTTCATTTGTTTGGCGGAAAGATCAGTTAGAATTGTTACTATTA

>scaffold104.1.523624

ATTTTAATTTTATTTTAAATAAATATACGTTTACACGATAAAAAGAATTTTTTTTCTCGTTAATCGTGATAAATAAAATTGAAAAGGGGGAAGGAGGAAA**[T/C]**AAAAAGTAACGGTGAAAGCTGTTATCGTGCGAATGAAAAATAAAAACTGATAAATAATTTTCCAGGAAGTGAAGTGCTTTTAAAAATTTTTTTCCAATCA

>scaffold104.1.713889

ATTTGCGATATAATAAAATGAAGTTACAGTTAACATATATCATATGAAAAAAAAGTTGAATTATATTTTGAAGATTAAATTAAAAATTTTGAAAATTAAA**[T/G]**AAGCATAAAAATTTATTTATTCTTTTATCATAAAATATAGAATTCTAAAATTCTAAATAAATAAATGAATATTTAATATTCAATAACTGTACATCGTATT

>scaffold105.1.113579

CTTGACCTGCATTTCATTCAGAAATAATATCGTCACGTGCAGCATGTGCACGATCGCGAGGTAAGACCATTTATCTTTAAAAACGTGCAATCTTTCGTTT**[C/G]**TAACGAGAAATATTTTATAATTTTTGTTTCGTTTTTTTTCCCCCCGTAGAATATTTCTTGAACACGTTACCCTTTCGAACGTGGAATATTTTTTAGTTCA

>scaffold105.1.170217

TTATCGCGGTATCTATGACTAATGACTTTATATATGCTCATATTTTTAACTTCTAATTGCAAGGCCAATCTCTTATTTGTTTTTCAACATCAAAAGTTTA**[C/G]**TCGATGCTACGTGAAGAGAATCACGAGTGCATGCAGTTTGTTTTGAATGGTACTTTTACTTGGCTACAGCCGTTCTCTTTAATTTCTACTTCGAATATAG

>scaffold105.1.216528

TCTAAAGAATTGTTCAAACACTTGCAACTTAGTTGTAGTTTTTCCTTATTATCGGATGAGTCGGTGAATAATTAGAAAAAATTTCGTTTTTTATTTTCAA**[C/T]**GCATTTTTGATTAATTTTATCGTACATTTTTTTATATTATAACATTTGTTCATATGATAATTAGAATGGACGAATATTTTTATAATTCCATCGAAAGAAT

>scaffold105.1.269781

GGGAACAAGAATTTTTATTAACCCACCGTTAATATTTCGCATCGAAAAGAAATCGAGCGGGAAAATGGTAAATTTTAATTTCCACGATTCTCTCGTAAAT**[C/G]**TTGGTTGGCACAACTTGTTTCTATACAACGTGAAATAAATGCATTACAGATAAGATTTGAACGAATGTTTTAAAATTATCGTAAATGTAACATAAGTCTG

>scaffold105.1.55255

ATTTTTATATAGCTCAATAAGAATACAGAATGATCGATTTTACGTTCGTTTTAACAAGAAAAAAAAAGAAAAAACTCTCATTAAAAATATGGAAGTTATT**[C/G]**TAACGATTTTCTCAATGAAATTTGGGTTAAACATTAGGCATGATTATTTATTCGCGAAATAAGAACAAACGGGATCTGATATCGCGTGAAAAAAAGATGA

>scaffold105.1.7384

TTTATCGCAGTATTAACAGTGACTCGAATGTAGTCAACAATAACTATCGAAAAATTACACGATTGAAACGATAAAATTCGACGCGAGTTTATAATTCCTA**[C/T]**GCGTTTATGGCGATGAAACAAAGAAAAAAACCGTTTGTTCGGAATTCTCTATCTTCGATCTACACCTTGACTCGCAATCTGTACCCCTACGCAAGGATTG

>scaffold105.1.230622

TTGCACGATGCGAGTTAAAAATTATCGCTTCTAATAAATTTTAGAACAATTTTTGCAAGTTTATAACCCGTCTATAATTACAATTCGAAATTACAAAATT**[G/A]**CGAGAGAAAGATTTTTTTTTACAAATTTCTATGCTTTCAATTTGTCAAGATTCCGACCCAACTTCGAAAGAGAGAATACATCGTGGAATACTGAACGGAA

>scaffold105.1.7417

TCAACAATAACTATCGAAAAATTACACGATTGAAACGATAAAATTCGACGCGAGTTTATAATTCCTACGCGTTTATGGCGATGAAACAAAGAAAAAAACC**[G/A]**TTTGTTCGGAATTCTCTATCTTCGATCTACACCTTGACTCGCAATCTGTACCCCTACGCAAGGATTGTGAACGCTAGATATTTCAAAGTTAGACGAAGAT

>scaffold105.1.143200

TATTCTTTCGCAGCAAGAGTCCTCAATTAAGAATTCCTTTGTACCACATGCCTGTGACATAAGCTACGTGACTCAGGTCTCATTAACTTAGGGAAATGGA**[T/C]**GTGGAAAAGTGCCGCGTACGTTTCGCAGCTCGTTACATGATCGGGCGCGACTCAAAAAGGCGATACATCTTGGCTTTAACGGGTTTTCGAAGAAATCCAT

>scaffold105.1.79604

AAACCCCATTATTTAAGAAAAAAATTATTGATATTATATATAGAAAAAATTATAAATATGCTATTTTACAAATAAATTCCTTATCTTATTTTAATAAAAA**[T/C]**AAAATATAGATTTTATATTGTAAAAGTCTTAAAAAACTTTTATTAGAATCATTTCCAATATTACCGATCTATCATAAATAAGATTATAACCACAATGTCA

>scaffold108.1.1169130

TATTTCCGAGTTATAAATCGAAAAATCGAAATCCAATAGATATTACGTGAACTTATGTGAACAATATGAAGATTTTCCCCTAAAATTTTTTACTATATTC**[A/G]**TATTATATTTTAGAATAAATTAAATTTAAAAAATTAAATAAAAAGAAGATTAATATTATATAATTATGAAAAACGTAAGAAAAATATAATATCACTCATT

>scaffold108.1.12588

CCTTTGCAATTACATGCAGGTACAAGAATGAATATTTTTGTTAAAGGAAGTTTAAATAGATTTACGAGGTATACTTAACGAGGTAATCAATATGATTATT**[A/G]**CTTATATATTTTTTAAATAATAGAGTTTTGTTCTTTTGAAAGAAGATAAAAATTGACAAGAGTCAATTACTATATCATAGTAATACATAGTACTTTTTGA

>scaffold108.1.608476

ACGTTATAATCACTTACGAAGAATCGATACGATTCGAAATCGATATTTATTTCGTGCTCTCTTCGATTTATTTATTTATTTATTTCCCTCTCTTCTCCTC**[A/G]**TCAATTTTCAAACGCGCACTATCACTCGAAGGAATAAAGAAATTTTTTCCTTTTTCGGTTTTATCTTTCGAGTTAACTAAGAAAGAAAAAGAATCGAAAT

>scaffold108.1.619043

TTTTAAACATTTTATTCATTAAAGTGAAACATTTATTCTCAATTAAAATATTTAATTTTTTCATTCATTTATAGCAAATATTCTGTATTTTGTATATTTT**[A/G]**TCGTCAGCATTAATCTGATATTATATTAATGTCAATATTACTTCGAGAGAGAATCATTTGTTTCATTAGTGATATGAACGTGACAATAAAAACGTAAAAA

>scaffold108.1.1278428

CCAATCATTTAACATACATCTTAATTTAACACAAATCTTAAAGATCCTTTCTTCTTCAACGATTAATTTCATACAATTTCCACGACTTTGACCCAAGGAA**[C/T]**GCTTCATCAACGACCTCGTCAAACTTTCTCTCCTTTCGAGTCCCAACCAGTTCAAAGCGATCTCAATCTCCTAATAACCGTCGATGCAACCTGGTCCATC

>scaffold108.1.1496828

GCAAAAACTCTGCAAACTCTGATCGGAAATATCGATAACCTAGTCGTTTTATTCTGTACGCATTTTTCTATTCATCTTTTGCGTAATTTTTTTTTTAATA**[C/G]**TTTTATCTTATCTATTGTTGTTCTGTAAACCCTTTAGACGACCATGGTGACGAGGATTCGATGCAGGTGTTGAACGAGCTGGAAAAGATCGATGACGATT

>scaffold108.1.2191651

ACGTTTTTTCACTGGATAACCAGTCAAAATATTTAATAAAAATTCTTGAAGAGAATTTCACATCAATCAAATCACATATGTATATGTAGTTTAAAAGAAA**[C/T]**AGATAAATAGTTCAAAGTTTACTAATAGTTCAAATATTAAAATATCATAGCTCTTTTGTCCATTGTTTTAAAATATTCACTTTTCATCATACATATGTGT

>scaffold108.1.2363366

AACGTTATTTTTGACTTGGTAAAAATTATTGTGCATGCGATCCTCTCATCCTCGTGCGCATTTTATCTTGGAACAATTAAATGAAAAATTTCCTGCTTTA**[C/T]**GTCAAGCAAAAGTTGAGTCATCAACTTTTATTATTTTCTAATACTTTCATTAATCCTTGTATTAGAGATTTGCATTATTATATTTTTCTGTTAACTTAAA

>scaffold108.1.2400647

ACTGATCAAAGAATGTAGGATCGTTTCTATCTGCAATTATTCTTATTCTTTTATTATTCCTCGTCAATTCATAAATAATTTGGATCAGGATCAAATCTTT**[C/G]**AGATTTATCCAAGTCTTATCTTTTTTTTCAATATAGATATTATTCGATATATATATACATTTTTTTTATTTTGTATTTTGTAAAAGTGGTTTTCGATTAG

>scaffold108.1.1777444

TTTTCTCAACTTGTAACTACATATAAGTTTTTTTTCTTTAAAAGTTATAAATTTACTTTTGCAATCTCTATACATACAGCAAATTTAATAATACTGATTC**[G/A]**TTCGAAAAATATCTTTTAGTTGAAATATTTTTTAACAAATTCATACCATAAAAGTACGAAAAGTCATGAAAGTCATAAGTCATGAAATATACTTTATACA

>scaffold108.1.2268276

AAAAACTTTTTGTAATTCACAATTTTTTTAACATTTTTCGCATTTTCGATAAAATTAAAAAATTTCTACCAATTATTAATACACGTCACACATGCACGTT**[G/A]**CGTGATAATAATATCTTATTATTTTTTACTGTACTAATTGTACAATTAAAAATGATTAAAAAAACAAATTTGATAAAATATGATAAAACTGTGCAATGCT

>scaffold108.1.2278766

TGTGTAAATTGTGAACGAGGTTCTTTGACAAATTGAATTGAGTTAGATGAGCAGCAATTGGGCGATACGGAGATAAACGAATGGAATTTTTATTTCAATT**[G/A]**TTGTCCTAATCGATTTGTAAATGATTTTAAATGATATTTGAAAGATAAATTATTTTCATATGTATTATTTCGTTTTTTATTTGATTTTAAATTTGATTAC

>scaffold108.1.2326210

ATTAACATCCATCACTTATTATATTAATCGTAATTCCACGATTTTCCAAATTCTCGCCTCTTTTGTTCAACAATCTTCAACAACTTTGATATTTCTCCCT**[G/A]**TCCCAAATCCTATGGCTTTAAATAACTTTGGACAATCCTTAAAGATTCGACGCAACGATTCCGAAATCTTCCCCAACCAGTTTCATAATCCCCAAGATCT

>scaffold108.1.2360081

CACGTTTTTATACAATTAAAAACAAAGATTCAATCAGTGTATATGAAAAATATACATCACGTATATCGGTTAAAATACGCCCACAAACGTGGAGAAAACC**[G/A]**TGCCATTGAAATCTGTTTTTGTTTTATCCCGATATCTCAATCGGTTGCCGAGATATAAGGATTCGAAACGTTCCGTAACGCAGAGACAGCGAAGAGAGAT

>scaffold108.1.1194503

AATAACTTTAGTTATACTATATATATATTGTGAATAATTATAAATTTATTCATGATTTTTATAATATTAAAATAAAAATTGGAAGATTTTCCAGAATCTG**[T/C]**AATTCATTTTCTTACAAAACTGACATTATAAATTAAATCAACTCAAATAATTGTTTGATTATTATTTATCAATTATTTGTCAATTATAATTTGATTATTG

>scaffold109.1.1741658

GATGGATCGAATAATTATGTAGAGTTCATTTTGCGAAAAATTCATATTCAATGCTTTTTTAAAGTAGAAAATGATATTTCTAATTAAATTCAAATAATTT**[A/G]**CCATTTTTTTAATATATAAATCTATAGTCATATCGATGTTTAATTTTTAAAATAATAATACAGACAAATGTCTCTTACATTACAAAGAAATTAAACATAT

>scaffold109.1.2521415

TTATATTAAAAGTCCTTAATGCGGGTAAGTGGCCTTTTCCTTTCCTATGATATTACGAAAAAATTCATATTATTTTTTAAATTTATTCACTTTTAAATTC**[A/G]**CTAGGGATGGATAATAATATGCACTACTCGAAATTTTGTAAAAAATTAAAAAAGGAACACATACGTAAACGATTATTTTATCTTTTTATCTTGATTTATC

>scaffold109.1.1870603

TTTTTTTTTAAACAAATGATAAAACGAACGATAAAGAAGAATTATTTGGAAGATTGATTATAACCGTTTTCTTTTATTCCGTTCGTTGATATTTATTCGA**[C/T]**AATTAGAAAAATAAAAATTTTATCATACTTTGCATTCTCGAAAAGAGGAACTAAAATAGAATTTAAATTCTCTTCTTACACTTTAGATTTTTATTGAAAA

>scaffold109.1.1967732

AAAGGTAACATTTAATCAAATAAAATGTGTCAACGTGTATCATTCAGATCATCCATCCAATGCTCAAATATTCTATTTAAATAATATGAAACTCTGTTCA**[C/T]**GTACTTATCTTTAAATGAACATTTCTTATTATCTCATCAGCCAAACGTTCTTCATAGTATCTCAATAATCGAACTCAGAAACGAACAAGGAATACTGCAA

>scaffold109.1.2252460

GATCGTAGATTCCAACAGTATTATAGCAAATTGCAGCAAATTTTCCAATAATGCAGAGATTAGAAATGTAATTAATTATTTATGTAATTGAAAATTGATA**[C/G]**TATAATTTGTTATTCCTTAAAAATTTAATCATCTGTTAGAATTCGCCATCAATTGTGATTTCTTTTGTTATATATAGTTATAATGAATTTTTTTCAATTT

>scaffold109.1.2273830

TTTCTCGTCTGCAAATCGTAATGATTGCTTGTATTTCTAATAATTTTTGTACTATTTAAAATGTAAAAAGTGTAATAAAATTAATTCGAATATAAAATTA**[C/G]**TATATTTAAAGTCAATATAAGCTGTTTAAATATTTCATATTATTTTAACATCCTATTTTAATAAATATAATACGTTCTAGAATTATAGAAGAAGATATTT

>scaffold109.1.2283796

TTGTCTATAATTCAGATCATATAAGAATATTAAGAACAAAATGAAGTATTTGTAAAAATTCGTAAAATAGAATCATTCATATAAATAGAAATACTATGAA**[C/T]**GCATAAGTGTTAATAAAAAACTAATTACGAGAAACAATTTTAAATAAAAGTAATATGTTGTATTATTTTATGTTAATAATTATTTATATTATTTAATGAT

>scaffold109.1.2312621

TTGTTACATCCATATCCTATTCTTTCTTCCCATCGCATTTCAGTGACCCATTGTATCACGAAACTATGATTTTTTGAGTCTCATTAAATCCTATCCAATG**[C/A]**TTTTCATCGATTCTATTCGCATTATGTCACAATATAAGTCTAATATAAGTCTAATAGAGACTTGAGTTTCTGCGACGAAAATGAGATTCATCTTATAAAA

>scaffold109.1.2333444

TTATTTCTTTTTTTTTTCTTTTTTTCTAATTTCTTTCATTATTCGAGAGATATGTCATGGAACTCATATCGTCTCCATCGAAAAGCATCGTGTACATCGA**[C/T]**GTGACGAGGATTAGAGTTCCGTGTAGTAAGAGTGTGGTCCGCGAATTCGGCATGGAGGCCTGATTTCGCGAGCGCTGCGTATCGAACGCTCGTTCTTTTC

>scaffold109.1.2489078

AGGGTGGAAAATCGCTTTTTGAAATTGACCGGATGATTGGATGGGATGAAGATGAAGGGGACAATTGAAGTTGGAGATGCTCGGAGGAGAATCGTGATGG**[C/T]**AGTCCTTATTGATAGTCTGATTTCGTGGAAGTATCGTGGTGATATGTAGTTTTCTTTTTTTTTATGATTTTTGATAAATTTATTTATAAGAATAGAAATA

>scaffold109.1.2559494

ACATTATTATCCGCGACAAAGAACACTGAATCGATGGCGAACGATGCTTGGTTAAATTGCGCAATTTTACGAATCTGTGAATTATTTATATATTATTCGA**[C/T]**GAAGCGTCATTTCGTTTTCTTTTTCTAAAAGCTATTTTTAAGAGAAGAATTTAAATACCAAAAGCTTTTCAATATAGAAAATAAGAATCTTTGATAAAAG

>scaffold109.1.2849855

CAAGTGTATCGAATTTTCTTTGCGCGCAATTCTTCGAATTTGACGAAACTAATTTTTCACTTTAGAATATCAATTTAAAATTCATACTTTGTTAAGTCAA**[C/T]**AATATCGTTACTTTTTCTATCATATTATAAATCTATTAATTTATTAAACTTATATATTATATTGAATATCTTATTCTTTCACCGAAGTAATCGATGAACG

>scaffold109.1.2929443

ACAAATGAAGAAACAAATTCAAAATAAAGTAAATAAAATAAAAAAATTTTGTAAGAAATCTAAATAAATCATTAACCAAAGAAAAGAATACATCACTAAT**[C/G]**AAATGAGAGAATAAATAAATAATTTGATGCATACATAAATGAACAAGACTAAGAAATTTACAAACAAATCATTAACAAATGAATGAACACATATATAATT

>scaffold109.1.1567538

ATGAATTTTTGTTCTAAATTGATGATTTTTTAAATTTATGGATTTAAAAAATACTTCCAAGTATTAATCTGTTTTTTAAATTTATTGGATTTACTTATAT**[G/C]**AAATATTATTTGATGATTGAAATGAAATATTAATTTGAATTGGATAATATTATTATTGGACTGTGAATTTTTATGCAAAATTTTTATATTTTATGATTAA

>scaffold109.1.1626750

TCTTATCTTGTACAAGATTATATTATTTTATTACTTTAAAAATCTTTATAAATAAAAATGCAAATTTATCTTATTATTATTTTATCAACATTATATATCT**[G/A]**TCTATTTTCATTAATATATAAACAATTCTTATAGACAATTGTAATATTATTCAAATAAAAACAACTCGAACTATTATATTTCAATTCACAATGATCACAA

>scaffold109.1.1763268

GTTAAGTGGTCCATTGATATAAGCATATCTAGGAGCACTTTGCACTTCTTGAAACAGTTGAAACGTAAAAGTCATATATACGAATTGATACGTAAAATTC**[G/A]**TAACACGTTATATGAAAAATTTTATATTTCAAGCCTACAATTTTGCTATTTTTTAAATATATTATAATAAAAGAATCGATATCACAAGTTCAAACAACAT

>scaffold109.1.1800321

TTTTTGAATTAAATAATTATTATTTTAACTTAATTATAATGTATTTAATTATAATGTAATTTTAATTATTATAATAATATTTTTAATGTATTAAATTAAA**[G/C]**TATTGTTTATAAACTATATATAAATACTATAAACTTTTGTTTGCCTTGAAAGTAAATATTCTTTTTGCATTACTGTACATATTTCGAAATGAGTAATGCT

>scaffold109.1.1883187

CATAGTTCCACGCGTTTCACAAATATATTTTCTTTTCAAAAGCGAGGAAAAAATTAGCCACTTAAGTAAAAAGAATATTTCTTACATTTTGATATTTTTA[**G/C]**TTGATCGATATTTCATTGATTATTTATTATTAATTATTAACGAAGAAGAGAAAATTTATAAGAAAAAAAAAGAAACATCGAACGAAATCAAGTAAAAATT

>scaffold109.1.2091562

CTTTCCAATCGGGGAAGCCAGTTGAGGGCGAGATGCAGGTTGCTGAGGAGGATCCAGTTCCCGTTTCTGTTTGGTAAACAATGTTGATTCGTTTTACCTT**[G/C]**TTTTTTATTCGATAATGTGTGTTGTGAAAAATAATTTTTATTCCGTTTCTTTTTTTTGTGATCATTAAATTTTAAGAAATCTAATCTTAATATTCAAATA

>scaffold109.1.2296707

GTTTTACATTCAGTTTGGATTCTGAATTAAAATACTAATATCTCAAAATAGTAATTATTCTTACATTTTATATTTAAAATATAAGTTTCCATTCCTTATT**[G/C]**AAATTCGAAACTTAATTCTAAAATGATAATGTAATTACTAATTACTTTTATAGATTGATGTAATCTAAAAAAAAAGTCATTATAGTAAAACATTAACATA

>scaffold109.1.2460097

AAATATCATTAATATTTTATTGTAAAAGTATCTATTCCTAATTTTTAGTACAATAGAGTACAATATTTTTACAAATATTCAAAGCGTTTTATATTATATT**[G/C]**TAAAAGTAATCCGTTCAAGTATTCAAAGCAACCTTGTCATTCGTTTTTCACAGATGCTACAACGAATTTGGAAACCCGGAGGAATTGGTTTCGTCAAAAA

>scaffold109.1.2484549

ATTTATTTTTGCATTAAAATCACTATCAAACCAAATATACTATTGTGTCATGATTTTAAGAATCTTGTAAATATTTTTTATTTTCAATTTCATATTATTC**[G/A]**TTTAATTTTAATTTATGTATAAAGATGTAATATTATTTAGAATTAAATAATTTTTTATAAGTAAGATGTGCAATCTTACTTGTAAATATTTTTAAAACGT

>scaffold109.1.2525878

AAATATATTCGTGCAAAATGATATTTTCTTTGAAACAAATGAATATACTAACTTCGTTTTTTTTTAAAAAATTAAATGGTTTTCTTTTTAAGAGAAGTTT**[G/A]**CTTTTTAAATGAAAAGTGCTATTAGTTTTTCACACCTCTTATCGAATTGAATGAAAAATTTATTTAATAATATATCTGTTTAACATCTATCGTTTAAATA

>scaffold109.1.2549469

TTTCCTAGAATTCACTGTTTCGAGTAGCTTTTTACTCCATGATGGAAAATGATGAAAATTTTTTTCACCGAGACTTTTGACGTTATTTCTAATCCCTCTC**[G/A]**TTTCAATCGTCTAAATTTTAATTCTATTCGAACACTCGAAAGATTTCTATGTTTTCTTTGATGTGTAATGGTAAATTCTTTTTTCTTAATAATTCTCCTT

>scaffold109.1.1791010

CATAAAGACATGCAATACAAATTCAATTAAATATTAATTAAATGATCGATATTTTTATACAATGTTATATGATAATCAAATAGAATTTACTAAATTAAAA**[T/C]**GATGGTTAAAACGCAATATAAGAAAATACTTGAAAAAGATTGTTTTCGAATATTAAAATAAATACATACAATACTATGTGTTGGCATGATTTCCCATTTT

>scaffold109.1.1945865

AAAATAACATGAAATTATAATTCTATTATCTGGATTGGACCATTATTTGGACCTGTAATTCTCATATACAGAGTCTGACCGAATAATATACACAAATGGA**[T/C]**AAAAGTTTCGTTAAAAAATTCGTTAAAAAAATAGAAAAAGAAGGGAAAGAAATTAGAATTGGAAAAGTTTTTCGTTCAAAATTTCGAGAAAATTAAATTT

>scaffold109.1.2067975

GTAAATTTCAGAACAAAGTTAGAACAAGTTACATCGATGATACGATCGATTAACGATGATTAATTAAAAAAATTTTTTAAAAATTAAAAAGAAAATTGTA**[T/C]**GATATTTATGGAAAGTGGAAATTCAATAGATGTCGATGTTTGCATTTTAATGAATTTTGATACGAAAAATTTGAGAATAATAACGTGTTACGCATCAATG

>scaffold109.1.2304584

TGTTGTTGCGTGATTTTTTTTTGCATAAGAAAATTATCGATTATTTATTGATTTAAATGATCACATTAAAAAAATTAATTAAAATAGTTGAAATTTGCAA**[T/C]**GTATCGAAATCTTGAAGCTCAGAGAGAAAAGAAATATTTTGAATATCAACCTCATCAATTTCATATGTTTCTTTTTCATAAGAATTTCCTTTTTTCATTG

>scaffold109.1.2501986

GTGTACGAATTTTTCAAAAACCAATTTTTTTGTTCCAGAATAATGGAGATGTCTCGTTATATTGAATATATTCATGAGAGATTCTAGAGACTAAAATAAA**[T/C]**AGAAAAATTGATATACAACAACGATTAAAACTTATTTATTAAAGTCAAGCAATTGAAGTTTGCATTAGAGAAGTCGAGATGGAAATAAAACGAATCTTAT

>scaffold109.1.2920059

ATAAAAATATCTTTCCTCTATAAAAAACAAAAATCTCAATAATCCGATAAACATTTCCGTTAAATGATAATACATACATAAGTTTCATTTTAATTCACGC**[T/A]**GAATATAATATAATCCTAATAATATATCCAATCCAATAATTAGTTTCTGCTCTGTGAATATTTAAATCGTCGATTGTATCAACGAAACTCAGAAGTGGAT

>scaffold109.1.2925765

TCAAAAATTGAGCGAACGAGTATTCAGAAAATAATATTTTAGCGGAAAAATATTAACAGAATTAGTTGTAAATGTAAATAAAAAATTTATTTAACTCAAA**[T/C]**GTTATCACAAAATACGATTAAAAAAAAGAATAAATAAAAAAAGTTTTTAACGATAAATATTATATTAATATATTATCACGTTGCGATATTTAAAATAATT

>scaffold112.1.1090517

AAAAATGATCCTTCTCTCTTGCGCGTTGGACGAATTTTAATTTCGAGAGGTGTCGAAAAGTGTCGAATAAATAAACAACGAAGTGACGAAAAATTAAATC**[A/G]**TTAATTTTTCCTCGAGTTATTTTATTATTATTTGTTATATATAATTATTTTATATTGAATTATTAACTTTAAACTTTAAATTGATATATACTAAATATCC

>scaffold112.1.637228

GAAATTAAATGTATTTTTCTTTTTTTTGAAAAGAATATTAATCACGTATCCGGATAATAAAATTTAAAAAATGGTGAAAAATACAATCGATCAGTTTGAC**[A/G]**TTTTTGAAAAATTCATTTTGCGTACAACAATTTTTGTTTCTCGTCGTCAATATTACGAAAAATCACAGCGGAGAAAAATAATTATTCAATGAAGAGTCGA

>scaffold112.1.690356

GAAGGTCTGAAGCAAAGAAAGAGACAATTAGATTGATATTAAAGAAAAATTAAAAGGACAAAGTTTTTCTTTGGTTGTTAATTTTATTAATAATGGATTT**[A/G]**TTCTCCGAAATAATTTCTCAAATTATTCCAATTCTAATGGACTGAAAATAAATAATGTAACGTTTGTAATAATAATGATATCATTGATGGAATTTACAAA

>scaffold112.1.1047516

ACTTTATTCGTTTCTTCGCAAAAATTACCTTTGAAGCTTATTTATTAAGATACATATGAAATGGGCTATTTTTATTTTTGAATTTCATTTCATCAAGAAT**[C/G]**ATGGATAAAATTTTTGAAAAATCATATCAGGGAATTGCGAAATAAAAGAAATTGAAATTTTGATGTAACTAACTTGAAATTGAGTGCAATAGAGTGGTGT

>scaffold112.1.1150763

TATTGTTATTATAATTTAGTTAATTTTAATTATCATAAAGATATTTCTAATTATAATAATGTTATTAGATATTTATTATACTATCATTATAAGTGCTTAA**[C/T]**GATTCGTGACACATAGAAGCATCTATAAGTAGATGACAACCATAATGCTTATTTATATTAATTAATAAGAATGAATTATTTTATTCGTAATGGAATATTA

>scaffold112.1.619598

CTTTTACTTATTCGTATATTATTCAAAGTATTGTTTAACGAGGCCATTCAACGATCATTTTCAACACCATTCCATGTTATTTAATTATTCCAGTTACGTT**[C/G]**AAGATACGTATTTTTCGTTCGAAACTTATATCGTTCCATCCCGAGAATATTAAAGAATTATGAATAGCTAATATAGACTTAATAATCAAGATTAATATTA

>scaffold112.1.668791

TCGAACGAAGGGAAACGAGAAAGAAGGGAAGAAAAGAGCGAAGAAGGGCAAACAGTGGAGGGAAAAAGAGCCCAATTCCTCTCACGTAACCATTCACCGG**[C/T]**GGAACGAAAACAAGTGGAAACGGAGAGGAAGGTGAAAATTCGCACGTTTCGTTTCATTGTTAGGGCGAGGAGAGAGGTTAACGATTCGTAGGGAAATTTC

>scaffold112.1.825480

TTGGTCTATAGAGAAATATTCTTTATTTGACATAATGTTATTCGTTGACATTATTGAATTATCATTATGTTGTTATTATTATTATTATTTTTTTTTATTA**[C/T]**GAAGAGTCTGGTTTTTACAAAGCGTCAATACCGTCAGCCCCATTTGGCCTTGTTTTCCTTCTTGCCCCGGCACCCCTTGTTCTCCTTTCGGTCCCTGGAT

>scaffold112.1.939711

TAAAGAGCTTTGTAAATATATAATGCGTGTTTTACATTATATCCTTTAATTGATTTTATAATTTTTAAAAAATTTATTTCAGTATTTAAGTTTTGTATTA**[C/G]**TACGTAAAAAAGTTGGAGAGTTTACATATCAAATGTCCATCGATAAAATTCGAAACGATAGTTTTTTGAAAAAAAAAAAGATAGGGAAAAAGTTGTTTTA

>scaffold112.1.1086594

ATAAATAAATTTTTCATCTGGCAATGAATACAATATTTATAATAAAAGAAATCTTTAAAAATTATTCACGATTGCATTGAAATTGTATATTAAAATGTGC**[G/A]**TCGTCATTCAAAAGTTTTAACAATTTTTTCTAATGTTTAAATGCTATTTGAAATCGTAGCAATAAATTGTATATGTCTTGAAAATATAAAATTATTATAA

>scaffold112.1.1088702

CTAATATCAATCTAACACATATTATTAAAATTTCTATTTTCTTAAAAAATTACCCTCATTAAATTGATAAAAATTTCAATTAATAATATTTTTATTTTAC**[G/A]**TACTTTCAATATCCAAACAAAAATTATTAAAATTTCATTTACAAGATCTCAATATTCTGATATAGAAAACTTACATGATGAACTTGTTCACTACTCTTAT

>scaffold112.1.1122167

ATATTGAAAGTAAAAATACATTTTTATATCTACGCGATTCGTCATTTTAATGCTTGCTTTAATTTAAATTTATCCATTTATCATAATTCATAGAAAACTC**[G/A]**TAACTCTATCTTATTTTTCTCAGTGACTATCACATGTATATCATGTATATCACGCCTATGTATAAATGATGATATATACTCGCTTCGTCATCATGATTCG

>scaffold112.1.227473

ATTTAAGCTAGCCGACTATTATATGGCAAACATGCCACTTGTCTAGTCACTTCATCTTCGCCATCCCGTGGCGCCTTTGATTATTTATTTCATATCTTGC**[G/A]**TCAAATATAATTAAATAAAAATTGGGTAAAATTATAAAAATAATTGCAGAGTGTTTTTTTCATATTTTTTACTTTACGATCGTTTACTATCACTGTATTA

>scaffold112.1.289745

CCGAAAATGATTGATTAGATATGTTGCTACTTAAGAAATAGTTTGACATGTCATCATTTTTATGAGTTAATATATTTTCTTTAAATATTGAAGTTATATT**[G/A]**TTTATACCTTTGGAGATTAAATTATTAGATGAAACATTCATAGTGGAGTTAATAGTAGTTGACATCATAGTAGAATTTAAATTTTTAAAAACATTTGTAT

>scaffold112.1.834995

GTCACTCGAAATTCTTTATTGATCTATCTCTTTCAATAATTTCAATTTTTATATTATTTTTCTTTCTAAAATAATAATTTACAATTATAAAAAGATTCTC**[G/A]**CCACTAAAATATTCTATTTTATCAATAAGAATAATTTTTTGAACTAGTTCATTAATTATTCCTTTTCTATCAATCTCAAATTATCGTTAATCTCAATCTT

>scaffold112.1.1096421

CCCATCATTTTCACACTTTACCCGCCTCAGGAAACTATATCACCCCTATTCCAAACATCATCTCGCACGACCGACAGAACCCTCAAATAAAACAATTACG**[T/C]**ATCCCTGCCTAAACTACCCTGAAAACTCAGAACAAACAACAATATACGAGGAATACTAGTCTCACGAAAATTTCTAATTATTGAATGAGTTTCAATATGT

>scaffold112.1.235552

AGAAGATTGCTTGCTTGTTTCTTCCTTTGATGTAATCTCTTTCGAGAAGTTTCGTTAATATCCAGTTTTAAATCTGTATAAGGAAACATTCGAGTTTGAA**[T/C]**GTTGAAGTATAATCCATTACGATTTGCTTCTTTGCAATAAGATTGAATAGATGAAATAATTCATCCGGCTTCAAAGCTTCGTTTAAACTTCGTTTTTATG

>scaffold112.1.256714

CAATTATTAATTCAAAACGTACATCATCATAATAATTCGTAAATGAATTCTCGAATCGCTCGACATATTAAAAAATTTAAAAAATCTAACCCTCCTATAA**[T/C]**AAAAAATACGATTTATCTGAATGGCATTCCACGTGTATTGCATTCCTGAAACCATATGCAACTTCGTTTCGCATTGAATGCCTTTATTGAAATAATTGTA

>scaffold112.1.316456

AAATATAATATCAAAAATAAGTTGAAATTTTTTTTTTCGTGATAGATTTTTTAATTTTAACGTCAATTTAATTACATTACATTTGTTTGTTTGATCAAAA**[T/C]**GCCGGTGTTTCTAAATCCGTAGAAGAACCTAAGGCAAATTGTGCATCTTTCATACGAATATAGAAAACCAAAGATAGATTGTTCCCCTTTTCTAAAGACG

>scaffold112.1.527195

CCATCGTTCTCGAACAGGAGTTGGATTAATTAATCGAGCTCAATTGTTTAATAAACGTTGCACAAATTATCGGGATTATGAATCTCGTGTCTGGATTTGG**[T/C]**ATCGTTTAAATTATTGCACGCAATATAAATATAATGATTCACCATTCTCGTAAACAGAACATAATTACATATATATATATATATCACGATAAGTAAATAT

>scaffold114.1.120428

GTAAGAAATGGGACGGGGAAAGTTATTGCGAAGAGAAAAGTTGGTCGACTGACTTCCCAGGCAGGAACATCAATCTGGATTTACTATAGTAGATACTGCT**[A/C]**GGTTTTTATATTGGAAAGGAATGTAGATCTCTGAGATGTGAAGTTTTTGTATTATCATAAGGAATAATGACTATATCTATTAGAAAGTTTCTAATTCTTG

>scaffold114.1.23677

TCTGTTTAATGAATATTAGAGATATTATTTAATCAGATTCAATAGTCATGGAATTAAAAGTTAATCTAGAGAAATCGATTTCAATTAAATTATTTTTAAC**[A/G]**TTGCTTTTAATATTTATAACAAGTCCTCTTGTGAATTCAATGGAAAATTAACATAAATGCATTATTTCCAGAATTATTTAACAAATTCGGAAAACGGATA

>scaffold114.1.53440

CCGTACACAAATTTTCGCCATAATATCGTGTCGAAGGTAAAATTCGTTATCATTTTTCTTATATTTTATTTTTATAATTCGTTATAATTTGTTTAATTTC**[A/G]**TACGACGCACGATTGCGTTTAAATATTTCATGGCGAGTAGCAGTTATTTATTTAATTGCCGTCATCAAATTTAATGAGTTAAAATTTTACTGTATATATG

>scaffold114.1.189280

AAAAAGAATAAAAAAAAAGAAAAAACTTTCCACGTGCTTGGTTGCGTTACGAACTTCGCCTGCAACGACTTTTGTAAATATTTAAATTTATAAGGAATAA**[C/T]**AATTTAACGGAGGGAAGGTAGTAAAACTCGATAGTTTGAGTAATTTTATTGCGCGGATAGATTGATCAATGGTTTATTTCGTTTAATTTGTTATCTCACC

>scaffold114.1.248128

TTAGGAAACACTTCTTTAAGTAATTTTGAACTCGATTTCTGAATATATCTTTTTTTCAGTTCTGATGAATCCTTTTACGAAAGAGTTAATATTCACAATG**[C/T]**AAAAAATTACATTGGTATTTCATTAATATTTCTGCATACTATTAGTAAAGAACAATATAATTCAATAATAAAATACAATGTTTTGAATTATAAAATATAT

>scaffold114.1.253134

AGAAATAAATTAGTTAAATGTAGATATTCATTTATACTTAAAATTCTTAAAACCAGTCTATAAACTAATTTAAAACATGAATAACACTTCAACTATAAGA**[C/T]**GAGAAATTATTTGATAATCCCACTCGAAAATTTTATAAAAGTAACTGTGTAAACTTCTACAACTTGTACGAAGGGATACTTAAGATAAGTGGTGAGAAAA

>scaffold114.1.253423

CATCAACCTTTGATAATAAAATATAATTTTAGAAATTTTCAATAACTCGAAATAAAATACGTATATACAATATTGAGCATCAAGTTTTAAAATATAATCT**[C/G]**AATCTCGATTGTTTGACTACAAAATCGATTAGAAATTTTGTACATTGTATATCTCAGAAATATCTCATAAATTTCTTTCAAACAAAAACAATACATTAAA

>scaffold114.1.147985

AATTTTTATCAATTTTATTGAGAAATTTATTCGCGAAGACGAAGCAATTGGAGGAAATAAATTTAGATAAATCACGTTTGTTTTATTTATTAATGGAAAA**[G/C]**AAATGAGCCGTTTCCTTGGAACGAGGATGCTGCGTATAACATTCCAAATCTCCGAGTGATGATTCTTTTTTCTCGTTGGTGAAACACGTTTGGCGACTTC

>scaffold114.1.88199

TATTTTTCTAGTCATTATAACATAACGATAATAATAAAAAAATAGCACGAACAATAGAAAGTAGTAGAAAACTATTCGTCAAACAAGCATTTTCACTTTT**[G/A]**CCCAGAGTAATTTTCCAAGAAAAGGAAGAGACAACTCTAAAAATTCAAGAAGAATCTCCATCTAACCCTATTTTAACCCCTTTAAACCCTTCTCCCTTCG

>scaffold114.1.39193

ATCACGATAGAATAACTGCTGTTCCTTCTAGTTTTTAAATAAAAATTAAAAATCACTATCTCTTAATGACGAATGTGGAATTAATTCGTGTTAGAAAGAA**[T/C]**GAAAAATCTCTCGATGTATAAAATATTTTTAATGTTACTTTTTATTTTATTCGTTTTAATTTAATTTTATCAGTATATGTAAAGTGAATGATTATAGTAC

>scaffold115.1.44215

ATCGAATGTCAATTACTTCGAATATCTCATTGTATCGAACTCAGAAGCGGAAATTTAAATTCATCGTTGTTTTATCTCCATTTAAAAGGGAAATGAAATT**[A/G]**CGTAATTCCGATTAAAGATAATTATTAAAAACAAATTTTACAGAAACGTATTTCTTTTCAAATATTAATCAATTTATAAATATATTACGAAGTATCACTA

>scaffold115.1.163893

TTATACCAATTTCATACGAATCTCTAGACTTTTTTCTTTTTCTTCTTTCTCTCTTCTCCGGAAGTGGGCCATGTTAAAACACTTAAATGTATCCATTCAA**[C/T]**GCATTAACTGTACAAAACCGTGTCTTAGCCGAACAATCGTCCATCCGCTTACGTAAATCTCACCTCTCGACTTCCTGTCCTTCGTCACGGGAAGGGGAAA

>scaffold115.1.59107

GTACCTGACATTCATGTTACTGTTAAATACATATAAAATTAAAAATCATTTCGTACCATTGATTCGCAATACCGTATTAATTTTTTAATACAAGAAGGAA**[C/T]**AAACTGCACGTGAAATTTCACTATAACTGTGAAATATAACGTGTTATAAATAATAAAAAAACCCAATCCTTTCATCCAAACAAACGTCTCAACTAAAACA

>scaffold115.1.15503

TTTGTAGCGTCGATATCACGTCTTCTTTCTTAATCGACGTCAGCTCGCATATCTCACTTATCGTGATTTGTGGTTTTTCATTTTCTACTAATGGCTTTAC**[G/A]**TTCAATAGAATATCCAATATAGTATGAGCCCAATAACTTCTATAAGATAATAATCCCAAATCCGACAATGGTTTTTCAGGTGAACCTGTTTTACCTTCGA

>scaffold115.1.34549

GTCGATTTTTATCGTAGAAGAAATCTAATTATTTTTCATCGATAAAATTTATGCGTTGAGGGATTAAGTAATGGGATCTTTAATGAAGATGTAGAGAAAA**[G/C]**TTGATTATTTGCGTAGCTTTGTACAGGGAATTTAAATAACTTCTAACGTACACAGCTTTTTACATCAAAGACCGCTGCTCCTCTAGTTTCTTTCATTTTT

>scaffold115.1.104860

AAATCTGTTTCGTGTTCTATTTTAATAAAATATAGAATACATTATGTATAATAAAGAAATTTATTATACATCCATAAATTGAAAAACAGAATACAGAAAA**[T/C]**GGAATAAACAAATCGTAGCAACTTCGAAAATAAAATAAAATTAAAAAAAAAAAAAAAAAAGAAAAATTCGCCATTCCGTTCGAAAAATCCTAATAAATTA

>scaffold115.1.191570

ATACTCGTTATATCTTTATCATATAAAATACCAAATATATCTATAATCTAAATGTATCTAAATATCAAATATTTATCGATACATGCCTCAATTTTTTAAA**[T/C]**AAATGTAATAAATCTCTCTAAACGTACAAATATACTACCCATGAATTATTTTTTTTTTTAAGATAAATAATAATTAAATTCTTCAAAAATATAATTCTAA

>scaffold115.1.201250

TGATAAGTGATTTGTTAATTGTGAGTGAGGATGATGTAAAGAGAAAGTAGATTGGATATTTTGAATTATAATTTTCGGAAATTTTTGATAATCGTGATTG**[T/C]**AAAATTAAACTTTCGTTTTTAAAATACTAGTTTGTATAATTTAAATAAATCTTTCCTATGTCATCCTCATGTGTAAATAATTATGTAATTCATACAATAG

>scaffold117.1.311644

TCGAAGCGAGCAGTTTAAAATCTCTAACCTAAAAAAATAATAAGAAAGAAAATTAATTTAACCCAACTTTTATGAAATCTCCAAGATAAATAAAACGGAT**[A/G]**CCCTTATTACCGATTCTCGGTAAAAGAGTGTTTTAACCTTTTATGTTGCCCGGATATGTCTCTTACTTCCGCTCGTGTGTCGTTACAATAATAAGAAGGT

>scaffold117.1.45483

ACGATTGCTTCGACAATTTCCTACAAAGTAAATTGCACCTCGATGAACGCTCAAAGTATTTAATCCATTCGCCTTGATAATATGTCCGTGTTGTTATTTT**[A/G]**CCGACGGAGAAAATTCGAAGCTTTTCTTTTTTTTCTCTCTCGTAAGTACAAACAAATCCGTGATACGATGGAATTAGGGGGTTGAAATTCGAGATAGGAC

>scaffold117.1.362322

ACTTAAAAGTGCAAAAATTTTTAGTAGAACACGAATAATTAATTAATTTTCAATATAGAATTTTCAAAATGGTGGGAGGTATTAGTCTCTGTCCTTCTCG**[C/T]**ACAGGTTCTCTCTTTGTCTATGACGTCACGAATACCTCCTTGCATATTCTGCTTTGCTAGTAACAAATCGAAGCTTCCAGAGTCCAAACGCGAGATGGCG

>scaffold117.1.217471

CAGTGTCCACTTAAGATACTCTGGCACGAGTTTTATTCTCAAACGGATTAATTTGAATCGATATTTTACCGTCTATATATTATCCTTTCCATCATCAGAT**[G/A]**CAGTGGCAAGGCTATTATTGGTAATAAAGTATTGAGAATGATTTTATAATTATCATATAAGTTTATGTAAGTAGCAAGTGCAATGAAACTAGTTTTTTTG

>scaffold117.1.740928

AAAAAAGAAAAGAAAGACAAAATTTCGTTCTATTATAATGAACAAAATGTCAATATTTGTGACTGTTATTCCAACGATATTATAAAAAATGATTAATTCC**[G/A]**TTTGCAAAAGTTGTTTGGAATATCCTATCATTAATTGTGCAATTGTTACGATCGTTACGATGATTAAAATATTCCATATTCTTACACATGTATCTGTTAT

>scaffold117.1.816577

GTTCGGCCTTTTGTCGTAGTTGGAACGAGAGAGAGAGAAAACGGATTGAATTTATCAACGAAATAAACAGGTATCTGTTTATTAACGACACGTATACGAA**[G/C]**TTGGGAAAATCTGTGGAAAGTGAATCGAAATTCTTCTTCTTCTTCTCCTTCCATTCTCCTTTTTCCCATTCGAATTCGAAATAAATAATAACGCCGTTTC

>scaffold119.1.236829

AACCAATTAATGCTTTACATTTTTCTTGTAACATCGTGAAGAAGTACACTTAAACGAAAATCGTTTTGGACGAATAGATATTCTTTTAAACTGTCCATTT**[A/G]**TCGTGTAATTTAATTATCGGTAATAAAATTAAAACTTTGTCATTACCACATATTGTTAAAGCATAATTGTTAGTTTTATAAGCGTTAAACTATTTATGCA

>scaffold119.1.239700

TTTATACATGAATCTTTTCTACTTTTATACATAATAATATAATTAATTAACATTTATTTATTTTTTTATGTGGAAATTACAGCACCAAATTAGATTCTTC**[A/G]**TTTTCATAATATTTAAATAATGATCATCCTTCTAATTCTTTCATTAATTCAAATTTACAATATGTACAAAAATTACTTGGTTTTCTCAATTTCAAAGAGC

>scaffold119.1.228408

TCATTAGAACGTAACATTTGTTGTGAATCGGTGGAACAGCGATGATAGTGATCGTTGGATTAATATTGGGTCTTGCCTGGATCTGTGAGATGTCTTTTAA**[C/T]**GTTGGAGGACGACCAAGGAGTAATATTTTTGGGGATGAGTAGAGGATTTTGTTGCAAGAAGGGGGTAAACTAATTTTTTGAAATAATTGGAGGAAACAAA

>scaffold119.1.17482

TCTGTTGATCCATTTTCTATAATCTATGAGTATAATGATTTCTAATTTTAGCAAGTTTCTACAATTTTCTTTATTATCGTTTTATTTTGTACAACAGATT**[G/C]**ACAAATTTAATTATTAATAACGTTCTTTAATAATCATTATTATATTATATTATATTAATTTTAAGGATAATTTTAAATTTCTCAAGATCTATTATCTCTC

>scaffold119.1.60335

GGTTTTTTAAACACAATCGATTTTTATTTTATAAAAATTAAATTCTAATACGATGATATTTCGCGATATCTTATCTTAAAGAAGATTGAAAACAGAAGTC**[G/A]**TCATTACGAGTGCAACAAATTGTATATGGGAATTAGGATCCAAGAATAATTGCTTATAAATTTGTTTTATATATTTTACTTAAAGAGGATTACAAATGGA

>scaffold120.1.15417

AAAAAGCCGATGAATTTTATAAATCGCAACATTAGATTATGGAGTATAATTCTTCTCGATATTATTAAAAAAAAAACAGAGGCAATTCCAGATAATAAAA**[C/T]**AACAAGAAAACCTGTCTCCAAAATCGATTCTCTAATCTTTCTTTCTCCAAAATAGAACTTAAATTAATAGCTTTCAATTTTTTTTCTAGCCAGGAAAAAG

>scaffold120.1.78022

AGTTCAACCGTGCTCGAGATCTAAGATTGATTGGGAAGGAGGAAAAATATTCTTGGGCTAATTTCTAGAGCTAATTTGAATGAAATATGATGTCTAATCT**[G/C]**AACGTAGACACGCAACGTAGAACTTCATTATACGAAATATTCTTGAATAATTCTTGAACTATTTCTAACATAAATTTGAGAGAAGCGGATAGCTGTTTAG

>scaffold120.1.112822

GTCAATTTTTCATCATATTGTAATAACATAGAGACGAGATAAATTTAATTCGTAAATATACATTTTTTCTTCATGTTGCAAAGATAATCGTCTCTAAAAA**[T/C]**GATGATATTACTACTAACACGAATGAATATTATTTTTGCAAGAAAAATTTTTCATCTCGCGTATATTCCAATGAGGAAACCGTATCGAAAAGTTGGGCAT

>scaffold121.1.176000

ATTTATTGACTGTAGATCTTATCGTGATACACGGCTAATTTTAATAATGAAAAAATTATTAACCACACGCATACTACGTCATATATATGATCCTATCTAA**[C/T]**AATTTTCCATATTTTTTTTCAAAACTTTTTATGTTGACGAATTTTCATCAGAATGATAATTTAATTCATGACATATATTATAGAATAAAGAAATAAATTT

>scaffold121.1.470504

GACAATTACGAAGATATAAACGATTGCAACACTCGCGAAGGTAAGAAACGGTACAGCGATAACTAAAAACTTATTATTATCTTTGCCGAATAATTAAAAG**[C/T]**ATAAATTTCCACGAGAATTTGAAAAAGAAAAAAAAATTTTTCAAGAAAGTATTCTTCAAAACCTAAAGAAATATTCGATATTTCGCTGTGTGAGTTTTAT

>scaffold121.1.155744

ATACAAATTGCGACTCTTTAACTACAAACGATAATTTTTATAATAATTAATATCATTTATAATATCGTATCCCTATAAGTTTGACATTTTACTCTTGTTC**[G/A]**TTAATTTGAAACCGATATTCCTTCCCTTTTTTCCCAAAATCAAAGATGGTTATCGAATTACAGAAAAAAAGGTTACAAAATCACGTAGTAGTTTATTACG

>scaffold121.1.159251

AAAATTTAACTTTCTCTCTTTCGACAAATTTTTTTTTTTCATTTCTTTTATCTTTCGGTTCGATTAAATTTCATCAACAATGGACAGAAATGTTCCTATC**[G/A]**TTTTTTACGAATTTATCCTTTTATTTACTTCCGATACACCATTTTGTCGCGTGTAATTCTTCGTGATACAATTATCTTATACTTTTGTTAAAAAAGAAAA

>scaffold121.1.220947

TAAATTAATTGAAGCTGAAATCAAATTAATTTAATTAATATTCATTCGTTCAATTTGAATCGAGTTTCGTAATTAAATTAATTAATATTCGATTCACATT**[G/C]**AATTTGAATGAATTAATTCAAATATATTTCCATATATTTTATAACAAACTTGAAACGTTTGTCAAGTGACTCTAAAAACGATAATGCATTTTATTACGCA

>scaffold121.1.273007

CTGTTATCTTCCAATTTATATGTTGTTGTATGTGAAAAATCAATTTTATAAACAAGAACTTTTTAAGTTTTTTCATTTTTTTTAAATACCAAATATATTT**[G/A]**TTAGTTCAAAAAAAGAATGGATTCTCATAAGATACGTTAAAAAAATTAATGATGAATAAATTTAATAAAAAGTGAGAAATAAATTCACGATATTTGTATT

>scaffold121.1.474029

AACACCTCGTGATGTTTAATAAAATTGGATCGTTACGATTAATCTTATTAATTTATAAATATAATTTATCTTATGTTTGAATGGAAATTCGTGACTGTTC**[G/A]**TCGCGGACGATACTTTTTATTTAAAATTGATATATCATTTTCGATCTTTTCACGATCAAAGTGAATGAGTGAGAAAGGAAGCTTTCGAAAAATGATTTAT

>scaffold121.1.509636

TTTCGTTTTTTTGTTTGTCGGGGGGATTTTTCTCTTTATGCAAACGATTTTCGCATCGAAAATATTTTTCTATGGAATCAGATCGTACATCTGTACATGG**[T/C]**AAAAATTAATCTAATTATTTGTATATTTTTTTTCAGAAGACCAATAAGGCGTTGAATAATAAGAGTGTATGGATATTAAAATTATAGAGATAAAGTATTA

>scaffold122.1.15996

AAATAAATTAATTAGTAGAGTTAATTGATGAAAAAATGGGAAAAATTTCTGGAAATAAAAAACGAAAGAGAGATCCATAAAATATATTTATAAAAACAAA**[C/T]**GTTCTTCGTACCAAAACAGCGAGAAATAACGATCCTGCACCAATTCCCTTCGTAATTCCTGCATTTTCGCCATGCTATGTATTCTCTTTTTCCATTTGTA

>scaffold122.1.23171

ATTATTATTTGATAAAAGATTCTCTTGATTATCAGAAATTGGAGCAGTGTTACACGGTATACAATCATAATATTCAAACAACAAGCAATGTTCACTTCTG**[C/T]**ACGTACAATTGGGAATTCAAAACTCATTTAGGCAAATGAGAAGAACACGAATCAAGTAAAGAAAAATAAAGAAAAATTTGGAAATTTCGTTCGAAGCTCA

>scaffold122.1.42286

AAATATTCTGTCTTCTGTTTGCACTATTTATTTATCATTTGTCGAATTCGAATTTGCCGCCATTTCGTTCACAGTAATTCGAAACGTTCTCAAATTCGTA**[C/T]**GGAACAAGTAATCGAGGATCGAGAATACGATATCGAATGTTTATAGTTGTATGTTTAAAGTATCGAGGATGAAGGAAGAAATGATTCTGAAAATAAAAGT

>scaffold123.1.217767

TTACAACTTTCTCTTTGGGATTGTCTGTCGAAGAACGAATGAATTATGCTTGGTTTATATTATCTGTTCAAGACACGAAACGATATGAGAATGATTCCGA**[C/T]**AAATATCGGACGATACATCATATCAAAACACTTTAAACTTTAATTCATTTGTGTATTATTGATTAAATGGTATTGATATGATAAATATTATCAATAACAA

>scaffold123.1.288985

AATCTTTAAAAATTGAATTCAACTTTTTAAAAAAGATTTTAATAAAATTAGAACGTACACAAATTTTAATTAAAGTTTAATTTAATTAAATAATAATTAA**[G/C]**AAAATTAGAATTTTCGAAGCAGATAAATTTTCTTGATATCATGCATGAACAAATCAAGTTCAAATTCAACGAAGCGCAACAGAAATTCAAAAAATACGAA

>scaffold124.1.16650

TATCCTCTCTTCCAGAAGTGTTTCTTCCACGATCCAAAATTACTTGATAAGAGCATCGTACCTGAAAAAAGAAAAAAATATAAGTTAGTTAAATTTATTT**[A/G]**TTTTAAATATTTTAATAATTCTTGTTTTAATTCAATCAAATTATATAATATTTTCAAAACTTTCCAATATTCCTTCATAATTTCTTCCTTTTATATTTAT

>scaffold124.1.263086

CCGTCATGATCGAGGATGTCGAGGAAATAGTGACCGAGGAATACATAACGCAGGAAGTGGAGCGCAGGCTGAAATCAAGAATTCAAGACTACGCTTCCGG**[A/T]**CGATTGAGCTTCACTTATTATCCCAAGTACAACGAGGCAAGTGAAAGAGAAAATTTTAAGAGATTTAAACAGAAAAATTTCTTTCTTTTCTTTTTTTTTA

>scaffold124.1.615521

AAAGCATCAATGATCATAATGATCAATTTTTTAACCATAGGTTGATATAATGCATCTCTCTTTATTCTAAAAGATGCATTAGAAGCAATTAAAAAATATG**[A/C]**TTTTTCTATGAATCTATATTTTTTATATATTTTTTAATTTAATTATGAAACTTACCTCACATTTTCTATAGACTTTGGTATATTATCTTGTGTGGCTTTA

>scaffold124.1.612440

GAATAATAAATAAATAAAAAATAGGCCAAAACCGGAGATGAATAAGTATTAATTAACAATAATGAACCATTAATAAATGGAATATACGATTGTACACCAA**[C/T]**GTAACCTGCAGCTACATCAATAGTTGCTAAACTATTGGAATTTCCCTGTAGAAAATTACTTTTTCATTTCAAATTTGTTTTAATTACAGCAATTATTTGA

>scaffold124.1.629070

CAGAAATCATCGAAATGCTCAAATTAAAAGATTTCCAAAAAGAAATAATTAACAATCATGAATTACGAAACAGTGAGTATAATTTTTTGTTTCTTTTTTT**[C/G]**ATGAAAAACAAGAATGATCTTTGGAAAAGTAGATATGTATAAAAGATAAAAAGTGGTTCAGAGAGGCGCACAGCTGAAATTTTTAATATTTACGCACGGC

>scaffold124.1.30173

TTCATTATTTCTAATTCAAAGTTCTCAATCAGATCGATAATCATATCGTTTCCTTTTTGTTAAATATTGAATATTAAAACTTTCAATTCGTACGTTTTCC**[G/A]**TCGTTTTTCCATCCCCTTCTCTCTTTCTCTTCAATTCTTTCTTATATTTCGATGCCAAATTATTAGATGAAATTACCTTATTAGAAAATTATATAACGAA

>scaffold124.1.390507

TGTTACAATTTATTAGGAAAGTTAGGTCGATTTGTTGAAATTATATTTTTGTATGTATCGTCGAAAATGTGATTGTTAATGAATAAATTTATTCCACGTC**[G/A]**CTATTATATTTTATATAATTTATATGAAAGTTATATCGATTCGTTATTAAAATTATATTTTTCATTCATCGATCCAATCGTCGATACTTCAAATTTGTTG

>scaffold124.1.548860

TTGTTCGAATAAAAGCATAGTGACTCGGCAATTCTCGATTCCTAATAAAAAAACAACAAATTTTTAATTTCAAAAAAAAAAATAAATAAATAATTCGATC**[G/T]**CAAAACCTTTCCTTTTCATCGAAAATTATATCCTTCATCGAAGAGAAGAGATCCCCCTGTCGAGCACAATAAAAGATGAAATCATGGAATCGAAATAATT

>scaffold124.1.731309

TTTTTTCTCATTTTTCTCTTTTTATTTTTTAAAATAGAAATATGCTTATATTATATGTAGTGGAAAATAATTAATTATATTTAATTACATATCAATTTTT**[G/A]**CAATTAATACTTTCTCATTTACACAGTCACTTATCATTAATTTTAAAATAGATTAGTATTGAGAAATATTTTCACCATTAATAATAAGCATTAGTTATGA

>scaffold124.1.949793

TGATCGATTGGTTTCAGGAACGAAAATTTTTGGAAATATGTTATTCGACAACTCTTCGTGCGAGGCTTAAGATTAGAGATGAGATTCTAAGAATCACTCC**[G/A]**TTCTGCGTAAAAAGGGAAGATATCAACATCAATTGTAAGAATTAGAAGTTTTGGAGTGAAACATTAACCTTTGGATAAAATTTTCTTGGAGAAATTTCAA

>scaffold125.1.263327

TGGTCTTAGATTTATTGAAGTGTTTGCAAGCTCTGGTATCGGGAAACATTGGAAACGTGCATCATTATTTGGATTAATTGCACCATTTGGAATTTGCTCC**[A/T]**CCTTTGAAAGAAGAAACGTTCGTTTTCAAATTGGAAGATATGACGTTATTTAAAAATCAATTGCTATTATTAAATCGTGACGGGGTGGCTTTTTATGCAG

>scaffold125.1.588648

TTGTCAAATACAATGATTGTATTAATTAAAATTATAATTATAAAGAAAAATTTCAAGCATTTGTTTAATATTTTTTCCCATCGTCGTTTCTTATTCCATC**[A/G]**TAAGTATTCCACAAAGTGTTAAAAATTTGAATTATTTACATCTATGATAAGACTAAGTTTTATTATCTCGTGAAAATGCTTTTCGTGTTTGTAATTTATT

>scaffold125.1.618437

TGGTGAATTTTTCCGAGGAAAAATTTAACATTTTTAACATTTCACTTTATTAAGGAAATTAAATCTGATAAATTTTCAAAGCGTAGATCAAAATTGTATC**[A/G]**TCTATTTTTGAGAAGATCAAAAATTGAAGATACACTTTTCAAATTCAATTTTCTCAAAAATTAATCACACACATATAGAATTCTTATAATCCATTCGTAT

>scaffold125.1.210107

AAGGAGGATGGATACTTTTTGGAAATTTTTTAGCAGAGGAATTTATAAATATGATTTTGTTTCTATTATTCTAAGGAAGCAAAGCATCATTTGTGCATTT**[C/G]**TTCTTCGAATCAATTTGAAAATATTTTGAAATTTGTACAATTTGTAAGTTTATTCGAGTGATGCATTTATGTATTTATAAAAATTTATCCCAATGATTTA

>scaffold125.1.299197

GCGCCAATTTGCCAAACTTTCTGATTAAATGAATGGACGAAGTTATATACGCATATAAGTAAGCTTTTCCAATTATTAGTTCAATATTTTTAAACGGCAA**[C/T]**GAATCTTCGCGCCAAGATTTTCTCAACTTTCTCATTCTTCTATCTAACCCATCTGCTGAGCTTTGAAGAAATTTTGAAGGATCAATTGTCATTTTTCGTT

>scaffold125.1.553927

GTTGGCAAGTGTATTTAGTTTTTAAATATATTGGAGTAGAGATAGCGTTGAATTTTTCAACGACGCATATAAAGATATATTTTTAAGTACAAATTTGTAA**[C/G]**TTTAAAAGATATGAATATTGCTAGATATCAACTGGTGTATTAAAATATATATGGTTCCATTTAAAGAAACAATATTCACGTTTACAAAAGTTTTATCTAT

>scaffold125.1.649795

TGCTATATATACACGAAAACTTCCATTATCTAACTAATAGGGATGACGAGATAATGGAAGAAAAATTATTTTTAAATAAATTGTTCTTATACGAGGAAAG**[C/T]**AAGTAATATTTATTAAGAATTGTTTGCAAGTATGCTACATTGGGACAATGAAAAAGGATCCCATATTTTTTCAGCTGACAAAATTTCACGAATTTTTCAA

>scaffold125.1.142218

TTCAGGATACAGGATACAATAAAATTATCGTTTACTTTATAAAATATAACGTAGAAACATGTATTTTTATGTAGCACAGACAATTTAAAAATTATTTGTT**[G/A]**CATATAAAAACAGAAACTTCACAAAAAGTGGATATATAATAATTTATACAACAAACCATCGACCTGTTTCACTAATTGTTCAAACTTGCATACTCTTTTT

>scaffold125.1.20320

TTATAAAGTATGAAAAAAGAATAGGAAAACTGAATATTAACAATATATTCAGGAACTAAATATATTATCGAAATTTTTATTTATCATGAAGTATTTTATC**[G/A]**CTAAAATTGAATTTAATCACAAAAGATGAACATTTCTATGTTAAGCATAACTAAATTCTGAATGAGCATATCAAGTGAAGTTTTTAAAATTCCAATACTT

>scaffold125.1.299953

ATTCGAAAGGAGCAACGCGATCTCTCGGTCTTCCAGATCGAGTGACAGGTAAATCAAAACAATGCAGCAAGAAACAAAAGTGAGAAGCAAAGAGGAAAGT**[G/A]**CAGGGTATTCCAGAAACACGGGTCGATCGAGTAGGGGGTGATGGAGGGTGACAAAAGAAACGTTTTTATCTATTCGATTCCAATCTATCTTTCCCCTTTC

>scaffold125.1.350253

TAGAAAATTATTCTTCCCTGTATAATTCAACAAAAAAATACATAACGATATGTATACATCATGATATTCAGCATCGTGTCTGATCGTGCTATTTTACATT**[G/A]**TTAATTGCTAATAAATTATCATTATACGTTACATAAAATAATTTTCACTTTGATATCGGTATTTGAAAATTAAGCATGGCTATTCTCTTTCATAAGTTAG

>scaffold125.1.433571

CACGTGTGGTTTTAAATTCTATTGCAAGAATAATAGAGCGCCATAATTCTATTTCTAATAATATCCAAAATACGAGATCTATCTTGATTTAAGCATAACC**[G/A]**TATTGATAAAAAACAAATTACTTATGATAAAATTGATGATTATCAAAACATATTAATTTTTTTTAAAATGAATATTCAATAATTTATTTTATGGTGATGA

>scaffold125.1.516801

TATTCCGCTTTATTAACTGCCAATTTTTCTCATTTACATCGTTCACTTAATATCGGGGAGTTTCTCAAATTCAAATTTAAATACATCCTTAAATGATTAC**[G/A]**TTGGCAGATAATCGTCGAGTTAAAATTAGCAAGTGCGTAATAATTGATTAATATTAATCACCGAAACCATTGTTATTTAATAATATCCACGCCAATATGT

>scaffold125.1.655038

GTTAAAATTGACGTGTTCATCTGGACGGAAATTTTTAATTTTTCTTTTCTTTTTTTTTTTTTATTTTACATCGATCATTTCTTCAGAAATCGTTGATCTT**[G/C]**ATTTGATCATTTTCAGTGACCAAAATCATACGATATATTTGCGTTATTAAGTAAAAATGTGTATGAAAAATTGAAAAGATGAAAGGAATTAGTACTTGCT

>scaffold125.1.243520

ATCCTAATGAGTTATGAGAGATTAACAGTGGGAATAAGTGTGTGTATATTGTGCAAAAAAAATTGCAAATTTGTGAAAATATGTAAAAAGACAAATATTA**[T/C]**GATGACTATGTGAAAAAGATGAATGATGATATAATCTTAAATAAAATATTTGGTTATAAATATTTTAAATACTGAATAATGAATTATGAATACAAAATTA

>scaffold127.1.381211

AAAATAATGTGTATAACAGATGAAAAGTTTATAGAAATCTCATAAACTGCAAGTTGATATATTGAAAAAGAAAGAAATTTACACTCGACGTGTATCGAAC**[A/G]**TAGTAACGATTCGTGTCGGACGAAAAAACGACTTGTTCTACTGCAGCCACAGATACCAATTCACTCTCGTTTCACTTAAAATTATCTAGCTACGAATCCT

>scaffold127.1.389106

AAAATTATAATTAAAGTTGTCAATAAAGATTTTTGCTACGTATTTACGAACATTGGAAATTTCAATTATTCATATTATTTACGTCCAACGCACTTGTCTC**[A/G]**TTATTGATCAACAATAAACTAATTAAGGTAGTTTATTGCAAACCATATTTTGTTATATATACAGAATATCTCCAGATCTTTTTTTCTTTTTTTATATCCT

>scaffold127.1.1011170

TGGAAATTATTTGAAGAAAGATACCGTTCTTCGATCTTGATCATTTTTATTCGATATAGTTTGTCAAGATTATATAAGTCTCTAATATATGAAGATTTTA**[C/G]**TAACTAGTTATTCATTGTAAGAAATTATTGAGACAAGTTTTTTTTTAAGAGGTTAGGTTAGATTTAAAGTTTGGTTCAACTATCTCAAACACAATACAAA

>scaffold127.1.539062

TTGTACATATAGAATAAACATAAATATAATATTGTAAGAATTATATTTACAATAGATTATGACTAATAAATGCAAATTCATAACATGCTTTTTTGCCTAA**[C/T]**GAAATAAACAAAAAAAGAAAATAGATTAAATTTTTTAATATTATTGCAAACAAAGTTTTTTTTTCAAAAATTCAAAGAATTATATTTAAAATGGAGTACG

>scaffold127.1.672494

CTTTGTTAAGAATGTAGTGAGTGACAAGTAGCGAAAAGTAAAGAAGGATTCAAATATTTGAAGAAATTTATGATATATGATTTTAATATGAAATTATTCG**[C/T]**ATTAATGGATCCAAGAGAATATCTTTACCAATAATAGAAATGATATTTAAATATATCCGCGAGAAGAAAATATAACAGAATATATAACAGAAAAATATTG

>scaffold127.1.1005988

CTACGAAATGTTAAAATATTAATATGTTAAAATGTTATAAATGTTATTTCAAATAAATATATTCGAAATTTTAATTAATTAATTTTTCTCACACTACATC**[G/A]**TTCACTTATTCATCAGAATCCTCTCGAAATTAAACAGTTCAGGATCGTTTAATACTATAGCCGATAAAATGGCAGGAGATCAAGGCAATTAAAAATGCGC

>scaffold127.1.1175179

GAAACGTAGAATTCAATTTCTCGTAATCAAGTTCTCGCTTCTTCTAATATCAAAGAATATCCCAGAACTCGAATCGCATTGATTTCTAAAATTAACTTAA**[G/C]**ATTATGAATGGAGAATTTCTCGAAAAATTTGCTCGATTTTCTTCTTTTACGAATCATTAAAAATATCGTTATTGGAATATATACTTGATCGAGAATTTAT

>scaffold127.1.1692998

ATTATAAATTATATAAAATTTCGAAGGATGGAATAATTTTTCTTATTATCTCTCACGGATCCGTTTATTATTATTATTACGACCAGAAGAAGAGAAGTTC**[G/A]**TTGGAAAAGTTTCTCGCAATCGGAACTGTTCGAAAAATTTTCGCCGTCTATTTACGACGCACATAATTATCGTCAAATTCGCATCGCGTAATTACGAGAT

>scaffold127.1.195908

TAATTTTAAAAATTATAATATATTACTGATTAAATATTCAAAAAAAAATATACAAAGAAAAAAATATATCATCATCATTATTATTATATTAATATGATAA**[G/C]**TATATACAATGTATATACAATGTACAAAATTGCTAATATTGATGGAAAATAACAATCACTTGAACTTTATACAAAGAATATTAAATTTGTGAAATACGGA

>scaffold127.1.236175

TCTAACTACCTATTCAATTTTATTGGATAGCACATTCTTAAAATACATGTTTGTGATTATAACTCATGATTTAGCAAATATGCACTCTTGTATCAACTTT**[G/A]**TTTTAATCACATGTTTTTCAATAATTCCAATTTTCAAATACAATGTTTTTTTGATAACTAAATATCAATTTAATTTTTAAATATGTTCTTTTTCTATGTG

>scaffold127.1.32180

ATTCTTTTTTTCGTTCATACGTTCGGTAATTTTTTGAAATTGAATTATTCGAAAATTTTTGGATATTGTGCGAAGAATATTCTACTTTATTACGCGATAT**[G/A]**CTTATTATTACAGTAGATACACGAGAATAATGATACTTGGAAATAATACTCATTAAGCTGCTATTGATAACGAGTAGATTAGTTAGGACTTTTAAAGAAC

>scaffold127.1.852313

GGCTACTGATGACCCACGATAGAAAAATTAAAATTTACTTTAAATATTATTTTATTTTATATTATTATTCTTCTACTGTTCTATTATTTTTAATTTTTTC**[G/A]**TGGCATGATGACGTATTACATCAAATTCAAAAATTTACATGTGTCATAAAATTGATATACTCGTTCAATATGTTCGTTTCGATTTAATCAACGAATCTTT

>scaffold127.1.890541

TAAACTTATATATATATTTTTTAATTTTTTATGCTATATTTATATCGAAATCAAAATAAATATCTTTATTTTAAACTTCAAGTTAAAGTATTTTTATATT**[G/A]**TTGTTAGTTTATATTGTTGAAAGTACACAAAAAATCGTCAATTTTAATATTCTTTGTATATAAATTTCACCGAAAAATAAGTTTTATTATTAGAATAAAA

>scaffold127.1.979992

GAAATTTCAAGAAATTTTAAATATTTGGTAGATTTGAAATATTTGATAGATTAATAAAAATTATCATTTTGCAAATATTTTTAATTTATTGATTTATATC**[G/A]**TTCTTGATAATCCTTGGTCTTGAATAGCTTATAATGTAATTTACATTTTATTCGTTTTTTGTCATCCAATTGAAAATGAATGTTAATATTAATATAACCT

>scaffold127.1.986823

TCTAAACGACTAAAGCTGTGTGACTAGGATCCGCATGGAAGAAAATTCGATTTTATTTTACAGCATAAGCGAGGAATTCTGAAACAAAGTAGAAACGTTC**[G/A]**TTTACAAAAACAATTGGTTATGAATTGTTCAATATCAAATTGTTGCAAAAATTTATAAAAAAAATATTGATCTGCTATATATTTTTGTTATACAATATAA

>scaffold127.1.1005337

CGTGCACGTGGTCATTTTTCTCTTAATATAGCGTTCATGACTATTTTCATCCTTGTACAATATAAGTAGTATATAAAGATGAAGAAATAGTGGAGGATTG**[T/C]**AGAGATGAATTTCATTTTCATTAAAAATTATACACAAAAAATATAATACTCATAAATATATTCTATCTATATATGATGATCAATAAAATTCTGTTTCTTT

>scaffold127.1.1124765

TCTGATTGATATTGAAATAAATTGAATCGCATATCAGGGTAGAGCCTTGATTTATAATATAGAGATTATAGATTATGCGATACACGCCACGCTATTTTTA**[T/C]**GATACATTGATCGAAAGAGCTTACATATCGGCGTATCTATCAGTTGAATAAAATAATCTTACAATTTTTCTCGTTACCATTATTTGAATATTGTATGATT

>scaffold127.1.196795

TGTATATTTCGAAAATTAAAATGATCTTGAAAATCAGAATTAGTGAGATATCTAAATAATTATCAAATATAATTTATTAATAATAACATTTTGTCGTAAA**[T/C]**AGACAATTTCTACAGTTTTTTTTCCCCCATTGATTTCATACAAACTTGTGTTAAACTTGTGAAAATGGATATACGTCTATCAACGATATCATAAATATTT

>scaffold127.1.339490

TCAAAATAATTAATTGTTTTAAATATTTTTTCATTTGAACGATATTTAATCTATAAATTATTTTCAACTTTTTCAATTTAATTTCGATTTAAATATTTCA**[T/C]**GATATGATTTATTTTATAAAATTTTTTTACATTAAATCATTTAAATTGTTTATTTTTTAATCATTTTAAAAGGTTAATCGAGGATCGACAGATTGAGGGA

>scaffold127.1.660771

ATTTTACCTATTGCTGCCGGTACGGAATTCCAATTAAAATCGAATTCCGTTGGTGCGGTATTTTCTTCCAGAAGCTTTTGAAAATGACATGTATCTACGA**[T/C]**AAATTAATAAACATTAAATTATTAATTTTTATATTGTTCAATCTCTGATTAAACTTTACGATCATATGTCTATGTGTTTAAATATAAAATTTCGCATCTT

>scaffold128.1.317744

GATAAAGGAATAAGAAAAATCTGTCATTACGCGTAAATTTCGATACGTATTCTCATTACTTGTTTCTAAATCATTTTCTCAATTCAAGCTTTCATCTTCA**[C/T]**GTTATAAATAGTGCATTGAATGACAATGATTGTGTTTACTTTTAAAACATTATTACATTTTCGATAAAAACGAGCATGCAGAGTTAGAAATCGCAAAAAC

>scaffold128.1.521451

GTAAATATTTAATTTTATATTTGAATAGAGATAATAATTATACGTATATACAAAGACATACTATGAAAATAATTAATAGTTTCGAAAATATTTCAGCTAA**[C/T]**GTAACGTATTCAATCTTATTCTAACGTAACTTCATAGATGAAAATAATTTTTACGATTTTGAATCTATTATTATAGTGTGGTGCGATGTCAACAAAGATT

>scaffold128.1.665913

CACTCGCCTCTTTCTATCCTCTTCTCTCATACAGATCTCATCTCCGCTCTTTTCGAGCTCTCGATTAAGTAATACACTGTGCAATCAACTTACTGGGGAA**[C/T]**AGTGTAAATACACAGCACCCCATTGAGAATCTTCTTGAACGATCCTCTCACTCACTTGACCAACTTTGGCTAACATAAGGTTAGAAATAATATTTCCTAG

>scaffold128.1.680878

CATTCACAAGTGTACGATATTTAAGACCGTTAAAAAATATTTAAGCTATAATTTAGATAAAATAACTATAAAGAAAAAATCTTGAATAATTTAAATATAA**[C/T]**AACACAAAGTGATAATTTTAATTTTTCATACATTAACACGTTCACTTCGATTCAAATTTCCATTCCCTATCCAACAAATAACTTAACTTCGAAATAAACT

>scaffold128.1.760784

TTAATAAATCCTCTCCCTTTTCGCCGTTCAAATAAAATCGTATCAGAATTCGATACAAATATTTTTTTACAAATTCCTTCTTTTTTTTCAAATATTTGAA**[C/T]**AAAATAAATATATAACGATTTATTAAATTTTAATAAATCCTCCCGTCTTTCGAATAAAACACGATGACAATAGGATTGTTTTACCGATAAAATCGAATAT

>scaffold128.1.1139127

CTATTGAAATTTTTTCACTGCAAATATTTCGAAGGCTATAGCTTATTGAACTTTATTATACAGAAAGGAAACGTCTCTTCGAAGTCTCTTCGTAATTACC**[G/A]**TAATTAAAAATATTTAAAGAATTCAGATCTAATTTCCAGATCTATGATGAAAAGATCAAATGCAAAAATATCTGTTTTCCGCGACATCGAAACGGCTTCT

>scaffold128.1.353988

TTATTGTTTGGTTATTTAAGAAATTTTTTTCGTTAAAAAAAAAATTCGATCAAGATTTTTCTTGATCTTGTTTTCATTCTTCGATGAAATGCGTATTCCT**[G/A]**TTTAATTATTATCCGTCCCAACTGAAATGTTATGATAAAATGAATATTGGACATGTGTTCACGCGAATGGTATAATCTTGTAAAGGTAAGCCGATTTAAA

>scaffold128.1.474584

CAAAGTACAAGAATAATAGGACAAATTCTTTATTTTTTTTCTTTTAATATCGATTATTTTATTATCGACATATTTTTGTCTTATTTTTAATTAAAAAGTC**[G/A]**TGATTTGTTGTTAGAAATGCATAAAGGAAACGTGGAATCATTATCACTTGATGCGAAAGAAAGACGATACAAAAGATTATAAAATACAATATTCAATTAT

>scaffold128.1.701231

TCTTGTCCGAAATGTTTGAAATTCTGTAACTTGACTCTGGGTCAGGTCAAACGATAAAGTTATCGAATCGAATATTTCATATTTTTAGCGAAACATAAAA**[T/C]**GAAGCGACGATATATGGAAGAAAGATAAAAAAAAATACATATCGATACAAACGAAAGGAAATTAATCCAGAATCATAAATAAAAAGAGAGAAAATATTAC

>scaffold128.1.840846

ACAAGATTAAAACAATTTCTGAGAAATTAAATTTTAAAAAAATAATATGTGGGTTACAGGATTTTTATGATTTCTAAATTTATTTATTAAAAATGTGTAG**[T/C]**AGAGCCAATGATTGAACTTGGAACATTTTCATAACCTATTGGATTTATAATTAATTCCACTTTATGATTTTATAATTATAGAAATAAAGTATTAAAGCAC

>scaffold129.1.280389

CAAACATCGCGCTTTTTTCCCTTCCTCTGTACGTTCCGTTTTCCCCGATTTAATTTTAACCCACTGCTATAATAAACGGTGTATCCTCCCGGTTCTAAAT**[A/G]**CTACTATTACCGATTTTCCATTCGCGCTTTACTCATAAATTTACTCACAAACTCGTAAACGATTAATTTACCGATCGATCGAACATATAGTCGATTCGAT

>scaffold129.1.411767

GAAAACATGATAAAAAATCAAATATGTTTAATCAAATTTATTAAAAAAATGCTTATATAACGTGCTCGAAATAAACTTATATCAAAGTGCATTGTGAATT**[A/G]**CATAAATAGTATAGATGACTAATGTAGAATCATACTTATGAACAGAAGATGAATTGTTCGATAAAGATAAATTTTGCTGCGCATGACATTGACAAGAAAT

>scaffold129.1.364841

ACATGCATAATAAAAAATTGCTTTCTCAAGATAACATTGAAAACGAAGCAGATGTTGGGTACAAAAAATTAAAATTTAAACAAAAACCTATCGAATTCTG**[C/T]**AATATCAACGATCGAATTTCCACCGATGTACGTTTAAGACCCGTGAAAAGTAGTATGTTCGACAACGGTAATTTTATTCGAAATCAATTGTTGGATATAA

>scaffold129.1.234047

TATCTACAATAAGTATCTATTTTAATAAAATTTTGACTTTAACAAATTTAGACTGAATTGTAAACGTTATCAATTTTATTATAAGACATTTATAGTTGCA**[G/T]**ACGGTAAAAAGATCGTCAGATAGACTGGAAAAGAAAAAGCTCTTTTAAGAAGCGAATTTTTCTATTTTCGGCTTCCTCGATTCTTTTCAGACGAATCTTT

>scaffold129.1.433559

TTACAAAGAATTAGCTCTTCAATAAACTATCTGTTTTTTAAAAGAATTTTTAGCTATTAATATTTTTTGTAATTGAATAAAAATCACTGTTGAAATAATT**[G/A]**CAGAATGGATTTAAATGTAACAATAATAAAGATAGAATGATTTTTTTAAATTATTTAGGGTACGTCTGTGGACAGTAATCCGTCTAGCCCTGCCAGTGTT

>scaffold129.1.471565

TTCAACGATGTAATTCTTCAAAAAGAAGACGTTCGTGTATCCTTTTCAGATAAAAATATGTTGAGTGATATTTACGAAATTGATACGAAAGCTCGCCTCC[G/A]TAATCGGACACGCGTTTGTCTCTTTTCATCTTTTTCACGAAGGATTGGACGATCATTCACACACCGATTATGCAAGAATCGCGTAATGAGCGTGCGTGTT

>scaffold129.1.67704

CGACATTCGTCAAGTATTTTTCGCTCGTGCAACTGGAAATTTCCGATAATTTCTCTTTTATTCTTATCTTTATAATGGAGATTGGAAAAATAAACTTGGC**[T/A]**CGAGCTGTTGTATCGTATAGACCATTCTAATTCTTTGGAAATAGATAAAATCGGTCGATGTTTTCTCGCTGTTTATCTTTTCCAAGTTTATCCTTCCCTC

>scaffold133.1.203193

CGAATTCAAGAAACGCGCAAGAAGATCCCATTATTTGCATAAAATCGTATAAATCTACGCACCTTTACGTATTCCAATGACGTTTAATCTTCGAGTAATT**[A/G]**TTTCATCAAATTATTTCAGGGAATACGATGGCTTCATATATCGGAATATAAAATTAATTCCAATAAAAAAAGTTCACTTATTACATCTCACGATTCTTTA

>scaffold133.1.218299

ACCACAGCAAACGCCTGGATAGAGTTATTTTCGGAAATTATTTCCATCCCCCGGTCTCTAAAGATCCACCAAGAGATTTGCATAAATTTTCTGCGACCAC**[A/G]**TTTAAAAACTGGATAACCATTTCCAATACGAGTGTTTCCTTAGAATGTTGAAAAATAATTCTGATTAACGATTCGAATGCGATTTTCAGATTGGATAAAT

>scaffold133.1.60715

AAGCTTAAGATTTATTTTGATTTTATGAATTTGGAAAATATATGAATTGGGGAAATATAGAACTTTATATTCATTATTAATTGAAGATTGATTTTTCAAA**[C/T]**GAAACATTTGTGAATGAAATATTCCCTGAAATTCGCTTAAATTAAAAAAAACTCGTTTAAATTAATATTCATGTAGAATGATTCCTGTTTTGTTAATTAA

>scaffold133.1.1251

CTTTGTTCATTCTTATCTTCGTACGACTCTATGATTATAAAACTAATATAATTTTTATTTAGAATACTTCTTATTTAGAATACTTTACTTATACAGTTAT**[G/A]**CACGAAAAAGATTATTTTAAATAGAAACCTTTGTGAAATTACATTTTCGTAATATCGTAAAAATATCGTAAAATGAATATGATTAATAATTTTTTATTTT

>scaffold135.1.168562

ATCAATATATATCGGGGGATGAAAAAATCAAATCTCGCGAGATTTCGAAAAAAAAAACGCTCGATGATACACGTACAAATTTCGTAAAAATATATTCGAC**[A/G]**TAAAAATTATTCACAGATAAATTTTATTATTCAGCACTTTTTTTAGTCGGCGCTTTTATTTATTTTTTATAAAAATATCTGAACAGTTTATCGGTGAAAA

>scaffold135.1.170076

TCGTGTCAAGAATAAAATACGTTACGGTATAACCAACATCTCCAATTTTAATGGAATAAATTTTTAATCTTGTCCAGATACTCAGGAAAATTTGTGAATC**[A/G]**TTCTTGCAAAGGCTTTAAGATTAAATTCTCTTTTCCTCTTGTCTAACAAACGATTTTCATTGCGGGTCGTTTCCGGATGGAAGTGGAAACACAGGTATAA

>scaffold135.1.154527

AAATGTGTTTATCGATCGTAGAAATTCGTCCATCGTTTCTTTTTGTAATTTGTAACTTCAATTTTGTTCGATCTTAGTTTTTATTAAATTAAACTAAAAT**[G/C]**AGAATTTTAACATATACAAGTAGAAAATCTATAAATAAATTGATTGTTTAAATAATAAGATAATTTATTTTTTCAGATTGAACGAGGGATTGATGATTAA

>scaffold136.1.19375

ATAATTACATTCTTCGTATAAAGTAAATGTTAATAATTGATTCAAAAATATTAAATTTTTCTTAAAAAATCAATAATGGAAGTTATTATTACTTTGAACC**[A/G]**TTCAAATATGAAATAATCATTAAATAATGATTAATGTGCTTATATTAATATCCACGAATTTTTCTCAAATTTTGAGTACATTTATAAATACATAAATATA

>scaffold136.1.330572

AATTTAAAAAAAAAAGATTCAAGAATTTTATTTAGTATCACACCAGCGAAGTAACCATCTGTTCATATATCTAATTGGGGAAATAGCCAATTTTTCTTGT**[A/G]**CTTCAAATTTAATTTATACGAAAACGAAGAAATTTTTCTCTACTTTCGATTTATTTTTTCGTGTAAAATTCTATTCGGTACTTCATCACTAATACGAATA

>scaffold136.1.192327

TTTAATTTTATTTTATAAAATATATCGGTGAAATTTGGAGGAAAAAAAATTTGGGACAGCTTGTATTCGTTTATCGAATTTGTTAATAAATAAACTAAAA**[C/T]**GTTTTTCAACAATATTGATATTATAAAGCATTTTTCATAAATAATGTAACTTTTGCAATTACCATTTTACATATGTGACAATTTAATTCATTAACAATAA

>scaffold136.1.243633

TTTTTTTAATTTAAAATTTTACGAATTTTTATTCACCTAAAAAAAGGGAAAATTATATAACGAAACTTGAAGGATTGATTTCACCTACTTCACGAAATGA**[C/T]**ATTCCATTAATGAAAAAAAAAAATTGTTCCATAATTGATGATCCATAATTTCACAATGTCTCATATAGAACGGAATTATCGTTGATGAACAACGACAAGT

>scaffold136.1.243953

CTTTTTTTATAGCCATATGCACGAGTCCAATATCTAAAAAGGAATCAATTGTAATGACACGAAGCAAACGGAATAATGGTCGAAACAAACAAGCAGGGAA**[C/T]**AGTTAAAAAATTGTGGAAAGGGGGACGTGTAAATATCTCGCCACAAAGCTTTAAAATCATTAAAAAGATTTCATTGTTCGACGTCATATAAAGTTAAAGA

>scaffold136.1.203886

TTTTTAAAAAAGTTATTATTAGGAAGAAACTAACCTTAATGAAATTTCAAAAAATTTTATAATTGTTTACGTATTTGCATACGTATTTTTTACAGAAAAT**[G/A]**CCGGTTGCTCGTAAAATTTTCGATCGATTTCAATGAAATTATAATAATTAAGATAAATCATCAAGAATAATTAAAAAAATTATCACGTTTTAAAGGATGT

>scaffold136.1.317855

TTATATTATTTATACTTATTTTAAGATAACTTATATTCACTTTTTGCGCTGTTTGTCATCATTGCATAAAATATAGGATAATCATAAATATCTTCTTATC**[G/A]**TTATACAGGTACACTTGTTACAAACAAGAAAAATTCATATATATATGAAATAAAAAAAAATTAATCTAAAATCTAAAATAAATTTTATAGAAAATTTGTA

>scaffold136.1.43209

GTAAGTTGATTCGTGTCGATGCCAAGTATACCAAGAACAAATATGACACGTACACGTGTGTATATATATATAAAACAAATCCCAATGCAATCGAACAATT**[G/C]**AACAGAGACGAATTTAATTGAATTCAAAACATTGAATTTCGTACGAAGTTCGTTTCCATTTGTAAAATGATTGGAGGGAAGAAAAAAATCGTTGCTTCTT

>scaffold136.1.72246

TTCGTGTCGACTGACTATAAGGCTATAAGGGGAAAAAAATCATTTGAAATTTTATCCATCTAAAATAAATTAATTGGAAATACTTACCGATACTTGGTAT**[G/A]**CAACCATCATTGCGAGGATTAGGATGATACTTCAAAGTACACGCGCAATATCCTGTCACGCAGTAACTGTCTCTGATGTAGCAATTCCGATGCGTTTGAC

>scaffold139.1.250508

CATATTTTTTATACCATAAAATCTCTTAATATCTCTTAATAACTATTATCTTGATTTATATCTATGTAATAAAATTCCTATCTGTGTATACATAATAATT**[A/G]**TTTTTTTATATATATTTTATTTATTTTTATTTCTATGCAAAATAGAAAAACTTGAATATTTTTATTCTTCTACAATTATTTATATGAATTAAATTACGTT

>scaffold139.1.10694

ATACGTATTTTATAGAAAATGTACTAAAATCTTGAAGAAAATTAAAAAATAAAATTTAATAAAATATTAAACATTGTAAACATATGTACATAATGTTTTT**[C/G]**TATATTTTTTAAAAAGTTGAAAATTATCTACAATGTAAATAATTATCTTATATAAATTCTATAAATTATTATTTTATTATATATATATATTATATATTGT

>scaffold139.1.189008

CAAGTTTGTATATAAGCTTCTTTTAATATTTCAGGATTTTCTGTAATAGTTTTCTGAAAAAAAATTGTTTTGTTAAGATCTATTGAAATAAATTTGGTGA**[C/T]**AGAAAATAGAAATGAAATATGAAATATTAATTCGATAATTTGATAATTATTCGGCAATTATGTGATAATATTACATTCAAATGATATAAATTCTCATACG

>scaffold139.1.279962

AGATTGTTTTACAACAATTTCATTATTAAAATTGAATTGATATATGACAAGAAGTAAAACAGAATAATTCTTTAAATATTATTATTTTGATTTTCGTCAT**[C/A]**GTTATTATATGTAAGAAAAAATGTTTCATCATCGAATTGAATCGTTTTAGGTATATTCTGATATTGGTTTTGTTTTTAGATGGACGCATAGAGAATATGT

>scaffold139.1.30997

TTATTATAATAATTTTAACCTTCAAAAATAAATTATGACAATGAAAATGAGATGATTTTTTTTACAACAATGATGTGTTTTTTTTTCAAAACAATGATAA**[C/T]**GATAATAATGATAAACTTGGTATTTAATGATATTTACTTAAATTATCTTTACAATATATTGACTTGGAAAATAGAATATTGATATTATTTGGATAATAAT

>scaffold139.1.337829

TGCAAATTGCAAATTAAGTATAATATAATATATATCATTATCAGTAACAAATAATTATTATTACATATATACTTAACATTTTAATTGATTCTAAATAAAA**[C/G]**TTTCCTGAAGAAGAATCAATTTTTTAAAACAAAAATAAGTAAAATTTAGAAATTTTTGGCAATGAAATTTAGCAATTGAATATATTAGATTTTTTAGAAA

>scaffold139.1.362107

TCAAAAATATTTCAAGCTTATCAGGAAACAGCGTTTTCGTTTTACTTATATGTGTCATTATTAATTAGGATGTGTTTGATAAAAAATAAATGGCGATCAT**[G/A]**CGATCTTTTGCCAATATTAAAAAAATTATTTAAAATATCTGAAAAACGTAATTTCTATTTTATCGATATCTCTGCATTATTTATCTATAGATTCTTTGAT

>scaffold139.1.479645

ATATGATCATAAGGATTAAAATACAAAAATTGATAAAAATGTCTATTAAAAATTATTTTTTAAGAGATTAAATTTTCTATTTATTTTGTTTAAAAATTCC**[G/A]**TATTCTGTATATTACTGGATAATAATTGGCAATGTACCATGAACTTATTATTAATTTGAATAAATGTATAAATTAATATTAATAAAAATCTCGTTTTTCG

>scaffold139.1.519370

ACAAATCAAATTATTAATATATAAATATATAAATTATAAATATTAAATTGTTTATAATTTAAAAAATAAGTCGATATTTTTCATATAAATTTTTGCGTTC**[G/A]**TTTTAATTTATCTAAAAACGATTCTTCAAAAATGTTTATATTTTTATAAACATCTTGTAAAAATATACAAGGAAAAGAAAACGAATCAAAACGAATTAAA

>scaffold139.1.65298

GTTCTCAAAAAAATTTGTGCACATTTTATTCATTTTAAACGATAATATTTTTTATTGATTTTCCTTGATATTTCCGTCTCTCAATCATTAATTTTCTTCT**[G/A]**CTATTAATTTTTTTCAGAATGAAAGTATTGTTCATGCAGCATACGACATGCTTTGGTACAATTTAAATCCACGGGACAGTCGAATTGTATTATTAATTTT

>scaffold139.1.65871

ATAAAGAGTAATATTAAATAAATAAAGATATAATTTTAAAAAATCTATTAATTATTGAAACTTTAAAACTTTTACAATTTAAAATTATCGCTAAATATTC**[G/A]**TTTAATGAAACTTTTAAATATCACATTTTAATATTTCAATCCTATTTTAATATCTCAAGATAAATATCACATATTGTATTCGATGTATAATTAGACTGAA

>scaffold139.1.480923

ATTATATGATAAAAGAATAGAATTCTCACAGTCTACTGGAAATAGATTTGTGCCTCTTGTCTTATTTCCACCATTATCGTCATCATTTCAACCTACGTCA**[T/C]**GTTTGTCTTTTCGTTGTTTGTCAACCTACTCTCAATCTCAAAATATCAAATTGTTATAATCATATTACTAGAAGAATACTAGAAAAATAGAGGACACATA

>scaffold143.1.174700

TGGTAGTCAAAATAATATTCTGACACGAACTTTAAACAATTTATGCATAAAATGTTATAAATATCCGATTATCGAATTAATGAATGTAATGATCGATTTA**[C/G]**TATACATGAATTCAAAATAATTTTACTTAATAAATTATAATATCAAATAATATTGAATAAAATCACGTTACCTGAGATCATAAGATTACGTAAAAAAATG

>scaffold143.1.131908

GCAACATTCTTCAAGATCTTAAGTTCCATCAATCTTCGTGAACTCGAGGATACCGACGTTTCATATATTTCTTTCTAATCTTCTTTTTTGCTTCTATCCC**[G/A]**CCGAGAGAACACTCGATAAGAACAAGTCGAACAAGTTGGCTGACTTTATTCATGGATCAATGAATGTAGTGAATTTCACATAGTGTTCGCTGATGTGCAG

>scaffold143.1.187957

AAAAAACACTACACTAAATTAGAATGAATAGATGAAAACAATACTATTAGTTCAGCAATTAAAACTCAATTTTTCCTCAATATTTACCCTTTTCAATGTA**[G/C]**AACAATTATCAAGTTTCTATTACAAGTCTAATAACTCTTCAGAAACTCGAACAATAGCACACATTAATCGTCACTACTATTCTTTGTTACAATTGAATAC

>scaffold143.1.2353

AGAATAATTAATAAAAAAAAACATTTTTACGTTATTGTTATAGATATTACATGTTTTTGAAATATTAGAATTTAAAGAAATTCACAAAGGACTTTCTTAC**[G/A]**TTATTTTTTCAAAAATTACTTTACCTTTCGTTAGATTTGCAATTACACATTGTTTCCTAAGAAATTCTAGGCATAGAGTACGTGATAGGCCAAACGCGTG

>scaffold143.1.92412

CAATAGCATTTTAACGCGGATAATGCATCACCTCGACAAAAGAAAATTAAAGTGTGCGATTTTAATTTCCAAGATTTGAACGATTGAAAAACAATTATAC**[G/A]**TAATCTTCAATTCTTCCTCTTATTTTCCCTTATCTTCGATTTCAATTAATAATAATTTACGATCCCTCTTCCTCGATTGTATCACAATCCACTAAGGCAC

>scaffold143.1.133477

ACACGTACAGATTTAATTGACACAAGTATGGCGTGTGTTCAAGAATGTTCGAGTTGCGAAATTGAAATTCTTCGAATAATATTTCTAATATTAAGGGAAA**[T/C]**GCGTTAGGGAGCAATAGTCTCAATAAGAATATATTATGGAAAATTTCGATTAAACTTCATGTTTATAGAGATAAATGTAATTGAGTTCAATAGTATTGGT

>scaffold143.1.233982

ATTCTTAGCTCTTTTCTGCTTACTCAATTACATCAATCTTCTATGCCTCTCTATCTCTCTTTAATAGATATTTTTTTTCTTTTCCTAATTTAATATTCTA**[T/C]**GTAGATATGTACATATAATAACTTATATACACAAAATAAAAATATATTCTTGACTAAATTTATTCATATTTGTGTAATTTTTATCTTTCTTTTAGTTCTT

>scaffold145.1.127270

TTTTTTGTAGATAAGTCAAGATGATTCAGGACGTGTCAATTTTTCCAGCAGCTATAACTTTTTCCCACCAGGAAGCAGACTTTTTCAATATACGTGTCCT**[A/G]**TTAGAATCATAGTAGTCAACATATACAATGCCAAAACGTTCTCTAAAAATTAAAATATTATTAATTTTTGTAATTAATTTAATATTTTCTAACAGAACAT

>scaffold145.1.127399

TGCCAAAACGTTCTCTAAAAATTAAAATATTATTAATTTTTGTAATTAATTTAATATTTTCTAACAGAACATGCATTTACACGTTAATTTTATTATCAAA**[C/T]**AAAGTACCTTAAAATTAAAGAAATATATGACTACCTATCTCTGATTTAGTATCTTCATAAGAAAAAAAACTTTCAAAACTTTAAAAAAATTCTGACTGAA

>scaffold145.1.133741

AATGATTAATTTTTAACCGATGTATTTATCATATATCATAAATAATAAATTTAATAATATTAACAAATCATTAAAATTATTTTTAATGTTTTTGAATAAA**[C/G]**TATGCAAAAGTATTTTCTCAATATTTAACAATGATATTTTTAATTTATGTTAAAAACAAATTTAAGTTTGATAATATTTTGGAGGGCAGGGGGAATCTCT

>scaffold145.1.100846

GTGGTTTCGCAACCGATCGTTTCTTCTCCAACTTTCGACCAGAGGACGCCAGCTTCGAGCCGCTGTGATAAATCGAAGATTCATGAAATAGGTAAAAATA**[G/C]**AATCTCTGATTTTGAAATTTTCTCGAAATTCCATTCTCGCGATGTTTAGCGATGAAAAATCGTAGGGATATATTAAAAAGTCGAATCGTATTCAGGATGT

>scaffold145.1.186849

CGAATTTTATTTCTGTATTCTCGATCGAATCACACCGTTGATTTTCGTTGTCTGAATTGCGTTTTAAATGTTATTATAGCAACAGAAATGAAAAAATTTC**[G/A]**CTTGATGATAATCGATCGATCGTTGTTTATTTCTTTGATTTGGTAGATAGGTTATACGAAAGTTTTTCGCTCTTCTGACTTCTGGTCTTGTGTCTATATA

>scaffold145.1.376339

GAATATCCGTGGCTAAATCCGTGGAAAAAATTAAAAAACTCTACAAATTCTTCGGCTAATTTTTTTTCAAAATCCTTCTTAATCTTCTGAAAGATTATAC**[G/A]**TGGTCAAAAAGAAAAGAAAATCCAAGGATCCCTCGAATGAGTTTGAGTGGAGGATAATGCTCGATTCGCGGTCACGCTCATCCTTGGGACGTAGGATCGT

>scaffold145.1.504795

CTAAATTAATAAAATTTTCTTCAAACAAAATATTCATTATAAATTATTCATCGCTATATGCACTCTGTCTCTCTTTTCTTCACATAAAATTAAATTTCTC**[G/A]**TAACTCAAGCGACGTATTGCCAATCTTTTCAAAATCTACGAACAAATATATCAACATTCTATCTCTATTCATATATTATAAAAATTTTCAAATAGAACTT

>scaffold145.1.109611

ATAACAATTGAATTAATTATATTAATATGTATACTTACACATAATAATAAGTTTCGTGCCATATGTTTTATAACAATTACATTATAAATTAATGATATAA**[T/C]**GTAGAGTAGTATACAATTGATTTTATTTATAAAATAGTACAGATTTTTTTTTTGTTTTTTGCACTCGAGTCACACACTATTGATCACGAATCAAATACTC

>scaffold145.1.480762

CTTCGTTAGTTAGAAATTCGTGGATTATTTTTTTTGCCTCGTATAATATAAATGAGAAGAAATGCGTGTAATATTTGAAGTTCCAATAGAAATCTGGAAA**[T/C]**GATTCTTCTTTATTCTAAGAAATAATAAAAATTATATATCAATATTTCTTTGAATCCAAAGATTTCAATATAATTTCAATATAATATGATAATTTTCTTC

>scaffold147.1.163193

ATTGCAGAAATTCCCCGGATATCGATAAGTATGTTTTATAAAATAAATAATCTTCCGTCTCTTAGCGCGAATATGAATAAAATTTATTTCATTTTCTCTC**[A/G]**TTATTTTGTGAGTTATATCTCTTAACAATGAATGTAATGGGTTTCTTTTTTTTTTCTTTTTCGTTTAATTCTTCGTGATACAATATTAATATTATACGCT

>scaffold147.1.21920

GTAGATGGACTCATAAAAAACTTATTAATCTCAATCTTATTTTTTTAAATGGCACCATATACTTTTTAAGATATCAATCTGTGCATCCTGGCATTCTCTA**[C/G]**AAAAAACTATAGGTCCATTTATAGCAAAAAATCATTGGTTTAAAAGTTATTTAATTAAACTTATTTATCATCCAAATTATAAATTTTTTGCTATATAGGG

>scaffold147.1.405264

AATTTTATTGTATCGAAAATTCAATATCTCTTCTTTAAAAATTCTATTTTTAACCGAACTTGATACGTAAAATTTTGTGCAAAATTAAATACTGATCTAG**[C/T]**ATCTTTTGATTAAATCGTAAAACTAATGTTACTTTAAAATTAATGTTATTCTTTTCATTCAAATAGAAACAAGATAACATATATCTTGATATTTATATCT

>scaffold147.1.91181

GCGTTGATATCTTTAAGAGGATTATACGTATACTTGCGGATACACCCGCAGATTGAACAGAATTCTCACGTTTTCACGATTAATCTTACGTCTTCGTCAA**[C/T]**GTGGAGCTATAATTATATCGCGAACGAAAATAAAAAAAAAATTATTAAATTGAACATCCATGTTTTCTTGCTACGAATAAATAATTTATTTTGCAAAATA

>scaffold147.1.132081

TTTGGATCATGGATTTGAATGAGATTTTAATATCGAATTTACGAAGGAAAAAAATGGAAGATCTATTGGAGAATATCTGACACGAGGTTGATGATGAGTT**[G/A]**CAGACCATCGATATTTGATATTCAAGCTATTATCAGATCATGAAATTAATACATTTCGAAATATATATTATGAGTAAATGTCGATATTTTCGAAGAGAAA

>scaffold147.1.287710

ACCTTCGTGCCGATTGAATCCCCTTCCAAATTAAACCGATCGCTCGAGGAAGCGACAAAATAACCGATGACTATTTTTAATGAAACACTAATTGTTTCTT**[G/A]**TTATCGTAACTAAAAAAAAAATCATTACATATCCCCTCATAAAAATTAATCAAATTGTATCATGCAATTTCGATTAAATTGCTATGAATTTTATTTATTA

>scaffold147.1.325380

AAATTTCTTTTTAATTTATTCTTTTTCATTAAATTTCTCTTTCTTCAAGTTCTTTCTTTATTCCTCTTAAAAATTTTGTCTTCGTATAACGCGAATTATT**[G/C]**ATACTTTTGTATAAATAAATACTTACAAATTATAGCGTTGCTGTCGCGAACATTTTCAACACCCATCCTAATATTCTGCACACTGAAATCCATCTTAAGT

>scaffold147.1.407106

TTTTATGATAATTTTTAGTAAAAACACGACTGTCATAATTATTTTACATTAAAAAATTGAGATTTCCAAGTTTCAAATATTAACATATTAATTATTATTT**[G/A]**TCATATAGAATATTTTTTTTTAGAAAAATTTAATTTATAAATTAAGTAGATACTCGTTGGATAATGAGTCCAAATTTTCTCAGCTTTCATAACAACAATG

>scaffold147.1.64411

AAGTGATGAAATATTTTGAAAATTTGATACCATATTTTGTCGTATGATCGAATTTCTTGAATTGAACGAGTTTGAAGAAATTCAGTTTCGTTAATTTTTA**[G/C]**ATCTTCGAATTTCTTCTTTTTTATTTTATTGAAATTTAATTTCAATATTACGAATTTTAATAACCTTTGTAATTTTAAATTTCTAATATTTTATTATTAT

>scaffold147.1.282991

AAATTAAAAAAAAAATTCTATTTAAAATGTCCAATGATAGTTTATTTATTTGTTCCTTTTTAATTCTTAAAATATAAGTTGAATTTGCACTGTGTATTTA**[T/C]**GAATGCACACCGATTCACTTAGTTATTTTTTTATATTCTTTTCTTTTTCCTTTTTAAGTTGAGCAGATTTCTTTTTTTTAAATAAACTTTTTTCTCTTCT

>scaffold151.1.351740

ATCTTTCCAATTGAAACCTCGAATCTAACAATAAATATATATATCTTTATACAATTAATAATAAAATAATAAAAAGAAATTTTAATTAATAAACTAATCG**[A/C]**TTAATAAATGCAATAACTTCAATTAAATTTTACGTTCATCACGATATACTTTATCTTAAATACTTTCTAACAACCTACATTTACATTAAATTAAAATTAT

>scaffold151.1.358418

CTACATGCGTTTCACAATAATTTAGCCACCGTGGTTACTTATATAAGTTGCTATGAAATAACAACTAAATTCCTTGTACTTAACTAGTTGACATAAATGA**[C/T]**GGTTTAGATTTATGTCGATGCGTGTGTGAAATAATGATTATAACAGACATACAGTTATTATTTTTTATATTGCAAATACATCATATTGATTTGAAAATTG

>scaffold151.1.303753

GAGAGAGAGAAAGAGAGAAAGAGAACATGTTAGTCCGATGATATATCGTGAAAAACGAACGATAATTTTCTTCTTCTTCTTCTTCTTTTTTAATAAGAAC**[G/A]**TTGTTGTTTTAATTTCCTCCCATTTTTATTTCATTCCCAGTACCAGTAATGGAAAATGCTTGATAACGATATTTGTTATTTTTTTTTTGTACGACGAGAG

>scaffold156.1.30431

AACGTAATGGAAAACAGACATATGATATATTTTTTTATGATAAGTTCAAAAAGAATTTAAAAGAAACTTTATGTTCGTAGAAAACGCAATACGAAATTAT**[A/G]**CTTCTTTTAAGTGATTCTATTCACTTTGTTCGGTAAAATGACACAAACCTTCAATTATTATTCTTAGTGTTATCCTTATATTTTCAAATATCACATAAAT

>scaffold156.1.47889

CTGGAATGTCGTGGCCAATGTAAACCGCGCTACACGAAACGACAACATCGGTTGTCATTGATAACCAAAATATCGATTTAACTGAGTCATTTTCCGAACA**[C/T]**GGAACAGAACGGTTCTGTTCCTCTCTGCTTCTCGCAGAATATTGTATCGTTAATATCGTTTATTGCATTGTTTCGTATAATATAAATACAGTTATGAATT

>scaffold156.1.71290

TTTATTAAATGATAAGTAAATTAATTAATTTAGTTAATTTTATAATATTAACTCAAGTTAAATTTTTAACATGCTTAATTCGTCATAATTGTCAATGTTA**[C/T]**GTAAGAAAAATTATCAATTTTTTAAATATTCTCGATAATGGCAAACTTACAATTAAGTGAAATTAATAATATATATATGATGCTCAATTATTAACAATAA

>scaffold156.1.70017

TAACTGGATATTAATTTTATTGTTAGATTGTCTCAAAAGTTCTTTTTGCTTTTTTATTATACGGCTATCTATTTAAAACTTCTTGATATTTATGTATTTT**[G/C]**ATTACTTTATCCTTACATTTATCACTCTATTTTTTCAAAATGATTTTTATTTAGCTATCTAAAAAAATGGCTTTTAATGTTATTGAAATAATCAATGTTT

>scaffold159.1.21149

CGAATAATAACACTCTCGTCGCGTGGTGAATATATCAAATCAAGAAAGGAGAGAAAAGATAAAAAAATATTGCAATGAATAAAAACCAAAAATTAAACCA**[C/T]**GATAAAGAAATTATGTTAAACAGATATTAAAAATATTAACAATCTTATATTCAATCGTCCATCGAAGCTTGACCCAGTAAGAAAAAAAAAAATGTAACAA

>scaffold159.1.33870

AAGGAATTTTGGAATTTATTTATTTAGGAAGATTGGATAATTCTTTTCCTTTTCACGAGTGAGTGAAACGTAATGAAAAAGTTTTGTGGAGTATTTAAAT**[C/G]**TAACTGAATTATTGCATGAATTCTAATCTTGATAATAGTGTGTAAACTTTATTTATTTACGAAGTTTATAAAGTTCATATTACATTGTTGTAAAAATATC

>scaffold159.1.102133

CTGTTTACCATTTAAGTTTCGCTTTTAAGCCGTCAACTTTTCCTCCCCTTACTTCGTCTCATTCTGAAATTTCTTTCACAAAGACATTTCTGTCTTTCTC**[G/A]**TTCATTTTCTTAATTGACTCTCCACTTGTAACATTATTTATATATAATTTAAGTTTTAATATGAAAATTTTAAATTTTACTGTTTTAAAAATATATAAAT

>scaffold162.1.10635

TTATAAAATAACCTTAAATCAATGTAACTCTTAACTCTTATAAGTTTTATTCAATAGCCATAAGAATCTAAGTTCGACACCCAAATTTCTTTGTATCAAA**[C/G]**AAAATATAATGATACCTAGATGTTCCAAGCCATATTTTAAAAGATTCAAAATTGAAAGGGAAATATTTTGACGCAATAAACAAACTGTGGTTAGATTGCC

>scaffold162.1.368211

CCGTAATTCGATTAAATTTACAAATCGATAAATATTCGATAAATATCGAATCTATTAATGTTTCCTATTTTCGACGTATCTATAATTGTATATTACTTCA**[C/T]**GAAATTCGACTTCGAATTAATCAAATTCCAATCAAATCTTTCTATACCGATCGCTCGCCGAGCGATATTGGTTTTATTGGAACGATAAATTAAATATCGG

>scaffold162.1.390236

TTTATTAAATTGAATAGACTACCACGAATTTCCATAAATTTCATTCCAAAGAATATTAACTGTCGAATAAAAATCATTTCTATTACACGACGCATCTCAA**[C/G]**TTCTCAATTCAAGTGTCAAGCTGATTCAAAATTAATTCAAAGTGCCTCTTGGGAGGTAAAGGAGGGGGAAAAATCGCGAGGAACGATTTTCCACGAGGCA

>scaffold162.1.102918

GTCTATATTGTTTCAAAAAGATAACAATGATTATTCGTTTTCTATATTAATCCTTTACATTTATTTTTCATTTTCTCATCAAGGTACGTTAATTTTTCTC**[G/A]**TTTATATCATTATTATCTGTCATACAAAAGAGAGACTTGAACTTCTCTTTTAGATCATTTATCACTTAATAATAGTGAATTGAATAGAAATTATAGGTTA

>scaffold162.1.189921

ACACCGACTACAATATTTCCTGATATCCTGTATTTTATTTAAATAAACGTTAAATATTTAAATAAATGTTAGAAGCAGGATTTTTCTCTTAAAGATTATT**[G/A]**CTTCAATTGGATTATTCTAAATCGAAATTGTTAAGTTGAAACAACAAATTATTTAAATATAGAAAAAGCGATTTCTCCAACCACTGTTTCGCAATCCAAC

>scaffold162.1.58308

GGTACATTAATTGTTCCAATTATTGATCCAATCTTTTTATTTATAAAATTGCGCCGCAATTGTCAATAATTTAATAAGCGTTATTGTAATTAATAGTTTC**[G/A]**TTTGTGTATGTTTAAAGATAAAGGAAACGGAGGGAGAAAATTATCGACGATAAGTCAGAAGACAATAGTGAAGGATTGGGCACGGATCGGTACGTGCCCC

>scaffold162.1.137709

TTCGAAATAATAGCGATGGGGGGAGGATCGCAAATGCGAGATAGTTTATTACGATGATGGTGATAAATCATTGTAAGATCGATTGATCGCGATAACGAGG**[T/C]**AACGGCTCGAGTTTCGGGATAAATCGAATGTTGTGCAACGCGTTAAAGATCCGAATCCTATCCGTTCGATCAAATCTGCTTCTGATTGAATTAAAGTGAT

>scaffold166.1.1289122

CGGTGCAAATATTATAGATTCTCATCTTATAGATTTTACTGGATTTATGAGAAAGTATATAAATCTTTGGATTAAAAATATGTTTTAAATTATATTGCTT**[A/G]**TCATATCGTAATTAATAAAAAGATTGCGTTATTTGGATAAAAGTGTTTGTTTCTTGTAGAATTTTGTTTTCGGTCACCTTGTTATAAATACAAAGCACCG

>scaffold166.1.175477

TCAACAAATTCATTTCTGACACAGTTCGAACGATCCCACGGTCGAAATCCCTCTCGCGTTACCTTTCTCATCGAAAAAAAAAAGAAAAACCACTCGATCC**[A/G]**TCCTATATATCCTTACCTCCCCCTGAACGCTAATTTCCAATCTCAAACAATTTCCAATTTCCTCCTGAAATCGATCAGAAAACCAATCAACTGCTTCCTC

>scaffold166.1.198455

CTGGAACAGGCACGATTTTAAAATAATTATACTTGTATATTTCTTAATTAAAGGAATAAATAAATAAAAAATTAAATAGCGTGCGAGAGGATCAGAAATT**[A/G]**CGAGAAGGCGAGAAATCAAAAATCTCGAATTGAATCCCGAAAATTCATCCATGTGTGAATTGAATGGACGGTGATTCCCGTGCACGCGAAATTGTCGAAA

>scaffold166.1.88698

TTCGACATCGATTGGAAGCCTCTTTTATCCCATTTTTAAGAAGATAAATAAATAAACAAATAATTTCAATTCTCAATTCTCCACTCTCCTCTAACTATTC**[A/G]**TTTAAAAATCTTTCTTACATAAATTGTTTGTAAACAATTTTTCAAATGTTTAAAATTCATTTTTCAAATATTTTTAAATCGTCTCTTTAACAAAAGTATT

>scaffold166.1.96090

ATATATGCACGATTGAGAAAATTGAAATTATTATTCGATAATTGATAAGTCGTAACATAAAAATTGTAACAAAGGAAAAGATAATCATTCCACGATTCTC**[A/G]**TTATGCTTACGATTTGACATTTCTTTAAGTTTAGGTACATTTTCTCTCGAAATCGACAACTAATCATCATTGCATGATAAAAATAAAGATCAAAAGAATG

>scaffold166.1.1037006

TTATCGTAAAATATATGACGGCTGGACGAAAAGGAATCGAGAGAGTAAAAAGAATTTCGAAAAATTTATGATTGATATCACTCATTGTGCTCGAAGAATA**[C/G]**TTTCAAATACAAAAAAATATATATGTATATATATTAATATATGCAAAGAATACGTATTATGCAAAATTAATGTAGGATATAAAAATATAATTTTCTATTT

>scaffold166.1.1037735

TATAATATAATTAATTTAAGATAGAAATTAGAAAATCAAAAACAAAAAAACGAGAAAAAAGTTTTTTGCTTTTTTCAAGAAAATCGTGGTTTTTGCAAAA**[C/T]**GATTGGAATTTACGATTTTTTTTTCTCTCGTTAAAAGAATAACAAAGATAAAAGAATAACAACAATGGATTAAATTTTAAAGAATACGATATTAGAAATA

>scaffold166.1.1055042

GCTGGCAGTACCGTATTATAATGTCGCTTGCCTAGCGGAGCCCCTCAATGAAAGTAAATGTATAAAAAATATTTTTTTATATATGATTGATTATTTCAAA**[C/T]**GAATTTTAGTAAGCTAGTATGTATTTTAATTATGCAAATTGAAATCTGTGTAATTATTTATTTAAAAAAAACTTACCTCGGACAATGCAATTTCACAATA

>scaffold166.1.1102275

AAAATATAGATATTACATTCTCAATACGTTCTCTAGAAGAATGAATCTTGGGAAACAATATATCTTAATATAAGATGCGAATATTTCACTACAGCAAAAG**[C/T]**AATGAAATTTAAATTTAAATAAATGAAAACTCCTGATTAAACGAAAAAAATAAGAATTTCAAAATATTCGATCAAAAAATGACACTATTGAAAATTTGTT

>scaffold166.1.1162924

TAAATTGAAAAATAATTAATATAAATATTTTAAAATTTTATCACAAGCGATAGTTACGTTTATTCTGGATTAAAATTATTCGCACTAACGTTACGTTATG**[C/T]**AATATTATCGTCGATTTCCCAATGAATCGTAACTTAAATTCAAGCGAGGTAACTTTGATTTCTTATTTTTGACACATCTAGAATATTAAATGCATGAAAA

>scaffold166.1.1172470

CGATTCATGTGACGGAAAAATAGATATCTTCAGATAAATTTCTTCTGCGATATAATCTCGAATAAATTCATACTATTTTTAAATTTGAACGAAATGTAGA**[C/T]**GATTTTCAAATCATTCTTTTGTTCTTTCAAAGTATTGAATTTTAAAGATTTTTGTCTAATTAACGGTCTAATATTTGCGATTATATAAATATATCGAAAC

>scaffold166.1.1172534

AATTCATACTATTTTTAAATTTGAACGAAATGTAGACGATTTTCAAATCATTCTTTTGTTCTTTCAAAGTATTGAATTTTAAAGATTTTTGTCTAATTAA**[C/T]**GGTCTAATATTTGCGATTATATAAATATATCGAAACATAATGCGTTAAACAATGAAATGGTCGAAATATGAAGTAATCGATTAGCATCGAGTGGCGTTAA

>scaffold166.1.125917

CCTTTCGAAAAAAAATCTTTGCAAAATTGAGAAAGCTATTTCCCCTTTTCTCTTCTTCTATCAAACTCTTTTTATTAAAAGTTCGCGTGACTAATTATAT**[C/G]**AATTAATTTCAAATAACTGCACGCGAACGATAACGATTCTATTCCATTATTGAATAATTTAAGTTCCTTTAATTAGTTCATTAATATCTAATTTCTTCTT

>scaffold166.1.1271630

TCGGTCGCCAAGAAACAGAATATCAACACCTCTTACCCCTCCTCCAACGACAACCAAAGACGTCGAGACCATCACAGGTTAGTTTTCTTTGACCTCGTTG**[C/T]**ACATGGTATTGATATTACTGTGGATCATCCCATAATACTGTTTTAAAAGACCAATTTTAATCATTTGTTGTAAATTTATTATAAATTTATGATTTTTTTC

>scaffold166.1.143298

GGCCCACCCATGAAACATTCATGAACTTCAATGTAAAATTGTATTGCGCCTCACGATCGCTGTTAAGCATGTTATGCAAACGATTTCAACAAACTGTAGT**[C/A]**GCCACCAAGAATTTTAACCCAAACTTACTCCGATCGAGAACTCTCTCTAAACTCTCTCCATTTTCAAGATTGTCACGATCGGGAAAAATAAACTTTTTCC

>scaffold166.1.1514877

AACATCTTAATTCAACATACGTTACAGAATAGAATCCAAACAAAAATAAAGCGATTTCGAAATTTCCTGGCTAAATGTTTTTTTTTTACATTAAATGCAG**[C/T]**AAATAATATTCATTAATCGGTAGATATTTGTTAAATAATCTCATACATAAATACACATACATAGAAGATGGTATTTGTTCATGATGAAAATGTAAAACAA

>scaffold166.1.237956

ATTGTACTTTGTCTGTTTATTATTTTAAAGAATATTGAAAAAGGAAACACGATGTTTTGTAAACAATTTATATAATAAAGTGTTATAACGCATAGAGAAA**[C/T]**AGCAAGCAAAATTGCAGGTAAAAGTGTTGGATCGAGAAAGTTGTTATGATGTAATTTTGTAAAATTAATCGTGACAATCGTCGTTTCGACTAATTGGCTT

>scaffold166.1.325616

GTCTTTCCATGATGGCGCCACGATCTTTGACTGAATATGCTTTCAACGATAAGCGGATGGATCGACGAAAGATCGGATTATTTTCAATTTACGACGTTTT**[C/G]**ATTGAAATTAATGAGAATTTAATGAGAATATTTATAAAATTAAAGTTACATTGGATTCCTTTGAATTTAATATTTTTAAAATTATATTTTATTTAATAAT

>scaffold166.1.404902

GGGCTGGTGAAAGTTCTTTTTTACCGTAATCACCGCCAGGATTCATCGTACCAACAAAAATAAAATTTTCATGAGCAGTGACTGTTGCATTTTCTTCGGA**[C/T]**GACGGTTTTTCAGCAATTAAAAGTTTACGTTCAGGTTCTAAAAGAGAATTCAATCGCTCAAGTACACTATCATCTGCTAAAGAAATTTCATCTATCAAAA

>scaffold166.1.482630

TTCTCGGCGAATAATCATTTCGTAAAAATATTACCAAAAAATATTGTTTCCAAGATACAATCTAGTTATTTTACGATCCGATTTAAATTCTCCGCTTACG**[C/T]**AAGTACGCAGATCAGTCGAAGATTATATAAAAAAGATTATTCCCCGTGTTTTCCTCTTCAATTTTTTTCCTACGCGTAAAATGTAACAAGATAATGAAGA

>scaffold166.1.76592

ATAATAATAATTTTCACGATAATAGCATAAAATATTGCAATCATCACGCTCTCTAAAATGATAAAAATGCTCGTGTTGCTTTAAGATCGAAGAAAAACAA**[C/T]**GAAATTCACAATTTCAGAGTTCGAAGTCGCGATCTTTTATCTAAAAAATTACAATTTGAAGAAGAAACGGAGAAATTCTCGTTCAATTATCGAAAACGAT

>scaffold166.1.98341

ATCTGCAGATATAGCTTCCCACTCACCTGAAACAAAAAATAAAAAGGAGCACGGTTATTTATCACTTAGAATTTTTCATTAACCATATTATTCGATAAAT**[C/A]**GAATTCATTTGTGACAGGAAATAAAAATAAATTGTCATTCGATCAATTGTCTTTTAAACGACATATTGGAAGAATTCCCTATACGAAACGAAATATAAAA

>scaffold166.1.1150494

TCAGCCACTGTTTCTTTTATTTCCGCTACCTTTGCCTCTTCCTCCGATACTACCAACAAAAAATCATTTTCATCATATGATCATTTCTTAGAAATATTTT**[G/A]**TCGATACAAGTAACAAAGCAGATAAATCTTTTTGAATTGTTGCGAATTTAAATTATTACAATTCAAGAAATAAAATTTTACCAATCGCTTTTTCCGCTTC

>scaffold166.1.1177941

TGCGATAAATATCAAAAGTAATAATATTTTTATTAAATCATCAATTTTTAAAACTTTCGTTACATATAGATTCTTATCTCAATATACTCGTATATAAATA**[G/C]**ATCAAGAAATAAAGATTACAATTGTTTTAAAATTTTATGTAAACATTTACAAGAAAAATAAAAAAAAATTAATAGATCAATTAAAAAAAAAAACAATAAA

>scaffold166.1.1444824

ATTTTTTTCGAAGAACATGGTGCGTCTTCTAATTTTTACCATCATTTTGTCGAAGAATAGAGGTTCTTTTTCTTTTTTTCTCTTTTCTTTTCCCACTCAT**[G/A]**CATAAGTATCACAGATCCGTGAAATTGGGACGTATCGTATTGGAAAACTTTGGACCGTGTTCCTTCATAGAGGATAAAAACATTCGCTCGACCACTTTGC

>scaffold166.1.244323

GATTGGATGATTTATTACGTTACAAGAGTTAGCTGTATGAAAACGAAAAAGATTAGAATTTAATGGAATGCTAGCGTAAAATATTTTGTTATAAGTTGAC**[G/A]**TAAATTCATGAATTAATTATAGGAAATTTCAAGAGAAAAGTCGAATTCGAATCATTTGGCCAGATACAGGTAAATCTAACTAATTAATTATAGGAAGTTT

>scaffold166.1.285434

TTAACTTAAATCTAAACAAGAGTCCAAGAATTAATCATATGCATATAATATATAATATTATACTCTATCATATGATCATATATATGTTCTTTCTTGCAAA**[G/C]**AATTAGGATTTAAATTTAATATTTAACTTGGCTAATTAATTAAATCTTTTGATTTTTTTTATTTTTATTTTTCTACAGAAATTTTGTCCAATTTCTCTAG

>scaffold166.1.480384

TACCGGTTTGTGGATAACGAAGGTAAAAATAAGGCAGATCAACGGGATTCGAGAATCCACGGATTCCACGTCACGGTGATCTATCTGTTCATCTTTATAC**[G/A]**TTGTACTTCTCTAACTCGAACATAACCTCGAAAACCTAGGTTAATATTTTATTCATTCCTTTTCGTTCTGCTGTGAAAACCTCTACACTATTGTGTCTGC

>scaffold166.1.771654

TCTCTCTTCCTTTAAACGAATTTTTTTTTCCTACTTTAAATAATTTAAAAAAGAAAAAAACAAGGAAAGAAAGATTAAATCTACGTATTAAATTCTACAC**[G/A]**TTTTTACAGGGCAACGTTCAACTCCTCCATCTCCCTATGATGAACGAGAACCTCCTTCATTTGGACATTGGACAGGACTAAAAACAAAGAATCGACAGGC

>scaffold166.1.973769

GTATATAATTTTTTTAAATTTCACTCACCTGACTTCGCATAGGTTCCGTGTTGATTTGGCACATGTAAGCACCGGAATCATTTTTTTGAACGTTCGATAC**[G/A]**TGCAATTTCCACGTGTTATGTCCATTATGCGTAACCGAGAGTCTTGGATTATGTGCTATTAAATGTGTATGTATTGCCAAAATTGCTCGAGAATCTGACT

>scaffold166.1.1177384

CTTTCGGAATCAGATTCGTTCTTGTTGAACTATTCGGATGATTCGATCTCTTCCGGAATTTTCTTCATTACCGTGTTCGAACTTTGCTTTCCAATCATTG**[T/C]**ACGTAGGTATGATTTATTCGAAATTTGAATATAATTGATATCTTTATTTTAATATGCATTCGTTAAATTTAAAATTGAAATCTTTTTTTTATTGAAAAAT

>scaffold166.1.1230787

AAGCAAATTTACGTATCTCGAAATTCTCTAAATTGTATAATTGTGTACGAAAATGGTTATTGCTCGAAAATGGTTCCAATCTGGATACATTCGATTCTTG**[T/C]**ATCCGATTTTTCGACGGATACACGATTTGTCGCTTTGATAAAAAGAAAACTATTTTCTTTAATTTTAATAATTTTTTTTCTCTCTCTCTTTCTTCAGTCG

>scaffold166.1.1274416

AACTATCGGTTGTGGTACAAAATTTTGATAATTTTATTTTCTTCTGAAAATGTCAAAAGTGTTTTGTGGAATTATAAATAATACTTATATTTATCAGTTA**[T/C]**GAATTTATAAATTATGATTATGTGCGCGTGTTCCATCTTATTAGTGTTAATATATTCGTGTTTATAACATTATACGTGTTTTTTTGTCACTTCTGTTTTT

>scaffold166.1.381699

TGTACAGTCAACTACTTATTCATGGAACTGATCGACTTAAAATTATAATCTGTTTATTATTATTATTTTTATAGACATATTTTTGATAAACAAGTAATAA**[T/C]**AATATATTTGCCAAGTTCCTTTTTAAATTATTTATTGATAAATACACGATTTTAAATATTTTTTGTTACTAATTTAATCATCAATTTAAATATTTTCATG

>scaffold167.1.90782

AAAATTTACGAACTCTGTAAAAACAGAAGCAGAATTGTTGGCAGAGAGATATCCTATATCCTTTTATTTCATTCACTTTAAATTTTGTCATTGTTTTAAC**[A/G]**TTCCAAATATTCGAAAGAATAATTAAAAACGAATGGATTACTTTTGTGGAATTTTTACACAAAAGTAAAATTTCTTTCAATAATTGCAAATCCACCAAGA

>scaffold167.1.87284

TCGATTTGATAACGAGAGAAATAACCGCTGTAATTTTTGAAAATAAAAGATGTTTCTCATTTTTCCTTTTTTTTTTTAATATTAAAATTCCTCTTTTAAA**[C/G]**TAAGATGTCGTAATAATTGAAAGCATTAAGAAGAATTTTTTAATAACAGCGACAAAAAAAAAGAGTAACGAAAGCATAAACATGTCACGTGATTCATGAT

>scaffold167.1.24618

GAGCAAAGTCGGATTAAAGATGGAGAAGCTAGTAGAAAGCTTCTCTCGTATTAAAACTAAAGTTGAAAATGAAAGTCGAATCGTTTGAATATTCATCAGT**[G/C]**ATTGGTGTACAAGTGTTTTCGAGTCTCCCTTCTCGATATTCCATTGTGAATAACTCTTATTCGTATATTCCAACGTATGAATATAAACGAAACCAATAGT

>scaffold167.1.9255

TAATTTTGAGGAAAGATTTGATAGATGTCAGCATTGTACTTGAATAATTTTATTAATGTCGATAGATCGATAAAAAACAATATCAGTATTGATTGAGATT**[G/A]**TTACATTGAGAAATTTGTGTTGATAAAAAGAAAAGATAGAGGATAGGTTAAAAAGAATAAGGAGAAGGATAAATATACAGGAAAGCAATAGCTTGTTAGT

>scaffold167.1.99397

AAATTCATACGGTAATCGCTTTCCAACTCGAATATACAGGGTGATTTGGAAATACAGGAAGAGACACTAGAGGGGTGTGTCTCTTTGTTGGTAAATGGTT**[G/A]**TTCTCTCGACTCGACTCGATTAGATCTTTTTGGGTTTTTTAAAATCTGCGCATAATTTTCGAGAAAGAAAATGAATTCAAGATCAATTCGAGGAATCCTT

>scaffold167.1.9731

TGTGCAACAATTATTTCACAAATCCGTGTATTTTAAACGATCGTTCAATTCGTCTAATTTCTCGAGCTAAAGGTAAATCGTAATAATTCGTAGAAAAGAA**[T/C]**AAATGTTGCAGAAATCAAAGGATACGATCAAGAAATCGAACGTGCTATTTTTAGTTTTCAATCCTGGTGGCAACTTCATCGATATCCAGATAGGAGGCGT

>scaffold170.1.493335

CTACTATCTTATTTTTCATTTCAAGGATCGCAATATTCACGAGCACAGATCCACGGATCCGCATAAAAACCCAGCCGAAATTTGCATACGTAGAGCATAC**[A/G]**TACACACTATCGAAAGAATTTCGCGAAACGATGACGCGTAGGTAAGCGAGAAGTGGAAACAAACGAGGATTCTGTCAGCGACGTTTACAGCCTTGGGGAA

>scaffold170.1.834260

TATAATCAATTAATATCTATCTATTTAATAGCATTTAATAATTAATTTTATTAGAATAAAATATTTTAACACATCAAGTTGATAAATATTTATTTCTCAT**[A/G]**CATGTAAATAAATCTAGCAAAAACTACTGAAAATAATATCGATACAATAAATATTAAGCAGCTATATATTGCTAGAAGAAGTCATTTTTAATAATCCTTT

>scaffold170.1.170400

AACAAGAATCAGATATTCAAATTATAAATTATTTTTGATTCAAATTAAAAAATTATATTTGATTTTGATTTAACTATCATACACTTGAGGGTTAAGAAAA**[C/T]**GAAAGGGGGAAGAAGAAAAAAAAATAAAGATAAAAAAGTATCAGCGACTCGTGGACATTTTTGTTTTCTCTCTTTCTCTCTCTCTCTCTATCTATCTATC

>scaffold170.1.176098

TACCATCTCAGATTCACTATACCCAGTAACTGATGTTAGCTTGTTTGTTTACTTGACCGCACTTGGAACAAATACGGCTTTGATTTCCTTCAAATTTTGA**[C/T]**GAACAACGAGGAACGATAAAGTGAAACTGTATCTAAAATATTCATTACGAGTTATTTTATTTGAATTTTGTCTTCAAAGAAAAATGCTTCGTTGGAGTAT

>scaffold170.1.206045

CAGAATTCTATCTTTGCTAACATATTCTGCAATTATAAATAAAATTTTCAATAATAAATTCATAAATATTATATAATAGAATCTTTCGTATCCGAATTAA**[C/T]**AATAGACAAATAGAAATATATAAGTTTGTCAATTTGTTTCAATATTTAAAAATTTTATATTATTTTAATAACTCGAACTACGTATAAATACTTTGAGAAG

>scaffold170.1.314877

TTCTATATAAATTTTCATTGAATTATTAAAATTATATTTTTTTAGAAGATAGAATCCAATTTAAAAATATAAAATAATGTAGCAGCGAATTTTCAGTGTA**[C/T]**GTCAAATTTTCATCTGAATTGGACAACAAATTCTATTACTATTACAAAGAAGTTTAGAAACTTCTGTCAAACAATCTTAGATAATCCAGTTGATTCTTCG

>scaffold170.1.421438

ATGGTACAGGTATATAAAAATATATAAAGAAAGTATAAAATAACAATTTCTTACGCTAGGCTCTATTTTGGAGAAAATTTTTCAACTATTTGAATATGAA**[C/T]**AGATAATCTTGATGAAATCAGTTTGAATGTTATTCTACAAAAATAAATTAAAAATATAGGAGTGAATGATTTTCGAATTTGTTTTTGCCAAAAATAATAT

>scaffold170.1.542539

AGTTTTAATGAACTCGTACTTACATGCGTACACAATGTTTCGTTGGAATTACATGCATGCCAAATTTACTTCATTCCATGTATTTTTGTTCTGAGAGGAA**[C/T]**GACCTGAGATGGCATTCAAAGAAGATATCGTTTTAATTATAATTGGATGACACTGTCGAAGCATTATATCGATTTAACATGTACCATCTGTCAATAATAC

>scaffold170.1.608444

TTATTAACAATGATAGTCGACTTTTCGAAGAGTCAATTCAAAACATATAAAAGTTGAAATATTAAAATTTTGATTAATTTCTTATTTATTAAATTATAAA**[C/T]**AATTTAAGTTTGCATTAGACAGATAGAGTTTCTATTTTAAATATTTTTATAAAAATATCTTTTCTTCGCTATTAGAATTGTCTTTCTATATTTCGTTCCG

>scaffold170.1.750142

TGCCTTTCATGAACAAAACTTACCTGCAACAAATAAAAAGATCAATTTATATTATATAATAAAATAGTGATAAAATTTAAAATAATTTAATTAAAAATAG**[C/T]**ATAATATATGTGTATAATATCATCTATTAAAAATCAATAGAAAACAATAATTAAATGCATGGAATGTAACATTACATTCATTTTAATGAAAAATTTAATC

>scaffold170.1.75433

ATAAATATATGAAACATTACATTACGAATGCATATTATAAATATTTGTCATTAATTTCTAGTCTGCAAAATATTATTGTTTCGTTATATTCGTATAACAA**[C/G]**TATATGCGTATAAAATTAATATAGAAATGATTTTTAAAATAAAAAATTACCTTATAATGATAATGATTTTAAAGTGACATTATAAATTATATTATAAATA

>scaffold170.1.845649

AGCTCGATAAGACACATCTCCTTTTCATTTAAATAATTATATCTTGAATACAACAACTTTTTCACACTTTTTAATTTTAACATATTAATAAAGCCTTTCA**[C/T]**GAATGACATAAAATAACAAAAATAAATGTATCAAGTTTCTTCCCCTATGATTTAATACATAAATAAATGTATAAGTTTAAATTGTACGAATAATTATCGC

>scaffold170.1.357808

CTTGTATTAAAATATATTTAAATCAATACTTTTCATTACAGATTTTAAAAGATAATTGAATTTATAAGTTAAAATAAACAGTGGATATTAATTTAATATT**[G/A]**TTTTAAAATCTTTCGTTAAATTTATTATTGCAAAGATTAACAATACTATAACAATACTCTATAGTAAATAAATATTTGAAATCTAAGTTATAAGTTATTA

>scaffold170.1.358960

GACTTTGATGCTAGAGTTTGATGTTTTTAATTTTTAATGAACAATTAAAATATGGGATAAAAATTTATAATCGTATATGAATATATTCAACTAGTTTGTA**[G/C]**AAAAAATAATTTTATATACGTTTATAACAATATTATAACATTTATGTTATTAATAAATTTTGTTAATAACTGAAACACTTTATCTGGCAATTTATTATTT

>scaffold170.1.441390

TTAATAATTTCTAAACAAAATAAGATAATTACACACCTATATATCAGAAAATATTACATTTCCAAAAAGCTCAAGTTATTTGATCCAATTCTAAGAAAAA**[G/C]**TTATTCTATTTTAATGATCGAATTTTTATATAATTTTAAGTAGAACATAAAAAATGAATATTATTGAAAATGAATAAAAAGCGACGATAATCCTTTAAAT

>scaffold170.1.452507

AAGAAGATGTTTTCTATGGAATCTTGATGAGAAGTTCCTTTAAGAAGATGATTTTTCGGCTTGTTATTTTTCTCAACGGATGAATTGGTTTTGTTTATTT**[G/C]**AATGCGTTTGAAGAGATTTATAAAATAAAATTTGTATTCTAATAAAAGGAGTAAGATACTTATTATTTTATTCTACAATGATAACCTGATTGTATAAGAA

>scaffold170.1.607510

TTTATTTCATATTTTGTTTCGTTTTCAAATTCAATTATTTTTTATTTGAATTGTAAATTTTTAATATATAACAGAAATGTCATATAAAATTTTGAAAGCT**[G/A]**CTTATCATAAATTGAAATATATTTTACAAAAATATAGACGATTCTGAATATTTTCATCTTTTTATAATAATTTTATTTTGACTATGGAGTACATATTTAA

>scaffold170.1.188721

CATGCGTGTCATCTCCATCAAGAACTATTCGGATGACATTCGCGTTATCATTCAATTAATGCAATATCACAACAAGGTATTGTTATCATTAGATATTTTG**[T/C]**ATGTGATTTAAGTTTCCGTTAGAAAAATTTTGTTTAGAAAATATATATTTAATCTAATAATCATCATAATAATTTGAATAATAAATAATTAACAATTCTA

>scaffold170.1.426677

TTATTTTTATCGATTTTATACATTTTCATTCAGCAATTGACAATAATTTAATTTTGATCTTTAAGAAATATTGTTTCTTTCTATTTATAAATAATTTGGA**[T/C]**AAAGAGTGAGAATGTGAGAATTAAATTTGGAATAAATTAAAAAGCTATTTAAACATCATCATTTCAAATATTAATTTTACTGAAATTATCTTTTATGCAA

>scaffold170.1.675357

AACAATTTAATAAAGTATCTTTGATAATTTCCTGTCATAAAATATTATATCTCGAGAAATATATCAATTAATTATTGAAAACCGCGAAAAGAAGATAAAA**[T/G]**ACCGCGATGAAGAGGAATAACAATCTTGGCTAAAAAGCTAATTTTTCTATTAATCGAATACGATTAAATTTCTCTATATACTAAATAGAAAATAATTAAA

>scaffold170.1.676163

GATAAGTTTTTATTTCATTTTTATTCACGAATTTTTCTAATTTAAGACTGTCCATTAGTTGTGCCAAAATATCAAAAATGTTATTACTCTTCGTTAAGGA**[T/C]**AAATGATAAAATCAATTTATTGATTTATTATTGTTTTACTAATCGAATGTCAAAAATATCATGAGATGAATTTGGATTCATATTGCTCAAAATGAAAACA

>scaffold170.1.678861

TTTTTTCTTCAATATAATTGATAATACAATTGGCAATAAAACAAAATTTAATGATGGATTCATTAATTTTTAATTATGAAAGAATGACATTTCACATGCA**[T/C]**GAATTTTATTATAATGATAAATTGATAAAATTAATAAAAATAAATTGATAAATGTTAAAAAATGAAATATTTGTAAAATATTTGTAAAATTAAAGAAAAC

>scaffold171.1.2098244

GGAGACACCTTTTAACGTGCTAGAATTAATTCTGTGTCTCTTTCTAATTACGATAGAGTTAATTATTTAATAAGAAAATATTTTATAATCCTTTTATTGT**[A/G]**CAATTAGAAAAATTAGAAAAATTTATTTTCGTATTTTCTTCTTATTTTAATACGTGAAAATATTTGTTTATTGTTGCATTTGTTATAGCGTTTAAATATT

>scaffold171.1.2797620

GGAAATCACGATTCTTTACGAATGTTCGTAACAAACTTGAACGCGTATTGCACGCAATACAGTTCCTTCGTTTCTCTAACGCCTGTAAATATGCCTCGCT**[A/G]**CAAATAGGAACGATAAAAACCTATTTAACTTATTGTCTCTTCCTTTGAATTGATGACGAGAAACGAAGGTCGACGTTGAACTCATCGGCTAAGTATGAAC

>scaffold171.1.1530720

AATAATAAGAAGTTGTCGATAATGATTAATTAAAATAGTTTCCAACTGTTTCTGAAACGTAGAAAATTTAAAATTACTGGGATATTAGAATATTTAATAA**[C/T]**GTATCGTAATATTATATAATTTAACAACATTTATTATGGCAATTTAGATCTATTATTGTATTTAAATACTTTTTGATTACTTTATCATGACAGTAATTCG

>scaffold171.1.1695892

CCGATTTATTCGAATTATTAGGAAATCTCATTGAGAATTTTGTTCGTTGCAAAATCATTATTTTAACGTCAATAGTAAACGTATACTTTTTTGTACGTAA**[C/T]**AAAATATTAATTTGGTCTTATTGCAAGATAAAATATATAAATTACAAATATAAATATTATACGATATGTTCACGATTTATTCATATTTCTCATCGCGATC

>scaffold171.1.1750645

TGTTTATGTGAAAAATAAGAGAAATAAACAGAGATAATGATAATTTTGTTATTTGAAAATGGAAATTTTTCACATATGTTCAATATGAACATAAAAACGA**[C/T]**GATAAAAATTGCAAATTATGCTAGATTTAAGATATACATGAATAAATTATAAATTAGTATTGTACTTCTCTTGTAAAATCAAATGTGCGAATCAAATATA

>scaffold171.1.2450939

GAATAAAGAATAGTATTTAAACTTGAATATTTAAATTAAATTCCGGTATAAAAGAATAAAATAAGTAAACAAGATTTGTTTACTTCGCTTATTTATCGAG**[C/T]**AAAAGGAAATCAGTATGCAAATTAAAATACCTCCTAAAATAAAAAGTTATCACTGCGATAAAAATTACAAGATTCCACTCGATGATTTCAAATTGAAAAT

>scaffold171.1.2478237

TTATAACAACGGTGTATTCTCTTTATTTTTAGACATTTAGATCCAGGTAAACACAGTTTGATCCTGTAATTTTTGATGAAAAATATCGATTATCACGCGA**[C/T]**GATGATTAACTTTGGAATCTTATTTTTAATTTTGACAAAATTCTATCTATAGAACTATGTAATTAGGTATAAAAAATTTTATATTTTAGTAAAAGAAATA

>scaffold171.1.2758232

TGAAGGATATCAGAGGTAAAAATATATACGATGTGTAATATTAAGAGTTATTTATCGAGTTATATAATTTAATTTTCTTCTTGCATTGGACGAAGCGAGA**[C/T]**GTCTCGTCTCTCACCCTTTCTCACTTTCATCTAATGCAAATAGAAATTGACTTATAAGACAGCAACATATATTTATGCAAATTGAAATTTTTATGAACAC

>scaffold171.1.711666

ATTGCATCAGAGAAGTATATATTACCATATTAAGATATATTTTCATGAGATAGTAAATTATGATTTTATGATATCATGATTTTTATCATCGTCGCATTAT**[C/G]**ATGATATTAAATTTTGTATAATCACGATATACAATGCCATGCTGACATACAATGAAACATGACAACAAAAATCGTGAGATTTAATTATATATAAGCTTAT

>scaffold171.1.1481580

AGAAAAAAATTGTTTAAAATTTTATTCTGACAATATATATTTTATTGAAGCTATTCTAAATAATTGTTGAAATTTCTAATAAATAATTTTTAAAATTAAT**[G/C]**TTTATTATACTATAAATATTATACTATAAATAATAATTTATTATAATATAATAATATCTTGAATGATTGAAGAGTGTATGTAATTAATTTATTTAAAAAA

>scaffold171.1.1616530

TTATTTTCTATGAAAATACACTTTTGCAGTTTATTTAACATCAATTTTTAAAAATGAAAAAAAATGATTAAAGAAATAAATATTCAATCTTTATCATAAT**[G/A]**CTAAAAAAAGATATACTTAAAAAATTTATAATTCTTGATCGAAAGATCAAAGTTTAGAGAACATAGTTTCTATTGAAGACGTTAACCAGTTTCGTTCATG

>scaffold171.1.1695859

CCAAGCAGATCTTGCTCTTACATCACGCGAATACCGATTTATTCGAATTATTAGGAAATCTCATTGAGAATTTTGTTCGTTGCAAAATCATTATTTTAAC**[G/A]**TCAATAGTAAACGTATACTTTTTTGTACGTAACAAAATATTAATTTGGTCTTATTGCAAGATAAAATATATAAATTACAAATATAAATATTATACGATAT

>scaffold171.1.1734689

ACAAGATTGAAAAAAAATGCTCATCGTCACATTCTGATTTGAAACATCGAGTAAATACTTGTTCGTTATATCAACACTTTCCAGAAATAAATATATACAT**[G/C]**TATAATCTCATTATGTACAGCATGTACGATTATGTAAAGAAAATTATTAAAATTATTAATGAACTCTACTAAACTTTGTGTATTTTATTTTGAAATGTAT

>scaffold171.1.1910150

ATTTAGAATAGAATTTCTGTTCCATATTTAAAAAGTTAAAATGAAATATATAAAAATGTAGTTTATAAATTATATAAAATTAACAATGTTTGCAAAATTT**[G/A]**CCGTTATTAATGAAAAACAAAATTGTCACGATATTGGAAAGAATAAAGATATACATTTATTATGCTTTGAACGAATATTTTATAAATTAACTTTTTCGGA

>scaffold171.1.1966947

TTAACAAAGATGTAGATCGTTAAAGTTAAACATATGCTATAATGGCTGAATATTTTTGCGAGTAATTGTGCATGATAAGTAAGAGAGACAGATATATCAT**[G/A]**CGACGTTAAGTTGATAAAGTACTTATCTGGATGTAATATTAAAATCGTTATACTTCAGTCGCGTGTCATTTAAGTGTCCTTTACCCACGTCAAGAAAATT

>scaffold171.1.2100031

AAAAATTTAACGTCGAACGATCGAATCACGAGAAGACGCGAATTTCAAACATTTTTATTGTATTTTGATTCAAACCCTTAAACTATCTTTCTTGTTTCTT**[G/A]**CCATTTCAACGGCAGAATTTTTTCCATATTTTATAACAAATATGTAGAATATTATATAAATTCGTAAATGTGAAACGTATGTCGAATAGGTTAAAAAGTA

>scaffold171.1.2433698

TGTGCACGTGTTACATTCGTATTATTTCTGAAGGATTGCGAATCGATAAAATATCTATCGAGAATCGGTAACAAAAATTATTCTTGACAAAGTGGATTTT**[G/C]**AAGTTTGAAACTTATTTTCGAGTAATCATTTGCAAAAGAGCGATTCTTAGCAGCTTTATCATCGAATGAAATTAAATATCAATCTTTGTTTTCAGCGTCG

>scaffold171.1.2450925

ATTTATTTTCTACAGAATAAAGAATAGTATTTAAACTTGAATATTTAAATTAAATTCCGGTATAAAAGAATAAAATAAGTAAACAAGATTTGTTTACTTC**[G/A]**CTTATTTATCGAGCAAAAGGAAATCAGTATGCAAATTAAAATACCTCCTAAAATAAAAAGTTATCACTGCGATAAAAATTACAAGATTCCACTCGATGAT

>scaffold171.1.2698562

CTTTTAATTTTTATCCAATTCCTATATAAATAATTATAAATCATGCCATTTCGCATGCAACGATCTAATACATGCATAATCCATTCAAATCATCGGGAAC**[G/A]**TCACCGAATCACCGAGCAATGACGGCAAAACTATAATTAATTGCTGTCATTTCTGAACGCGAGAGTGAACTTAATCGTGAATAATTTGAATCTAATAGTG

>scaffold171.1.671981

AATATTCCATTTAAAAAATTGAAGAATTTTGAATCTTGGATAAAAAAATATTAGAAATATAAAAAAAGTAAATATGCCATTATGTTGATCAAAGGAAAGT**[G/A]**CAAACTTTTTTTCATCTTTTTTTCACTATCTTTTCTATATCCTTTCTTTTCTTGTTCTTGTACGTAATATTTATGACTAAAACGCTGATTGTAATATATA

>scaffold171.1.1684278

AAAAATAGTGAAAAAAATATGTATAATTGTTTAATACAAAAAGATACTATTCTTTGATCTTGATGATTTTTTAATATGTCCTCAACATCATTATTTTGAG**[T/C]**AATGCATAGTTTTATTTGACGATTCTTTATTATAAAAAAATTATTAAAAACGTTTCTTCGATACAATTTAAGTTAGATTATGTTTTGATCAAATTAATCG

>scaffold171.1.1786492

ATTCATTAGATCAACAAACAAATTTTATTTGTACGTTTAATCACTATATTCTGAATAATTTTATATAGAAAATAGAAGAGAGAATCTAATATAATTAATG**[T/C]**AATTACGATAATAAAATTATATAAAAGTTACGAATATTTCTATATTTTTTAGATTGACAAATAATAATTCATTTCACTTTTTCTTCACACTGAATCCTAT

>scaffold171.1.1939657

ATAATATAATAAAATAATAATAAGAAATTGTCAAAAGTAGATTTGCACTGTTTTTGTTCGGAATCTTCAATTGTATCTAATTATAATAGCATGTTAAAAA**[T/C]**GATAATAATTAATCTTAATTAAGAAAAACAATTTTTGAGATGTGTCAATCATATTTTTTAGTGTATCGTTGAAATTTATATTATATAAATCGGATTTAAA

>scaffold171.1.1963520

ATCTATATTGTAGACAAATATTTTATTTCAATTATAAGTGTCTAAACTTTATGTTTGTACTAGACATTGCAGATTTGTAAAATATTTTCAATAATTATCA**[T/C]**GTATATTTTAAAAACTTCAACGTGATATTATTTTATTATCTCTATATTATTCTATACAAACTTGTTCTTTTTAAACATTTTTCATTATATAAAAAAAATA

>scaffold171.1.2012149

TAAATAATTTTCATGGTTATAGCAAACCAGTAAGACTTAACCTAATTATTAATCTATTAACTATTAGATTACACTGGAATTAAGTATAATCCTAACCTAA**[T/C]**AAGCTTTGTAAAAATTTTTTGAAAATATCTCGAAAATAAGCAATAATACATATGTGCATATAGAGGAAAAAATTTACTTGGAATAATTTTAGCCAACATG

>scaffold171.1.2046998

TTGATGAATTATTTATTCATAAAAAATTATATAATATTTAGATCTTTTTATTGAGTTCTTTGATTAGTTGAAAATATATTTTTATATATTTATATAAGTA**[T/C]**GTACATATAATAACATATAGATTTAAAGAAAATATTATATATTTTATATAATTATACTTTTTTTTGTTAATTCTGTGATCAAATATCTATATTATTTTAG

>scaffold171.1.2142302

TACTCTTATGATGAGCAAACTAAGTGATCAATCGAAAAATAATTTCTTTTCACCAGAATCAGTATTTAAAAACTGGCACGACGCTGTTTAACTTGTTAAA**[T/C]**AGAAGAATTCTTTTTCTAATTAAAGAAATTATTATCAAAAAATAAATTTATTTTTTCTTTTGAAATTTCTTTGAAAATTTGGCTTATCTAGAAGATAAAT

>scaffold171.1.377422

TATTAGATATCCATTTATTGAATATCTATATATATTTGTTACATGCCAATATAGATGAGTAAGCATTATAATCAAAATGTTATACAAATACATACAACGA**[T/C]**GATACGCAACGTATCATTCTATGATGAATTGATATACGAATACACATCAAACATATAGTCTTATATCGAATGCATATATAAATTCTATGTAAAAGAAGAA

>scaffold171.1.918275

CGCGGAGCTAAATTCACTTGAAGAATTTGCTCTTTTTTCCTCTAAAATTCAATTAGTGGCGTAGAAAAGCAACATTTCATCCCGCGTATTTTCACATCTA**[T/C]**GAAAAATAGAATCGTAAAAGAATAATTAATATTTTTGAAATATAAAATTACAAATCAATTTTTATAAACTATCTTGATTAACGTATCAATCAATAAGACG

>scaffold174.1.1046144

TTTTATATATCACCAATAATCCGTACAAAATATGCTAATTCCAAATAGAACAACGGAAACTATCCAAACTATAAATCTAAACTTCAGAACAGAAAGAAAT**[A/G]**CTTGAAAACGAAACACTCTTTTAGAAATCTAAGAATATACTTACAGAAGAACAACAAATCTCTCCCTATAGAGAACCTATGTTTTAACATAGAACAAAGA

>scaffold174.1.1220946

ATAAACTTTAAAAATCAAAACTTCGTATAACAATTTATTTTGATCTCTAGAATATCAAATATCGAACGACGACATGCGTGGTAATAGGGGAAAAAAATCC**[A/G]**TTATAAAAGTACATTTAAACTTCTCTTACTCCTTTTTCTCGCGAGTTTAATCGTTACAAATAATTTAGATTCTAGATTCAAGCGATACATGTTGCGGATA

>scaffold174.1.1221020

TGCGTGGTAATAGGGGAAAAAAATCCATTATAAAAGTACATTTAAACTTCTCTTACTCCTTTTTCTCGCGAGTTTAATCGTTACAAATAATTTAGATTCT**[A/C]**GATTCAAGCGATACATGTTGCGGATAAAGAAATCTTCGGACGAGTATTTTCTATGCGCAAGAGAAAAATAAGAAGGGAGAAGGGAGTGGAGACTCGACGT

>scaffold174.1.1760890

ATCGATAATAAAACGACGCGACGTCGCACAGCATCCAATCTTTGCCAGAATGGAATCGTTTTCCTTTAAATGGTTAACCTCTTCGGAGAAACTAACGCTT**[A/G]**TTTAAATTAAAATTATTCCAAGTAATATCTGTTGAAGAATTCCTGTCGGAATCTTTTAGTGGTGAAAAGAGAAAAAAAAATTGTGAAGAATGTCTTATAA

>scaffold174.1.1863029

ACAGACAAATGATTGTTCGAAGTAAAAAATTCTATGTATTGCGTTCGATGAATTTAAAATTCACAAAATTCGATTCACACTGTTCTCCTTTCGGATTGTT**[A/G]**CACGTGTAAATAATTCTTGTTAAACCCGGACAATCATCTTCGAAATTAAAAATAGAGAACGTTGAAGAAATGTTTTTCATTTCTTCGAATCGTGTTCTTT

>scaffold174.1.2231161

CTAACCTCACAGAACACGTGCAATCGTATTTTTAGTGATATATCGAATCGTATCAAAGTATAGTGTCATTTGTGGGAGATTCGTTTCAATTATATTTCTT**[A/G]**CTACTGATGATATCAGATTGTGTCAATTAAATATTCGATTTAGATTCATTGAATAATCTAAATAGCTGCAATCATATTTTGATGTATCGAATTATGGCAC

>scaffold174.1.2385061

TCAACCTACCAATGAACGGTGTCATTAGATAGGATATTATGGCGCATATGGCAATAGTGGTAACAGAGGAGATGAAAAAAATGCAAATTTTCTTGCAATC**[A/G]**TCCAAAATTTCTTTCTCGTGATAATCGTAGTTGACGTTCCAAAAATTTTCTTGCATGTTTACGATTAAATTCAAAAATTTTCCTTTATATATTATTATGT

>scaffold174.1.727165

ATATATGTATATTTGAAAAATAAAATTATTAAGATTTATTAAAAATAAAATTATTAAGAACAATTCATTTTTGTATTACTAAACTAATCAAATTATACTC**[A/G]**TTAAAAATCACTAAAAATTTATTTCTTGTCTCTATATAATAATATTAAAAATAGTATGATATAGAAGACTCTATATCATAAAATTCTTTAAATTATAATA

>scaffold174.1.1199601

TATTTTTATAAAATATTATTATTAATTACCATTAATTAATTTAATTAATTATCATCGTAATATTAAATCAATATGATTAATATAAATAATTTATTTAGAG**[C/T]**AGTTTCTTTACATTAGCTAATCATATTTCGACATATCTAATTCTTTCCATACATCCGTTTATTACATCAAATTCGAATTACAAAGTAAAATAAATTCATA

>scaffold174.1.1257906

TTGTTTAAAAATAATATTTGGCTAAATAAAACGATATACCACTTGAATCTTACTTTTAATATTTATTATTATTATATTATAACATATTAAATAATATTTA**[C/G]**AAATAAAAATGACATACATTATACATTTAAAAAGTCATTGTACGAAAAACAATATTTGAGCATTGCAAGAAGAATTTCTGAGAAACTATGACAAACAGAG

>scaffold174.1.1292250

AGATTGTTAACTACATTAATAACTTTTTTTTATAATATAATTTCCTTAATTACATATTTCGTGTGATTCTATTAAAATTTTAAACGATTATTAAAATTGG**[C/T]**AACTAAATATTCATTCGACCCAAATATCTAATTAATAATCAATTCGTGATATAAAATAATAATCTGTAAAAAAAAAAATCTTATCTTTCTTCTTTTAATT

>scaffold174.1.1310330

CACAATTAAGTAATACATTCGTAGAAATATATCTTTAATATAATTTTCAAGTAATTCCCATTTCTTATTTAAAACTTATTTGCGAATCATTTACAAATTT**[C/G]**TATTATTTATATTTAATTCTAATCATTATTAGTATATAATCACGACTTCAATGATAATAAAATAGAAATATACATAGAAATATATTTTCAATATAATTTT

>scaffold174.1.1405233

CTTTATAATATTGAATAAATTATTGTTATATCGTAATATTCCTGCAAAAAATCTATAAATCCTTTTCAAATTCTTCTGGACATAAAAAAGAATTATTAAT**[C/G]**AAATAAATGATTATTTTACTTAAATTATTCACTATTATCTTAATTAATTATTCAATTACTGAATGAATTAGTAAGTACATTATTTGTTTATAGTAAGTGA

>scaffold174.1.1423400

ATCACTTTTACCATATTAAATAATCCATTTAGGTTAATTGCAGATTCAAGGATTCAAGTTTATGAGAAAATTTAAAATCCCTCTTCTCAATGTCCTCTGA**[C/T]**GGCATATGAATAAATTATAATAATACAGATATAATAATTCGATACGTGTACTAAAAAATAATAATAATAAAAATATAGAATATTCATTTTATTTTTTGAT

>scaffold174.1.1509561

CTTTACAAAAAAATATTTAGAACATAATACTCTTGACGGATAAATCGATAATTGGATAATTTATCAACTCTGCCCTCCTTTGAATTTATTAATTTTAATA**[C/G]**TACGATCACATATCGGATCATTTTGAATGGATCAAAAGGGAATGATTCTTATATTTAATCTGGAGCAAGGATCTCGAGGAAAATCAATTAAATCGACAAG

>scaffold174.1.1787956

GATGAATGTAATTATGGAAAAATATATAGAAGAAAATTAGATTATTTATTTCATTTCATCGATTTCAATTTAATGATGAATCGAAGGATTATAAGCTCTG**[C/T]**AATAAAAGATCTTCAAATTGTAATATTCTTTTCTGATCTAGTTATTGAATTTGAAGATTGTTTTCTTTGAGCTGTCCTACATTTGTTCTTGAAGAAAAGT

>scaffold174.1.2022097

TTTTATTATTTCTGTTGTGTTTTATCTATTTTTACGAAACTCCGTCGATTTGACGGGTTAATTTATTAACGAAAATGAAGTAACAATGAATAAGAAAAAA**[C/T]**AATATCGAGCAAATGACGACTGTTTAGAATTGTGACATGATAAATAATTAAATAATCCCCTCAACCAGGTGAATTTTGCAGAGAATAGAAGTGATTTTTG

>scaffold174.1.2129724

AACGTTGATGTGTCGAGATATTTATTAACAAGGATTTGATCATGATTGTTGCGATAAAATATTCGAATATTTAAAATTGTTGACGATTGTAGATCGTGAG**[C/T]**AGAAGAAAGTTTTATAAATTGTTTTTTTATCGAACATCGATGTTATATAATTTTAAAATACGAAGAAATATTCGAATATGTTTTTTTTCTTTTTTAATCT

>scaffold174.1.2280253

AAGCGTCCAATTGATATAAGTTTGTTAAAATCTTGTTCTAAGTTGTAAGCAATTGGACCTAAATATAGAATTATAAAATAAATCCATTAAATTCAATATA**[C/G]**TTATAAAAATTATATATTTTTTATTTACCTTTAAAATGCAATAAAAATTGAATACATGCTCCATTATATAAAACTTTAGTTTTTCTATTATCTATAGATA

>scaffold174.1.2290172

TTATTATACTGATCACATTAAGACGAGTTCAAAATCATTGAAGACAATCTCAATTGGATTATTCGTTATTTACTTTAGAGTTTCAAATAAAAGTATTTTG**[C/T]**AACATTGTATGTCGAGGCTTCGATTCATAATTAATAAGAATAATAAAGAATAATATTTTTTATCGATTACATCCACCAGGCATTCCAGCTTATGGATCAA

>scaffold174.1.597089

TAATTGCGGTTAAAATTAGCTAGCTTAAGGTAGCTTTTTGTATTATTAAACAAAGAATACATAGATACATATGTAAAATTTAAATACGTTACAATAGTTT**[C/G]**AAGAAAAAGTGTTCTATTTTGTTTTTGACTATATATATAATCTTATAATTATGTTTCTATTACACGTACTTTATATAAGATAATTATATATAAATTCCAA

>scaffold174.1.966231

CACCCCCTTTAAAACTTCTCCATCCAATGCACCCATCAAATTCATCCCCCTTTCTCTTCAAACTTTTTTCTCCAGCCACACCCCAATTAACCCTGTACTA**[C/T]**GGTGAGTAAAAAATGACAGAATATCCAAGTTTAATTTTTGTCTTATAATTATATGCTATAAAGATCAGACTATTAAATAGACCAAATCAAAAAATGATCT

>scaffold174.1.1043878

TTAAAATAATTACTGATTAGAACATAGTTTAATTTTCTCTTAGTTATATTTACACCATTATAATTTTTAAGTTCTCTATATAAAGTTTATATATAAATTT**[G/A]**CTTCATTTTTCAAATATCATAATATATTTTGTGGTTTTTTTATGTCGTGTATATTTCTGCAGATTTTTATACATTAAAATTTTATATTTTTGCATAAATG

>scaffold174.1.1088605

TGGCAGACGGAAGTAGCAGACGGCTGCGGAGACGATGGCCTATACGAACACGGAAATACTACGTGATGTTGGCATGTGCATAGTTGAACCACGCATACAT**[G/C]**ATATACACACGTGTCTCTATGTAAAATACGGTATACTAGCCGCGTGTGTATCTTGACACGGTATAGATATTTTTGGGTGTGAGACACTGAGCTTTCTTAC

>scaffold174.1.1258623

AAGAATTTGAACTTTTTAAATATATTCAATTATTCTGTTTGAAATTCTGAAATTTAAATAAAAAAAAGAAAAATTAGTATAGATGTATAATGGTGTCATT**[G/A]**TCATTCTTAAAAATTATATTATCTTATAAAATTCAATTTTAATTTATACGTAGCTTTCAACGTTAAAATCATTAATATTAATGTATGCATAGGTATACAT

>scaffold174.1.1524539

TTAAAGTTTCTTTATCTCATAAAATTAAAAAATCGAATTAAAAAAAAAAGATATTAAAATTATGAAGTAATGTATCTCTTATTATTTCATTAATAAAATT**[G/A]**CTCCATATATAAGTGCTTACTCTTTAGGTAATCGTAAAATTTCATACAAAACAAAAATTACAAAAATTATTGTTTGTTTTTATAATACATAAACTTGTTA

>scaffold174.1.1779887

GTTAGATAATTCCATTCCATCATTGATGTTTTAATATGATAAATGAAAAATTCATAATTGTTTCTAAGCAATGGAGTTCCTAACAAAATTTCCACACATC**[G/A]**TTTGAATTTATTTTCAGACGATTACAAACGTCTAGATCTAAAACTGTTGCTTGAAACATCGAAATTCACAGAGAAGGATCTTCTTAAAATATGAAACACT

>scaffold174.1.1784068

TCTTATTAAAGTTTTAAGATTTAATTTTATTTCCTATCTATTGGAATTGATTTAATGTCAATGGATAATATAAATACCGTAATAAAGTAAAATCAATAAA**[G/C]**AATATTTCTTATATAATAATAAATTCTTGAAATAATGAATTATATTCCAATATTAGAATCATTTCATTTATTATTGATTTAAATTAAACAAAATTTTTTC

>scaffold174.1.2232589

GTCTGATCCAATCTTTGGATAATACATGCGCGTTAAAATAGGTGCGAAGAGCCATCTCTGAGACAGCATAGCTGCGAAGATCGGCGAACGATTATAAGAT**[G/A]**CTATTGCCTTTCTCTTTTTGTCTTTCTTTTTTTTTCCTCTCTGTCTACCGAACTACGCACTTTTCTACTTTTTCCCCTCTTTCTCTCGCGTGGGAATAGG

>scaffold174.1.2423293

CATCTCTTACTTTCTTCAGTAAGAGAGATAAATCTTACATCTTCTTATCAAACCATCAACCTTTTCTTACCTTTTTCTTAATCTCTAGAATTATTTTCTT**[G/A]**CTCGTTCAACAACAATATCATTTGCAAAACAACTCTCAAATATATTCTGAATCGTCTCCTTTTCGGTTCAACGATGAACCAAGATAAAAATCGAACAAAA

>scaffold174.1.290474

AATTTAATTATTAACTTCGTTTATTTTATTTCTATTTTCATTTTATTTCTCTCCGTTATTCACCATATTATTATTTTTATATTATTTATAATTTTATCAT**[G/A]**CAGAATTTAAAAAATATCTATAAATACCTATATAGTGTCAAATATTTCTATAAAAGTCCAATAACATATTTAGAAAATAAAAACAAGAAAAAAAATATTA

>scaffold174.1.804407

TGTATTAGTTTAAGAGACGATTTTTTATATTTTTAAATATTCTTTATCACTTTCAATTTTTGATTTAATCGTTTGAAAAAATTTATAGTGTTAAATATTA**[G/C]**TATGATACATTTTAAATATTCTTTTTATTTTTAAAATGTTGTTATTTTTAATAATTTAGTTTTCGTTTTATATTTATTAGACTTTATACTTTATTATCGA

>scaffold174.1.968744

TTATTTTTTCGTCATTAATACTTATGATATGCCTCTTTATTTATAAAGAATTTATATTAACGATACAGTTTATTATCATTTTTCAAAATATTTAATTATC**[G/A]**TTTAACGAAATAAACATCTCAATAAATATTCCACAATTTATTCAAATCTCTCGAAAGAGATTTCTAGAATAGTAAAAAAAAATAATTGTAAACATAATGC

>scaffold174.1.1405241

TATTGAATAAATTATTGTTATATCGTAATATTCCTGCAAAAAATCTATAAATCCTTTTCAAATTCTTCTGGACATAAAAAAGAATTATTAATCAAATAAA**[T/C]**GATTATTTTACTTAAATTATTCACTATTATCTTAATTAATTATTCAATTACTGAATGAATTAGTAAGTACATTATTTGTTTATAGTAAGTGAAACAATTT

>scaffold174.1.1534039

TCAACTGCACTGAAAGAAATTGCCAGGTTAAATTATACTTAAAAAAAAAATGAAGAAATGTAGAAACTTTCGAGGTTGATTTCATTCTGTTTGTCTTGAG**[T/C]**AATATTGCTCTACTATTAACCGTAAAATAACATTCAAAAATATTTTCACGATCCAAGATATATTAAACGTCAACAAAAAAGTATCGAATTACATTGATAT

>scaffold174.1.1783296

TTTTTTTTATAATTATTTGAAGTAATTAAATGAAGTAATTATTTTTGATAACAATTTGATATATATCTGTTTGTATAATCTTAAACATTAGAATTTAAGA**[T/C]**AATTCATTATAAAAATTTGCTTAATTTAAAAGTAATTTAAAAATAAATCTAGTTTACTCTATTACTTAAAATTTTATAAGAATACAATTTACAATAAGGT

>scaffold174.1.2353343

CGTATTCACGATGAAAATTTCTTTTCGATTTTATAAAAATAGCTATCATTATATCTACCTTGGTATAAGTTTCCAACGATACAGGGAAAAATCCATTCGC**[T/A]**GTCAGACAACAAACTCGTCTGGATCGCTTAATCACCATTATCAGATCTTTTCGAAGCATTCTCCCAAATTTGTCCATCGGCATGATCGTCCACAGTGCCG

>scaffold174.1.2430855

CGTACAAATTTTCATGGATTAATGACTCGAGAATATATTTGAATATTTTCTTAACAGAAACTAGTTATCATTTAATAAATATTTCATTAAAGTTGATATC**[T/G]**ACGCTTCTTTTTCAGAATTTTGAGAAAAACAATCTGTCAGATCGATCGGAACAAATTTTATCCCTAAGTTCAACATAATGGAATACATTAGAGATTTCAA

>scaffold175.1.199569

TGAAGATAATGAATTTAATCTTTTAATCTCAATTAATATCTAAAATATAAAAATGATATAAAAGTTAAATTTTCAATTCAAAATTAAGATACACAACTTT**[A/G]**CGCGTGAAAATTTGTTTTACATAGATACGAATTACAATATAGGCATCATTTTCCAGATTTTTATTTTTATTTTTAAACAAAAAAAAAAAATCTATAAGTA

>scaffold175.1.342806

TGAAAAATGTTTATAAATTGTGTAAAAAGATGTTGTTTCAATTTCGAATATATTCGTATTTTCATTAATGTACCAATATTCCAAACTGCGATTAAAATTA**[C/G]**TAACTATTAATTAAACTTTATCACCGTGAGTAATTAACGATTAATTGGATTGTCTTGCGATATCGTATTGTATGTTAATATTAACAAAAAAAAAAAAAAG

>scaffold175.1.345319

ACTTGATCCAGCTTCATAAATTTTGATAAATCTTTTATTTTCATTTTCACCTCGAATATATTAAAAATTAATTCATTTCGAGAAAATGAAGTTTTTGAGA**[C/T]**AGAAAAATTACCTTAATTGGCAAAGCAAAAAATATTTTCTCAGTCATCTATTCTTGATGATTTAAAAAATATCGAATTCAAAGTGATTCTACTCTTTGAT

>scaffold175.1.516594

TGATATACATATTGTATATTATAAACATTATAAAAATATTATATATTTATGATATATATATTATATATATAATTATTGAGATATATGTAATTATTACAAT**[C/A]**GTTGTTAATTGTGTTATAATGATATTTTAAGATGATAAAATTGGCAAGCATTATCTTTTTTCTTTTTCTTTTAGTTTTTAAATTTCTTTCTCTTCCTATA

>scaffold175.1.704674

ATACATATTTTCGTACCACGACAAATATAAAATAATAAGTTGAGAATTTCGAATAGAGCTACTTTAAGTAATTACTTAAAATGCTCGAATAATATTTATT**[C/A]**GCATTCCATTATATTAGACCTGTTTTTCCTGTTTTTATAACCTCGCCATTATAATTATTTCCAATTATTATTTTGTTCATCGCTATATCAAAAGATTTTT

>scaffold175.1.1024768

GAATTGTTCCTGTAATAGGATAAGATTTTATATTTACTCGAAGAGATTCGTTCGAGGATTGTAATTGCAATCGGGTTAAATCTCGAGGTTCTAATATTTC**[G/A]**TTGAAACGAATTTGTCAAACAATAATCCACAATTTATCGTTCCAATCAATGAAGAAGCCTGGATTAAATCTCTGATAATAGGACAAATACTTCCTTTTTC

>scaffold175.1.454360

AACAATTACGTCAAATGCATTTTACATAGCTGGATCAACAAAATAAATGTAATTATTCTACATGTGTTTTCCAATCTTTTTGGTTTCATATAAAACAAAT**[G/A]**CGTACTTTTTTCATATAGAATATGAATATTTAGTAATTTACTATATATACTAACTTATTACTTCAAATAATTCGTTTAAAAAATTTCGTATACAAAAATT

>scaffold175.1.612410

TTTACGAGAACAAGTATATTAAAAGAAAAGAAATGTTTTTTTGCAGCAATTTTACTTTGTTAAAAAGAATAAAAAACATTAATTTCAAGATTACAAATAC**[G/A]**TTCTTTTAAATATTATCGCCTTATCCTTCACACATTTAATCACCATATTTATGCAAATTGAATTTAATAACTTTCGATAATTGATAATAAAACACAAATA

>scaffold175.1.667166

ATGTAAACGGCCTCGTAAATCAATATGACCGCCAGCTTCTCTGCGGTTTCCAGTCCAGCGCGTCGTTTAAATCGTAAGAAAACTTTAAAATATCGTAGGC**[G/A]**TAGAATGTTATCTGGAATTTGGTAGAATTTCGTAAACTTATTGTTTCTCTTATTGTGTGATGGAAAACCGCCGATTAAAAATCAAATTTGTGTAAAATAT

>scaffold175.1.82779

GAATTTGCCCAAAAAGCGAGTCTTTCGTTCGCGAGTCTTTGCCAATTTTATAAAAGACACGACTCCTTGTCCCCACTTATGAGCAATCTTTCTTCTTTGT**[G/A]**CTTTGAAAATTTAAAAGAAAAGAGAAAGAAATAATTCGTTCATTCAAAATAATTACAATTTCGAACATTATTAGAACGAAATTAAAATTAAAATTCTATA

>scaffold175.1.894128

ATAATCGATCTTTCCATTCCATTTTTTTTTTTGAGGAAGAAGATGACCCCACTTTAATGATATCCATAAATATCATTGTTGAAAAATATATTACCATTAC**[G/A]**TTCTCTCGGCATTCAATAAGATTCGTTCTATATTTTAATTTTCAATATTAATTTTCAAATTTTAATTGAAAGATTGTTTATTTTCGTAATATTGTTTCTA

>scaffold175.1.265945

ATATCTCGATCCACAACACGAATTTAAACATTAGCAAAACTGGTTGTTATTGTTTTCTTTCATTGGTTACCTGATCTATTCGTAATTTGATTTAATGGAG**[T/C]**ATTTAATTTGATTGACGGCCATTTTGATGCACGTGAAAAAGTAGAAAGAACGATAATTTGAATAAATAATAATAATACAATGATACGACATAGAAATCTC

>scaffold175.1.452475

TTGTCGAACAAAGACGATAAAAATATTTGAGATGGTGAAAAAATTTAATACGCTTCAATAAGAACACGAACATCGAACGTTTATCGCGATCGAAATTAAA**[T/C]**GACAATGACAATTTACACGTGTAAGAGCACACAATTATTCTCGCCAATATTTTATACAGTTTCGTGCAAAATCCATTATTCGATCTAATCGAATTTTAAT

>scaffold176.1.26792

TAGTTATTAGAACTACTAATAACCATTAATATCAATTAATAATAAAATATTTATTATACACAATATCAAAATTAAACTACATTGGTCACTATTAGTATTT**[C/G]**AATATTTGGTGATCGCCTAAAAATACTATAGATCTATATATGAGAGAAAAGTAACGAAAAATGGGCAAAAGATCATTTTGAATATGAGTGCAGAATTCTG

>scaffold176.1.34644

TTGACTCCCAATCCTATCAAAATGATGCGCTCAAAGTGTTCATTATGCATACAAAACGAAACGATTTGATGAAGAATTTACAATCTATAACTACTTTCGA**[C/T]**GATTTATTATTATAAAAAGCTGTATGATGAAATATAATAAAATATTGATTCTTGAGTAAATAAGAGGACATTAGAATCTTTCAGTTAATTTAGATTAATG

>scaffold176.1.85107

GTCAACGTTTTTAAAACGTATAACCGAACAAGTTGATCAATATTTAACTTATATTTATCATATTTACATTTACATTTTTTAAAAAAATATTATTTTATAG**[C/T]**GGAATAAAATTTTACTTTAAAAAGTGACCTTTGCTATTCTTACAAAATTTCCTTCAGAAGAGATTATCTATAAAGAATAATCTAAAATATTTTTATTTCA

>scaffold177.1.125379

TCATTATACTCCTTCAATTCCAAAATTTCATAAATATTTTCAATAAACGTACGATTCGTAAAGTTTCAATTCTAAAATATCATCAATCTGCAGATAATAG**[C/T]**AATTCATCTAATTTCTATCATTATACTCCTTCATCGTCAATTTCAAAACTTCAAAAATATCTTCAATAAACGTATATTCGTTACTCATTATACTCGTTCA

>scaffold177.1.155908

TGATAATATAAAAGAACTTTAATCGTGATTAATATTTTATATCTATTTATGTAGAAAATTGATCAGACAAAATGATAAAACATACAAAATATTTTTAATT**[C/G]**AAAATGGAAAATGGAAATCATAGAATACAAAAATATTGTAGTAGTATTGTACTTTGTATAATATATAATAAATTTAATAATATAGCATGGTGTTTAAAAA

>scaffold177.1.237882

CAACTTTTCACGGGCAAGATTACGTTCGATTTTTATCTTTTGCGTATATTAGAGATAATGACAAACTTTTTTATAATTCGATATGAGTTGATCAATCGAA**[C/T]**GAAATAATTATCGACATGCATTAATACATTTTATCTTTCTTATAATTTCGATTGAATTAATTCTTGCTTATCGATAGTAACTCAATGATATACATCGCAT

>scaffold177.1.115400

CAATCGATAATATTTTAAATATTACATTAATTCTTCTTTCGTTAAAATAAAATCTATCTCCTTTTACAATAATATATATATGTATTTTAATAATATTTTA**[G/C]**TGATTCATTTCTTACGAAATTATTTCAAAAAAATTCACAGAAATTCAAGAAATTTTTTTTTTTAACACTGCTTCTTTAAGTAATAATTTTTTTTCAATCT

>scaffold177.1.129085

CCAATTTTTGAGACACTCTGTATAATTGCTTAAATACAGATTGTGATTATAATTGCTCTGCTCTTCCCTTTCCTTTCTTCCCCTCCTTTGCGAATTTTTC**[G/A]**TTGGTGAAGCATTTTCACGAAATTGTGGAAATTTTGATTTGGTAAAATGATATTTTGTATATTATTGCTTATGTTCTGATTCCATTTTTTTTATTTTTCT

>scaffold177.1.152532

TAAAAAATGTTTGAAAAAAGACTTCTCAAATTTTTAAAATTTACTAATGAGTTACAGATTTGAAAACATCTCTTTTATCATATTTTTTCTTGAATTCATC**[G/A]**TCATTCTCCATTTTCTACAAATAAATAATTTAGTTAAAATAATTTACGAAAATGGAGTTGACTAAATTTTCACTGATGAAAAAAAAAAATTCCAAATCAA

>scaffold177.1.161390

AGATACACATTCAAACGTGTATCATTTTTACTAATTGATGGACTGTATTTGTATATTTATTTTAACAGACAATTTACATTTTACATTTACAAAATATCTC**[G/A]**TTGATTATTAATTCTATTAAATTGTATAAAATGAATATATAAATTAAATATAAATCTGAATGGAACATAATAATATTAATAATCAAACTAAAAATCTATA

>scaffold177.1.177236

ATTTTCAAATAAATTTTTCTTAATTCTAAGAGTTTTGTCTTTTATTATTATTATTAACATGATATATTCAATATTTTTAAAAAATGTACGTACAAAATTT**[G/A]**TTAATTTAATATCTATTTAAGAGAAAGTTTAAAAAGTTATAATCGAAACTTCATTCAATATTATTTTAATCTTGATAAGAGTTAAATCATTCGTAATCAT

>scaffold177.1.180644

AATAATATTTTTTATTCCATTTTAAAAAATTCTTCTTTTCAACTAAGAAGTTAAAAGTGTTTGTGATCTGTATTTTTAAATATTTGAATTTAAAAAGATT**[G/C]**AAAGTTAGAGGGAACATGGTGATTCTTAACATTTGATTAGAAAAATATGGTGAATGTGATATATTTGTGAATATTAGTTCTATAATTCATTCATAACTCA

>scaffold177.1.222046

CTTTTGTAATTTCGGTTTTGGATTATAACAGTAGTGGGTTATACAGTAGTAGAGATTTTAAATAAGAAAAGTTTTGAATCGATATAAAAGATATAAGACT**[G/A]**TTTATATTTTCGGATAAATAAATATGTATGATGCATTTCAATTTTATATTATTACAGAATGTCGATATAATGTCATAGAATATCCTTTGAAGGGTCGATT

>scaffold177.1.125388

TCCTTCAATTCCAAAATTTCATAAATATTTTCAATAAACGTACGATTCGTAAAGTTTCAATTCTAAAATATCATCAATCTGCAGATAATAGCAATTCATC**[T/G]**AATTTCTATCATTATACTCCTTCATCGTCAATTTCAAAACTTCAAAAATATCTTCAATAAACGTATATTCGTTACTCATTATACTCGTTCATTTTCGATT

>scaffold178.1.1342639

TACGTACGATGTTGCAACAGGCAGGAGACAAGTGGTATCCAAAGGATTGAATATTGATATTGGGAATAAAAAAAGGCTCGATGAAAAATTAATAAAAATT**[A/G]**TTAAGATTTCACGATTTGCGGATTTTCCTTTTCTTTTTTTCTCTCCTTTATTGCGATAATATAAGATTATTGTTATTCTCGAACGATGAATAACGTTTTA

>scaffold178.1.652746

ACTAAAAACGAAAAGGATTAGGATTACTGTACCGTATTGTGAGATATGGTAAATAATAATAAACATGAATTGCTATCGTAGACAGGATTCATCGTTAGTT**[A/G]**CCAACATATTTTATATCTCAGAAATTAAACCAAAAACAGTTTGCTCTAACAATAACGAATACAAGTTGGTTAAGTTTCATTAAGTTTCAAAGTAAATCCA

>scaffold178.1.991262

TGCTTCTTATCAAAAAATAATTGATACAACGACGATTATTTTTAAGTAGAATATTTTTTTAAAAAGAATAAATCGATTTTAAATGCGAATAGAAAAAATC**[A/G]**TGCATAATCCATAAAACGAGAATCGAAAAGTATATTCGAATCAATCTAATTTGCCTAGAATCATAGATTTAATTACTAATCCCTCTGTGTTTCTTATCAA

>scaffold178.1.245994

CGTTTCACTTCTTCTTTTTTTTTTTTGTGCAAATCTTTATTGCGTTGTATTGTAATTTTTTCGTCGAACAAAAGTTTCGTCGATATAATAATAAATGTAA**[C/T]**GAAAGACGCATAAAATGACGTGGAAGAATTACAAAAAGTGAGATTTGTCGATAATAATCTTCTAATCCCTAAAGAGAAAGAATTATATATGAATTAAATC

>scaffold178.1.705990

CGTGAAATCATTTCAAGTTAGCTAGTAAGAAAACGCATCGAACGTGTGGATTCGAATCGTGTGAAATTACTGGATTCGACCGAGCAAAAAAGGCTTGGAA**[C/T]**GCAAAGGGTTAATTATTATTATACGATATGGAGGATGAAAATGATTGATTTTAATCAATAACGAGGGTAAAAAGTATTTCTATGATGGCGAAAGCTTCGT

>scaffold178.1.793876

TTCTCTCTTTTTTCTTCTTTTTGTTAAAATTGTTAGATTCTCCTCGAAAATGATTTTTAAGCGATTCAATCGAGTAATTTTGAATTAAAAATTATTGTAA**[C/T]**GAATTTAGCTTTCGAAATATTGGAATAATTTTGGAAATTTCATAACGATTAATTCAATGTGCTTTAAATTTGCAAAATTTTCCGATTTTTATTCAACACT

>scaffold178.1.1174863

GAGTTTATCATGTATAAAATAGAAAAATAATTTATTTTTTTTCCCCGTTCCATTTCAGTTTGTGTAAAAAACAAATTTAAAAAAAAAATTTTTTTTTTAC**[G/A]**TATTAATCGTAAATAAAAATCAATTAATTCCATTTATTTTGGAGGCTTGTTTTTTCGAGGATTAAAAGTTATGGATAATTAAGCTTCTGTAAACTTCTGT

>scaffold178.1.1225047

CGAGTGAATCGTTGTAAATTTTTGGAAATAAGAAGATTTTGATATTTTGATATACGAGAAATAAAAAAAATAAAAAATTATACGCTCTTTGATTCGATTC**[G/A]**TCGCTACGATACTGTAAATACGTATAAAAAAAGTACGTGTCATATGGTTTTGATTAAAATTATTGGTAAAAAAAAAGTGACATTTTTCTCGTTGTATAAA

>scaffold178.1.1577373

ATTATAATATTTGTTATGGAGTATTTAGTGTGTATTTAGAGGTTAGGTTAAATTATAAGATCCGAAAAAAAGGATTCTTCGTTGAGTGAAATTTTATATC**[G/A]**TAATTGATGAAACATATTGAACGTATATCTTTCTCGATATAAGCGGACAATTTATTCGTTTTATCCTTCGGTTTTCCTCATTTCGTAAAAATTATATTCG

>scaffold178.1.1614807

TAAGTTAGGTTCACAACTTGAGAATTACTTGACCCAAATTTAACTCTTAAATGAAATCTGAAGTCTGTCTTATTTTCTTCATTGGACACCTGAATCCCTT**[G/C]**TTAAAAGCTCTAAAGGTTTACTTAATAATTAAATTTGATTAGATTCACAAAACTTGAGAATCAAAACTTGACCCAAATTTAACTCTTAAACTGAAATCTA

>scaffold178.1.1742545

AATTACTCGAATTTTGGACTAATCCTTGAATTAAAATAACAAGAGAGATTTAACGGAAGATATACAGCTGTTGACCTAATGTGATTGTTGAACATACTTT**[G/A]**CATACACTTCAAGAATACTTGGAAATAAAATAGCATTAAAAATAGAAATTAAGGAATTTTTATTTTAATTTTTCTACTGAATCCAATATTTCAATTCTAA

>scaffold178.1.697338

TGTCTCGGTAATAGATTAAAATCACGGCATACAACCGTATTCACACCGTATTCCGCTTCTTTTGCGAATTGTCGCGTTCGCGTGAAAGATTGTGAAAAAC**[G/A]**CGAATTCGAAGCTGGAGAAACGCCTCCGTTTTCCCTTTTTCTCGGCACACAATTTTTCACGGCGATTCGAGTGGATTCACAATCACGTAACTAGCATACA

>scaffold178.1.2002392

AGAATTATTATCACGCATGAATATGGAATACCCGATCTAACCTAATTCCAACCTTTTAGAATTTAGAAAGTCGGACGTAGACGTCTGCTCGTACGGATAA**[T/C]**AAAGATTTCCAGATTATGTTTTCGTGAGAGAAGAATTCAGAGATTGGGATATAAATGAAGAATAAGATCTTATTATCAGTTCACATTTCGTATTTTGCCT

>scaffold178.1.283121

AATTTATCAATTTTATTTTCCACTACTTTTGGGGAAAATCGTAAAAAAAAAAATTTCTTCTTCAAAAGATTGATAACTCGTTTCGTGAATTTTCCTAAAA**[T/C]**GGATTTTTCGTAAGCAACAATTACGTTGACATTCCTTCGAACGATCAAAGAGAATAGTCGATTTAGCGTCGACGTAAAATTATAGCAACTCGCACGCTAG

>scaffold178.1.495966

AACAATCTACCGGTCGAAATAATTTAAATTTGTTAAATCATGGCAATTGGTTCCTATGGTGCAACGTCATCGGGGCAATGAGACAAGATTTTTCCTGCGG**[T/C]**GGATAATTGGAAAATAACCCTCTATTTCTCAATTATTCCGTATCAACGGTGATCAGTGGCGAGCTGCATTAGAAATAACGAGATTATTATCATTAACGGA

>scaffold178.1.587163

TCCAACTTTTCATCAAACAAAACCGTGAAAGATTACACACAGTATAATTGATATTTGACGAGAATTCTCGTGTTGGAGAAGGGGTGCGAGAAAAAGAAAA**[T/C]**GGCGAAACTTATATTTGTATATAAATACATTATATCGAGTTGATGAATTTTCTTGCACGAATATTAATAAATATTCTTCGTTTGTTCGAATTATTTTTCA

>scaffold178.1.831951

ACGGGATTCACACACAGGCGTTACATGCTGAAGGGATTATATCACTGCCTAGTCAGACGAGATGCTACTGCGGAGAAGAATGAAGAGACAGAGGTGTAAG**[T/A]**CCATTGTGGATGGAGAGGAAAAAAACTAGGGAGGAAAAAGAGGAGATGGATTGTAACAACGAACGATTGGAATCTGAATGGCGGAGAAATTACGCGAATT

>scaffold179.1.250223

CTCTGATTTCCGCATTCTTCGCACATCGCATGTAGGAAGGCGTGAAAAAAATTCAAAAAAAAAAAACAAATAAATAAATAAAAAGAAGAAGGAAAAAACC**[A/G]**TTCCATTGTTCCGTTGTTTAATTTAAACGTTTCACAACTCGTACGATTTTTAAAATACGTTTTATCGGGGATATTTTTTTTTCAGTTCGATTTCGACTAT

>scaffold179.1.323586

TGATTCTAAATAAGATTCGAAACATATGAAAAAAAATAAATCAAGTCACGTGATAGAAAGAGATCGTACTGCGAGTTTTATCCGTGATAGATAGAAGATT**[G/C]**TAAAATATATACGTAAGAAATTCAAACCGTTACGATATGCAATGTAATATTCCTTGAAACCACGTCGAATCGTGCAACTTTTGTCCATTGTCCCTTTCCA

>scaffold179.1.343096

CACTTTTAATTTATCCCGTTTTAGCAGAGGAATTCTATTTCCTTTGGCATATTAAATGAAACTCTACAAAACTTTATCAATTCGATTCATCGTGATGGAA**[G/C]**TTCCAATTCTGAAAAGAAAATCGTCACAAAATTGTATAAATTAAAGGGGAAACTTTAAGAAAATTCATCTTTAAAATCTACGAAATCAAATTGAACTTTA

>scaffold179.1.297621

CGATCGATTTTCCATCGAATCGGAAATGTGGAGATTTGATGCGTGGATGCGAATGAAAAAGTATATCTCTTTCTAAAAAGATGTTTGTCACAGTTTGATG**[T/C]**AGAATTCTCTCATCTTTAATGGCCGAATGACGTGTATATATATAAAATCGATGAACAGTTACGATTCCCTATTTTAATTCAACTTCTTTTGCCGCACCAT

>scaffold180.1.225911

TCCAACAAATTATCCAGTAATCGATAAAAGTTATATCTTTGTAAAACTGTATCGTGAAATATTTCCTACAAAAAAGAAACAAAAGATTACGGATAAATAA**[C/T]**GTTTCTGTAAAAAAGTGAACACGAACAATGATTTAATACTAATCGATATTTGTGAATTATTTACAGGAAATTACCTCGACTTCGAAAGAAACCCGTTCAA

>scaffold180.1.66657

TTTTCCAACTCGAGCAATACTTAAAAGAATCTCGATCCTTCGTTTAAAACCTGAAAAGACTACTTCAAATTACGTAAAATAATAGGTGCTCGTTAATAGA**[T/C]**GTAGAATTTTAAATTGAAAGGATTTGAAGTGATCGATAAGAAAAATATTTCGAATAAATTTCTCGCTAATAAAAAGATTTCGACGTTGTCCAAATTCAAC

>scaffold180.1.87690

TTTATTAATTTATTTATTTATTTCTTTAATGAAATTAATTCTTACGAAACAAGGACGGAAGATCGAAATAGAAACGCTGATTGCAATTTTAATTTTGAAA**[T/C]**AATATTCTTCATTTACGAATTAAACAATATAAAATAATCGAATGATAAGCTCATGAATTGTGCGAAGAATTATTTACATACTTCTCGATTTGAACGATTC

>scaffold181.1.1204568

AAAGTCAATCGAATACTCAAGATTATTTGTCCCATTATTAGCACTTCTATATCCAAATTTCCTGCTATAAAGATTTTGATGTAGAAATATTCGTAATATC**[A/G]**TTATTAAATTACTTTACTTGTTAAACCACCACTCTCTCAATAAAAGCACCACGTATTAAAATTGCTTGCACAATTATTCCAAAATTCTCTAACATTCATT

>scaffold181.1.1445690

ATGTTTGTGTTTTTTGGTATTTGCATATGTAATTTATCTTTTTTCATTTTTTTTTTATTTATGTGCACCTATGTGCTATCAATAGTTTTTCGAAAGAATC**[A/G]**TGTCGTGTTATATTCCTTTCCTGACAATATATGAAGAATAATAAATGCTTATCCAATTGCCTGTTCGAACTTTGGTTGTTTCTCGAGATATACTATTATA

>scaffold181.1.1512310

TTATTCTATTTATATTCAATTCTTAATTGTTTCTGAAATATTTTTCTTTTGCATTTTTCTTTTCTGTTACATGAAAAAAACAAAAACTTAATATTAATTT**[A/G]**TTTTTTTTTAATTTTTTAATTAATTTTTACAAATATTATCTGTATCATATAAATGTATTCTATTATTAGAGAAGATATTTTTGATAATAAAATAGGGAAT

>scaffold181.1.1639688

ATTTAAAATTTCTTTCTATTTTTATTTTTTCCAATTCAAAGATTATTATTATTATACATTCTATAAAATTTTATAAAATGTATAAAATTTCCTCCAATTC**[A/G]**TCACCATTCTTACTCAAAATATTACTACTATTATTTAAGAATTTTTTTTACACAAGAATATAATATTTATTTGTTATATATAATTCGTTGTATTTATCCT

>scaffold181.1.705329

TAATTTGAAATCAAATATCCGTGAAATAATACGGTAGGAATTTTATTATCCAAACACCTATTATTCGAATATTATTTATATTTTATTATTGGGATTGGAT**[A/G]**CCATCTTTATTTACAACAATATGAAAACACACGTTTTTCGATATTATTGCTGTGATAAAAATTTGAAATTATTTTTAATTAGAAATTTAATTAAAAATTA

>scaffold181.1.710248

CTTTTCGTGTCTTTTTAAGAGAGATCCAACTATATTCTCTGTTCTGGTAATATTAAAATACTGTAACTTTCTAACGTATTACAAAAAAGATTTCTCTTTC**[A/G]**TTTAAGATCCTTCAATTTGCATCAGAATAAGTTATTTATAAATTATCATATACTTACCTATGACATAACAAAATAATGAGCATTTGATAATAAATTATTC

>scaffold181.1.908941

AATCCAAAAAGCAACTAATGGCGCACGCGTATCGACGTATCCATCCGGCTATCCGTTCATCTTTATCACGCAGGGCGAAATTTATTGCAAATAATAATTT**[A/C]**GAAATAATTATTTCAAATAATTCCAACTTAATTTATTGAATATTCGCGAAACTGTTTTATTTTTAATTAACATTCAGTCGATGTATATTTGAGAAAATAA

>scaffold181.1.1103487

ATGATCGCCATAAAAATGTCATCCTTGGAATCGTGTTGCGCTTCGGCCAGACTGAAGTATGTCAAACATATACAATTGTAAGTGTCGATTTCTCAAAAGG**[C/T]**AGTTTTCTCAGACAATTTCTTTGTTTAAGCTTTATGAAGCGATTTATAGTAAAAAAAAAAAAAAGAAGAAAGGTTTTCTTGGAGAAACTTCTTCGAATAA

>scaffold181.1.1123326

TAGTGATGGGATTCAGTTATAAGAATGTGCTTATCTTCTATTCAATATGCTTGTAAATCTAAATTAAATTCAACTCAAGAATATTATTAGTAAGGTATAA**[C/T]**GAATATATGTAATAATTGAATTATGAAAAGCAAAAAAATATATTTTATTGAATTCTTATAACAAATTTAAAAAAATTATATAAAATTATGAATAGTAAAC

>scaffold181.1.1153861

AATATTATTTTTTTCTTTTTTATTTGATTGCGGTTTTTCAGATTGAAACAAAGATTACCACGCTTCGAGGATTGTTTTCAAATTCCTCCGATCGAATTTA**[C/T]**GTCTGCGCATACATTTTATTCGTGAGAACCGCTTAACAAGATTCGTGGACGATTCGAAACATTGAATTGGAGAGAAAAACTCGGAAAGGAGTGAGGATAT

>scaffold181.1.134630

AAATTGGTGGAATAAGAAAAAAAAAATTAAATAAATAAATATATGTAAATATATGTACAGAAAAGAATAAAAAAGAGAAAGATATACCGTATTATAACGA**[C/T]**GAAGATGAAAAGAGATTATGTGAAAAAAATATGTATATTTAAAAAGAGATAAAAAGTTAAAGACAAACATGGGGAAGAAAAATGAGATGAAAAGAGATAG

>scaffold181.1.1377758

ATTTATTTAATTTCTGTTTTCAAGATTTATATTTGATTTAGATTAGATCAGATTATTAATTTAAAAAAAAAAATCTTATCTAATCTAAAATTATTTTAAA**[C/T]**AGAAGAATTAGAATTTGTTAATAAACAACGTTAATAAACAATCAAATAAAAACGTCAATTAGTTACTATTCAATACTTTAATATATTGATTGGAAAGTAG

>scaffold181.1.1533001

TATTTATTTGTGATTTAATTAATGCCATCACTACTGATACAGATTAACGTTTATGTACGCAATTATTCAATTTAATTAACCGTCACCATTAAATACCGGA**[C/T]**GAATACGTGTGACGAGGATGGTCTCAAGATTATCCCCATCATTTTATGGGAGAACTTTAGGCGACTCATCGTAGCACGGTTTATGGCGTAATGCGATACG

>scaffold181.1.1620253

CTAATATTCTATCTACAATCTTGTATTGTATTTTTAGTGAGTACTTCATGCATTTATCTTTTGAACAGGATTCAAATATATTTTTAATATAATGTTGCAG**[C/T]**AATGAAGAAAATAGATTGAAGATGTATAAATTGAAAAATATTTCTAGGAATAATCGTGCAGGCTTATTATGAATTACGTTATAATTTTTTAGCAAGATAT

>scaffold181.1.283876

TAAAATTCATCGAAAAAATTGCATGTGGTATTAATACGAGAAGCACGCAACATTTATTTCATAATCCTATGCAACCGCGCGAAAAACTTTACATATTATT**[C/G]**ATGTGAAAAAACTATTTTTCTTACGATCATCCAACATTTATCCAAATAAACCAAAATAAATTAAATACTTGAATGAATTATATTATGATGTTTATTATAT

>scaffold181.1.618303

AAACAATATTCATTCGATTTGTATACAATTTTTCGTTTTAACTTTGAATCCGCGAAATACAAAAATAACATATAAAACAATCTTTAAGCGACTGAAATGA**[C/T]**AATGATATAAGGTTCGTATAATTATAATGTTTCGTTTAATTGAATACGATATATACGATATCTTACGAGGAATTAAAATTATTAAAAAAATGTTTTAACA

>scaffold181.1.623982

GACAGCTTTGCTCGAATCGTTCTTTTGCCTATTTTATATTACCTATATTCAAATATTCTCTTTTTTTCAGTTATTTATTAGAAAGTATATAATTTCAATT**[C/G]**AAACTTTTATGATAATTAAAAGAAAAAATTGAACGAAATAAAATTTCTAACAATATCATTGATCACCGTCCGACTCTTTGCGTAGCGATTTGTATAATTC

>scaffold181.1.970413

TGGACCGGTTCGAAACGTGGATGAAAATAGGGCGATATTGAACCACCCTTCTCGATCAAAATGCACCCAAAATCGTGCTTAAAATCGGCGAATGAGCGAA**[C/T]**GAGTACGTGTGAAAATTTTTGCTTTCGAAAGTATAAATTGCTTTTTAGTTTATTTATATTTATGGGATCGAAGAAGAAATTTTCTGCTTTCCTTAAAATA

>scaffold181.1.971294

ATTGAGAAACCCGAGTGAAAAGTTTATTTTCGTAAATTTAAAAAAAAAAGAACACTATTATATTCAACTGATCTATTTAACAAAGCATATCGTTCCTCTT**[C/G]**TACAGAATTAATCAGAACGAATTAATTCTTTCCCTCTCTTTTATCTCTAATTTTCTCTGATCTTCAAATTTCTTTTTACATTTTACGTTTATAATAAATA

>scaffold181.1.991350

TGTAAGAAAAAAAGACACGGTTTGAAGATGCTTATCTTAAACGTTGAGAAAAATTCGCATAAACTGTCCTAAAACGAGAAATAAAGAAGAAATAAATAAA**[C/T]**AAGTGTGTTGTCCCACGTTTCGGATTCGAGTGTGTCGAAACGATTCGCGTGCGGAATCGGCGATTGTGCGTCCATAAGTGGAATTTTAAATTGACAACGA

>scaffold181.1.1089664

TGGAGGATTCGTTATCCCATGATCGATTGGAACGATTGCAAATGTTTGGTCGTTGAGCATATTTTTTTTTTTTACCGTTACGAACAGTTTTTATTCGTAA**[G/T]**AATTTTTATTCCCGTAATAATGATAATAACATTAAACGAGTGAAAAAATTTTGATATACTTTTGTATACCTCGAGTTTCAGAGCAATTCCAGATATACGA

>scaffold181.1.1117814

ATATAAAAATAAGCAAATATAATTTTTCTCATTTCGATAATTGTTACAAATAACTAAATAAATTTACTCACTGACGAAAATATTCATTCAATGACGAAAA**[G/C]**AATTTTAACCAATTTTAATTGAAAAGATAATTATTATTCTGATTTTTATTTTTTTTTTTATTTTGATTATGACAATTATACTCCTTATTTAGAAAAAATG

>scaffold181.1.1370766

ATTTTATTTTGATTTATTCATAAGTATAAAAAAACTAACGCTAATAATTGATATTTATTAATCATTATATTTAAAAATATGTTGCAGTAGAAAATATAAC**[G/A]**TAATAAGTCAATGAACACAATATTCCTACAAATTATATATATCGATGAAGATTAACATGATGATAGCTTGCCTTCATTATAAATATATAAATTATTTCTG

>scaffold181.1.1476523

TGACCAAAAAAATGTTTGAAAAAAAAGATTTTGATATAAATTTCAAGAAAATTGACAAATTAATTTTAAGTTAAATTAATTTTAAGTTTTTTGAAAAACT**[G/A]**CCTAAAGATGATCGGTAGCAAAAGATACAATAATAAAGTCCAACCATTTTGGAATTTTGAATGAATAAAGAAAAAATGATTTGATAAGATATACGAATTT

>scaffold181.1.1501542

AGAATTTTCTCTAAAAGAGAATAAATCGTCTGAAGAAATGGAATAATAACTATCAGATACAATATAAATACGAGGAAAGTTTCGGTATATTTTTTCAGAA**[G/C]**ATAAAATTCTTTTAGAAATGGACAAAATTATAAGAATTGAAATTAAAAATAAAATTACTTTAATAATTTAATAAGTTATTTCAATAATTTATTATTCAAA

>scaffold181.1.191191

AGTTTACATTTTAAGTGAAATAAATATTGCAAAACAAATATAAAAAATATATATATATAAATTAAAAGTTAAATAAATAAAACCACGACAAAAATGACTC**[G/A]**TGACTTCTAAAGTAATATTGTCATGCTATAGCAATAATCTCATAATTCAAAGAGTTAAAATATCAAAATAAGAAAAGAAAACAAAAGGAATGACCTCTCT

>scaffold181.1.294513

TGTGACCTGATTGAATATAAATTAATATATTATTATTATTGTGGGACATTTAAATTGAAAATATATTTTTTTATATACACAAATGTCAATTGATTTTTAT**[G/C]**AAGTTAGAAACGGAATGAAGAAAACAACATGTAGATCAAATGTATGTAGATCAAATTATCGTCTCGTTTTATCTCCTTGATTCATTTAAATTATTACACA

>scaffold181.1.306777

TCGTTTGCAAAATTGAAAAGCGCAGTCATTAGTGATATTAAATAGATAATTATTATAAATAATAAAATAGATTTTGAGATCGATCGATTTTTATTGTTCC**[G/A]**TTTATTGTTAAATTAAATTATTATAATTGAAATAATTGAAAAATTTTCAAAAAGAAATTAATTATACAATTTTAATTAATAATCATATCATTTTATAGGT

>scaffold181.1.313666

GATCTTATTCTCGTTTCGTCTCACTTATTCTCGTGACGTAAACTTCAGTTACTTATAATTTAATAAATTTTAATTTAGATATATTTGTATAACAATTTTT**[G/A]**CATTTGTTTTAATTTTTAATCGATACTTCAAAAATATCTCAGGAATATATTAATATTTGTATAAATAATAATTGTATAATGCTGTTAAAATATCTAAGTC

>scaffold181.1.349489

CAATGTGTTATAGATGCAAGACATCAATTCAATAATTTCAATTCAGAGATTACGAAAGGAATAAATAATTTTTAATTAACTTTGATTATTATTTTTGTTT**[G/A]**TCTAAACAAAAATGTAGAAAGTTCGATGAGACAATATTGCATTCAATTCCACTTTGAATTGTTTAAGCATATAAAAGAAATACAAAATAGACTTTTTATT

>scaffold181.1.510379

ACAAAATGAAGAATAACGCGAGCCATCCTGCTCTTTTTACCGTATAAAATGGAAATCCTGTTGAAACCACGAAATAATATCCTATCTTTTTCCAATAATC**[G/A]**TAAGAGGTGTATATATACATATACGATGTATATCTTTGATCATTGTGATTACTATTTTTTTTTAATTATAAATTGTGCATTAATCAATTGAAGAATTCGT

>scaffold181.1.602528

AGTCATTATAACATTATTTGGAGTGAGATTCTACTTCAAACATATTTATGTTTATTGCACTTTTTTGAAATATCTAATCTAATATTTAATATCTATCTTT**[G/A]**TTTTTAGGTGGTTGGATTAATTTGGGGATATGTTATTCAGCAATTTTCTCAAACAATGTATATATTAGGAGCAGGATTCGTATTGGCAGCTATAATTACA

>scaffold181.1.620286

ATATCACTATAAATTAATATTATATCTATTTTTCTCTCTCTATCATATTGTATTATCATATTTTAATTAATAAACTTTCGTAATAAACGTAATAAATAAT**[G/A]**CTAAGCGACTAACGAGGATAATGCTTGCGAACTGTTGAAACTATACTGTTATAATAACACTGTTTCAGTTTTTATTATTATATTGAATAAAAAACTTAGC

>scaffold181.1.893559

CGAATAACTATTTTCAAGATATATCTAATATCTTTAAATACAGTAATAATTTAATAATTAGAACGTTATCCAAAATCAGTAAATATTTTAGACCAAATTT**[G/A]**TTTGGATTTTGCTTTAGATATTGTCATATTTTTGTGTAAAATGCTGAAACTCTTGCTTAATTCTCCTTAAAAAATTAGAAAATCGAGATTGTTACACTTA

>scaffold181.1.1134790

TTATTATCAATAAATGCTATAATATATTATAATTAATATTGGTGAAATAAATATCTTAAATATTATTTAATTATTTTAAATTGTGATTTAAATAAAATAA**[T/C]**AATTTAAATATAAAATTTTGAATATAAATAAAATATTTTCATTTTAATAAATGCCAAAAAAGTAAAATAGTACAATATAAAATAAATTAATCTTGCTTAA

>scaffold181.1.1265531

TATATAGACATATTTCTATGATTACATTTAATTTATCTAAAATAATCAATTAGATCTGCATTTTTTTTTAAAAATATCTTTCTGAGTGAAGCATAATAAA**[T/C]**GATTATTTTTATTATAATTTCATTAATAATGTTTAAATTTAATGATATGAATATATTATTTGCATTTTTATTATTTAAATACTTATCTCATAACATGCAA

>scaffold181.1.1368906

TCTGATGATTGAAGGTATTTGAGCAATTCTTGGAATTCAGCATCTTCTTTTTTGTAAACGCTGATGATTGCGTTAATTTTCTCTAAGGGAATAAGATCAA**[T/C]**GAATTTTTGCAATTCATTAGCAAGGGCACCACTTCCAATGCTTGGGACTTTGTAGGCCTCCAAAGGATTAGATACAGCCAAGGCGACCAAAATTACCAAA

>scaffold181.1.1550837

AAATTTCGTTTTGCTAATACACCTATAAGAGCATATGCATTAGTATGTAATCATCATTTATTTACTAAATACATCATGACGGTTGCCATCCAAAATGTAA**[T/C]**GGCTGACTTAATATCATTACATTTAATGCATTACTATCTATATCAATTTCAGTCCGTAAAAAATTATTCTATATTTATTTTCGCATTCATTGAATCCGAA

>scaffold181.1.306094

ACGAAGTATCGTGTCAAAGAGGTCGCTTAGATCTATGAATACAACACAGATCGAATTATTCATGTGAAATATATTTATAAAATTAAATGAAGTTCAGTAA**[T/C]**GCATTGCTAAAGATGAGTGAAAATATTTATGATGAAATTTGTGAAGTTTTACGAAGATTTAAAATAATCGATGAATTGTTTAATTTTATAGTCAGTTGTG

>scaffold181.1.95761

AATTAATTAATATAAAAAAATTGTAAAATAATATATAAAAATTAACTATGATTGTAATTAAAATTTTCTAAGAATAATTTTGAGAAGATTCTTATTTATG**[T/C]**AAGATATGTATCATTAGAAAAATAAAAATTAAATATAGAGTAATTTAATAATTTAACAATAATTTTATAATCCAAATATTAGAAGTACTTTGTTATAATA

>scaffold183.1.134731

CTGACCTATTCCAAACTGCAGACAAGCGACAGACGCGATTCCTGGAAATAGTACGCATTTCCACGAAAATATGTAGGTCACTGACCCATTCATAATTTCT**[A/G]**CCCTGTTTGAAGCGTTTAGAAACTGTGAATCCATATATCTCGGTAATCGATTGAGATATCGGGATAAAACAAAAAACTACTTCAATGGAATGATTCTCTT

>scaffold183.1.122297

TATTCATGTTCTCATCGAGAATAATTCAAAATACCGCTGTTCGGTTGTTCTTTAACGTTTCCACGTTTCCATGTTTATAATTGCGCGTTTTGTCTTCGAA**[C/T]**GATTTAAAAGAGGAAAGCGGTTCATGGAATGGTGAATGGATTCGAGGTTAATTTGAATTGTGCTGAGAATGAAAGAAATTCTATCTTCTTTGGTTTACTT

>scaffold183.1.30824

AATCATATTTGTTGCGTAATGAATCGAAAATAATTTTAGACAGGTATATGTTCACAATAATGTAGCAAAATGCATACAAGCTATCAATCAATGTAAAATA**[C/G]**TTAAATATGTAGGGATACTGCATCGAATTTGGCATCAACACTTGAAACATGAAAAATTTAGTTGTGTAACGTGCGATTGCGGATGGGACGTGATGCGAGA

>scaffold183.1.44707

ATAAAGTTAAAGAGAATGAATTAATATTAGGATTTCAAGTAATGATAGAGTATTTTGACAATAATTGTGAGACGAGTGCAGGAGTTTGATTCTTTGGATG**[C/T]**AATATAAAATAATTGGATTAAAATTATTTTTATATTTATTTAGAATGTTTTTATTCAATGTGTTTATTTTTGCACGATTTTTCAAATATCCAAAACTTTT

>scaffold183.1.43113

TTAATTCAAGTACATAAATGTAAAAAAAAAAACAGAATTGGAAACGAGAAATGATATTTGTTATCAAGTTTAAAAAAAATTATGTCGTAATAATCTTATC**[G/A]**TAATAAAAAATTTGACAGAAATATAAAAATATGAAAAGAGAAAGTTATTTAACGGCCAGATTTGAATGTCTATGCTCATTCCAAGCCTGAATTAATCCAT

>scaffold198.1.133566

CGTACGGATAATACGTTTCGCGTTGTGTTTGTGGAGCTCGAAGCAGCTGCTTACGGATAAATAAAATTTCATCCATGGCAAACTTCATCTTTCGCAATAA**[G/C]**AAGTTTAACGAAGCATCTCGTCACATGGTTTCTTTTATATTCGCTCAAATGGATTGCGATTAAATTTTTTTTAGAAATTCTAAGATAGTTCTTCGGAGAC

>scaffold198.1.117528

GATGTGCTTGAATCCTTTGAACCATCGTAAAGGTTTTCTTTGTTATTTAAGGTGTATTGGTGCTGTTTTAAATTGTTTTCGATGTGTGTATTTAAGGGAA**[T/C]**GATGGATTGGATATTTGTTTTATCCTTTGTTATGCTGCACAGTTTGATATATTTGTAATATATATTTTGTAAATATTTGATTATATTTATTTGATTAGTT

>scaffold200.1.11490

CGAGCATAAACGCTAACACGGTGCCAAATTGGGAAACTTGGCCTGGAGGATCACGAGCACGTACAGTGTGTACATTTTAAATACGTTTCGAGGAGGATAG**[C/T]**AATACAAATTACCAAAGGATACCGATAGAATTAGCCAACAATTTTCAGGAAGAGAAAGAAAGAGAGAGAAAGAGAGGGAGGAAGAGAGAAAGGTAAAGAA

>scaffold200.1.44325

TATATTTGAAAATTTTTCCTGATCGAATTCATTTCCAAAATTTTAATTTAAATCTTACGTTTCTTTTTAAAATTAATTTCCCTCGTGATTGATATTTAAA**[C/T]**GATTAGAAACTCTATATATGATTAGAAATTTATGTAAATACTTTATAAAAGAAGGGATTAATAATGCATTCATTGGAAAGAAGAATGATAAATTCTACAA

>scaffold200.1.67259

TGATTCAACGGTTTATTTTATTAGAAAAAGGAAGAAGAAAAGAAACGACACGACGCGATTTTCTTGGGAGAAAAGTTTTCGATTGAATTATTTAATTTAA**[C/T]**AATTAAATCTTTGTATAATATAAAGTAGGAAAGAGCATTAAAAATGTGATAAATTACGTGACTCGAAATTGATAAATAATTTCAGTTACAGAAACGGTTT

>scaffold200.1.45829

AATTGGTAGTGTGAGCTGATTCTTTGATCTTTTTCAACTTTTTCTTAATCTTGTATCACGCAATGCATATGATGACAGATTTAATTTTAATTATCTTTGC**[G/A]**TCTCTGTAACGAAGAATGCTCGAGAGGAAGCTCAACGAAAGATCAACCAAAAAAAATTGTTCACAGAACGATAAACACGTATTCAAAGAGATTTTAATAA

>scaffold201.1.125742

AATGATTAAAGGAAGGGAAATACTTGGGGATCCAATTATTCTCGATCATCGATTAAAAATATATTTCACAGGAATACTCATCTACTTTTATCATTTTAAA**[C/T]**GAAGAAGATTTAGACACGTATGTGGAAATCGTGCAGAGGATACATTACATGATGCATTAATTAAAACGAGTTTTACTTTGAGCATCGAATTCTTTATCCA

>scaffold201.1.14048

CAGCTTCTCCCTCAGGATATATGAAATGAAAACTGCAATCTTTTTATTCGTGTAAATTTAATAACATTTGCGCAATTGTAATACTTTATATCTTTTGATA**[C/T]**GATACTCACCTTAAACCATTATGTTTCATATTACTTTATTATCACTTTATGATTGAAGAAATAGATTAAAGAAAAATAATTAATAAATATCAATTATTTT

>scaffold201.1.91755

ATGAATATATTAATATGTAAAATAAGTTTTGTCCAGACTCGTCGGTCCACCCTGCGGTATAATTCACATTTGTGCCTTATATAAAGAATTGAACGAAAGA**[C/T]**AATTTCAAAGATCTTAGCAGGATTGTTTTATTAACAATGACAACTAAGATTAAATTTCAGCTCAGATCTTTGAAAAAGTTTGTGTGATCCTCTCACTTCA

>scaffold201.1.96979

TTCCTCTAAGCAATTAAAAAGTCAAAAAATTGCCAAATTATGTTTTCTATCTCTCTCTCTCTCTCTCTCTCTTTTTATTGTTCGATCATTATAAATCTAG**[C/T]**AAAATTCACGTGCTGTTAAAAATAACGAGAAATAAAGAGAAAAAAAGAGAATACGTGGAAGCAAACATTCTAACAGGTCTAAAAACCTATTATATCGATC

>scaffold201.1.65621

TTGAAGATAATGTTTTGTGTCTATATCTTAAACGTTTTAAGATAAATTTGGAATAGATTAAAACAAATATGAAGAAATGAAGTACTTCTGTAAACAAATA**[G/C]**TATTTGGAGACAATATTATATTGTTTAAAGTTTGGTAAATCATGGTAAATCTAGAGGAAAATAATGTTTTGAGATAAAATTGTAAATGTGAAGTAACATA

>scaffold201.1.74862

ATGAAGCGTTTATCTATATCGTAAATTGCTTAATTTATCGCTACATCAAAATATCGCCAAAGACTGCAGAAATTAAATAAACATATTAAAACAGTTTAAC**[G/A]**TTACAAGACTATAATAATATATCATAACCCCTTTTTCGTATAAATATATTAATATTATATACATATCCTTATTTTTATTAACGAATTGCATTATGCATAT

>scaffold201.1.132057

ATAATTCAAAAACGTCGATCAGTAAATCTTCGTTTTAAAAAAAAAACTCCAATGCAAGAATTTGATTCATTTTTAAAATTTCCCTTCGAATCTTTCCAAA**[T/C]**GTTTTTTCCCGAAAATTTCTCAAATGTTTGCAATAATTTTTATAAAGACAGACCTCCGGATATCAACAAATTACATATTTCCATGTGCAATTTTCAAATT

>scaffold205.1.101476

TAAGAGAGACGAAGAGGGATCGTTGAAGCAAGAAATAAACACTGTGACGGATAGCATAATAAATCAAGAAGAGAGATTCACCGAGGCTAGTCAATTAAGC**[A/G]**TGAACTCAACCAGTAAAGGAAATAACAAGGAGGCGATTAAGATTCTTCCAGAAAATTTGAAGGAAATCATAGAATTGGAAACTTTCAAAAAATCGAGCAA

>scaffold205.1.66757

ATCATAGTCAATTATCAAGTTTTCTAATTAAAATTATCCTATGTCATTTTTTAATTATTCCTTTATGCTATACAAAAATCACATTTACATTTTTATATTA**[C/G]**AATTCATTCAATACCATTTTTTAATTATTTCTTTATGCTATATAAAAATCACATTTACATTTTTATATTAAAATTCATTCAATACCATTTCTTTGAAAAC

>scaffold205.1.23444

TATTGTTGTAAATTATATAAATATTAATATATCGTATTATAGTATCAATTACTGTACATTTTTAACACTTTATATGTAAAATTAATATAAATTACATTAC**[G/A]**TTACGTTACATTACATTAAATTACATTAAATTAAATTACATGCACAACAAATATAAAATTTAAATTAATATCGTATATCTTTGATTATCTGGTTTAAGAG

>scaffold206.1.586442

ATTTTACTGTGAATAATTTCAAATTTTTTGTATTTGCCGGAATTTGAGTGATAAAAATGAAGAAATATGTCATGCATTGAGAGAAATTTACAAAGATTAC**[A/G]**TCAAAAAATAAGCATACATTTTCTAAGAATGCTCTAATATTAATAAATTGCATTTTAATTTCTAGATATTTTAATTTTCTTAAATATTTCTATTTTTTTA

>scaffold206.1.607830

ATTATTCTTTACTAAAGAGAAAGATAAAAGATTTTAAAAAATTCTAAAATTCTTTCTTATTTTTGTATTTCATATTATAATGCTTTTATTATTAAAATGA**[T/C]**GAATTTGTTGAGTTTAATAAGAATTGTAAAAATTGTCAATTTTATTTTATGAAAATATTATATCTGAAGATTAATCTTTTTTATATTTGTATTAAAATTA

>scaffold207.1.76207

AATTTTAGTGAAAAATATAATTCATTATTGATTATTTAATAATTGAAATATCAATAATTAACAAAATAATCTTAAGCTACTATTACTCGTTACATAATGC**[A/T]**GTTCATGATAATTAATTTTAAATTACAAATTTTCCTCAGTGATAATTAACGATTAAAGAATTAAGAACGATTAAAAAAATATTAATAATTTCTTATGTAA

>scaffold207.1.18211

GAAAACGTGTTTCCAAAATCCTCAAACACACCTCCTCTGTAGAAAAAGAAGAAGCGAAATGAAAACAAAATCCATATTTAATTTTCGCGAAAGTGATTCG**[C/T]**ACGATACTGCGAGCAGAGTATCGCAAACAACTCTTGAACATGCGATGTTACCAGAGGTGAGTTTAGCTATGAACCAGCACCGAGCTGAAACCTTTATTTC

>scaffold207.1.70646

TTTTTCTTACATTTCGCAATTTTATCAATTCGCTATATTCCACCATTTACTTCTTATATCCCATAAGAGTCTTCAGAACATTTTTCTATGTCTTTTGAGA**[C/T]**GTTTTAAATACGCTTTCTAAAACGAGTTTTATTCCTTTTGCTTGTCAACCATTAGCAATGTGATATTAACCCGATATTTTTCGTTCTATTTTATCGAATC

>scaffold207.1.23858

AACAAAAATGATTGGACACTTTTGTAAATAATAATAATTATTCTCCCTATTATGATCTAAACTTTTAGCCAAACAAAAGATTAATTATCAAATACAATAA**[T/C]**GATTAACAATAATTCAATAAAACTCACGGAATAATACAATAACGCGAGCATAAAAATTTCATTGACGTGTATCGCCATTCCAATTAAAACCCACGCGTAA

>scaffold207.1.14115

CTTCCTTTCGTTTCTAAAAAATGTCTAATTTTATTAAGAATGAAAATTAATAAATTAATAAGTAATAATATTAGTCGTTTTTCTAATCAAAACGAATCAT**[C/G]**ATTTTTAATTAAAACAGTCTAATTAAAAGAATATAAATTCTGAATATAAATTCATTTAGATTTCGAGAATTTCGAATCACAAAAATAACAAAAAATTCAA

>scaffold208.1.193004

TCATCATTTTATGATATGTCCGTAAGATATGTCTATTCGACTTTTCTCGAATGGGAAGTCGAGCTATCTATCCTTTATTATTTAAAAATAATAACCCCTC**[A/G]**TTTTCAAATAAACACAAATCGCAAGAAATAGTTATATGTAACATTTACATATATTCAAAATAATCTTACCATTTTTTAATAAATAAATTAATCAACGTGC

>scaffold208.1.731258

TGGTTCTATATTAAATTTTTAATTGGTCGAGAAATAATAGATGTTTAAAGATGTCGGTTAAAATCTAATTGTAATAAAAATTTCAATAAAAATGAAATCC**[A/G]**TTAAATGTTGATTTTTGTGTATAAATCATTTTTAAATACATATGTATAATTTTATTATTATATTTATTTAGATTATATATGTATATGGTTTTTCAAAAAT

>scaffold208.1.289116

TGCCAAATAATTCACTTGTATCCAATTACATGAAATTAATCAAATTCTCTCAAATAATTAACAATATCGATTTTTTCGAAATCGGAAAAATTCTGATGTT**[C/G]**TTTTTTCTTACTTTTACGATCACCTCCTGCTTGATTATACCACCACTTACCAGATACCCCGTGTATACTATAATCTACATTCTGAAAGATTGAAAGGTCT

>scaffold208.1.778704

AAAAGGAAAAAAAAAAATGTAGTTTTTAGTTTTATATTTATATTATAATATATATTTCTGTTTTCGTTTTCTAGGTCACGTTATTCAAACTTATTATTGA**[C/T]**GTAATTTTTATATTTTTATATTTATATCATGTAAATATTATATTTAAAAATCCTTTTGTTTTTTAACATTTTTTATCTTTAAAAATTATAAATATGAAAT

>scaffold208.1.98065

AGAAAAAAGTAAAAATACAAATAAAAAAATATTAATCTTAGAATTTGGATTCTGGAGAATCGATTCTAAAAATAATCCTAATTTAATTGTACAATGTTTT**[C/G]**ATCACTATTTTTTATACATTTCTATCGAGTGAAATGAATTAAACTTTTATTATCACTGGCAATTATTAAGATTTCACAATATGCATTCGAATTTTCAATA

>scaffold208.1.98265

ATTTATTCAATATTAACTCAATATTTCTGTAAAATTTTATTTACTTTTTTTTATTGCACGTCAAAAATCTCTATTAATCGAAATATAAATATATATCTTA**[C/T]**GTTATTTTTACTTCTATAATTTGAATTAAAAATAACTCAATACATTAATTTAATACAATATTGAGTCTGTAAGTAAAATTAAAATAATATACGATATAAA

>scaffold208.1.423269

ATTTAAGTTTTATTTTAAAATATTTAATATATTATTGTGATATATTTTACATTATTAATTAAAATTATAAGTTCATTGCATTTATGATTCATTTATTATC**[G/A]**TAGAATTTTATTTTTTTTTCTTTTGTAGAATTATATATAAAATATATTATATGTAAAATACACGAAAAATAAAAATATTGAATGAAATTTTTGCAATATT

>scaffold208.1.613837

AAATGATTTTATTTAAAAAAATAATATTGATCTTTGAATGTCCTCGACATGTTTATTTATAAAAATTCAAAAAAACAATAACTACTGTATATATCGTTTA**[G/C]**TTGTTACAATAATAATACAAAATTAGGAGTTTTTTTTAATAATTATTATAGTAATTGTTAAAAAAGTCATTGTTACATATATTTTTTATCTGTAGAATCA

>scaffold208.1.617767

AAAAAAAATCACGGATAAATCATTACTACCGAAACAATTAATCGACTTATTGTCGATTTATCGCCGAATCAAGATTGGCCATGTCTCCAACGAATGTATC**[G/A]**TTCATTTCTATAAAAATATAATAAAATATATTTTTTTTATTTTTATAAAATTCGCATGTATTTTTGAATTTTATTTAAAGATTATCATAAAGATGTATAG

>scaffold208.1.706073

AAAAAAATATTATTCGATAATATAATCAAAAGAATTAAAAATTAAAAATTATCTTTTCTGCGCGAAATATTTAAAATATTTATACATTTTCGATTCTTTC**[G/A]**TTCAATTCTATAATAATGAATATTTTGTTTTTTCGAAAGCAATTTTTTAACCAATTACCGTTTTTTTTATCAATTTATCAATTTGCCAATCACTACTCAC

>scaffold208.1.7237

ATACATATCATACGGTTAATAATATCTATTTCTAAAAGGAAAATAGAAAACTTTTTCCTGTTCATAAATTTTTCTTCCCATCGTGAGATATCTGAAAATC**[G/A]**TGACAAGTATTACAATGAAATTATTTTGTTTCCAAAGAAGAAAAAAAGAAGTAGTTTTCGAAACTCATTACGTTTCGAATTATAATAAAGAAATAAAATG

>scaffold208.1.243671

AAATAATATTTCAAAAAATAATTATATATTTCAATTGTGATAAAATGTATTTTTTAATCGATTTTGCTTCTTATATTCAGAATAAATATTAAACTATAAG**[T/C]**AATTATATTAGTAATAATTTATAACAATAATAATTGTAGCAAATACTTAATAATATAATAATATTTGTAACAAACTAAATATAATGTAAACATTAAACTT

>scaffold208.1.706248

TTATCAATTTGCCAATCACTACTCACTACATTCAATCAAAATATTTTTTATACAATTTTATATTTCCATAACAAAATAATAAATATCATTGTTGGGGATG**[T/C]**AATTTAAATTTTGAAAATTGAATGTTGCTTTTGTAATTTCAGAATTCAAAACAGTTTTTTAAAAATTACACGATTAATTTCAACTGTAATTTCTTTTTTA

>scaffold208.1.784016

CATTTTGAAATAATATGTTCCATAATTTTAAACAATAAAAAGCCATAATTTTAAGCAATTTTTTTTTTTATATAGTTGGCTTTAGTTTTCGAATTATTGG**[T/C]**AAAAAATTATTCACAAAAAACTTTTTACATATTATACTGCATTATGCCTCTTTCGAATTCCCAGAAAATGCAAAAATAAAAAGAAATCTTGTTCGAAATT

>scaffold209.1.185863

ATGTACCAAGCATTTAAAGATATTAATATTTTTTCAAAATGAGAACTTGAATGATGTTTTCAAAAATAAAATATTCTAGAATAATAATTTTCAAAAAAAC**[A/G]**TTTGTATAAGTTTAAGTTCTGCTATCCTGAAACATTTGATTTCGTTGCAAATTATAAAAAATAACAATCTTATTTAATTTTAAAAATTTTAAGAATTTCG

>scaffold209.1.212226

GATTTGTTTAACTATCGTAACGCGAAATGGTGAAAAATATTTATCGTGTTTTTTCATAAAGTAAAGTGCATATAAGAAAAAAGTTATTTTGATATATACC**[G/A]**CTACAAATGAAATATTTTTAAAATTTCACCTTTTTTGAAATTAATTGAGATAACAAAAGTCCTCTTAATATATAAACTTTCATATTCCGTAGTTTTTATA

>scaffold209.1.191169

AGAAATGAAACGAGTGGATTATCAATTATTGTTAATAGGATAATAGCACGTTTTAATTGTATTCAAATGTATTTATCATATTTATAAGGATTTCATTTTG**[T/C]**AGATTATAATTATAAGATAACTGCAAGTTTCGTTGTGAAAGCTCTAATTTTCTCAACAGTATCATTTCTATACGAGCTGTGAAGGTATATATATGTACGT

>scaffold212.1.30854

AGAAGAAAATCAAAGAAGGGAAATTATTCAATTGCAAGAAATGGCAAATTGAAAATCCTCAGTGTTAAATCTTGAAAAAATATTGTTTTACATTATTTTG**[C/T]**ATTATATTAGATTTGATCTAATAATTTCGTGAACGTGTTTCTTTATGGGGAATGTTCTCGCATTCGTTCGCGAAATTTAAGAACTGATTTCGTTAGCATT

>scaffold214.1.58003

GTACACACCGTGTTTCATTGGGTAATATGATTTACGAGAAATTTTTCGAAATGATAAGAAACGGATAAGAGAAAGATTTGATTTACGAGCAACCTTTTTA**[T/C]**GAAGGATAAAAAAAAGGGTGTATATTAAAATGATAAACTTCGTCTTTCACAATAAACGTCCAAATGTACTGTACTCTTTCTCTCTTTCTCTTCCTCCGTG

>scaffold217.1.788007

ATTTTAATCGTATAATTTAAAATTTATTAAAATATAATAAAGAATATTCTTTTTATCCGCAGTATTTTAAAAAGATTTTACGGTGCCTTATAAATAATTT**[A/G]**TTATGTATGAACAATTCAAATTAGAGTTTAACAACAAGTAAAATAATATAAATCCAAAATGGCGATTCAAATTAATCGAGGATAATATATATATATATAT

>scaffold217.1.302582

GAAAAACTCGAACAATTGAAGGCAATTCTATATTTTTGGAAATTTGCAAATCTTAACAAAATTCCACGAAAGCGATAAATACATGAAATAGAAAAACGGA**[C/T]**GGATGGATTTCTAAAAGCGAGAGATTCCGCGATTCCACGGAGCATAGGAACCGAACGGTGCCTAGGCTCATGCATTGTCATCCGGAAAAATGTAATTCAA

>scaffold217.1.551325

GGAGAAGAGAAAAAGATATGTACGCGTCTCTTAAAATAAGAAAGCTGATAATTAGAGAGTGAATAATTAAGAAGAAAGTGTCGATGAGATTTTTGTTCAA**[C/T]**GCTTGGGGAAAAAAGGAAAAAATGGTAAAAGTGTGAATATTCGTCGTCACGAGATCATCGTAAAATTTAGTCGAATCTCCGCCTGATGATTCTTCGTTTA

>scaffold217.1.635317

CAACTAAACTAACAAAGAACTTTCAAATAAACGTGCGAAATTTATTTATGCGCCTTTTAATCAAAGTGATTAATCTCGCGAAACAAAAAATCTAAAAGTA**[C/G]**TAAATATATGAACTAGCAATTGAATTCCAAATCTCATTTATCGAACATTCAAAAAGTAACGTTCATATCAACGAATCAATTCCATATTTGTCTAACAGGA

>scaffold217.1.655130

TTAATTCTTTATTATTTCTCTTTACACTATCATATATTCTCGTTTAAATATTTTATAATTAATCCACTGCATTCTTTTCTTTGATCTTACGAGTTTTTAA**[C/T]**GGAGAATTTTTCTTATAAATTAATTCGAAAATTTTTGAAGATCAGTGCCATCTAGCGTTTGGATCACCGAAGCTCCAACTTGTTACTAGCATAGCAGAAT

>scaffold217.1.177160

CCAGAAACCGGAACAGGGATGGATAAACTCGAACGAAAACTAAAAACTTCGATTCCATCAAAGGAAACGTACAAAACGCAAAGATATTTCGTAAACAAAC**[G/A]**TTAACAATCTTAATTTAGAGACTTTATTCAAGAAATGACGAGCCTTAAAAAAGAGAAGAAAAATGATTCGCGTGATCATCAGAGAATAATATCACTTTTT

>scaffold217.1.549954

CAACATTTTATAGTAAAATATACGAGATCCAAGACAAGAATTTAAATTTGAATAAAAATTCAATTATTTTCTTTCATTTCACTCGTTCGAAATCTTACCA**[G/T]**AAGTGTATCAAAATTCAATAAAAGAAGATATTTCTATATCCTATTAAAACGTACGATCCCTTGTTTACACTATTCTTTCACGTCTTTAAAATCAGTCTAA

>scaffold217.1.610809

GGAAAAAATGAATTCTATTTATACTTATGAAACAGTTGACAAATTAGTATGTATATTGCACCAAAAAAATACAGGCGTGAAAATTGTTTTTATTTTCCTT**[G/C]**ATTCTTGAATTCAACTTGTAAAAAACAATGGAATTATGAAATAATTAAAAAAATTCTATCAAACAAATTATACAGTTATAATAAGATAAGATTAATTTCA

>scaffold217.1.623778

ATTGTAAAATACAGAGATTTAAACGAAGATTGAAAATTAAAATATTGAAAATTTATATTCTTTTTTTTTTTATTTATTTATTCATCTACCAAGAATAATT**[G/A]**TTTATTTCAATCCTTCGTATTTATTGCAAATGTGGGCGGAATACGTTTCCAGAATTCAGATTGATTAGCGATAGATGAAGATCTTGTTAAATCTTAACAA

>scaffold217.1.655017

TAATTAAAACATACTAGCTAGTCGAATGTTTATAAAAAAAAATAAAAAATGTGTTGTGTACATTTATTTATACATATAAACATTCCTCGAATACATATGT**[G/A]**CTTCCAAGAATATTAATTCTTTATTATTTCTCTTTACACTATCATATATTCTCGTTTAAATATTTTATAATTAATCCACTGCATTCTTTTCTTTGATCTT

>scaffold217.1.440887

AATAATCATACAAAAGTCAAGTTGCTTAATCGAAAATAATTAATAAAAAATGAGTAGTTGAGTCGAACAGACATTAGTGGGAAAGATGATTCTCGTTAGA**[T/C]**GAACTTTTGAAAATTTCAGTACGGCCTGTACAGCAGTTTCATGGGATGCTTCGTCTACTTGGTGTTCGGTAGCTGCAAAGATATCACCGTGGGACCTACG

>scaffold217.1.673247

TAGAGATGGCACTATAAAAAAGAATATTCAATATGAATTATATTATTTACAAAGTAATAAATAATAATATAAACAAAAAAAAATAATATTTTTATTTAGA**[T/C]**GATAGATGAAACATGCATATAGACAAAAAATGCAAATAGTTAGAGAAGGACAGAGATAGGATAAAAATATTGAAATCAATGCAACAAATGAAACACGCAA

>scaffold234.1.1465057

TTTCTCTTATTTAAGACAGAATACTATATACTTGGAACAGATAAAATTAATATAATCTAACTTAACTTAATCAGAATAGTTAATGGTTAAATATTGATTT**[A/G]**TTTTGCTATTTTTTCGAAGTTATATATACTTTCACTTTTATCTTTTGTTTCTGCTTATTGTAACTTATCTTCCCAAAATAATTTTTAATTAACATTAATT

>scaffold234.1.1468476

ATCCTGAACTTTGCTATAAGAGATAGCTATAAGAGATAACTATAAGCGTTTATTTAATAAATTTCATTAAAATTTAACGTAATAATAATGACAATTTTTT**[A/G]**CAAATATCTCGGAAATTAATAAAAAAGTGTTCAAAATATTAATTTCTATATTTATTTAAAAATCATTGAAATTGATGAAGAAATGGTTAATCTTAAATTC

>scaffold234.1.1613850

TTCGTATAAATGAACGGAACGAGCGAAATCAAGAAAAAATTAATAACAAATCTTAGAGCATCTATTTTTCTAGACAATTAATTAAACATTTAACATATTT**[A/G]**CATAGGCTCTGTAATTTAATTTAACCTATAATAAATCATTCTGATTTTATTATATAAAGTATAAAGTAATGTTATCAAATCTTAATAAACCAAAAATTAT

>scaffold234.1.1236484

ATAAATTGCTTGATATAGTAACAATTTTTATATAGTTTTCAATAATTTTCGAAATTTTTTCTTCAAAATATTCATTGAAGAAAAGAAATACGAATATACA**[C/T]**GAAACATATTATTAATAGAAATTACGATATGTCTATTTTTTAGATGATAACTAAATTGAATTTACAATATACTATTTTTCTGACATAATTCTTTGTCAGT

>scaffold234.1.1496036

TATGAATCATTAAAAAATTATTGTGTTATTATATAATTGGATACATAATCTTAGAAAAATCATATATATGTATATGAGATATGAGCGATTATCTTTAATT**[C/A]**TTAGTATCAACAATTCAACAATGTAATTAATGGATTTAAGAAGAGTAGAATATTCATATAACAGAAATATATTTAAGAATTTTATCCTAAGTTATATGAC

>scaffold234.1.1541848

ATATCTATATGCTTAAAATAAATAACAAAAATAATTTTACGAGATACTATAAATACATTATAAATATCTTCATTATATAATTAATTAAATATCTTACTTT**[C/A]**GATACACCAGTTTCATGTTTTGTATCAGATGCATCCCATTCTATCACATAAAATGTTTTTTATTGTAATATTATAATATTAATCGCAATATAATAACGTT

>scaffold234.1.1614061

TTTATTTCTAATATAAAATGTGCTGATATTAATGGAGAATGTCTCTGCTCTCTCATTCAAAAATATTTAATAATCTATTATTAATTTAATTTATCGTTTA**[C/T]**GATAATGCAAATAACTTTTAACGATATGTGAACTTTCATAATAATAAACTACATATTTATGAAACGTCAAATTCTATTTCAATATATTTTAAAAAATAAA

>scaffold234.1.1628824

AAATAATTTTGAAATTTACTATCAAAATTTTTATAATGTCGTGTCAGAAATGGCATACTTTCATTAAGAATTAGAATAATTTGAAAAATTATTGTATAAG**[C/T]**GGATCAACAAATTATAGATTTCTGATGTATTGCAATTGGTTAATTCAATTGCATCAATTTTAAATTTAATGAATTTAATCACATAAAAATTGGTCAATTA

>scaffold234.1.477823

CTCTACACAATTATAAAATTTTATTAAAAATACAAAATTTTGTTCATTAATTTCATGAAAAAATAAAAAACTATTCAAATATTTTCATTTGAACCTATAA**[C/T]**GAAACAAAAAAATAAAAATATTTTTGTTTCTTTTTTTCACGATTTCTTTCAAAATAAGTAAAAACGAACAAATTTCTAAAGGACTTTTCTTTTATTGATT

>scaffold234.1.833025

TTTTTTCTCCTACATCATTTCTTTTTTTACATATGTATATGAGTATGTAAATATAATCATGTATATCAATATGTAAATATAATATAATATATATATGGCA**[C/T]**GATTTTTTTCATATAAAAGTAACATATTTTTTGTTACAAATATATCAATTGTTTATAGCAAAAGAATGGAAATATTAATCGTACAAAATGGAAAATTGTA

>scaffold234.1.1255768

TGAAATTAAGTATATAATGAAAATGAAGATTGATAACAGATAGCAGTAAAACAATCTCTCGAAAATAATTCTTTGTTAATAAGATTATATCAAACATTCA**[G/C]**TAACATTCAATACATTTTTATAAAGGAGGATTTATACAAATTTATACAAATTATTATACAAAAAAACAAATGTATCAATCGATACTATTTTTATCTAAAA

>scaffold234.1.1496913

ATAAATAAATGTTCAAATATTAATAAATAGATATAAATTGAATGTAATTGAATTTGATTAGATTATTGTTTTTGTACGATAATATTTATAATTATTTTTT**[G/A]**TTATTCTTTTAAAAATAATATCATATATTTGATTGTTGATTTGAATTTTAATAAAAAACATTATTTCTAAAATTGTTTTTTATTTCGCTAGAAAATAAAA

>scaffold234.1.1556360

TGAGGCGTCAAATTCTTTCATTTAATCATACTGAAATTTATTATAAATTATTTTTCTCATTTCCCGACAAATGAATATAAATAAAACTTTACTGTAAACC**[G/A]**TTGCCCGTGCGATTTTTCTCTTTTAACGAAAATAACGAATATCGATTTTCAATTAAAAAATATCTAAGTTAAAAATATAAGGAATAATAGAACATGATAT

>scaffold234.1.1595675

TAGATTATAAATGTTAGATAATTCTAGAATAAATGAGAACGATAAGAAATTAATAAAGTCTTTGTTTTTTTCACAATTTTTTTAATAATCTTCGTTAAAT**[G/A]**CAGGTCTACAGCCGATCATTTTATTTGTAAATTGGTTAATCGATGTGTTCTTTATTCATCAGTATGGAATATTCTATATTCCAAATTCATTAGTATTTCG

>scaffold246.1.633096

TTTTAAAATTCTTTTTATATTTTTTATTATGTACTAATTTTCTCATAAATTGGAAAGAGATTTGAAATTTTTACAATTCTTATAAGATAACATTTTCCTC**[A/G]**TTATTTGAATTTAAAAAATTTAACTTTTATTCAAGAATATAACTTGAATCTATAAATATCTAAAAAGATTATTAAAATATAATTTAATTTCGTTTACACG

>scaffold246.1.886461

GAACTACAATCCTACATCACGATGAACATAATTCATGTTGGTCTATGTGTCATAGTTATACTAATACATACACCTGTTCATACCAGAATTCGGTAAAAGC**[A/G]**TTTATGATCACATTGTATGTGTATTGCATTATGCTTAATCAATTTAATTTTCAATTTTTCCATAAGATATCGTTTTTATTATTTTTAAGATAAAAAATTT

>scaffold246.1.396276

CACAAGCAATTAGTTCGCATAATAAAAGCTCAACAACGAATTCGGTAAATTGGACATTCTATCGACATTAGCGCGCATAAATAAAGTTCAGATAAATAGA**[C/G]**TATCCTATTATAAATATAAATATACAAATTATAATGATTAAAGTATACGTCTTATTATGAAAATATAAAAGAATAATAATATTTTATGCTCAAAAATATA

>scaffold246.1.456724

CTGCATCCCTGCACTTTCTTCCACAGAACGCCACGCTGCTGCAATCCGGACATCCTATCGGAGCTTTGAATCTGAAAAACAAAATTGAAAATCAAAGTTA**[C/T]**GAAGAATAAAAAAAGAAAATAAATTTGGTTTTTTTTCGTTAGTCGTTGGTATATTAAGGTGTTGGAGGATTTAATCATTCTTTTTCTTGTTTTAAGTTTT

>scaffold246.1.555054

TTTTATAATCAAAAGTGTTATGTGATTTTGAAAATATAAAATCTGGATATGGTTTAGTTTTTACCTTTTCTACAGTGACTTTAGTGATTTCGATGTTTCG**[C/T]**AAATTTTTCTTATAATCAAAAATATTTTGATTATAAAAAAATACTATTAGATCATTTCGTGTAATTATCCAATTTTTATTTTTATAAGTGATCAAGTGAA

>scaffold246.1.882398

TAAGTATAATTGTATTTAAGAAAATTTGAAATAATGACAAATACTTTGAATTGAAACAATAAACAGAAAAATTTAATAAAATTATTTTTGATTATTTTTA**[C/G]**TTAACTTTTATTTCATAAAATTAATTGTAAATAAAAAAAATTTGATTTGAAAAGAAATTTCTATTTCACATTTGAAATACGATATTAAGTTTTAGAATTA

>scaffold246.1.113409

TACTATTGTAAAATCTAATATACTAACATTTATTTATTTATCTATGTATTTTTTTAATTATTTATTTTTACTTCTTCATATCTCAAATATAAAAATTTGT**[G/A]**CAATTTGTCAAATTTTTTTAATCTAGTTACACAACATATATTCATTTCTTTAAATATCGCTAATTTCTTTTGTATCTAAAGATTTCTTCAATTACTTTAT

>scaffold246.1.349495

CTTTACCGCTTCTTATATCAATTTATTTCACTTTCATTTATTTTTTTCTTATTTCTCTGATTTTCTTAACACGTATTCAGCTATTCGATCAATCTTTTTT**[G/A]**CTCTTCATATATAAGTTCTGAATGATTTATTCATTATTCGTTATTCGAATAAAATTTGCCCAACTCTGTAATAATCTCTGTAATGTAAAAATAAAATTTT

>scaffold246.1.501860

TTTCAAAATTTTCAAAAAATATTTTTTTTGTTTTGTTCCAAATTGAAAATAATAATATTTTTATTGTTTTTTTTTCTAATTTTTGATACAAAAATCTTGA**[G/C]**TAAACATCTTATTACACCGATTGATCAATCACTATTCTATTCTATTTTTAAAAAAAAATTCAATTGATATTTTCAAATTTTCATCTAAAAAAATTGAAAA

>scaffold246.1.635097

ATGTTTTAAAAAATAAAAATAAAATTTTCTTTTTTGCATGTTTCCTTATTTCTAATTATGTATTACATATGTAGCAATATGTAAGATGATTTTATTTAAC**[G/A]**TTATTTTATTTAATGCTTTATTCTAAAAATTCTAAAAACATATTTTGAATGATAATGAATTAATTAAATATTATTACACTATCTTTTATTTTATTACAGT

>scaffold246.1.939139

GAGTATGATGTGTTAATTCTTGCCACAAATGCCACTACTCAAATATGATTTTTGTTTCTATCGATAAAATATGTTTTTTATGTAATTTTCATAACAAGCT**[G/A]**TCGTTTTTATTTTATTTTTAATTTATGATTTGACTAGTAATAGAAAAAATCCAAAATAATTTTTTTATATTGATTAGAATATTTGAATACGATTTGTTAT

>scaffold246.1.102733

GTAGATATTAAAACAAATAAATAGATAATAAAAATATTCTAAACATTTGAAATAATAATATATTCTATTTAATATGTATAATATATAAAAGACAGTAAAA**[T/C]**GTAAAAAAAAATTGTTCAAAATATGTCTGAAAACATATTTCAAAGCCATCGAAAATCAATGATGAGGTCATCTCTTTTCTGCAAATAATTACTTAACATT

>scaffold246.1.110203

CATTAACACACTGTAGACAAATTCAGTGGGGACCATTCAGAAGGTCGCTACAATGGCATTCAATATCCTCGGTCAGTCGACGACTTTGTGTAGCAGCTAA**[T/C]**AAAATATATGTAGAAGAAATCAATGTGTTATTCACGTGAGAGCTATCGTTTTATATATATTGCTACAGAAATCACATGACCTAGAATAACGTTGCCAGAA

>scaffold246.1.295460

TGATTCATAGATGTTGTGATTTTATTTTTCGATTAATTGGAAAATGTGAGTTGCAATTTAAGTTTTGTTTGTTTAGTTTTTTCGTGAATAAGAAATTAAG**[T/C]**ATTAAGATAGATTATAAATTAAGAAAATTAAGATAAAATAATCAAAAATAAGAATTGTTGAAATCTGATTTTTTAGATTTGCGAAGCTCGAATCCGAAAT

>scaffold257.1.35543

CTTAAGTAATTACATTTATTAAAATTTTACAATAACAAGTGTTAAATAGTTAACTATTTCTCATAATATGATATTCAATAAATAAAGCAAATATCCAGCT**[C/G]**ATATTTAAATAATCATAATAACACAAATTAAATTCACTCAGCATTATCCCATACAGAAGTTCATTGATCCGTGGGGCACAATAATACACGTCCCGCTTTT

>scaffold257.1.36146

GATAGGAGCATGTTCGAACAGTATTTAGATAGATTTTATTATGCGAATGATGGTAGTTAATTGATATGAAATGTGTAGATAGTATATGTTGATTGTACAG**[T/C]**GATCATGTTAATTAAAACGAGAAATAATTATGAATTATTCCGTAACATTTCGATTTCTGGGGGTATTATAACATTGGTTCATAAGTGTTAAGGAAAGGTT

>scaffold282.1.11069

TTTTATCATATTGATAGTAGAAAATTGATAAATAAGATTTCTATGATATTTTGATTAGTGTCAGAAAAAAATAAATTTCCTTCATATATTTATAAACAAA**[C/T]**AATTTTTTTCTTTTTTTATATTTTCAAGAAGAGTAAAAATTAATTAAAGAAAAAAGAAAGTAAAGAATAATGAATGAAAATTTAATTCAAAGATTAATTT

>scaffold282.1.258968

CGATCGATATCAATTAATTCAACAAATATTTTAGCATTTCTGGCAATACTGGAGCAAAAAATATTTACAACAGTTATAACATCGGTCAATAGGCACCTTG**[C/T]**AAGAAAATGATCTGGTAATAATACGAGAAGAAAACCTTCCTTCTCCACAATAGCGCATGAAAATACTGATGCAACTTCATATTGGAACAGACGCGCGTAG

>scaffold289.1.304925

TACCTATCTAGTCTTATATTTATAAAATAACAAAATAAGCACACATTCATTCAATGATTCTCACGCGTAACACTTGAATGCAAAAGTCTATTAGAAAAAA**[C/T]**GTCTCAGGATATGTGTGATATCCGAATTTGGTACACTACTTTCATACTGTAACGTACCATTTTTCTGAAATTGATTGATGATGGCTGAAGACGTAAAAGA

>scaffold289.1.7774

TTAATTTAATCTTAGTTGAATTGACTTTTTCTTTTTTTTTGAAAATTTAACCCATCGTTAAATTGTTTCAAATTTCGTTCAACAAATTAATCGAATTAAA**[C/T]**AAATTTCTTGAATATAAATCAGCCTTTTGAATAGATTTTGTTTTATTGCATGAATACACGCGAGAGTTGATTAGAAAGTAGAAATGAAAGTAAAATTCAT

>scaffold289.1.8075

GATCGATTTTGAATTCGAAATTGTTTATAGTAAAATGAACATCTTCAAAAGTTTTTTCGGTGAAAGAAATTGGAAATAAACAAGTATGGTGGATAATTAA**[C/T]**AACTAGTTCAACTTTAATTTCTCTAAAGTTATATCGTGTTTGAAAAAATTAATGATAAATCAATTAAATTAAATATTATTAATTAGGCTATTTTATAACT

>scaffold289.1.103127

CATTCAATAATTTTCTTGCATCCGTTTCTCCTTAGATATTTTAAATGTAAATAATTCGAATAATCTAAACAAATGATTAATTCACTAGTGAAGAGATCTC**[G/A]**TATCGATATATTCAAACTCGTAATTTTTATTATCTAATTATCATTATTACCGGCTTTTCTAATCAAACGAGTCGAAAATGACCCAACCGCAATGTTTACA

>scaffold296.1.83083

AAGTCTATTAAAGCTTTTTTCATATTTGTCTGTATAATAATTGCAATATTGTTTTATTAATTATATTTTATTGTTTGTGCAAGCAAGTTAAAGCATGTGA**[C/T]**GAGTTTAAGAATGTAACTAACTATTTTGTTTCATATATATCTCTAAATTTAATTGATATTGATACCTAATTTAATGATATGATGATCGAAATTATCAATT

>scaffold296.1.12024

GCCTTGAATATTGCGACTATTTGTAATAATATGAAAAAAATGTTATACTTGCATTGCATAGAGAAAAGTATATTGTGATATGAATATTTTTTGCGTAAGT**[G/C]**AAAGCATAAAAAAGATTTCAAGCTTATCTCTATTTTTTTAAATAAAATTCTATGTTTTTTTTATGTAATAACTAATGTTGAAAAGACTTCAATCATTGAA

>scaffold313.1.66976

GCTCAGATCGAAGAATAGGATAGATTTCCAATTTCGGGCGAACATCTTAGCAAAATGACACTTGGAAGAGCATTATTACAATAGTTTATAAGAATGACTC**[G/A]**CTTATTTTTGTCAATGGATATCAAATATATATTTTCGCTAAAATATTGAAATGATTTATTATAATAATGGATAGGAATGTCTCGTTCATTTTTGTCTGTC

>scaffold316.1.12333

AAATGACAAAAATGTTTATAAAAGTTTTTATAAACATTATTATTATAAACATTAACATTATAAACATTATTTTATAAAAGCATTTATTAGAGATGTTTTG**[C/A]**TTATAACTAATGTCTCTTTCAAAATTAATTAGGCTTAATTTTGTTTTTTTTGATTTTCGAAATTCCATTATTTGTGAAAGAGAAAAGAGAAAATATGCGC

>scaffold330.1.47155

AATTTATTTCACAAAATAATTTTCAAAATTGTTTTTTTGAAGATGAACAATACATTCCTTTTTTCAAAGGAAATATTTTAACATTGTAATTTTACCACAA**[C/T]**GAATTCGCTAACTTTATTGTCAAATCTCGTATTTAACATATTTTCTATCGCATTAAATTAAAATAAAAAATTAAATCTAAAATTTTTATTAAAAAATATA

>scaffold330.1.92646

ATATTTACAGATGTAGAATATTAGAACTTTGTTATTTTAGAGTTATTAATATTTATTAAATACCAAATAAACTTTCTTATATTTAAAAATATCTAAATAA**[G/T]**AAATGTCAACAAAATTTTAAATAATAACATTAAGTTAAAAACTATTATTAAGTTAAAAACCATTATTCAATTACATATTTTTATTTTACTCTCAAAAATA

>scaffold372.1.7844

ATATATTCATAAATTTATTTAACTATTGTTTGTTTAATTATTTATATATTTAATCAATTATTTGTTTAATGACTTATTTGCAGACTTTTATATTTTATTT**[A/C]**TTTATATATGCATCAAATTATTTATTTCTTTATTTTCTTATTTATTTAAATATATATATATATATGTATATATTTATTCATTCATTTGTTTTATTAATGA

>scaffold418.1.966

TGTTATTATATTTGTTTATTACTTATATTGCAACATTTTATTCTTTTTTATATGTTGCAACACGTAAATTGCATAATATATTGAACCTAACAAAACCTAA**[C/T]**AAATATGCAACATATAAAAAGGAATTTTCAGCATATAAAAACATAGTCATAATTCAATTAATACAAAATTGATAAAAAATAAAATGAGAATAAAGTGAAA

>scaffold423.1.204

AAAACAAAAAAGTTTGCTACAATGTTTATAACTAAATCATTTAGTAAACAAAGAAATAAATTAAATGATAAGTAATTAAACAAAAGAATGACATTTGCAG**[C/T]**AATGTTTATAATTTATTCATTAAAAAAATATATAAATAAATAAATAAATAAATTGATGTATAAATGCAAAAGAGTATAATTTTAAAAATACTACTATTAT

>scaffold445.1.364

TCTTCATCAAAACATGTTTTCGAAAAACATATAAAACTAACGTTTAAAATTTCTTGCTCTGATTCAGAAATCTACTTGTCGCGAAGCATCTTCTTGCACG**[T/C]**ATTTCAAACATTTTTTTACGGAGAAAATAATTTTACATTTAAAATCATAAGTTTGTGACAAAAAATCAAATAATTAAATATTACAATAAAATCAAAGATA
